# Taxonomic and functional characterization of a microbial community from a volcanic englacial ecosystem in Deception Island, Antarctica

**DOI:** 10.1038/s41598-019-47994-9

**Published:** 2019-08-21

**Authors:** Emma Martinez-Alonso, Sonia Pena-Perez, Sandra Serrano, Eva Garcia-Lopez, Alberto Alcazar, Cristina Cid

**Affiliations:** 1 Department of Investigation, Hospital Ramón y Cajal, Instituto Ramón y Cajal de Investigación Sanitaria, 28034 Madrid, Spain; 2 Microbial Evolution Laboratory, Centro de Astrobiologia (CSIC-INTA), 28850 Torrejón de Ardoz, Madrid, Spain

**Keywords:** Microbiology, Environmental microbiology, Microbial communities

## Abstract

Glaciers are populated by a large number of microorganisms including bacteria, archaea and microeukaryotes. Several factors such as solar radiation, nutrient availability and water content greatly determine the diversity and abundance of these microbial populations, the type of metabolism and the biogeochemical cycles. Three ecosystems can be differentiated in glaciers: supraglacial, subglacial and englacial ecosystems. Firstly, the supraglacial ecosystem, sunlit and oxygenated, is predominantly populated by photoautotrophic microorganisms. Secondly, the subglacial ecosystem contains a majority of chemoautotrophs that are fed on the mineral salts of the rocks and basal soil. Lastly, the englacial ecosystem is the least studied and the one that contains the smallest number of microorganisms. However, these unknown englacial microorganisms establish a food web and appear to have an active metabolism. In order to study their metabolic potentials, samples of englacial ice were taken from an Antarctic glacier. Microorganisms were analyzed by a polyphasic approach that combines a set of -omic techniques: 16S rRNA sequencing, culturomics and metaproteomics. This combination provides key information about diversity and functions of microbial populations, especially in rare habitats. Several whole essential proteins and enzymes related to metabolism and energy production, recombination and translation were found that demonstrate the existence of cellular activity at subzero temperatures. In this way it is shown that the englacial microorganisms are not quiescent, but that they maintain an active metabolism and play an important role in the glacial microbial community.

## Introduction

**Figure 1:**
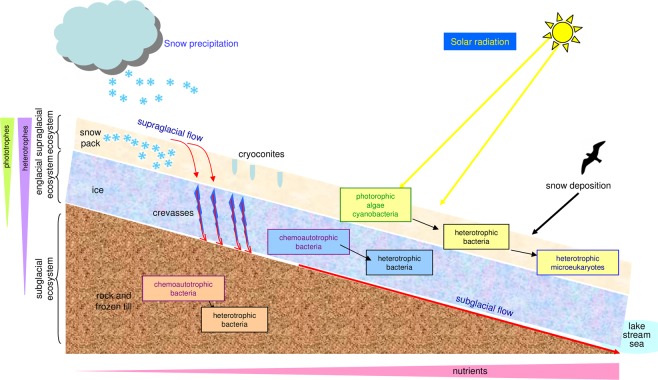
Ecosystems of a glacier colonized by microorganisms. The **s**upraglacial ecosystem is inhabited by photoautotrophic microorganisms, and by heterotrophic bacteria and microeukaryotes that feed on organic particles from atmospheric deposition. Microorganisms in the englacial ecosystem can be chemoautotrophs or heterotrophes that feed of solubilized products. Subglacial ecosystem contains a majority of chemolithotrophic microorganisms that are fed on the mineral salts of the rocks and basal soil and occupy ice/till veins.

Ice from glaciers occupies approximately 11% of the Earth’s surface^1^. Although glaciers were traditionally considered to be an uninhabitable environment, it has been proven that they are populated by a large number of microorganisms including bacteria, archaea and microeukaryotes^2,3^. Among them, microorganisms inhabiting the Antarctic glaciers are much more unknown than those living in other frozen environments^4^. Three ecosystems can be differentiated in glaciers: supraglacial, subglacial and englacial ecosystems^5–7^ (Fig. 1).

Each ecosystem experiences different environmental conditions including temperature, solar radiation and nutrient content^8,9^. Microorganisms from the supraglacial and subglacial ecosystems have been the most widely studied. Among the three layers of a glacier, the upper layer (supraglacial ecosystem) is the most habitable, due to the atmospheric deposition of dissolved organic carbon, besides the plant and animal remains, and the particles and black carbon from atmospheric dust^10^. The supraglacial ecosystem is largely populated by autotrophic microorganisms exploiting the ample solar radiation and liquid water, and abundant nutrients available during ice-melt episodes^11–13^. The subglacial ecosystem is dominated by aerobic chemoheterotrophs and by anaerobic methanogens, nitrate reducers and sulfate reducers^14–16^ in basal bedrock and subglacial lakes. As there is no sunlight, these microorganisms obtain energy from the inorganic compounds originated from weathering and erosion of subglacial rocks^17,18^. Little is known about the persistence, physiology, and ecology of microbial communities inhabiting the englacial ecosystem. It has been reported that microorganisms enclosed in ice present very low metabolic rates, using energy only to repair damaged biomolecules, and not to grow and reproduce^19^. More research is necessary in order to define their importance^20^.

**Figure 2:**
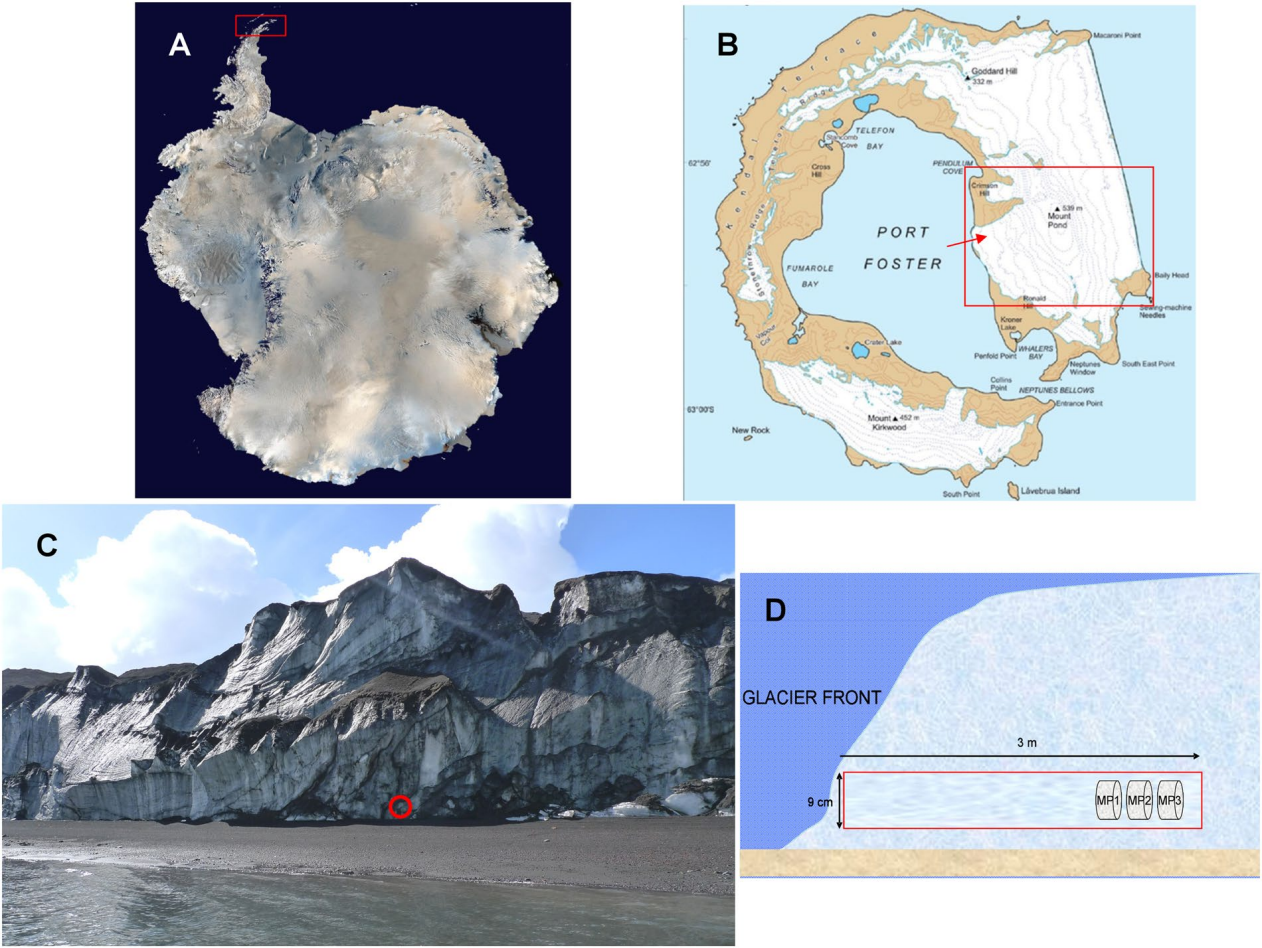
Geological setting of Mount Pond Glacier, Antarctica. (**A**) Map of Antarctica showing South Shetland Islands (Credits: NASA, http://visibleearth.nasa.gov). (**B**) Map of Deception Island referencing Mount Pond glacier and sampling point (red arrow). (**C**) West front of Mount Pond Glacier and sampling point (red circle). (**D**) Schematic representation of the sampling site.

Mount Pond Glacier is located on an island of volcanic origin, Deception Island, South Shetland, Antarctica (Fig. 2). Volcanic eruptions have caused the black color of glacial ice, which contains remains of lava and ash. These elements constitute an important supply of materials that are included in the ice, and form the small niches where microorganisms are housed. The products of the volcano in Mount Pond consist of pyroclastic rocks, lava flows and scoria^21^. The main volcanic materials are basalt (rich in Mg and Ca, and in alkali oxides of Na and K) and olivine (magnesium iron silicate). All these chemical elements are abundant in the englacial ecosystem (Table 1) and can be used by microorganisms. Microbial activity may play a significant role in the chemical exchange between basaltic rocks and glacial meltwater. The significant amounts of reduced iron in these minerals provide potential energy sources for chemolithotrophic bacteria. Furthermore, the organic matter contained in ice, that can be available for microorganisms, comes from organic remains dragged by the glacier, and from other dead microorganisms transported vertically^22^.

**Table 1:** Chemical analysis of ice meltwater from Mount Pond Glacier.

Sample	pH	Salinity (ppt)	NH_4_^+^ (mM)	NO_2_^−^ (mM)	NO_3_^−^ (mM)	TDN^a^	SRP^b^	Fe (ppb)	Mg (ppb)	S (ppb)	Ca (ppb)	Cl (ppb)	Mn (ppb)	K (ppb)	Na (ppb)
MP1	4.50	0.18	2.10	3.30	4.86	187.99	0.42	3.57	104.40	6733.10	79.20	113051.45	1.81	93.13	671.46
MP2	5.51	0.27	2.57	2.67	5.18	190.24	0.39	3.30	110.39	7845.21	80.23	125478.32	2.31	102.36	874.21
MP3	5.58	0.31	1.98	2.90	4.40	191.35	0.55	2.90	120.69	5698.19	85.26	136578.11	7.33	77.54	547.33
Average	**5**.**20**	**0**.**25**	**2**.**22**	**2**.**96**	**4**.**81**	**189**.**86**	**0**.**45**	**3**.**26**	**111**.**83**	**6758**.**83**	**81**.**56**	**125035**.**96**	**3**.**82**	**91**.**01**	**697**.**67**
(SD)	0.49	0.05	0.25	0.26	0.32	1.71	0.09	0.28	6.73	876.71	2.65	9609.81	2.49	10.24	134.73

^a^TDN: Total dissolved nitrogen.

^b^SRP: Soluble reactive phosphorus.

Several techniques have been used to identify the microorganisms that inhabit the Antarctic glaciers. Traditionally, microscopy techniques^23^ were used, subsequent attempts were made to culture^24^ and isolate the microorganisms^25^, while 16S and 18S rRNA sequencing techniques have more recently been used^26^.

So far, the temperature limits at which microorganisms in ice present an active metabolism had been studied by several methods, including the incorporation of radiolabeled compounds, measurement of ATP and ADP concentrations, measurement of nitrate and ammonium, etc.^9–27^. Metabolic networks in other microbial communities have been elucidated by metagenomics^28^ and metatranscriptomic approaches.

Previously, few studies have focused on the biogeochemical cycling in the englacial ecosystem. Although it had been traditionally reported that most of the microorganisms found by sequencing were not cultivable^29,30^, some researches have demonstrated that culturing samples in several culture media^24^ and their subsequent functional genomic and metaproteomic analysis^31^ are possible. This can allow both the identification of proteins involved in microorganism metabolism, and the identification of microorganisms that take part in biogeochemical cycles inside the glacier^24^.

Functional culturomic and metaproteomic analysis are ideally suited to englacial ecosystem studies for several reasons. Firstly, species that have not yet been recognized by 16S rRNA sequencing can be identified by the sequences of their proteins, and moreover, it is a way of verifying that microorganisms are alive and have the capacity for metabolism and growth. Taking into account that proteins are synthesized only when they are required, the detection of whole essential proteins is an indicator to detect living cells. Secondly, culturing can also increase the abundance and activity of microorganisms, enabling metaproteomic studies to model the structure of the potential microbial community. In addition, the knowledge of the proteins used by these microorganisms makes it possible to find out their metabolic pathways and how microorganisms are able to survive in this extreme environment.

In this work, we have provided new results through 16S rRNA sequencing, culturomics (a culturing process that uses multiple culture conditions) and metaproteomics. Several interesting questions are addressed: (i) what are the microorganisms that inhabit the englacial ecosystem?, (ii) are they alive or quiescent?, (iii) if these microbial communities are alive, what are their survival mechanisms, their physiology, and their ecology? This study details a strategy for the identification of the englacial microbial community from an Antarctic glacier combining genomic and metaproteomic techniques.

**Figure 3:**
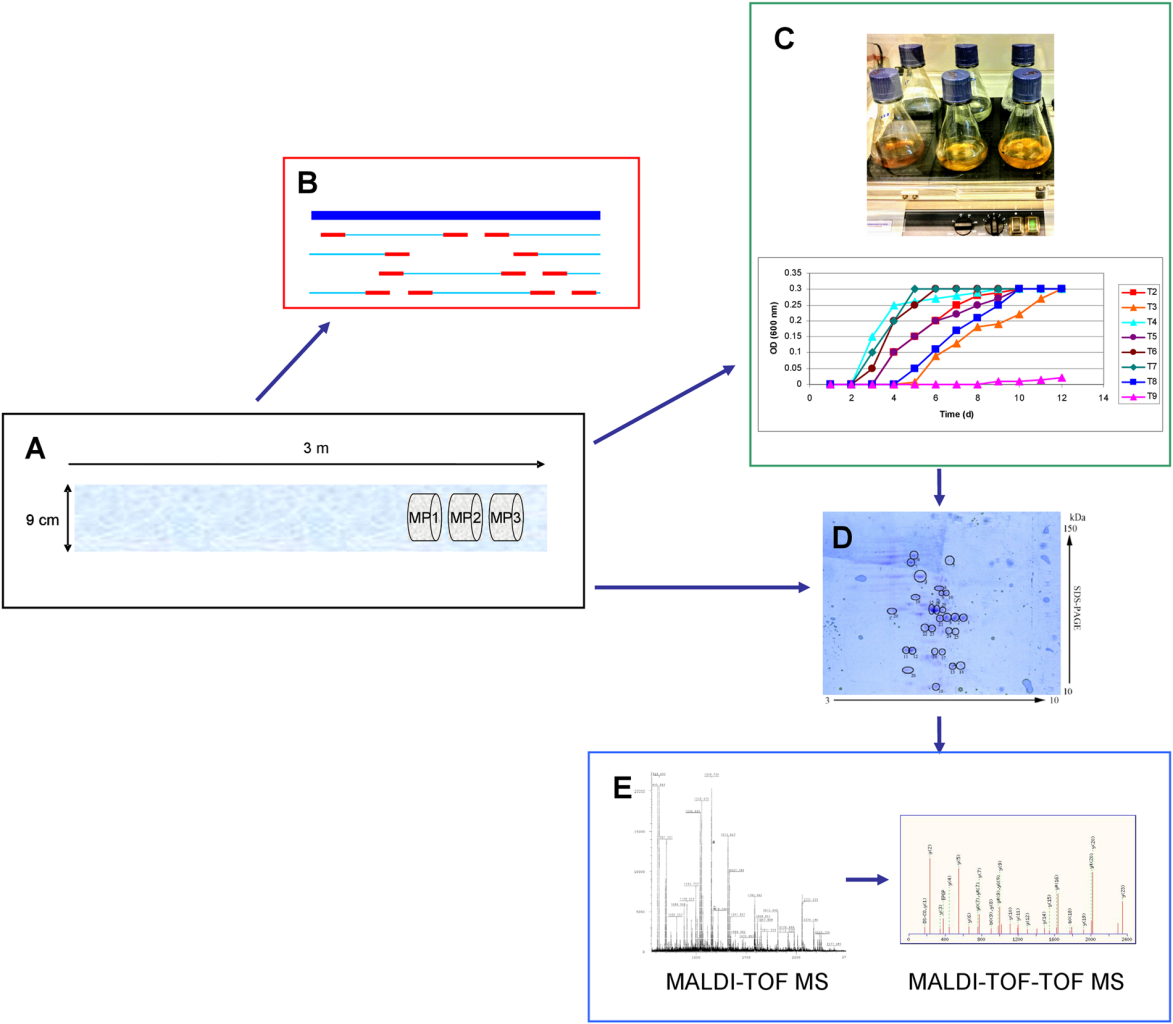
Summary of the overall experimental strategy. (**A**) Sampling (3 direct ice samples: MP1, MP2 and MP3). (**B**) 16S rRNA gene sequencing. (**C**) Cell cultures and growth rates were monitored by optical density at 600 nm. (**D**) Coomassie stained 2-DE gels. (**E**) MALDI-TOF and MS/MS fragment ion spectra.

Firstly, samples from the innermost end of an ice core from an Antarctic glacier were analyzed by a 16S rRNA gene survey in order to identify microbial species. Secondly, microorganisms were cultured in various culture media and at various temperatures. Lastly, metaproteomic techniques were used to verify that englacial microorganisms present an active metabolism and to elucidate their potential metabolic routes. A summary of the overall experimental strategy is represented in Fig. 3.

## Results and Discussion

### General characteristics of the ice samples and chemical properties

The physical parameters, as well as the chemical composition of water from the thawed ice (Table 1), were taken into account to prepare culture media that could imitate the meltwater composition. The temperature of the samples (−3 °C) was not very low compared to samples we had collected from other Antarctic glaciers at the South Shetland archipelago such as Livingston, Greenwich and King George Islands (data not shown). This is due to several reasons: firstly, the samples were collected during the austral summer. Secondly, this glacier is located on a volcano that produces heat, which partialy melts the ice and produces liquid water in veins between ice channels. Finally, the englacial ecosystem supports high pressures, which increase the temperature. The pH in our samples (5.20 ± 0.49) (Table 1) is lower than the values found in the englacial ecosystem in other glaciers (between 6 and 11)^5,11^, and more similar to those recorded in the supraglacial (average 5.5)^5,32^ and subglacial ecosystems (average 5)^33^, which tend to be more acidic. The salinity of samples (0.25 ± 0.05) is similar to values reported for the supraglacial ecosystem^32,34^ and lower than published results from the subglacial ecosystem^17,35^. The low nitrogen and phosphorus concentrations quantified in our samples (Table 1) demonstrated that the englacial ecosystem is as oligotrophic as the melt pools on Arctic pack ice^32^.

Glaciers worldwide are very different from each other depending on several factors such as temperature (cold, temperate or polythermal glaciers), glacier setting (polar or high mountain glaciers) and the sea proximity (continental or marine glaciers). The type of glacier that we have studied only provides partial information, but it is very representative of other glaciers in volcanic areas such as Kamchatka or Iceland^33^.

### Microbial diversity in the englacial ecosystem

#### Microbial diversity in the englacial ecosystem inferred from 16S rRNA gene amplicons

**Figure 4:**
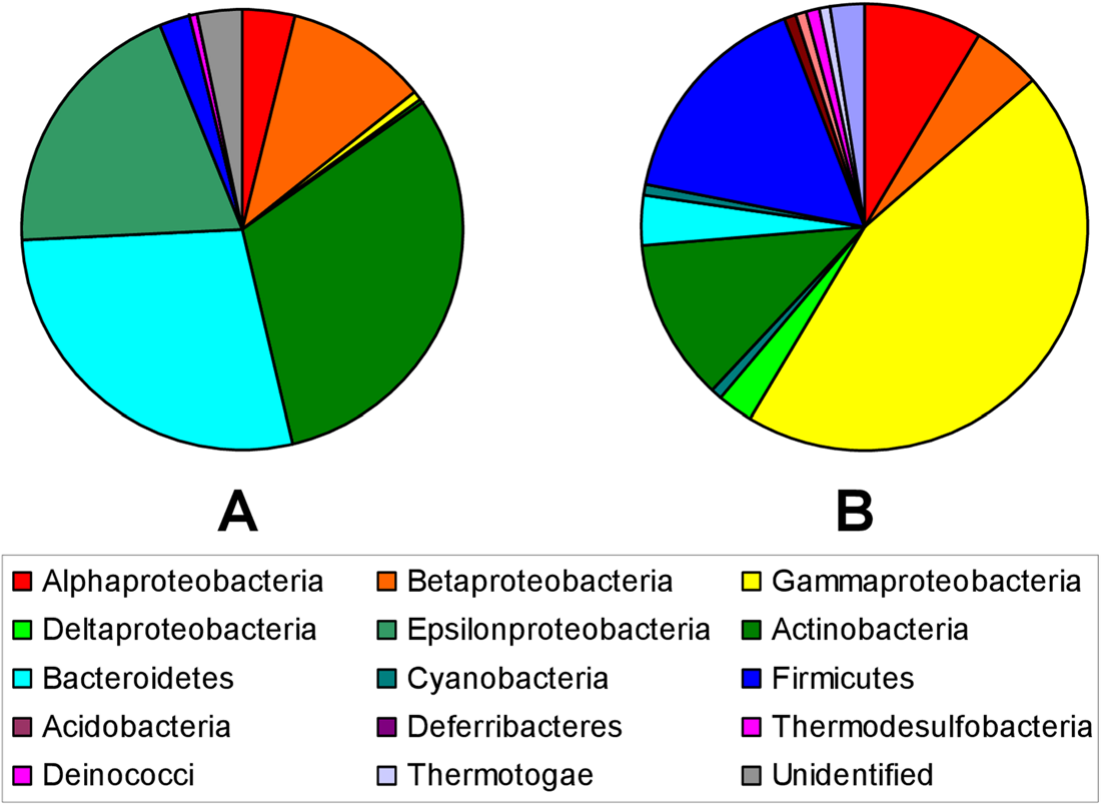
Microbial composition in the englacial ecosystem in Mount Pond Glacier. Pie charts represent relative abundances of major taxa of bacteria in the englacial ecosystem in Mount Pond Glacier, based on (**A**) 16S rRNA gene sequencing and (**B**) proteomics data.

In order to know the microbial communities that populate the englacial ecosystem, the V3 and V4 regions of the 16S rRNA gene were sequenced by Illumina MiSeq (Table S1). A total of 359992 reads were obtained which belonged to 738 OTUs spanning 17 phyla. Microorganisms mainly corresponded to the phyla Actinobacteria (30%), Bacteroidetes (27%), Cyanobacteria (19%), Proteobacteria (15%), and Firmicutes (2%) (Fig. 4A). Other phyla such as Acidobacteria, Deferribacteres, Thermodesulfobacteria, Deinococci and Thermotogae were scarce.

Some of the identified bacteria (such as *Polaromonas* and *Cryobacterium*) have been described as being psychrophilic and psychrotolerant. Other microorganisms identified (i. e. *Caldanaerobacter*, *Thermomonas* and *Thermosulfurimonas* have been catalogued as mesophiles^36^ and even thermophiles^37–40^. The presence of these microorganisms can be due to the heat given off by the active volcano of Decepcion Island. This same phenomenon has been found in other cold and geothermal areas such as Iceland^41^.

#### Microbial diversity in the englacial ecosystem inferred from cultured-metaproteomics

**Figure 5:**
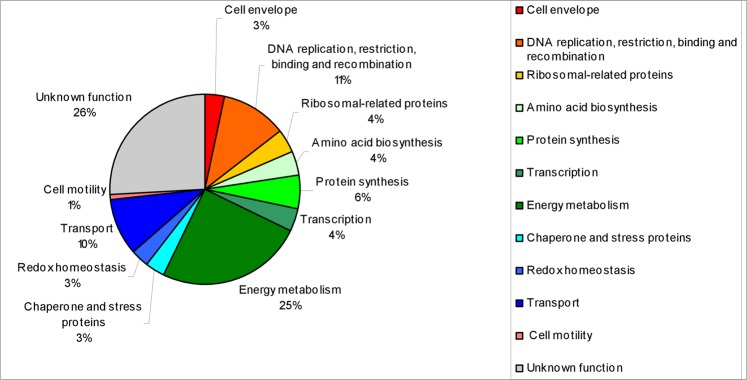
Functional classification of proteins identified in gels from cultures.

Culture conditions for cultured-metaproteomics are summarized in Table S4 and Table 2. Uncultivated meltwater samples were named T1. Culture conditions were named: T2, T3, T4, T6, T7, T8 and T9. Cultured cells were lysed, and proteins were separated by 2-DE (two-dimensional electrophoresis). 168 spots were detected in protein gels, which corresponded to 124 different proteins (Table S2). These analyses allowed the identification of 26 OTUs of microorganisms in the englacial ecosystem that were not found by 16S rRNA sequencing (Table S3). Among them, 22 OTUs belonged to Bacteria, 2 belonged to Eukarya and 2 were Archaea (Fig. 4B). Most of the proteins could be identified by matrix-assisted laser-desorption/ionization time-of-flight mass spectrometry (MALDI-TOF MS), but some of them were hypothetical or unknown (Fig. 5). Determination of function for hypothetical proteins is a challenging problem. Some methods have been developed for predicting protein function using the information derived from the comparison of a hypothetical protein sequence with other sequences from one or more well- known proteins^42^. Nevertheless, this approach should be confirmed with different techniques such as function assignment based on 3D structures, protein-protein interactions, protein complexes, comparative genomics and phylogenetic profiles^43^.

**Table 2:** Culture conditions for microbial culturomics characterization from the englacial samples of Mount Pond Glacier, Antarctica.

Culture conditions*						
Culture media (g/L)		Temperature (°C)				
		−4	0	4	20	28
T1	−	−	−	−	−	−
T2 (20x)	5 g peptone, 0.15 g ferric ammonium citrate, 0.2 g MgSO_4_·7H_2_O, 0.05 g CaCl_2_, 0.05 g MnSO_4_·H_2_O, 0.01 g FeCl_3_·6H_2_O	~	+	~	−	−
T3 (20x)	1 g glucose, 1 g peptone, 0.5 g yeast extract, 0.2 g MgSO_4_·7H2O, 0.05 g MnSO_4_·4H_2_O	~	+	~	−	−
T4 (20x)	1 g glucose, 0.5 g casamino acids, 0.5 g yeast extract, 1 g KH_2_PO_4_, 0.5 g CaCl_2_·2H_2_O, 0.5 g MnCl_2_·4H_2_O	~	+	~	−	−
T5 (20x)	R2A 20×	~	+	~	−	−
T6 (1x)	R2A	~	+	~	−	−
T7 (20x)	Marine broth 2216	~	+	~	−	−
T8 (20x)	Trypticase soy broth	~	~	~	+	−
T9	Ice meltwater	−	−	−	−	−

*3 replicates were cultured with each culture condition. T2-T6^107^, T7-T8^98^. Growth was monitored by optical density at 600 nm 12 days. Cultures achieved absorbance values of 0.3 (+), 0.1 (~), 0.0 (−).

In our experiments, culturomics experiments using several culture conditions were performed. Culture media were prepared according to the composition of the melted ice. These culture media had been tested previously^26,44^. T1 samples were obtained directly from meltwater (Table 2 and S2). Therefore, it likely reflects the microbial populations living in the glacier. Proteins found in these electrophoresis gels were related to metabolism and energy production (e.g. isocitrate dehydrogenase), to recombination (e.g. recombinase) and to translation (elongation factor Tu) (Fig. S1, Table S2). A vast variety of proteins (12, 10%) found by metaproteomics were ABC transporters. Powered by ATP hydrolysis, ABC transporters mediate translocation of several molecules across membranes, and take part in the regulation of several cellular processes^45^. Proteins found in gels from glacier samples (T1) were related especially to Proteobacteria, Actinobacteria and Firmicutes (Table S2). Some of these microorganisms such as Firmicutes are able to form spores, which constitute a survival mechanism in environments where water availability is scarce^46^. Several proteins from the methanogen archaea (*Methanobrevibacter*) were also identified by proteomics in T1.

The remaining samples (T2–T8) were obtained from cultures. They show that microorganisms are able to grow and which are the fast growers. In addition, the metaproteomic analysis of the samples from cultures allowed the study of their metabolic potentials. In T2, a culture media that contained ferric salts, proteins involved in iron metabolism were identified (Table S2). Spots 16 and 28 were identified as Malonyl-CoA O-methyltransferase from *Geobacter*, and Ferritin from other Geobacteraceae bacteria respectively. Other Bacteria, *Pseudorhodobacter ferrugineus* also grew in these conditions. This bacterium is capable of growing with sulfide or S^0^ as electron donor, and it is also able to use reduced iron ions. T3 is a rich media in which many OTUs (38, 31%) were found, not only Bacteria such as Firmicutes (*Bacillus*), Actinobacteria (*Corynebacterium*, *Actinomyces*, *Streptomyces*, *and Mycobacterium*), and Cyanobacteria (*Synechococcus*) but also eukaryotes (*Phytophthora* and *Giardia*). In T4 only three bacterial OTUs were recovered, and two of them were fermenters (*P*. *fermentans* and *Clostridium*). The third one was *Petrotoga mobilis*, a thermophilic bacterium that had been isolated from the hot oilfield water of a North Sea oil well^47^. In T8 at 20 °C, some mesophilic Bacteria were identified, i. e. *Klebsiella*, and some of them were fermenters (*Serratia*) or acetogenic (*Acetobacter*). T5 and T6 are rich media, in which most of the OTUs were recovered. Lastly, T7 promoted the growth of marine species such as *Salinispora*, *Halomonas*, *Marinobacter* and *Pseudorhodobacter*. These species could come from sea salt aerosols that are transported from the coast to the front glacier^48^.

The best growth temperature was at 0 °C for all culture media except T8 (Table 2; Fig. S2). In T8, cultures grew better at 20 °C. At −4 °C, cell growth was very scarce, although it has been reported that there may be cell growth, or at least DNA break repair at much lower temperatures, down to −15 °C^27,49^. In this research, cultured-metaproteomics have found few proteins from eukaryotes. Probably, these culture conditions are not suitable for eukaryotes.

### Proteome-inferred microbial activity

Previously, biological activity at low temperatures in microorganisms had been investigated by indirect methods such as the reduction of the chemical compound 5-cyano-2, 3-ditolyl tetrazolium chloride (CTC), which had been used to detect cellular respiration^50^; or the incorporation of ^3^H-leucine, applied to study protein synthesis^50,51^. Reports have also been published on methanogenesis, carbon fixation, metagenomics and metatranscriptomics studies, etc.^52^. Here, enrichment metaproteomes were generated to detect an array of active microbial metabolisms (e.g. energy metabolism, protein synthesis, and adaptation to stress).

Most of the identified proteins belonged to the Gammaproteobacteria *Pseudomonas* (Table S2; Fig. S3). These bacteria are chemoorganotrophic and aerobic, having a metabolism with oxygen as the terminal electron acceptor. The classical reactions of the tricarboxylic acid cycle were detected in all the species of *Pseudomonas* inhabiting the glacier. Several proteins (Table S4) involved in the tricarboxylic acid cycle were identified i. e. isocitrate dehydrogenase (Fig. S3, spot 1) or succinate-CoA ligase (spots 2 and 14), acetyl-CoA acetyltransferase (spot 29) and ketol-acid reductoisomerase (spot 24). Dihydrolipoyl dehydrogenase (spot 98) is a component of the glycine cleavage system as well as a component of three alpha-ketoacid dehydrogenase complexes^53^. Other proteins found in *Pseudomonas* were ATP-binding cassette (ABC) transporters, membrane proteins that translocate amino acids (spots 18, 36, 56, 61, 79 and 90) and sugars (spot 78), either out of or into the cytosol. When bacteria experience a lack of nutrients as phosphate limitation, they synthesize proteins such as PhoH (spot 33), which are involved in the transport and use of various forms of combined phosphates or free phosphate^54^. Further proteins found in *Pseudomonas* were related to amino acid metabolism such as isovaleryl-CoA dehydrogenase (spot 3), arylsulfatase A (spot 27) or glycine cleavage system T (spot 38). Certain amounts were RNA polymerases as RpoA (spot 114)^55^ or were involved in the protein synthesis: elongation factors (spots 15, 35, 91) and ribosomal proteins (spot 30). In prokaryotes, DNA is segregated by cytoskeletal filaments^56^. ParB proteins (spot 66) are found only on plasmids and take part in the DNA segregation process^56^. Additionally, several important proteins involved in the cellular stress response were chaperone proteins GroEL (spot 4) and DnaK (spot 6). Typically, *Pseudomonas* cells are motile by one or several polar flagella. Protein BAKDH, (spots 56 and 120) is involved in flagellum organization.

Other Gammaproteobacteria found in the englacial ecosystem was *Halomonas* (Fig. S3). Proteins from *Halomonas* corresponded to the ABC transporter system (spot 41) or to the stress response system. Spot 121 was identified as the dodecin domain-containing protein. This protein had been previously found in Eukaryotes and Archaea^57^, but it had not been described in *Halomonas* and its role in Bacteria is not clear^57^. Dodecin is responsible for the flavin homeostasis of the cell^58^. The crystal structure of the NADH: FMN oxidoreductase has revealed that FMN dimers bind in a similar way as those found in the bacterial dodecins^59^. This protein is involved in the resistance to environmental organic pollutants. Furthermore, in *Halomonas*, the hypothetical protein found as spot 43 is similar to the 50S ribosomal subunit-associated GTPase HflX^60^. This protein regulates a broad range of ribosome-associated processes, from the assembly of ribosomal subunits to the control of the ribosome in translation.

Proteins found in the Deltaproteobacteria *Geobacter* (Fig. S3) were malonyl-CoA O-methyltransferase (spot 16) involved in the biotin biosynthesis^61^, and ferritin (spot 28) with a function of iron carrying, storage and detoxification^62^.

Some *Bacillus* species have an unexpected potential to produce secondary metabolites and to activate stress responses. The protein polyketide synthase (spot 40) is involved in some intermediate steps for the synthesis of the antibiotic polyketide bacillaene^63,64^; PadR (spot 123) functions as a transcriptional repressor that inhibits gene expression associated with stress responses^65^; and MoxR family ATPase (spot 17) takes part in a chaperone system that is important for the folding/activation of proteins and protein complexes^66^. Hypothetical protein N399_09015 is very similar (93%) to type II toxin-antitoxin system PemK/MazF family toxin of *Aneurinibacillus terranovensis*. This protein belongs to a molecular system that allows bacteria to cooperate and compete with one another in the environment^67,68^. Hypothetical protein WP_088074644 (spot 59) is very similar (97%) to TonB-dependent receptor of *Gelidibacter algens*. It takes part in a protein system that facilitates iron translocation into the periplasmic space. Two other proteins are involved in cell division and sporulation: FtsI (spot 111)^69^ and N-acetylmuramoyl-L-alanine amidase (spot 92)^70^.

Metaproteomics identified several proteins from Actinobacteria. In *Mycobacterium* (Fig. S3), the mycolic acids are important components of the cell envelope. Class I SAM-dependent methyltransferase (spot 62) catalyze the introduction of chemical modifications in defined positions of mycolic acids^71^. Another protein, MetH (spot 94) catalyzes the B12-dependent synthesis of L-methionine, utilizing L-homocysteine and 5-methyltetrahydrofolate as substrates and generating tetrahydrofolate as a co-product^72^. The rest of the proteins from *Mycobacterium* found in gels are the hypothetical proteinWP_067770147 (spot 88), very similar (94%) to translation initiation factor IF-2, which guide the ribosome in the selection of mRNA in the initial steps of protein synthesis^73^. One more hypothetical protein found in *Mycobacterium* was SHY74220 (spot 84), which is similar to sugar-binding protein of other Actinobacteria, *Amycolatopsis keratiniphila*. This protein takes part in the transport of sugars across the cell membrane.

In other Actinobacteria, *Streptomyces* (Fig. S3), MerR (spot 57) is a transcriptional regulator that binds DNA in order to repress or activate the transcription of specific genes. As the mobility of microorganisms in ice is reduced, few proteins related to cell mobility have been identified in this study (spots 96 and 120). However, there is a communication between microorganisms through molecules that act as response regulators^74^. Some of them are required for aerial hyphae formation, or for antibiotic production. A few are regulators of oxidative stress responses or response to chemicals^74^. Another protein of *Streptomyces*, DNA-binding response regulator NarL/FixJ family (spot 68), belongs to a group of transcriptional regulators that are induced by cell envelope stress. They are implicated in the control of various stress responses, cell division, motility, and biofilm formation^75^.

A different protein, the molybdenum cofactor (spot 70), is the active compound at the catalytic site of molybdenum-containing enzymes. This is a key protein as a deficiency in its biosynthesis has lethal consequences for the microorganism^76^. Finally, the hypothetical protein WP_067156483 is very similar (92%) to membrane protein OKH93757 of *Streptomyces uncialis*.

### Metabolism and element cycling in the englacial ecosystem

**Figure 6:**
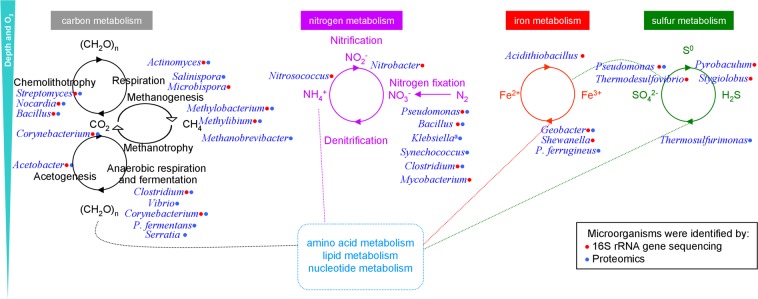
Overview of the metabolic potentials between dominant microorganisms in the englacial ecosystem of Mount Pond glacier. Representative microorganisms identified by 16S rRNA gene sequencing and by Proteomics. ^a^Nitrogen fixation occurs only under anoxic conditions.

As the englacial ecosystem is a habitat with little oxygen, anaerobic microorganisms produce reduced metabolites such as sulphide or methane, which can reduce molecules^14,77^. Electron acceptors include nitrate (reduced to nitrite by enterobacteria or to N_2_ by *Pseudomonas*, ferric iron (reduced to Fe^2+^ by *Geobacter*), sulfate (reduced to hydrogen sulfide by *Desulfovibrio*), carbonate (reduced to methane by methanogens or to acetate by acetogens), and certain organic compounds^78^. There are also aerobic microorganisms that use oxygen dissolved in water or contained in air bubbles. They oxidize compounds such as ferrous iron, manganese (II), ammonium, sulphide, sulphur, methane and hydrogen^46^ (Fig. 6). In Mount Pond Glacier, the contribution of minerals and carbon dioxide comes from inside the volcano. When the volcano erupts, it vents the gas to the atmosphere and covers the land and snow with fresh silicate rock. Calving glaciers, like the west front of Mount Pond Glacier, transport these materials to the ocean^79^. In the dark englacial ecosystem, the small contributions of organic compounds biologically synthesized by chemolithotrophs are relevant (Fig. 6). These compounds are degraded to CH_4_ and CO_2_ (CO_2_ is produced by respiration). Some Proteobacteria, Firmicutes and Actinobacteria^80^, oxidize CO to produce CO_2_, thanks to molybdenum-dependent CO dehydrogenases. For instance, it has been reported that Burkholderiales play a role in the succession of aerobic, molybdenum-dependent CO oxidizing-bacteria on volcanic soils^81,82^. In our cultured-metaproteomic survey we have identified a molybdenum cofactor biosynthesis protein of the Actinobacteria *Streptomyces* (spot 70). Proteins from some CO oxidizers like *Corynebacterium*, *Nocardia* and *Bacillus*^80^ have also been identified in the metaproteomic analysis of cultures. The reduction of CO_2_ by H_2_ to form methane is the major pathway of methanogenesis^78^, although methanogens such as *Methanobrevibacter* are able to produce methane from the fermentation of H_2_ + CO_2_ or from formate, alcohols, or acetate^78^. *Vibrio*, contributes to these processes by a fermentative metabolism^78^. In environments of high pressure and low temperature such as glaciers, CH_4_ derived from microbial activities is trapped as frozen methane hydrates. When ice melts, methane produced in anoxic habitats is insoluble and diffuses to oxic environments, where it is either released to the atmosphere or oxidized to CO_2_ by methanotrophs.

Biological nitrogen fixation is catalyzed by the enzyme nitrogenase which needs a molybdenum cofactor (spot 70, Table S3)^78^. The majority of nitrifying bacteria belonged to Proteobacteria and Nitrospirae, i.e. *Thermodesulfovibrio* (Table S1). Nitrification is the resultant of two consecutive activities: ammonia-oxidation performed by *Nitrosococcus* and *Nitrosovibrio* (Table S1), and oxidation of nitrite to nitrate (carried out by *Nitrobacter*) (Table S1). *Nitrosopumilus* (Table S3) is an autotrophic ammonia-oxidizing chemolithotroph member of the Crenarchaeota. This microorganism oxidizes ammonia to nitrite. The rest known nitrifiers are bacteria (for example, *Nitrobacter* (found by 16S rRNA sequencing) which oxidize nitrite to nitrate.

Important Fe^3+^ reducers include *Shewanella* and *Geobacter*, identified by 16S rRNA sequencing and metaproteomics respectively. Fe^2+^ is oxidized by bacteria such as *Gallionella* and *Leptothrix* (Table S1). In acidic and iron-rich habitats, the acidophilic chemolithotroph *Acidithiobacillus* also oxidizes Fe^2+^ to Fe^3+^. The englacial ecosystem has been defined as an acidic environment^5^, and besides that, pH measured in Mount Pond Glacier samples was around 5.20 (Table 1). In the englacial area where temperatures are low, the acquisition of iron by microorganisms is a difficult process. A mechanism to obtain iron from the environment is the production and secretion of specific siderophores^83^. The TonB complexes, binds siderophores, and facilitate their translocation into the periplasmic space. In *Bacillus*, the hypothetical protein WP_088074644 (spot 59) is very similar to the TonB-dependent receptor, and must be used by microorganisms living inside glaciers.

Volcanic activity constitutes a source of sulfur compounds, from volcanic emissions. Several Epsilonbacteria were identified by 16S rRNA sequencing: *Sulfurospirillum*, *Sulfurimonas denitrificans*, *Arcobacter*. Deltaproteobacteria such as *Desulfovibrio* reduces sulfate to sulfide, with lactate, pyruvate, or H2 as electron donors. But, in these anoxic environments, acetate-oxidizing sulfate-reducing bacteria as *Desulfobacter* or *Desulfosarcina cetonica* sequencing dominate. The Archaea *Pyrobaculum*, found in Mount Pond Glacier by 16S rRNA sequencing, can respire both aerobically and anaerobically with S^0^, Fe^3+^ or NO_3_^−^ as electron acceptors and H_2_ as an electron donor. Other species of *Pyrobaculum* can grow anaerobically on organic electron donors, reducing S^0^ to H_2_S. The Archaea *Stygiolobus*, also found by 16S rRNA sequencing, is also able to reduce S^0^ to H_2_S, but using inorganic electron donors^78^. In metaproteomic studies, some proteins related to sulfur compounds metabolism have been identified. For instance, the arylsulfatase A, which is involved in the metabolism of sulfonates and sulfate esters in Gram-negative bacteria^84^ was identified as spot 27.

### Comparison of the englacial ecosystem with supraglacial and subglacial ecosystems

Comparing current knowledge of microbial diversity from englacial to supraglacial and subglacial ecosystems^7^, there is a less diverse microbial community in the englacial ecosystem. It has been described that there are an interaction and a flow of water and nutrients through water channels of drainage inside glaciers^5,9^. The glacial surface, and especially the cryoconite holes, contain a great diversity of bacteria (mainly cyanobacteria, phytoflagellates) and algae with phototrophic activity^5^, which explains the retention of inorganic N and P^5^. We have also detected photosynthetic microorganisms in the englacial ecosystem, mainly Cyanobacteria and algae (Stramenopiles and Viridiplantae). Yet, according to our results, very few proteins belonged to photosynthetic microorganisms, and they were hypothetical proteins that did not give any useful information to elucidate whether these microorganisms had an active metabolism or whether they were dead cells transported from the upper layers. Most of the cyanobacteria are photoautotrophs, and they only require water, carbon dioxide, and light to live^85^. However, it is known that some cyanobacteria are able to survive in complete darkness with heterotrophic nutrition^86^. Furthermore, cyanobacteria with a hydrogen-based lithoautotrophic metabolism have been recently detected in deep subsurface samples^87^.

In general, the subglacial ecosystem harbors a similar microbial diversity to the englacial diversity we have observed. *In-situ* metabolic activity is poorly known and dominated by a limited range of chemoautotrophic and heterotrophic bacteria^5,88^. However, subglacial microorganisms play a main role in basal rock weathering and geochemical cycling. Recent research has shown that the flux of bioavailable nanoparticulate ions associated with glacial runoff from glaciers is an important source of nutrients to the surrounding oceans^88,89^. In Mount Pond glacier, both the englacial and subglacial ecosystems should be similar regarding their microbial populations; because the ice is covered by mudflow debris, and there is a complete mixture of ice with ash and lava due to volcanic activity followed by erosion. In this glacier, the biggest difference between the englacial and the subglacial environment is temperature, which is much higher in the subglacial ecosystem, since it is an active volcano.

Combination of low temperature and oligotrophy can be found in other Antarctic environments such as the Dry Valleys. We found that bacterial diversity in our samples was dominated by Proteobacteria followed by Actinobacteria and Bacteroidetes, quite similar to other results described for the Dry Valleys^90^. Nevertheless, the englacial ecosystem in Mount Pond glacier shows a significant microbial activity under ambient conditions, which was very low or even undetectable in Dry Valleys^90^.

## Conclusion

Our research demonstrates that (i) an analytical approach that combines a set of -omic techniques: 16S rRNA sequencing plus culturomics and metaproteomics provides key information about the diversity and functions of microbial populations; (ii) these techniques allowed both the identification of microorganisms that take part in biogeochemical cycles inside the englacial ecosystem and the identification of proteins involved in microorganism metabolism; (iii) the detection of marine species such as *Salinispora*, *Halomonas*, *Marinobacter* and *Pseudorhodobacter* suggests that these species could come from sea salt aerosols that are transported from the coast to the front glacier; (iv) culturing cells can increase the abundance and activity of microorganisms, enabling metaproteomic studies to model the structure of the potential microbial community; (v) metaproteomic analyses allowed the detection of OTUs of microorganisms in the englacial ecosystem not found by sequencing methods; (vi) cultured-metaproteomics provides information about the proteins that are actually being used in the community, and their metabolic potentials can be detected in many microorganisms at the same time; (vii) several proteins and enzymes (i.e. ABC transporters, proteins for metabolism, energy production, synthesis of proteins and adaptation to stress) were found that demonstrate the existence of cellular activity at subzero temperatures; (viii) the englacial microorganisms are not quiescent, but they maintain an active metabolism and play an important role in the glacial microbial community; (ix) since mobility of microorganisms in ice is reduced, few proteins related to the cell mobility have been identified in this study; (x) there is a communication between microorganisms through molecules that act as response regulators required for aerial hyphae formation, antibiotic production, oxidative stress regulation, and response to chemicals; (xi) cultured-metaproteomics analyses allowed the detection of new proteins in bacteria that had not been described before (i.e. dodecin domain-containing protein from *Halomonas*). The future research of the role of these proteins, for example in the resistance to environmental organic pollutants, will be challenging.

Similarly to what happens in other rare environments, many Antarctic microorganisms are little known. Future studies of microbial DNA sequences and available public databases will provide a deeper knowledge of the role of microbial communities in glaciers and their contributions to the wider ecosystem.

## Methods

### Sample collection and processing

Deception Island is the summit of a stratovolcano with constant seismic and volcanic activity^91^. The most recent recorded eruptions took place in the years 1842, 1967, 1969 and 1970^21^. The highest peak on Deception Island, South Shetland, Antarctica (Fig. 2A) is Mount Pond, with an altitude of 540 meters above sea level. Mount Pond Glacier (Fig. 2B) runs from its summit to the coast, permanently covered with volcanic ash and pyroclastic material^21^. Samples in this study were collected from the west glacier front to have easier access to the englacial zone (Fig. 2C). GPS coordinates of the sampling points are: S 62° 56′ 1.341″; O 30° 35′ 44.643″. Microbial community samples (designated as MP1, MP2 and MP3) were obtained during the austral summer of 2013. Samples were extracted by drilling with a Mark II Kovacs Core System^92^. One horizontal ice core of 9 cm × 3 m was extracted (Fig. 2D). Only the innermost end of the horizontal core (60 cm) was considered for the analysis (20 cm for each sample approximately). The *in situ* temperature of the samples at the time of collection was around −3 °C. Samples were wrapped in sterile plastic bags and stored at −20 °C until analyzing in the laboratory at the Centre for Astrobiology, Madrid, Spain.

Ice samples were decontaminated following the methods described in previous studies^26^. In a UV-irradiated laminar flow hood, the core surface was trimmed (a 1 cm thickness) to remove the exterior ice. Then, the ice core was soaked in ice-cold 95% ethanol, followed by rinsing with sterile MilliQ water. Each of the 3 ice samples was thawed in sterile containers at 4 °C (1000 mL), and separately used for all the analysis (geochemical analysis, cell cultures and 16S rRNA sequencing). All procedures were performed using ethanol-sterilized tools and sterilized gloves. To control for laboratory contamination, 1 liter of MilliQ rinse water was subjected to identical analytical procedures.

### Chemical analysis of meltwater

Assays for NH_4_^+^, NO_2_^−^, NO_3_^−^, total dissolved nitrogen (TDN) and soluble reactive phosphorus (SRP)) from each sample were performed as described elsewhere^26^ by ion chromatography in an 861 Advance Compact IC system (Metrohm AG, Herisau, Switzerland). Ions were identified and quantified with internal and external standards prepared from Certified Standard Solutions (TraceCERT^®^) (Merck). Chromatograms were done with the Metrohm IC Net 2.3 SR4 software.

Concentrations of Fe, Mg, S, Ca, Cl, Mn, K, Na, Al, As, B, Ba, Be, Bi, Co, Cu, Li, Mo, Ni, P, Pb, Rb, Sc, Se, Sr, V, Y, Zn, Zr, Cd, Cr, F, Ge and Ti were found out by inductively coupled plasma-mass spectrometry (ICP-MS) on a PerkinElmer ELAN9000 ICP-MS quadrupole spectrometer (Table 1). The method was previously described^93^. Quantitative ICP-MS analysis was performed by an external standard calibration methodology.

The run was performed as follow: a blank (HNO_3_ 1% (Suprapur^®^, Merck), external standards with different concentrations, samples and a quality control standard at the end to control the instrumental drift and possible memory effects. All the solutions used (standards and samples) were acidified with nitric acid HNO_3_ 1% (Suprapur^®^, Merck). ^103^Rh was added to the blank, standard, and sample solutions as an internal standard. External calibration and quality control standards were prepared from two initial multi-element solutions of 1000 mg L^−1^: Multi-element Calibration Standard 2 (Perkin-Elmer) and ICP multi-element standard solution VI Certipur^®^ (Merck) containing all the analyzed elements.

Of all the chemical elements that were analyzed, only those represented in Table 1 (Fe, Mg, S, Ca, Cl, Mn, K and Na) presented concentrations high (≥3 ppb). The rest were scarce (<3 ppb) (Al, As, B, Ba, Be, Bi, Co, Cu, Li, Mo, Ni, P, Pb, Rb, Sc, Se, Sr, V, Y, Zn and Zr) or undetectable (F, Ge and Ti). Detection limits for constituents that were below detection limits ranged from 0.3 to 0.7 ppb.

### Cultured-metaproteomics

Recently, microbial culturomics (a culturing process that uses multiple culture conditions) followed by metaproteomics or by 16S rRNA sequencing has been widely used to elucidate the understanding of microbial communities^94–96^. In this study, a similar strategy was carried out with the aim of deepening the knowledge about the englacial microbial community. Culture conditions are summarized in Table S4 and Table 2. Broth cultures (100 mL) were grown in the dark with agitation in Erlenmeyer flasks. Growth was monitored by optical density at 600 nm until the achievement of an absorbance of 0.3. Nutrient formulations for T2, T3, T4, T6, T7, T8 and T9 were prepared as concentrated stocks (20×), sterilized and added to 100 mL of meltwater (from each of the 3 direct samples) to get 1x final concentration. Blanks were made with 100 mL of sterile water supplemented with the same nutrients. Each sample was incubated at 5 temperatures (−4 °C, 0 °C, 4 °C, 20 °C and 28 °C)^97^. A total of 120 cultures were obtained (Table S1). The cultures that first reached an absorbance of 0.3 were represented in the growth plots in Fig. 3C (except T1, which were not cultivated). All growth rates are represented in Fig. S2. These cultures, in addition to three other uncultivated meltwater samples (T1), were analyzed by metaproteomics. The cells were centrifuged (10,000 × g, 15 min), rinsed in PBS and stored at −20 °C. Then, proteins were extracted as explained in previous reports^98,99^. A total of 120 cultures were obtained (3 glacial ice sample replicates in 8 culture media at 5 different temperatures).

### Extraction, quantification and sequencing of the 16S rRNA gene

DNA from samples MP1, MP2 and MP3 were extracted from meltwater (500 mL each). Amplification and sequencing of the V3 and V4 regions of the 16S rRNA gene (Table S5) were performed as previously reported^89^. Sequences were analyzed with UCHIME^100^, to identify and remove chimeric reads, and classified to eliminate those that could be considered contaminants. Sequence reads were grouped for their taxonomic classification using the BaseSpace platform and the Greengenes 16S rRNA database. In general, this strategy allowed the classification of sequences into taxonomic categories that were never lower than genus. The correspondence of OTUs to species was assessed considering a 97% identity threshold. Analytic Rarefaction 1.3 software (https://strata.uga.edu/software) was used to perform rarefaction analysis. It demonstrated that, at 3% sequence divergence, rarefaction curves nearly reached saturation, indicating that samples contained almost all the diversity at this genetic distance (Fig. S4).

### Metaproteomics

Although DNA sequencing of the 16S rRNA gene and the housekeeping genes (i. e. *dnaJ*, *hsp60*, *sodA*, *rpoA*, *rpoB*, *gyrA*, *gyrB*, etc.) have traditionally been used to identify OTUs^101^, it has been reported that the conserved genes can be transferred through natural horizontal gene transfer^102^ and their sequence also tend to vary by mutation. A polyphasic approach for classification and identification of bacterial species includes other methods available such as MALDI-TOF MS.

In this study, the cultured cells were centrifuged and lysed following the method previously described^103^. A total of 108 gels were prepared: 4 replicates of 24 cultures +3 direct samples (Fig. 3D). Protein spots from the Coomassie-stained 2-DE gels were digested and analyzed by MALDI-TOF MS with the method previously described^83,104^. The MALDI-TOF mass spectrometer was calibrated using a peptide calibration standard mixture (reference No. 222570, Bruker Daltonics) containing nine standard peptides in a mass range between ~700 and 3500 Da. Spectral data were analyzed to search the NCBIprot database using the Mascot database search algorithm. Combined peptide mass fingerprint and MS/MS ion search modes were used.

Given that we are searching for environmental microorganisms, some precautions have been taken to check the results from protein identifications. Only peptides and proteins with confidence scores of at least 66% (p < 0.05) were considered positive identifications. We have also considered other main features of proteins in 2-DE gels such as their molecular weight and isoelectric point to better confirm their identity^105^. Mascot search parameters were tryptic peptides with one missed cleavage, using 80 ppm and 0.3 Da for precursor and fragment tolerances, respectively. Theoretical molecular weight and isoelectric point were calculated for each protein with the Compute pI/MW tool (ExPASy, Swiss Institute of Bioinformatics, Switzerland). Apparent molecular weight and isoelectric point were obtained from each spot position in gels.

### Nucleotide and protein sequence accession numbers

Sequences obtained by 16S rRNA sequencing have been deposited in NCBI Short Read Archive (SRA) (accession number PRJNA430179).

The mass spectrometry proteomics data have been deposited to the ProteomeXchange Consortium via the PRIDE^106^ repository with the dataset identifiers PXD010060 and PXD010185.

## Supplementary information

Supplementary Information

## References

[1] Margesin R, Miteva V (2011). Diversity and ecology of psychrophilic microorganisms. Res Microbiol.

[2] Anesio AM, Lutz S, Chrismas NAM, Benning LG (2017). The microbiome of glaciers and ice sheets. NPJ Biofilms Microbiomes..

[3] Hotaling S, Hood E, Hamilton TL (2017). Microbial ecology of mountain glacier ecosystems: biodiversity, ecological connections and implications of a warming climate. Environ. Microbiol.

[4] Garcia-Lopez, E., Alcazar, P., Postigo, M. & Cid, C. The Effect of Climate Change on Microbial Communities from Glaciers In *Glaciers: Formation*, *Climate Change and Their Effects* (Nova Science Publishers) 71–88 (New York, 2016).

[5] Hodson A (2008). Glacial ecosystems. Ecological Monographs.

[6] Lanoil B (2009). Bacteria beneath the West Antarctic ice sheet. Environ Microbiol.

[7] Boetius A, Anesio AM, Deming JW, Mikucki JA, Rapp JZ (2015). Microbial ecology of the cryosphere: sea ice and glacial habitats. Nat. Rev. Microbiol.

[8] Edwards A (2011). Possible interactions between bacterial diversity, microbial activity and supraglacial hydrology of cryoconite holes in Svalbard. ISME J.

[9] Garcia-Lopez E, Cid C (2017). Glaciers and ice sheets as analog environments of potentially habitable icy worlds. Front. Microbiol.

[10] Garcia-Lopez, E. & Cid, C. The role of microbial ecology in glacier retreat analysis In *Glaciers* (ed. Tangborn, W. V.) (InTech, Rijeka, 2017).

[11] Tranter M (2004). Extreme hydrochemical conditions in natural microcosms entombed within Antarctic ice. Hydrological Processes.

[12] Aerts JW, Röling WF, Elsaesser A, Ehrenfreund P (2014). Biota and biomolecules in extreme environments on Earth: implications for life detection on Mars. Life.

[13] Maccario L, Sanguino L, Vogel TM, Larose C (2015). Snow and ice ecosystems: not so extreme. Res Microbiol.

[14] Skidmore M, Foght J, Sharp MJ (2000). Microbial life beneath a High Arctic glacier. Appl Environ Microbiol.

[15] Phillips SJM, Parnell J (2006). The detection of organic matter in terrestrial snow and ice: implications for astrobiology. Int. J. Astrobiol.

[16] Harrold ZR (2016). Aerobic and anaerobic thiosulfate oxidation by a cold-adapted, subglacial chemoautotroph. Appl. Environ. Microbiol.

[17] Mikucki JA (2009). A contemporary microbially maintained subglacial ferrous “ocean”. Science.

[18] Lerner, L. & Wilmoth, B. Chemoautotrophic and chemolithotrophic bacteria In *World of Microbiology and Immunology*. (Lerner, K. eds), http://www.encyclopedia.com (2016).

[19] Tung HC, Bramall NE, Price PB (2005). Microbial origin of excess methane in glacial ice and implications for life on Mars. Proc Natl Acad Sci USA.

[20] Amato P (2007). Bacterial characterization of the snow cover at Spitzberg, Svalbard. FEMS Microbiol Ecol.

[21] Baker, P. E., Mcreath, I., Harvey, M. R., Roobol, M. & Davies, T.G. The geology of the South Shetland Islands. In *Volcanic evolution of Deception Island*. British Antarctic Survey Scientific Reports, N° 78, 81 (1975).

[22] Smith, K. L., Baldwin, R. J., Kaufmann, R. S. & Sturz, A. Ecosystem studies at Deception Island, Antarctica: an overview In *Deep Sea Research Part II: Topical Studies in Oceanography* 50 (Elsevier Ltd.) 1595–1609 (2003).

[23] Deming JW (2002). Psychrophiles and Polar Regions. Curr Opin Microbiol.

[24] Christner BC, Mosley-Thompson E, Thompson LG, Reeve JN (2003). Bacterial recovery from ancient glacial ice. Environ Microbiol.

[25] Christner BC, Kvitko BH, Reeve JN (2003). Molecular identification of bacteria and eukarya inhabiting an Antarctic cryoconite hole. Extremophiles.

[26] Garcia-Descalzo L (2013). Eukariotic microorganisms in cold enviroments: examples from Pyrenean glaciers. Front Microbiol.

[27] Doyle, S. M., Dieser, M., Broemsen, E. & Christner, B. C. General characteristics of cold-adapted microorganisms In *Polar Microbiology: Life in a* Deep Freeze (eds Whyte, L. & Miller, R. V.) 103–125 (Washington, DC: ASM Press, 2012).

[28] Ishii S (2015). Microbial metabolic networks in a complex electrogenic biofilm recovered from a stimulus-induced metatranscriptomics approach. Sci Rep.

[29] Hug L (2016). A new view of the tree of life. Nature Microbiology.

[30] Pulschen AA (2017). Isolation of uncultured bacteria from Antarctica using long incubation periods and low nutritional media. Front Microbiol.

[31] Benndorf D, Balcke GU, Harms H, von Bergen M (2007). Functional metaproteome analysis of protein extracts from contaminated soil and groundwater. ISME J.

[32] Brinkmeyer R, Glöckner FO, Helmke E, Amann R (2004). Predominance of ß-proteobacteria in summer melt pools on Arctic pack ice. Limnol. Oceanogr.

[33] Gaidos E (2004). A viable microbial community in a subglacial volcanic crater lake, Iceland. Astrobiology.

[34] Han D (2014). Bacterial Communities of Surface Mixed Layer in the Pacific Sector of the Western Arctic Ocean during Sea-Ice Melting. PLoS ONE.

[35] Lyons W (2005). Groundwater seeps in Taylor Valley Antarctica: An example of a subsurface melt event. Annals of Glaciology.

[36] Mergaert J, Cnockaert MC, Swings J (2003). *Thermomonas fusca* sp. nov. and *Thermomonas brevis* sp. nov., two mesophilic species isolated from a denitrification reactor with poly(epsilon-caprolactone) plastic granules as fixed bed, and emended description of the genus Thermomonas. Int J Syst Evol Microbiol.

[37] Jamroze A (2014). The reverse gyrase from *Pyrobaculum calidifontis*, a novel extremely thermophilic DNA topoisomerase endowed with DNA unwinding and annealing activities. J Biol Chem.

[38] Di Lorenzo F (2014). Thermophiles as potential source of novel endotoxin antagonists: the full structure and bioactivity of the lipo-oligosaccharide from *Thermomonas hydrothermalis*. Chembiochem.

[39] Sant’Anna FH, Lebedinsky AV, Sokolova TG, Robb FT, Gonzalez JM (2015). Analysis of three genomes within the thermophilic bacterial species *Caldanaerobacter* subterraneus with a focus on carbon monoxide dehydrogenase evolution and hydrolase diversity. BMC Genomics.

[40] Mardanov AV, Beletsky AV, Kadnikov VV, Slobodkin AI, Ravin NV (2016). Genome Analysis of *Thermosulfurimonas dismutans*, the First Thermophilic Sulfur-Disproportionating Bacterium of the Phylum Thermodesulfobacteria. Front Microbiol.

[41] Lutz S, Anesio AM, Edwards A, Benning LG (2015). Microbial diversity on Icelandic glaciers and ice caps. Front Microbiol.

[42] Whisstock JC, Lesk AM (2003). Prediction of protein function from protein sequence and structure. Q Rev Biophys.

[43] Sivashankari S, Shanmughavel P (2006). Functional annotation of hypothetical proteins - A review. Bioinformation.

[44] Martin-Cerezo ML, Garcia-Lopez E, Cid C (2015). Isolation and identification of a red pigment from the Antarctic bacterium *Shewanella frigidimarina*. Protein Pept Lett.

[45] Yu J, Ge J, Heuveling J, Schneider E, Yang M (2015). Structural basis for substrate specificity of an amino acid ABC transporter. Proc Natl Acad Sci USA.

[46] Hallbeck, L. Microbial processes in glaciers and permafrost. A literature study on microbiology affecting groundwater at ice sheet melting. (Kärnbränslehantering, Svensk, 2009).

[47] Lien T, Madsen M, Rainey FA, Birkeland NK (1998). *Petrotoga mobilis* sp. nov., from a North Sea oil-production well. Int J Syst Bacteriol.

[48] Carpenter EJ, Lin S, Capone DG (2000). Bacterial activity in South Pole snow. Appl Environ Microbiol.

[49] Dieser M, Battista JR, Christner BC (2013). DNA double-strand break repair at −15 °C. Appl Environ. Microbiol.

[50] Junge K, Eicken H, Swanson BD, Deming JW (2006). Bacterial incorporation of leucine into protein down to −20 °C with evidence for potential activity in sub-eutectic saline ice formations. Cryobiology.

[51] Murray AE (2012). Microbial life at −13 °C in the brine of an ice-sealed Antarctic lake. Proc Natl Acad Sci USA.

[52] Lauro FM (2011). An integrative study of a meromictic lake ecosystem in Antarctica. ISME J.

[53] Babady NE, Pang YP, Elpeleg O, Isaya G (2007). Cryptic proteolytic activity of dihydrolipoamide dehydrogenase. Proc Natl Acad Sci USA.

[54] Kim SK, Makino K, Amemura M, Shinagawa H, Nakata A (1993). Molecular analysis of the phoH gene, belonging to the phosphate regulon in. Escherichia coli. J Bacteriol.

[55] Ma JF (1999). Bacterioferritin A modulates catalase A (KatA) activity and resistance to hydrogen peroxide in *Pseudomonas aeruginosa*. J Bacteriol.

[56] Gerdes K, Howard M, Szardenings F (2010). Pushing and pulling in prokaryotic DNA segregation. Cell.

[57] Grininger M, Staudt H, Johansson P, Wachtveitl J, Oesterhelt D (2009). Dodecin is the key player in flavin homeostasis of archaea. J Biol Chem.

[58] Garcia-Lopez, E. & Cid, C. Color producing extremophiles In *Bio-Pigmentation and* Biotechnological *Implementations* (ed. Singh, O. V.) (Wiley-Blackwell, Hoboken, NJ, 2016).

[59] Nissen MS (2008). Crystal structures of NADH: FMN oxidoreductase (EmoB) at different stages of catalysis. J Biol Chem.

[60] Blombach F (2012). An HflX-type GTPase from *Sulfolobus solfataricus* binds to the 50S ribosomal subunit in all nucleotide-bound states. J Bacteriol.

[61] Lin S, Hanson RE, Cronan JE (2010). Biotin synthesis begins by hijacking the fatty acid synthetic pathway. Nat Chem Biol.

[62] Carrondo MA (2003). Ferritins, iron uptake and storage from the bacterioferritin viewpoint. EMBO J.

[63] Chen XH (2006). Structural and functional characterization of three polyketide synthase gene clusters in *Bacillus amyloliquefaciens* FZB 42. J Bacteriol.

[64] Chen XH (2007). Comparative analysis of the complete genome sequence of the plant growth-promoting bacterium *Bacillus amyloliquefaciens* FZB42. Nat Biotechnol.

[65] Lee C, Kim MI, Hong M (2017). Structural and functional analysis of BF2549, a PadR-like transcription factor from *Bacteroides fragilis*. Biochem Biophys Res Commun.

[66] Snider J, Houry WA (2006). MoxR AAA+ ATPases: a novel family of molecular chaperones?. J Struct Biol.

[67] Holberger LE, Garza-Sánchez F, Lamoureux J, Low DA, Hayes CS (2012). A novel family of toxin/antitoxin proteins in *Bacillus* species. FEBS Lett.

[68] Benz J, Meinhart A (2014). Antibacterial effector/immunity systems: it’s just the tip of the iceberg. Curr Opin Microbiol.

[69] Yu XC, Tran AH, Sun Q, Margolin W (1998). Localization of cell division protein FtsK to the *Escherichia coli* septum and identification of a potential N-terminal targeting domain. J Bacteriol.

[70] Foster SJ (1991). Cloning, expression, sequence analysis and biochemical characterization of an autolytic amidase of *Bacillus subtilis* 168 trpC2. J Gen Microbiol.

[71] Boissier F (2006). Further insight into S-adenosylmethionine-dependent methyltransferases: structural characterization of Hma, an enzyme essential for the biosynthesis of oxygenated mycolic acids in *Mycobacterium tuberculosis*. J Biol Chem.

[72] Young DB, Comas I, de Carvalho LP (2015). Phylogenetic analysis of vitamin B12-related metabolism in *Mycobacterium tuberculosis*. Front Mol Biosci.

[73] Milón P, Maracci C, Filonava L, Gualerzi CO, Rodnina MV (2012). Real-time assembly landscape of bacterial 30S translation initiation complex. Nat Struct Mol Biol.

[74] Romero-Rodríguez A, Robledo-Casados I, Sánchez S (2015). An overview on transcriptional regulators in *Streptomyces*. Biochim Biophys Acta.

[75] Pannen D, Fabisch M, Gausling L, Schnetz K (2016). Interaction of the RcsB response regulator with auxiliary transcription regulators in *Escherichia coli*. J Biol Chem.

[76] Mendel RR (2013). The molybdenum cofactor. J Biol Chem.

[77] Foght J (2004). Culturable bacteria in subglacial sediments and ice from two southern hemisphere glaciers. Microb Ecol.

[78] Madigan, M. T., Martinko, J. M., Stahl, D. A. & Clark, D. P. Brock biology of microorganisms. (13th ed. Pearson Education, Inc. San Francisco, CA., 2012).

[79] Zeebe RE, Caldeira K (2008). Close mass balance of long-term carbon fluxes from ice-core CO_2_ and ocean chemistry records. Nat Geosci.

[80] Park SW (2003). Growth of mycobacteria on carbon monoxide and methanol. J Bacteriol..

[81] Weber CF, King GM (2017). Volcanic soils as sources of novel CO-oxidizing *Paraburkholderia* and *Burkholderia*: *Paraburkholderia hiiakae* sp. nov., *Paraburkholderia metrosideri* sp. nov., *Paraburkholderia paradisi* sp. nov., *Paraburkholderia peleae* sp. nov., and *Burkholderia alpina* sp. nov. a member of the *Burkholderia cepacia* complex. Front Microbiol..

[82] King GM (2006). Nitrate-dependent anaerobic carbon monoxide oxidation by aerobic CO-oxidizing bacteria. FEMS Microbiol Ecol..

[83] Garcia-Descalzo L, Garcia-Lopez E, Alcazar A, Baquero F, Cid C (2014). Proteomic analysis of the adaptation to warming in the Antarctic bacteria *Shewanella frigidimarina*. Biochim Biophys Acta.

[84] Kertesz MA (2000). Riding the sulfur cycle-metabolism of sulfonates and sulfate esters in gram-negative bacteria. FEMS Microbiol Rev.

[85] Mur, L. R., Skulberg, O. M. & Utkilen, H. Cyanobacteria in the environment In *Toxic Cyanobacteria in Water: A guide to their public health consequences*, *monitoring and management* (eds Chorus, I. & Bartram, J.) (1999).

[86] Fay P (1965). Heterotrophy and Nitrogen Fixation in *Chlorogloea fritschii*. J Mobiol..

[87] Puente-Sánchez F (2018). Viable cyanobacteria in the deep continental subsurface. Proc Natl Acad Sci USA.

[88] Nixon SL, Telling JP, Wadham JL, Cockell CS (2017). Viable cold-tolerant iron-reducing microorganisms in geographically diverse subglacial environments. Biogeosciences.

[89] Garcia-Lopez, E., Rodriguez-Lorente, I., Alcazar, P. & Cid, C. Microbial communities in coastal glaciers and tidewater tongues of Svalbard Archipelago, Norway. *Front Mar Sci* **5** (2019).

[90] Goordial J (2016). Nearing the cold-arid limits of microbial life in permafrost of an upper dry valley, Antarctica. ISME J.

[91] Logan NA (2000). Aerobic endospore-forming bacteria from geothermal environments in northern Victoria Land, Antarctica, and Candlemas Island, South Sandwich archipelago, with the proposal of *Bacillus fumarioli* sp. nov. Int J Syst Evol Microbiol.

[92] Green J, Koci B, Kyne J (2007). Koci drill for drilling in ice, sand and rock: drill requirements, design, performance, difficulties. Annals of Glaciology.

[93] Nna-Mvondo D, Martin-Redondo MP, Martinez-Frias J (2008). New application of microwave digestion-inductively coupled plasma-mass spectrometry for multi-element analysis in komatiites. Anal Chim Acta.

[94] Lagier JC (2012). Microbial culturomics: paradigm shift in the human gut microbiome study. Clin Microbiol Infect.

[95] Mourembou G (2015). Rise of microbial culturomics: noncontiguous finished genome sequence and description of *Beduini massiliensis* gen. nov., sp. nov. OMICS.

[96] Lagier JC (2016). Culture of previously uncultured members of the human gut microbiota by culturomics. Nat Microbiol.

[97] Williams TJ (2011). Defining the response of a microorganism to temperatures that span its complete growth temperature range (−2 °C to 28 °C) using multiplex quantitative proteomics. Environ Microbiol.

[98] Garcia-Descalzo L, Alcazar A, Baquero F, Cid C (2011). Identification of *in vivo* HSP90-interacting proteins reveals modularity of HSP90 complexes is dependent on the environment in psychrophilic bacteria. Cell Stress Chaperones.

[99] Garcia-Descalzo L (2012). Mass spectrometry for direct identification of biosignatures and microorganisms in Earth analogs of Mars. Planet Space Sci.

[100] Edgar RC, Haas BJ, Clemente JC, Quince C, Knight R (2011). UCHIME improves sensitivity and speed of chimera detection. Bioinformatics.

[101] Das S, Dash HR, Mangwani N, Chakraborty J, Kumari S (2014). Understanding molecular identification and polyphasic taxonomic approaches for genetic relatedness and phylogenetic relationships of microorganisms. J Microbiol Methods.

[102] Ochman H, Lawrence JG, Groisman EA (2000). Lateral gene transfer and the nature of bacterial innovation. Nature.

[103] Cid C, Garcia-Descalzo L, Casado-Lafuente V, Amils R, Aguilera A (2010). Proteomic analysis of the response of an acidophilic strain of *Chlamydomonas* sp. (Chlorophyta) to natural metal-rich water. Proteomics.

[104] Marin-Yaseli, M. R., Cid, C., Yagüe, A. I. & Ruiz-Bermejo, M. Detection of macromolecular fractions in HCN polymers using electrophoretic and ultrafiltration techniques. *Chem Biodivers* **14** (2017). 10.1002/cbdv.201600241 27518115

[105] Baggerman G, Vierstraete E, De Loof A, Schoofs L (2005). Gel-based versus gel-free proteomics: a review. Comb Chem High Throughput Screen.

[106] Vizcaíno JA (2016). update of the PRIDE database and related tools. Nucleic Acids Res.

[107] Bidle KD, Lee S, Marchant DR, Falkowski PG (2007). Fossil genes and microbes in the oldest ice on Earth. PNAS.

