# Supplementary material for: Taxonomic and functional characterization of a microbial community from a volcanic englacial ecosystem in Deception Island, Antarctica
Source: Sci Rep. 2019 Aug 21;9:12158. doi: 10.1038/s41598-019-47994-9 (PMC6704131; doi:10.1038/s41598-019-47994-9)
Supplement: Supplementary file 1 — Supplementary Information [file 41598_2019_47994_MOESM1_ESM.pdf]

<u>SUPPLEMENTARY INFORMATION FOR:</u>

Taxonomic and functional characterization of a microbial community from a volcanic englacial ecosystem in Deception Island, Antarctica

Emma Martínez-Alonso, Sonia Peña, Sandra Serrano, Eva García-Lopez, Alberto Alcazar and Cristina Cid[*]

[*] Corresponding Author: cidsc@inta.es

This file includes:

I. Supplementary Tables S1-S5

II. Supplementary Figures. Figures S1-S4

III. Mascot search results

Table S1. Analysis of bacterial 16S rRNA clones retrieved from ice samples by 16S rRNA sequencing. OTU level aggregate counts of three sampling replicates.

**Phylum**	**Class**	**Order**	**Family**	**Genus**	**Species**	**num_hits**	**%_hits**
						665	0.185
Bacteroidetes	Sphingobacteriia	Sphingobacteriales	Flexibacteraceae	*Hymenobacter*		36213	10.059
Cyanobacteria	Oscillatoriophycideae	Oscillatoriales	Phormidiaceae	*Symploca*	*atlantica*	23398	6.500
Cyanobacteria						20191	5.609
Actinobacteria	Actinobacteria	Actinomycetales				18764	5.212
Bacteroidetes	Sphingobacteriia	Sphingobacteriales	Flexibacteraceae	*Pontibacter*	*niistensis*	14745	4.096
Actinobacteria	Actinobacteria	Actinomycetales	Frankiaceae	*Frankia*	*alni*	12409	3.447
Cyanobacteria	Oscillatoriophycideae	Chroococcales	Cyanobacteriaceae	*Cyanobacterium*	*aponinum*	12111	3.364
						11945	3.318
Cyanobacteria	Oscillatoriophycideae					9601	2.667
Cyanobacteria	Oscillatoriophycideae	Chroococcales				7922	2.201
Bacteroidetes	Sphingobacteriia	Sphingobacteriales	Chitinophagaceae	*Segetibacter*	*aerophilus*	7903	2.195
Actinobacteria	Actinobacteria	Actinomycetales	Microbacteriaceae	*Salinibacterium*	*xinjiangense*	7675	2.132
Proteobacteria	Betaproteobacteria	Burkholderiales	Comamonadaceae	*Polaromonas*		6655	1.849
Bacteroidetes	Sphingobacteriia	Sphingobacteriales	Flexibacteraceae	*Hymenobacter*	*soli*	6476	1.799
Bacteroidetes	Sphingobacteriia	Sphingobacteriales	Flexibacteraceae			5305	1.474
Actinobacteria	Actinobacteria	Actinomycetales	Pseudonocardiaceae	*Pseudonocardia*	*thermophila*	4955	1.376
Cyanobacteria	Nostocophycideae	Stigonematales	Rivulariaceae	*Calothrix*		4798	1.333
Actinobacteria	Actinobacteria	Actinomycetales	Nocardiaceae	*Rhodococcus*		4568	1.269
Actinobacteria	Actinobacteria	Actinomycetales	Kineosporiaceae	*Kineosporia*	*mikuniensis*	4553	1.265
Proteobacteria	Betaproteobacteria	Burkholderiales	Comamonadaceae	*Methylibium*		4235	1.176
Cyanobacteria	Nostocophycideae	Nostocales	Nostocaceae			4192	1.164
Cyanobacteria	Nostocophycideae	Stigonematales	Rivulariaceae	*Calothrix*	*parietina*	4129	1.147
Proteobacteria	Betaproteobacteria	Burkholderiales	Oxalobacteraceae	*Janthinobacterium*		4100	1.139
Proteobacteria	Betaproteobacteria	Burkholderiales	Comamonadaceae	*Paucibacter*		4056	1.127
Bacteroidetes	Sphingobacteriia	Sphingobacteriales	Chitinophagaceae			3843	1.068
Proteobacteria	Betaproteobacteria	Burkholderiales	Oxalobacteraceae	*Janthinobacterium*	*agaricidamnosum*	3505	0.974
Proteobacteria	Betaproteobacteria	Burkholderiales	Comamonadaceae	*Variovorax*	*boronicumulans*	3314	0.921
Actinobacteria	Actinobacteria	Actinomycetales	Streptomycetaceae	*Streptomyces*		3188	0.886
Proteobacteria	Alphaproteobacteria	Rhodospirillales	Acetobacteraceae	*Acidisoma*	*tundrae*	2857	0.794
Proteobacteria	Betaproteobacteria	Burkholderiales	Comamonadaceae			2741	0.761
Actinobacteria	Actinobacteria	Actinomycetales	Pseudonocardiaceae	*Pseudonocardia*		2593	0.720
Cyanobacteria	Oscillatoriophycideae	Chroococcales	Phormidiaceae	*Hydrocoleum*		2576	0.716

Actinobacteria	Actinobacteria	Actinomycetales	Nocardiaceae	*Rhodococcus*	*phenolicus*	2562	0.712
Bacteroidetes	Sphingobacteriia	Sphingobacteriales	Flexibacteraceae	*Hymenobacter*	*daecheongensis*	2544	0.707
Actinobacteria	Actinobacteria	Actinomycetales	Pseudonocardiaceae	*Pseudonocardia*	*hydrocarbonoxydans*	2322	0.645
Firmicutes	Clostridia	Clostridiales				2056	0.571
Bacteroidetes	Sphingobacteriia	Sphingobacteriales	Flexibacteraceae	*Flectobacillus*		2054	0.571
Bacteroidetes	Sphingobacteriia	Sphingobacteriales	Flexibacteraceae	*Hymenobacter*	*roseosalivarius*	2042	0.567
Actinobacteria	Actinobacteria	Actinomycetales	Microbacteriaceae	*Salinibacterium*	*amurskyense*	2016	0.560
Proteobacteria						1887	0.524
Thermi	Deinococci	Deinococcales	Deinococcaceae	*Deinococcus*	*claudionis*	1866	0.518
Actinobacteria	Actinobacteria	Actinomycetales	Nocardiaceae	*Rhodococcus*	*percolatus*	1812	0.503
Cyanobacteria	Synechococcophycideae	Pseudanabaenales	Pseudanabaenaceae	*Arthronema*	*africanum*	1791	0.498
Actinobacteria	Actinobacteria	Actinomycetales	Microbacteriaceae			1696	0.471
Proteobacteria	Betaproteobacteria	Burkholderiales	Comamonadaceae	*Polaromonas*	*naphthalenivorans*	1602	0.445
Proteobacteria	Alphaproteobacteria	Sphingomonadales	Sphingomonadaceae	*Kaistobacter*	*terrae*	1595	0.443
Actinobacteria	Actinobacteria	Actinomycetales	Microbacteriaceae	*Salinibacterium*		1499	0.416
Actinobacteria	Actinobacteria	Actinomycetales	Intrasporangiaceae	*Knoellia*	*aerolata*	1448	0.402
Firmicutes						1275	0.354
Actinobacteria	Actinobacteria	Actinomycetales	Actinosynnemataceae			1246	0.346
Actinobacteria	Actinobacteria	Actinomycetales	Actinosynnemataceae	*Kutzneria*		1154	0.321
Actinobacteria	Actinobacteria	Actinomycetales	Pseudonocardiaceae	*Amycolatopsis*		1088	0.302
Actinobacteria	Actinobacteria	Actinomycetales	Pseudonocardiaceae	*Pseudonocardia*	*babensis*	1044	0.290
Actinobacteria	Actinobacteria	Actinomycetales	Nocardiaceae	*Rhodococcus*	*equi*	902	0.251
Thermi	Deinococci	Deinococcales	Deinococcaceae	*Deinococcus*		888	0.247
Proteobacteria	Betaproteobacteria					881	0.245
Bacteroidetes	Sphingobacteriia	Sphingobacteriales	Flexibacteraceae	*Hymenobacter*	*xinjiangensis*	823	0.229
Proteobacteria	Betaproteobacteria	Burkholderiales	Comamonadaceae	*Rhodoferax*	*ferrireducens*	792	0.220
Proteobacteria	Alphaproteobacteria	Rhodospirillales	Acetobacteraceae			785	0.218
Cyanobacteria	Nostocophycideae	Nostocales	Scytonemataceae	*Scytonema*		748	0.208
Cyanobacteria	Oscillatoriophycideae	Chroococcales	Phormidiaceae			732	0.203
Proteobacteria	Alphaproteobacteria	Rhodospirillales	Acetobacteraceae	*Acidiphilium*	*angustum*	720	0.200
Cyanobacteria	Nostocophycideae					689	0.191
Proteobacteria	Alphaproteobacteria	Sphingomonadales	Sphingomonadaceae	*Kaistobacter*		681	0.189
Proteobacteria	Betaproteobacteria	Burkholderiales	Comamonadaceae	*Variovorax*	*paradoxus*	657	0.183
Proteobacteria	Betaproteobacteria	Burkholderiales	Comamonadaceae	*Delftia*	*lacustris*	638	0.177
Bacteroidetes						594	0.165
Actinobacteria	Actinobacteria	Actinomycetales	Intrasporangiaceae	*Janibacter*	*anophelis*	580	0.161
Proteobacteria	Alphaproteobacteria	Sphingomonadales	Sphingomonadaceae	*Sphingomonas*		574	0.159

Bacteroidetes	Sphingobacteriia	Sphingobacteriales	Chitinophagaceae	*Chitinophaga*	*soli*	571	0.159
Cyanobacteria	Nostocophycideae	Stigonematales	Rivulariaceae	*Calothrix*	*brevissima*	545	0.151
Actinobacteria	Actinobacteria	Actinomycetales	Actinosynnemataceae	*Crossiella*		513	0.143
Bacteroidetes	Flavobacteriia	Flavobacteriales	Flavobacteriaceae	*Polaribacter*	*butkevichii*	501	0.139
Actinobacteria	Actinobacteria	Actinomycetales	Intrasporangiaceae			489	0.136
Actinobacteria	Actinobacteria	Actinomycetales	Thermomonosporaceae	*Actinoallomurus*	*luridus*	477	0.133
Proteobacteria	Alphaproteobacteria	Sphingomonadales	Sphingomonadaceae	*Zymomonas*		476	0.132
Actinobacteria	Actinobacteria	Actinomycetales	Intrasporangiaceae	*Janibacter*		472	0.131
Proteobacteria	Betaproteobacteria	Burkholderiales	Oxalobacteraceae	*Oxalobacter*	*vibrioformis*	461	0.128
Proteobacteria	Betaproteobacteria	Burkholderiales				454	0.126
Actinobacteria	Actinobacteria	Actinomycetales	Intrasporangiaceae	*Phycicoccus*	*dokdonensis*	445	0.124
Proteobacteria	Alphaproteobacteria	Rhodospirillales	Acetobacteraceae	*Acidiphilium*	*symbioticum*	425	0.118
Cyanobacteria	Nostocophycideae	Nostocales				416	0.116
Actinobacteria						399	0.111
Actinobacteria	Actinobacteria	Actinomycetales	Mycobacteriaceae	*Mycobacterium*	*pinnipedii*	395	0.110
Cyanobacteria	Nostocophycideae	Nostocales	Nostocaceae	*Nostoc*		388	0.108
Proteobacteria	Alphaproteobacteria	Rhodospirillales				380	0.106
Actinobacteria	Actinobacteria	Actinomycetales	Cellulomonadaceae	*Pseudoclavibacter*		379	0.105
Proteobacteria	Alphaproteobacteria	Sphingomonadales	Sphingomonadaceae			345	0.096
Actinobacteria	Actinobacteria	Actinomycetales	Kineosporiaceae	*Kineosporia*		341	0.095
Actinobacteria	Actinobacteria	Actinomycetales	Thermomonosporaceae	*Actinoallomurus*		331	0.092
Firmicutes	Clostridia	Clostridiales	Clostridiaceae	*Clostridium*		322	0.089
Actinobacteria	Actinobacteria	Actinomycetales	Microbacteriaceae	*Cryobacterium*	*psychrophilum*	320	0.089
Cyanobacteria	Nostocophycideae	Nostocales	Nostocaceae	*Dolichospermum*		319	0.089
Bacteroidetes	Sphingobacteriia	Sphingobacteriales				309	0.086
Proteobacteria	Betaproteobacteria	Burkholderiales	Oxalobacteraceae			300	0.083
Cyanobacteria	Synechococcophycideae	Pseudanabaenales	Pseudanabaenaceae			290	0.081
Actinobacteria	Actinobacteria	Actinomycetales	Microbacteriaceae	*Agromyces*	*salentinus*	288	0.080
Actinobacteria	Actinobacteria	Actinomycetales	Nocardiaceae	*Rhodococcus*	*baikonurensis*	287	0.080
Proteobacteria	Betaproteobacteria	Burkholderiales	Comamonadaceae	*Polaromonas*	*jejuensis*	285	0.079
Actinobacteria	Actinobacteria	Actinomycetales	Pseudonocardiaceae	*Saccharopolyspora*		283	0.079
Actinobacteria	Actinobacteria	Actinomycetales	Streptosporangiaceae	*Streptosporangium*		276	0.077
Proteobacteria	Alphaproteobacteria	Rhodospirillales	Acetobacteraceae	*Gluconobacter*		264	0.073
Actinobacteria	Actinobacteria	Actinomycetales	Pseudonocardiaceae	*Pseudonocardia*	*kongjuensis*	262	0.073
Actinobacteria	Actinobacteria	Actinomycetales	Promicromonosporaceae	*Isoptericola*		258	0.072
Actinobacteria	Actinobacteria	Actinomycetales	Pseudonocardiaceae	*Pseudonocardia*	*compacta*	237	0.066
Proteobacteria	Betaproteobacteria	Burkholderiales	Comamonadaceae	*Variovorax*	*ginsengisoli*	237	0.066

Bacteroidetes	Sphingobacteriia	Sphingobacteriales	Sphingobacteriaceae	*Pedobacter*		237	0.066
Firmicutes	Clostridia	Clostridiales	Clostridiaceae			231	0.064
Proteobacteria	Betaproteobacteria	Neisseriales	Neisseriaceae	*Vogesella*	*perlucida*	229	0.064
Firmicutes	Bacilli	Lactobacillales	Aerococcaceae	*Alkalibacterium*		218	0.061
Bacteroidetes	Sphingobacteriia	Sphingobacteriales	Flexibacteraceae	*Spirosoma*	*rigui*	216	0.060
Firmicutes	Clostridia	Clostridiales	Clostridiaceae	*Caloramator*	*mitchellensis*	216	0.060
Bacteroidetes	Sphingobacteriia	Sphingobacteriales	Chitinophagaceae	*Niastella*	*koreensis*	213	0.059
Actinobacteria	Actinobacteria	Actinomycetales	Microbacteriaceae	*Leucobacter*		213	0.059
Actinobacteria	Actinobacteria	Actinomycetales	Streptomycetaceae			207	0.058
Actinobacteria	Acidimicrobiia	Acidimicrobiales	Acidimicrobiaceae	*Acidimicrobium*		206	0.057
Actinobacteria	Actinobacteria	Actinomycetales	Cellulomonadaceae	*Cellulomonas*	*denverensis*	198	0.055
Actinobacteria	Actinobacteria					189	0.053
Proteobacteria	Alphaproteobacteria	Rhodospirillales	Acetobacteraceae	*Roseomonas*	*massiliensis*	188	0.052
Proteobacteria	Betaproteobacteria	Burkholderiales	Comamonadaceae	*Variovorax*		188	0.052
Firmicutes	Clostridia	Clostridiales	Heliobacteriaceae	*Heliorestis*		186	0.052
Actinobacteria	Actinobacteria	Actinomycetales	Gordoniaceae	*Gordonia*	*jacobaea*	184	0.051
Actinobacteria	Actinobacteria	Actinomycetales	Micromonosporaceae	*Dactylosporangium*	*maewongense*	180	0.050
Proteobacteria	Betaproteobacteria	Methylophilales	Methylophilaceae	*Methylotenera*	*mobilis*	174	0.048
Cyanobacteria	Oscillatoriophycideae	Chroococcales	Phormidiaceae	*Microcoleus*	*antarcticus*	170	0.047
Actinobacteria	Actinobacteria	Actinomycetales	Pseudonocardiaceae	*Saccharopolyspora*	*cebuensis*	170	0.047
Firmicutes	Bacilli	Bacillales	Bacillaceae	*Virgibacillus*	*salexigens*	163	0.045
Actinobacteria	Actinobacteria	Actinomycetales	Cryptosporangiaceae	*Cryptosporangium*		162	0.045
Proteobacteria	Betaproteobacteria	Burkholderiales	Oxalobacteraceae	*Herminiimonas*		160	0.044
Proteobacteria	Gammaproteobacteria					156	0.043
Actinobacteria	Actinobacteria	Actinomycetales	Glycomycetaceae	*Glycomyces*		156	0.043
Proteobacteria	Alphaproteobacteria					156	0.043
Actinobacteria	Actinobacteria	Actinomycetales	Micromonosporaceae	*Micromonospora*		155	0.043
Firmicutes	Clostridia	Clostridiales	Peptococcaceae	*Peptococcus*	*niger*	155	0.043
Actinobacteria	Actinobacteria	Actinomycetales	Nocardioidaceae	*Kribbella*	*ginsengisoli*	144	0.040
Firmicutes	Bacilli	Lactobacillales	Leuconostocaceae	*Leuconostoc*		141	0.039
Cyanobacteria	Oscillatoriophycideae	Chroococcales	Gomphosphaeriaceae	*Snowella*	*rosea*	140	0.039
Proteobacteria	Alphaproteobacteria	Sphingomonadales	Sphingomonadaceae	*Sphingomonas*	*oligophenolica*	138	0.038
Firmicutes	Clostridia	Clostridiales	Clostridiaceae	*Clostridium*	*carboxidivorans*	135	0.038
Bacteroidetes	Flavobacteriia	Flavobacteriales	Flavobacteriaceae	*Flavobacterium*		134	0.037
Actinobacteria	Actinobacteria	Actinomycetales	Cellulomonadaceae	*Oerskovia*	*enterophila*	132	0.037
Proteobacteria	Deltaproteobacteria	Myxococcales	Polyangiaceae	*Chondromyces*	*pediculatus*	132	0.037
Proteobacteria	Alphaproteobacteria	Rhodospirillales	Acetobacteraceae	*Gluconobacter*	*krungthepensis*	131	0.036

Actinobacteria	Actinobacteria	Actinomycetales	Cellulomonadaceae	*Demequina*	*aurantiaca*	129	0.036
Proteobacteria	Deltaproteobacteria	Desulfuromonadales	Desulfuromonadaceae	*Desulfuromusa*	*succinoxidans*	129	0.036
Proteobacteria	Deltaproteobacteria	Desulfuromonadales	Desulfuromonadaceae	*Desulfuromusa*		126	0.035
Actinobacteria	Actinobacteria	Actinomycetales	Thermomonosporaceae	*Actinoallomurus*	*purpureus*	125	0.035
Actinobacteria	Actinobacteria	Actinomycetales	Intrasporangiaceae	*Phycicoccus*		125	0.035
Proteobacteria	Betaproteobacteria	Neisseriales	Neisseriaceae			123	0.034
Actinobacteria	Actinobacteria	Actinomycetales	Thermomonosporaceae	*Actinomadura*	*maheshkhaliensis*	120	0.033
Proteobacteria	Betaproteobacteria	Burkholderiales	Comamonadaceae	*Curvibacter*	*gracilis*	120	0.033
Proteobacteria	Gammaproteobacteria	Xanthomonadales	Xanthomonadaceae			119	0.033
Bacteroidetes	Sphingobacteriia	Sphingobacteriales	Chitinophagaceae	*Flavisolibacter*	*ginsengisoli*	119	0.033
Proteobacteria	Alphaproteobacteria	Sphingomonadales	Sphingomonadaceae	*Sphingomonas*	*elodea*	118	0.033
Proteobacteria	Betaproteobacteria	Rhodocyclales	Rhodocyclaceae			118	0.033
Proteobacteria	Betaproteobacteria	Burkholderiales	Burkholderiaceae	*Burkholderia*	*ubonensis*	109	0.030
Actinobacteria	Actinobacteria	Actinomycetales	Microbacteriaceae	*Agrococcus*	*versicolor*	109	0.030
Actinobacteria	Actinobacteria	Bifidobacteriales	Bifidobacteriaceae	*Bifidobacterium*	*bombi*	108	0.030
Firmicutes	Bacilli	Bacillales				106	0.029
Bacteroidetes	Flavobacteriia	Flavobacteriales	Flavobacteriaceae			106	0.029
Actinobacteria	Actinobacteria	Actinomycetales	Microbacteriaceae	*Rathayibacter*		103	0.029
Actinobacteria	Nitriliruptoria	Euzebyales	Euzebyaceae	*Euzebya*	*tangerina*	100	0.028
Proteobacteria	Gammaproteobacteria	Xanthomonadales	Xanthomonadaceae	*Stenotrophomonas*		100	0.028
Proteobacteria	Gammaproteobacteria	Alteromonadales	Shewanellaceae	*Shewanella*		99	0.028
Proteobacteria	Deltaproteobacteria					99	0.028
Proteobacteria	Gammaproteobacteria	Legionellales	Coxiellaceae	*Rickettsiella*		99	0.028
Proteobacteria	Betaproteobacteria	Burkholderiales	Oxalobacteraceae	*Polynucleobacter*		97	0.027
Actinobacteria	Actinobacteria	Actinomycetales	Streptomycetaceae	*Streptomyces*	*lazureus*	97	0.027
Cyanobacteria	Oscillatoriophycideae	Chroococcales	Chroococcaceae	*Chroococcus*	*minutus*	93	0.026
Proteobacteria	Alphaproteobacteria	Sphingomonadales	Sphingomonadaceae	*Novosphingobium*		91	0.025
Actinobacteria	Actinobacteria	Actinomycetales	Microbacteriaceae	*Cryocola*	*antiquus*	90	0.025
Actinobacteria	Actinobacteria	Actinomycetales	Microbacteriaceae	*Leucobacter*	*chromiiresistens*	88	0.024
Proteobacteria	Betaproteobacteria	Burkholderiales	Burkholderiaceae	*Burkholderia*		87	0.024
Actinobacteria	Actinobacteria	Actinomycetales	Actinomycetaceae	*Actinomyces*	*naturae*	86	0.024
Actinobacteria	Actinobacteria	Actinomycetales	Intrasporangiaceae	*Janibacter*	*hoylei*	86	0.024
Actinobacteria	Thermoleophilia	Solirubrobacterales	Conexibacteraceae	*Conexibacter*		85	0.024
Proteobacteria	Alphaproteobacteria	Caulobacterales	Caulobacteraceae	*Phenylobacterium*		84	0.023
Actinobacteria	Actinobacteria	Actinomycetales	Nocardiaceae	*Rhodococcus*	*kyotonensis*	84	0.023
Proteobacteria	Alphaproteobacteria	Rickettsiales	Rickettsiaceae	*Rickettsia*		83	0.023
Actinobacteria	Actinobacteria	Actinomycetales	Cellulomonadaceae	*Cellulomonas*		82	0.023

Proteobacteria	Alphaproteobacteria	Caulobacterales	Caulobacteraceae	*Phenylobacterium*	*lituiforme*	81	0.023
Proteobacteria	Alphaproteobacteria	Rhodospirillales	Rhodospirillaceae			79	0.022
Proteobacteria	Epsilonproteobacteria	Campylobacterales	Campylobacteraceae	*Arcobacter*	*marinus*	76	0.021
Actinobacteria	Actinobacteria	Actinomycetales	Microbacteriaceae	*Microbacterium*	*lacus*	76	0.021
Cyanobacteria	Oscillatoriophycideae	Chroococcales	Phormidiaceae	*Oscillatoria*	*corallinae*	75	0.021
Firmicutes	Clostridia	Clostridiales	Clostridiaceae	*Clostridium*	*frigoris*	72	0.020
Firmicutes	Bacilli	Bacillales	Bacillaceae	*Bacillus*		72	0.020
Actinobacteria	Actinobacteria	Actinomycetales	Mycobacteriaceae	*Mycobacterium*		71	0.020
Actinobacteria	Actinobacteria	Actinomycetales	Microbacteriaceae	*Leucobacter*	*chironomi*	71	0.020
Cyanobacteria	Oscillatoriophycideae	Chroococcales	Phormidiaceae	*Trichodesmium*		70	0.019
Firmicutes	Clostridia					69	0.019
Actinobacteria	Actinobacteria	Actinomycetales	Microbacteriaceae	*Curtobacterium*	*pusillum*	69	0.019
Proteobacteria	Betaproteobacteria	Burkholderiales	Comamonadaceae	*Hydrogenophaga*	*defluvii*	69	0.019
Actinobacteria	Actinobacteria	Actinomycetales	Nocardiaceae			67	0.019
Proteobacteria	Betaproteobacteria	Rhodocyclales	Rhodocyclaceae	*Methyloversatilis*	*universalis*	67	0.019
Firmicutes	Clostridia	Clostridiales	Eubacteriaceae	*Acetobacterium*		66	0.018
Actinobacteria	Actinobacteria	Actinomycetales	Microbacteriaceae	*Microbacterium*	*marinilacus*	66	0.018
Proteobacteria	Alphaproteobacteria	Sphingomonadales	Sphingomonadaceae	*Sphingomonas*	*fennica*	66	0.018
Actinobacteria	Actinobacteria	Actinomycetales	Intrasporangiaceae	*Serinicoccus*	*chungangensis*	66	0.018
Actinobacteria	Actinobacteria	Actinomycetales	Actinosynnemataceae	*Lentzea*		66	0.018
Actinobacteria	Actinobacteria	Actinomycetales	Intrasporangiaceae	*Terracoccus*	*luteus*	64	0.018
Actinobacteria	Actinobacteria	Actinomycetales	Streptosporangiaceae			63	0.018
Proteobacteria	Gammaproteobacteria	Alteromonadales	Alteromonadaceae	*Marinobacter*	*arcticus*	62	0.017
Cyanobacteria	Oscillatoriophycideae	Chroococcales	Phormidiaceae	*Hydrocoleum*	*lyngbyaceum*	62	0.017
Actinobacteria	Actinobacteria	Actinomycetales	Microbacteriaceae	*Microbacterium*	*liquefaciens*	61	0.017
Proteobacteria	Betaproteobacteria	Burkholderiales	Oxalobacteraceae	*Ralstonia*	*detusculanense*	61	0.017
Bacteroidetes	Bacteroidia	Bacteroidales	Bacteroidaceae	*Bacteroides*		61	0.017
Cyanobacteria	Oscillatoriophycideae	Oscillatoriales	Phormidiaceae			60	0.017
Proteobacteria	Alphaproteobacteria	Sphingomonadales	Sphingomonadaceae	*Sphingomonas*	*sanxanigenens*	58	0.016
Acidobacteria	Solibacteres	Solibacterales	Solibacteraceae	*Candidatus Solibacter*		58	0.016
Proteobacteria	Gammaproteobacteria	Chromatiales	Chromatiaceae			58	0.016
Proteobacteria	Betaproteobacteria	Burkholderiales	Comamonadaceae	*Acidovorax*	*wohlfahrtii*	57	0.016
Actinobacteria	Actinobacteria	Actinomycetales	Cellulomonadaceae	*Oerskovia*	*paurometabola*	56	0.016
Bacteroidetes	Sphingobacteriia	Sphingobacteriales	Flexibacteraceae	*Runella*	*limosa*	55	0.015
Actinobacteria	Actinobacteria	Actinomycetales	Cellulomonadaceae	*Demequina*		55	0.015
Proteobacteria	Alphaproteobacteria	Rhodobacterales	Rhodobacteraceae	*Rhodobacter*		54	0.015
Cyanobacteria	Oscillatoriophycideae	Chroococcales	Microcystaceae	*Microcystis*	*panniformis*	52	0.014

Proteobacteria	Gammaproteobacteria	Chromatiales				52	0.014
Proteobacteria	Alphaproteobacteria	Rhodospirillales	Rhodospirillaceae	*Roseospira*		50	0.014
Proteobacteria	Alphaproteobacteria	Rhodobacterales	Hyphomonadaceae	*Maricaulis*	*indicus*	50	0.014
Actinobacteria	Actinobacteria	Actinomycetales	Nocardiaceae	*Rhodococcus*	*maanshanensis*	49	0.014
Proteobacteria	Alphaproteobacteria	Sphingomonadales	Sphingomonadaceae	*Sphingomonas*	*echinoides*	48	0.013
Proteobacteria	Alphaproteobacteria	Sphingomonadales	Sphingomonadaceae	*Novosphingobium*	*acidiphilum*	48	0.013
Bacteroidetes	Sphingobacteriia	Sphingobacteriales	Amoebophilaceae	*Candidatus Amoebophilus*	*asiaticus*	48	0.013
Proteobacteria	Epsilonproteobacteria	Campylobacterales	Campylobacteraceae	*Sulfurospirillum*		48	0.013
Actinobacteria	Acidimicrobiia	Acidimicrobiales	Iamiaceae	*Iamia*		47	0.013
Actinobacteria	Actinobacteria	Actinomycetales	Bogoriellaceae	*Georgenia*		47	0.013
Actinobacteria	Actinobacteria	Actinomycetales	Microbacteriaceae	*Agrococcus*	*terreus*	46	0.013
Proteobacteria	Alphaproteobacteria	Rhizobiales				46	0.013
Proteobacteria	Betaproteobacteria	Burkholderiales	Burkholderiaceae	*Lautropia*	*mirabilis*	46	0.013
Proteobacteria	Betaproteobacteria	Burkholderiales	Comamonadaceae	*Thiomonas*	*thermosulfata*	46	0.013
Proteobacteria	Gammaproteobacteria	Thiotrichales	Thiotrichaceae	*Thiothrix*		46	0.013
Actinobacteria	Actinobacteria	Actinomycetales	Pseudonocardiaceae	*Pseudonocardia*	*acaciae*	45	0.013
Actinobacteria	Actinobacteria	Actinomycetales	Micromonosporaceae	*Actinocatenispora*	*silicis*	45	0.013
Actinobacteria	Actinobacteria	Actinomycetales	Nocardioidaceae	*Aeromicrobium*	*marinum*	44	0.012
Firmicutes	Bacilli					43	0.012
Proteobacteria	Alphaproteobacteria	Rhodospirillales	Acetobacteraceae	*Gluconobacter*	*kondonii*	43	0.012
Actinobacteria	Actinobacteria	Actinomycetales	Pseudonocardiaceae			43	0.012
Proteobacteria	Alphaproteobacteria	Sphingomonadales	Sphingomonadaceae	*Sphingomonas*	*soli*	42	0.012
Cyanobacteria	Nostocophycideae	Nostocales	Nostocaceae	*Nostoc*	*entophytum*	42	0.012
Actinobacteria	Actinobacteria	Actinomycetales	Sanguibacteraceae	*Sanguibacter*	*suarezii*	42	0.012
Proteobacteria	Betaproteobacteria	Methylophilales	Methylophilaceae	*Methylotenera*	*versatilis*	41	0.011
Actinobacteria	Actinobacteria	Actinomycetales	Microbacteriaceae	*Microbacterium*		41	0.011
Actinobacteria	Actinobacteria	Actinomycetales	Pseudonocardiaceae	*Pseudonocardia*	*xinjiangensis*	41	0.011
Nitrospirae	Nitrospira	Nitrospirales	Thermodesulfovibrionaceae	*Thermodesulfovibrio*	*thiophilus*	41	0.011
Firmicutes	Bacilli	Bacillales	Bacillaceae	*Geobacillus*		40	0.011
Proteobacteria	Betaproteobacteria	Burkholderiales	Comamonadaceae	*Leptothrix*	*discophora*	40	0.011
Proteobacteria	Betaproteobacteria	Rhodocyclales	Rhodocyclaceae	*Hydrogenophilus*		40	0.011
Actinobacteria	Actinobacteria	Actinomycetales	Microbacteriaceae	*Agromyces*		40	0.011
Actinobacteria	Actinobacteria	Actinomycetales	Cellulomonadaceae	*Cellulomonas*	*cellasea*	39	0.011
Proteobacteria	Gammaproteobacteria	Oceanospirillales	Halomonadaceae	*Kushneria*	*indalinina*	39	0.011
Bacteroidetes	Sphingobacteriia	Sphingobacteriales	Sphingobacteriaceae	*Pedobacter*	*kwangyangensis*	38	0.011
Firmicutes	Bacilli	Bacillales	Paenibacillaceae	*Cohnella*	*soli*	38	0.011
Bacteroidetes	Sphingobacteriia	Sphingobacteriales	Rhodothermaceae	*Rhodothermus*	*clarus*	37	0.010

Bacteroidetes	Flavobacteriia	Flavobacteriales	Flavobacteriaceae	*Chryseobacterium*	*taichungense*	37	0.010
Actinobacteria	Actinobacteria	Actinomycetales	Pseudonocardiaceae	*Pseudonocardia*	*saturnea*	37	0.010
Proteobacteria	Deltaproteobacteria	Desulfovibrionales	Desulfovibrionaceae	*Desulfovibrio*		36	0.010
Actinobacteria	Actinobacteria	Actinomycetales	Cellulomonadaceae	*Oerskovia*	*ginkgo*	36	0.010
Proteobacteria	Betaproteobacteria	Burkholderiales	Comamonadaceae	*Pelomonas*	*saccharophila*	35	0.010
Proteobacteria	Alphaproteobacteria	Rhodospirillales	Acetobacteraceae	*Acidiphilium*		35	0.010
Proteobacteria	Alphaproteobacteria	Rhizobiales	Methylobacteriaceae	*Methylobacterium*		35	0.010
Firmicutes	Bacilli	Bacillales	Paenibacillaceae	*Cohnella*		35	0.010
Proteobacteria	Alphaproteobacteria	Rhodospirillales	Acetobacteraceae	*Acidiphilium*	*acidophilum*	35	0.010
Actinobacteria	Actinobacteria	Actinomycetales	Nocardiaceae	*Nocardia*		35	0.010
Armatimonadetes	Chthonomonadetes	Chthonomonadales	Chthonomonadaceae	*Chthonomonas*		34	0.009
Proteobacteria	Deltaproteobacteria	Myxococcales	Nannocystaceae	*Nannocystis*		34	0.009
Proteobacteria	Alphaproteobacteria	Sphingomonadales	Sphingomonadaceae	*Novosphingobium*	*stygium*	34	0.009
Firmicutes	Bacilli	Bacillales	Bacillaceae			33	0.009
Proteobacteria	Alphaproteobacteria	Rhizobiales	Rhizobiaceae	*Agrobacterium*	*albertimagni*	33	0.009
Proteobacteria	Alphaproteobacteria	Rhizobiales	Bradyrhizobiaceae	*Bradyrhizobium*		33	0.009
Bacteroidetes	Sphingobacteriia	Sphingobacteriales	Flexibacteraceae	*Hymenobacter*	*chitinivorans*	33	0.009
Actinobacteria	Actinobacteria	Actinomycetales	Pseudonocardiaceae	*Actinomycetospora*		33	0.009
Firmicutes	Clostridia	Clostridiales	Clostridiaceae	*Clostridium*	*aestuarii*	32	0.009
Firmicutes	Clostridia	Thermoanaerobacterales	Thermoanaerobacteraceae			32	0.009
Firmicutes	Bacilli	Thermicanales	Thermicanaceae	*Thermicanus*		32	0.009
Proteobacteria	Gammaproteobacteria	Legionellales	Legionellaceae	*Legionella*		32	0.009
Proteobacteria	Betaproteobacteria	Burkholderiales	Comamonadaceae	*Rhodoferax*		31	0.009
Firmicutes	Clostridia	Clostridiales	Clostridiaceae	*Alkaliphilus*		31	0.009
Proteobacteria	Gammaproteobacteria	Pseudomonadales	Moraxellaceae	*Acinetobacter*	*tjernbergiae*	31	0.009
Actinobacteria	Actinobacteria	Actinomycetales	Nocardiopsaceae	*Nocardiopsis*	*terrae*	31	0.009
Proteobacteria	Betaproteobacteria	Burkholderiales	Comamonadaceae	*Thiomonas*	*perometabolis*	30	0.008
Firmicutes	Bacilli	Gemellales	Gemellaceae	*Gemella*	*cunicula*	30	0.008
Proteobacteria	Deltaproteobacteria	Desulfuromonadales				30	0.008
Actinobacteria	Actinobacteria	Actinomycetales	Nocardioidaceae	*Aeromicrobium*	*ponti*	30	0.008
Bacteroidetes	Sphingobacteriia	Sphingobacteriales	Flammeovirgaceae	*Flammeovirga*	*pacifica*	30	0.008
Actinobacteria	Actinobacteria	Actinomycetales	Cellulomonadaceae	*Cellulomonas*	*composti*	29	0.008
Proteobacteria	Alphaproteobacteria	Rhizobiales	Bradyrhizobiaceae	*Bosea*		29	0.008
Firmicutes	Clostridia	Thermoanaerobacterales	Thermoanaerobacteraceae	*Tepidanaerobacter*	*syntrophicus*	28	0.008
Cyanobacteria	Synechococcophycideae	Pseudanabaenales	Pseudanabaenaceae	*Leptolyngbya*	*antarctica*	28	0.008
Proteobacteria	Alphaproteobacteria	Rhodospirillales	Rhodospirillaceae	*Azospirillum*	*rugosum*	28	0.008
Proteobacteria	Betaproteobacteria	Burkholderiales	Burkholderiaceae	*Burkholderia*	*brasilensis*	28	0.008

Bacteroidetes	Sphingobacteriia	Sphingobacteriales	Flexibacteraceae	*Hymenobacter*	*aerophilus*	27	0.008
Actinobacteria	Actinobacteria	Actinomycetales	Actinosynnemataceae	*Actinokineospora*	*inagensis*	27	0.008
Proteobacteria	Alphaproteobacteria	Rhizobiales	Phyllobacteriaceae	*Phyllobacterium*		27	0.008
Proteobacteria	Gammaproteobacteria	Xanthomonadales	Sinobacteraceae	*Hydrocarboniphaga*		26	0.007
Proteobacteria	Alphaproteobacteria	Sphingomonadales	Sphingomonadaceae	*Sphingobium*	*abikonense*	26	0.007
Proteobacteria	Alphaproteobacteria	Rhodobacterales	Rhodobacteraceae	*Rhodobacter*	*ovatus*	26	0.007
Actinobacteria	Actinobacteria	Actinomycetales	Thermomonosporaceae	*Actinomadura*		26	0.007
Bacteroidetes	Flavobacteriia	Flavobacteriales	Flavobacteriaceae	*Chryseobacterium*		26	0.007
Actinobacteria	Actinobacteria	Actinomycetales	Intrasporangiaceae	*Tetrasphaera*	*vanveenii*	25	0.007
Actinobacteria	Actinobacteria	Actinomycetales	Cellulomonadaceae			25	0.007
Actinobacteria	Actinobacteria	Actinomycetales	Streptomycetaceae	*Streptacidiphilus*		25	0.007
Firmicutes	Bacilli	Lactobacillales				25	0.007
Actinobacteria	Actinobacteria	Actinomycetales	Microbacteriaceae	*Yonghaparkia*		25	0.007
Proteobacteria	Alphaproteobacteria	Sphingomonadales	Sphingomonadaceae	*Novosphingobium*	*taihuense*	25	0.007
Actinobacteria	Actinobacteria	Actinomycetales	Propionibacteriaceae			24	0.007
Firmicutes	Bacilli	Bacillales	Staphylococcaceae	*Staphylococcus*		24	0.007
Deferribacteres	Deferribacteres	Deferribacterales	Deferribacteraceae	*Deferribacter*	*autotrophicus*	24	0.007
Actinobacteria	Actinobacteria	Actinomycetales	Nocardiaceae	*Rhodococcus*	*imtechensis*	24	0.007
Firmicutes	Clostridia	Clostridiales	Veillonellaceae	*Megasphaera*	*hominis*	24	0.007
Bacteroidetes	Sphingobacteriia	Sphingobacteriales	Sphingobacteriaceae	*Olivibacter*		23	0.006
Proteobacteria	Betaproteobacteria	Burkholderiales	Comamonadaceae	*Comamonas*		23	0.006
Proteobacteria	Alphaproteobacteria	Sphingomonadales	Sphingomonadaceae	*Sphingomonas*	*wittichii*	23	0.006
Firmicutes	Bacilli	Lactobacillales	Lactobacillaceae	*Lactobacillus*		23	0.006
Proteobacteria	Gammaproteobacteria	Alteromonadales				23	0.006
Proteobacteria	Alphaproteobacteria	Rhizobiales	Rhizobiaceae	*Candidatus Liberibacter*		23	0.006
Actinobacteria	Actinobacteria	Actinomycetales	Streptomycetaceae	*Kitasatospora*		23	0.006
Actinobacteria	Actinobacteria	Actinomycetales	Actinosynnemataceae	*Lentzea*	*flavoverrucoides*	22	0.006
Proteobacteria	Epsilonproteobacteria	Campylobacterales	Helicobacteraceae	*Helicobacter*	*suncus*	22	0.006
Firmicutes	Clostridia	Clostridiales	Peptococcaceae	*Desulfotomaculum*	*indicum*	22	0.006
Actinobacteria	Actinobacteria	Actinomycetales	Thermomonosporaceae			22	0.006
Cyanobacteria	Synechococcophycideae	Pseudanabaenales	Pseudanabaenaceae	*Leptolyngbya*	*laminosa*	21	0.006
Actinobacteria	Actinobacteria	Actinomycetales	Corynebacteriaceae	*Corynebacterium*		21	0.006
Actinobacteria	Actinobacteria	Actinomycetales	Micrococcaceae			21	0.006
Proteobacteria	Alphaproteobacteria	Sphingomonadales	Sphingomonadaceae	*Novosphingobium*	*mathurense*	21	0.006
Bacteroidetes	Sphingobacteriia	Sphingobacteriales	Sphingobacteriaceae			21	0.006
Proteobacteria	Gammaproteobacteria	Xanthomonadales	Xanthomonadaceae	*Luteibacter*	*anthropi*	21	0.006
Proteobacteria	Gammaproteobacteria	Enterobacteriales	Enterobacteriaceae			21	0.006

Actinobacteria	Actinobacteria	Actinomycetales	Nocardioidaceae	*Nocardioides*	*islandensis*	21	0.006
Proteobacteria	Alphaproteobacteria	Rhodospirillales	Rhodospirillaceae	*Azospirillum*		20	0.006
Firmicutes	Bacilli	Bacillales	Paenibacillaceae	*Cohnella*	*damuensis*	20	0.006
Actinobacteria	Actinobacteria	Actinomycetales	Cellulomonadaceae	*Actinotalea*		20	0.006
Firmicutes	Clostridia	Clostridiales	Clostridiaceae	*Alkaliphilus*	*crotonatoxidans*	20	0.006
Proteobacteria	Betaproteobacteria	Burkholderiales	Comamonadaceae	*Rhodoferax*	*antarcticus*	20	0.006
Bacteroidetes	Sphingobacteriia	Sphingobacteriales	Flexibacteraceae	*Dyadobacter*		20	0.006
Firmicutes	Bacilli	Bacillales	Paenibacillaceae	*Paenibacillus*		20	0.006
Actinobacteria	Actinobacteria	Bifidobacteriales	Bifidobacteriaceae			20	0.006
Actinobacteria	Actinobacteria	Actinomycetales	Micromonosporaceae			20	0.006
Proteobacteria	Betaproteobacteria	Burkholderiales	Comamonadaceae	*Comamonas*	*composti*	19	0.005
Actinobacteria	Actinobacteria	Actinomycetales	Streptomycetaceae	*Streptacidiphilus*	*griseus*	19	0.005
Proteobacteria	Betaproteobacteria	Neisseriales	Neisseriaceae	*Aquitalea*	*denitrificans*	19	0.005
Proteobacteria	Alphaproteobacteria	Sphingomonadales	Sphingomonadaceae	*Sphingobium*		19	0.005
Bacteroidetes	Sphingobacteriia	Sphingobacteriales	Saprospiraceae	*Lewinella*		19	0.005
Chloroflexi	Chloroflexi	Roseiflexales	Kouleothrixaceae	*Kouleothrix*		19	0.005
Proteobacteria	Gammaproteobacteria	Xanthomonadales	Xanthomonadaceae	*Arenimonas*	*malthae*	19	0.005
Proteobacteria	Alphaproteobacteria	Rhodospirillales	Rhodospirillaceae	*Rhodospirillum*		18	0.005
Actinobacteria	Actinobacteria	Actinomycetales	Geodermatophilaceae	*Modestobacter*	*versicolor*	18	0.005
Proteobacteria	Deltaproteobacteria	Bdellovibrionales	Bdellovibrionaceae	*Bdellovibrio*	*exovorus*	18	0.005
Firmicutes	Bacilli	Bacillales	Bacillaceae	*Bacillus*	*horneckiae*	18	0.005
Proteobacteria	Deltaproteobacteria	Desulfovibrionales	Desulfonatronumaceae	*Desulfonatronum*	*thiosulfatophilum*	18	0.005
Proteobacteria	Gammaproteobacteria	Alteromonadales	Pseudoalteromonadaceae	*Pseudoalteromonas*		18	0.005
Proteobacteria	Alphaproteobacteria	Rhizobiales	Xanthobacteraceae	*Labrys*		18	0.005
Proteobacteria	Gammaproteobacteria	Pseudomonadales	Moraxellaceae	*Acinetobacter*		17	0.005
Bacteroidetes	Flavobacteriia	Flavobacteriales	Flavobacteriaceae	*Tenacibaculum*	*japonica*	17	0.005
Actinobacteria	Actinobacteria	Actinomycetales	Thermomonosporaceae	*Actinoallomurus*	*yoronensis*	17	0.005
Proteobacteria	Betaproteobacteria	Methylophilales	Methylophilaceae	*Methylotenera*		17	0.005
Proteobacteria	Alphaproteobacteria	Rhodobacterales	Rhodobacteraceae	*Phaeobacter*		17	0.005
Firmicutes	Bacilli	Bacillales	Staphylococcaceae	*Macrococcus*		17	0.005
Firmicutes	Clostridia	Thermoanaerobacterales	Thermoanaerobacteraceae	*Moorella*		17	0.005
Actinobacteria	Actinobacteria	Actinomycetales	Streptomycetaceae	*Streptacidiphilus*	*thailandensis*	17	0.005
Proteobacteria	Gammaproteobacteria	Pseudomonadales	Pseudomonadaceae	*Pseudomonas*	*clemancea*	17	0.005
Proteobacteria	Epsilonproteobacteria	Campylobacterales	Campylobacteraceae	*Arcobacter*	*skirrowii*	16	0.004
Proteobacteria	Deltaproteobacteria	Desulfovibrionales	Desulfovibrionaceae	*Desulfovibrio*	*psychrotolerans*	16	0.004
Actinobacteria	Actinobacteria	Actinomycetales	Nocardiaceae	*Rhodococcus*	*opacus*	16	0.004
Proteobacteria	Alphaproteobacteria	Rhizobiales	Hyphomicrobiaceae	*Hyphomicrobium*	*aestuarii*	16	0.004

Cyanobacteria	Oscillatoriophycideae	Oscillatoriales	Phormidiaceae	*Phormidium*	*murrayi*	16	0.004
Proteobacteria	Betaproteobacteria	Burkholderiales	Comamonadaceae	*Azohydromonas*		16	0.004
Actinobacteria	Actinobacteria	Actinomycetales	Actinosynnemataceae	*Actinoalloteichus*		16	0.004
Actinobacteria	Actinobacteria	Bifidobacteriales	Bifidobacteriaceae	*Bifidobacterium*		16	0.004
Actinobacteria	Actinobacteria	Actinomycetales	Microbacteriaceae	*Microbacterium*	*kribbense*	15	0.004
Proteobacteria	Epsilonproteobacteria	Campylobacterales	Campylobacteraceae	*Arcobacter*		15	0.004
Proteobacteria	Gammaproteobacteria	Chromatiales	Ectothiorhodospiraceae	*Ectothiorhodospira*	*imhoffii*	15	0.004
Firmicutes	Clostridia	Clostridiales	Peptococcaceae	*Desulfosporosinus*	*lacus*	15	0.004
Firmicutes	Clostridia	Clostridiales	Peptococcaceae	*Desulfosporosinus*	*meridiei*	15	0.004
Proteobacteria	Alphaproteobacteria	Rhodobacterales	Rhodobacteraceae	*Paracoccus*		15	0.004
Proteobacteria	Alphaproteobacteria	Rhodospirillales	Acetobacteraceae	*Acidisphaera*		15	0.004
Proteobacteria	Alphaproteobacteria	Caulobacterales	Caulobacteraceae			15	0.004
Proteobacteria	Alphaproteobacteria	Sphingomonadales	Sphingomonadaceae	*Sphingomonas*	*japonica*	15	0.004
Proteobacteria	Betaproteobacteria	Methylophilales	Methylophilaceae	*Methylobacillus*	*glycogenes*	15	0.004
Proteobacteria	Betaproteobacteria	Rhodocyclales	Rhodocyclaceae	*Rhodocyclus*	*purpureus*	14	0.004
Proteobacteria	Gammaproteobacteria	Xanthomonadales	Xanthomonadaceae	*Dokdonella*	*fugitiva*	14	0.004
Actinobacteria	Actinobacteria	Actinomycetales	Brevibacteriaceae	*Brevibacterium*		14	0.004
Proteobacteria	Betaproteobacteria	Rhodocyclales	Rhodocyclaceae	*Denitratisoma*	*oestradiolicum*	14	0.004
Actinobacteria	Actinobacteria	Actinomycetales	Microbacteriaceae	*Frigoribacterium*	*faeni*	14	0.004
Proteobacteria	Gammaproteobacteria	Chromatiales	Chromatiaceae	*Thiocapsa*		14	0.004
Proteobacteria	Gammaproteobacteria	Thiohalorhabdales	Thiohalorhabdaceae	*Thiohalorhabdus*	*denitrificans*	14	0.004
Actinobacteria	Actinobacteria	Bifidobacteriales	Bifidobacteriaceae	*Bifidobacterium*	*subtile*	14	0.004
Firmicutes	Clostridia	Thermoanaerobacterales				14	0.004
Proteobacteria	Betaproteobacteria	Hydrogenophilales	Hydrogenophilaceae	*Thiobacillus*	*sajanensis*	14	0.004
Bacteroidetes	Flavobacteriia	Flavobacteriales	Flavobacteriaceae	*Flavobacterium*	*reichenbachii*	14	0.004
Proteobacteria	Gammaproteobacteria	Xanthomonadales	Xanthomonadaceae	*Rhodanobacter*	*thiooxydans*	14	0.004
Firmicutes	Clostridia	Clostridiales	Peptococcaceae	*Sporotomaculum*	*syntrophicum*	13	0.004
Proteobacteria	Gammaproteobacteria	Alteromonadales	Alteromonadaceae	*Candidatus Endobugula*		13	0.004
Bacteroidetes	Flavobacteriia	Flavobacteriales	Flavobacteriaceae	*Wautersiella*	*falsenii*	13	0.004
Actinobacteria	Actinobacteria	Actinomycetales	Microbacteriaceae	*Agromyces*	*terreus*	13	0.004
Proteobacteria	Gammaproteobacteria	Pseudomonadales	Pseudomonadaceae	*Pseudomonas*		13	0.004
Bacteroidetes	Flavobacteriia	Flavobacteriales	Flavobacteriaceae	*Chryseobacterium*	*soli*	13	0.004
Firmicutes	Clostridia	Clostridiales	Clostridiaceae	*Peptoniphilus*	*olsenii*	13	0.004
Acidobacteria	Acidobacteria	Acidobacteriales	Acidobacteriaceae	*Edaphobacter*	*modestus*	13	0.004
Proteobacteria	Deltaproteobacteria	Syntrophobacterales	Syntrophaceae	*Desulfomonile*	*tiedjei*	13	0.004
Bacteroidetes	Sphingobacteriia	Sphingobacteriales	Sphingobacteriaceae	*Sphingobacterium*	*multivorum*	12	0.003
Proteobacteria	Alphaproteobacteria	Rhizobiales	Methylocystaceae	*Methylosinus*		12	0.003

Cyanobacteria	Nostocophycideae	Nostocales	Nostocaceae	*Nostoc*	*piscinale*	12	0.003
Actinobacteria	Actinobacteria	Actinomycetales	Cellulomonadaceae	*Oerskovia*		12	0.003
Bacteroidetes	Sphingobacteriia	Sphingobacteriales	Flexibacteraceae	*Spirosoma*		12	0.003
Proteobacteria	Betaproteobacteria	Rhodocyclales	Rhodocyclaceae	*Dechloromonas*	*hortensis*	12	0.003
Proteobacteria	Betaproteobacteria	Burkholderiales	Alcaligenaceae	*Sutterella*	*sanguinus*	12	0.003
Proteobacteria	Gammaproteobacteria	Chromatiales	Ectothiorhodospiraceae	*Methylonatrum*	*kenyense*	12	0.003
Proteobacteria	Alphaproteobacteria	Rhodospirillales	Acetobacteraceae	*Acidiphilium*	*organovorum*	12	0.003
Actinobacteria	Actinobacteria	Actinomycetales	Streptomycetaceae	*Streptomyces*	*danangensis*	12	0.003
Proteobacteria	Deltaproteobacteria	Myxococcales	Haliangiaceae	*Haliangium*		12	0.003
Firmicutes	Clostridia	Clostridiales	Sulfobacillaceae	*Sulfobacillus*	*yellowstonensis*	12	0.003
Actinobacteria	Thermoleophilia	Solirubrobacterales	Solirubrobacteraceae	*Solirubrobacter*	*soli*	12	0.003
Firmicutes	Bacilli	Bacillales	Paenibacillaceae	*Paenibacillus*	*contaminans*	12	0.003
Proteobacteria	Alphaproteobacteria	Rhodospirillales	Acetobacteraceae	*Roseomonas*		12	0.003
Actinobacteria	Actinobacteria	Actinomycetales	Sporichthyaceae	*Sporichthya*		12	0.003
Firmicutes	Clostridia	Clostridiales	Peptococcaceae			12	0.003
Firmicutes	Bacilli	Bacillales	Bacillaceae	*Bacillus*	*litoralis*	11	0.003
Actinobacteria	Actinobacteria	Actinomycetales	Nocardiaceae	*Rhodococcus*	*marinonascens*	11	0.003
Actinobacteria	Actinobacteria	Actinomycetales	Micrococcaceae	*Arthrobacter*		11	0.003
Proteobacteria	Gammaproteobacteria	Enterobacteriales	Enterobacteriaceae	*Candidatus Blochmannia*	*castaneus*	11	0.003
Proteobacteria	Betaproteobacteria	Burkholderiales	Oxalobacteraceae	*Ralstonia*	*insidiosa*	11	0.003
Proteobacteria	Alphaproteobacteria	Rhodospirillales	Acetobacteraceae	*Gluconobacter*	*morbifer*	11	0.003
Actinobacteria	Actinobacteria	Actinomycetales	Actinopolysporaceae	*Actinopolyspora*	*indiensis*	11	0.003
Cyanobacteria	Oscillatoriophycideae	Chroococcales	Phormidiaceae	*Trichodesmium*	*tenue*	11	0.003
Firmicutes	Bacilli	Bacillales	Bacillaceae	*Bacillus*	*longiquaesitum*	11	0.003
Actinobacteria	Actinobacteria	Actinomycetales	Kineosporiaceae	*Kineococcus*	*gynurae*	11	0.003
Actinobacteria	Actinobacteria	Actinomycetales	Tsukamurellaceae	*Tsukamurella*		11	0.003
Firmicutes	Bacilli	Bacillales	Bacillaceae	*Lentibacillus*	*salinarum*	11	0.003
Actinobacteria	Acidimicrobiia	Acidimicrobiales	Acidimicrobiaceae	*Ferrimicrobium*	*acidiphilum*	11	0.003
Actinobacteria	Actinobacteria	Actinomycetales	Micromonosporaceae	*Micromonospora*	*rifamycinica*	11	0.003
Proteobacteria	Betaproteobacteria	Neisseriales	Neisseriaceae	*Chromobacterium*		11	0.003
Actinobacteria	Actinobacteria	Actinomycetales	Pseudonocardiaceae	*Jiangella*	*ginsengisoli*	11	0.003
Bacteroidetes	Flavobacteriia	Flavobacteriales	Flavobacteriaceae	*Flavobacterium*	*aquatile*	11	0.003
Actinobacteria	Actinobacteria	Actinomycetales	Streptomycetaceae	*Streptomyces*	*nanchangensis*	11	0.003
Chloroflexi	Anaerolineae	Anaerolineales	Anaerolinaceae	*Anaerolinea*		10	0.003
Proteobacteria	Alphaproteobacteria	Sphingomonadales				10	0.003
Firmicutes	Clostridia	Clostridiales	Clostridiaceae	*Caloramator*		10	0.003
Proteobacteria	Gammaproteobacteria	Xanthomonadales	Xanthomonadaceae	*Lysobacter*		10	0.003

Proteobacteria	Betaproteobacteria	Burkholderiales	Burkholderiaceae	*Burkholderia*	*phenoliruptrix*	10	0.003
Firmicutes	Clostridia	Coriobacteriales	Coriobacteriaceae	*Slackia*		10	0.003
Actinobacteria	Actinobacteria	Actinomycetales	Mycobacteriaceae	*Mycobacterium*	*lepromatosis*	10	0.003
Actinobacteria	Actinobacteria	Actinomycetales	Microbacteriaceae	*Microbacterium*	*halophilum*	10	0.003
Actinobacteria	Actinobacteria	Actinomycetales	Nocardiaceae	*Rhodococcus*	*kroppenstedtii*	10	0.003
Proteobacteria	Alphaproteobacteria	Sphingomonadales	Sphingomonadaceae	*Novosphingobium*	*subterraneum*	10	0.003
Firmicutes	Clostridia	Thermoanaerobacterales	Thermoanaerobacteraceae	*Ammonifex*	*thiophilus*	10	0.003
Bacteroidetes	Sphingobacteriia	Sphingobacteriales	Sphingobacteriaceae	*Pedobacter*	*koreensis*	10	0.003
Proteobacteria	Gammaproteobacteria	Thiotrichales	Piscirickettsiaceae	*Methylophaga*		10	0.003
Actinobacteria	Actinobacteria	Actinomycetales	Intrasporangiaceae	*Janibacter*	*corallicola*	10	0.003
Proteobacteria	Gammaproteobacteria	Xanthomonadales	Xanthomonadaceae	*Dokdonella*	*ginsengisoli*	10	0.003
Proteobacteria	Betaproteobacteria	Burkholderiales	Comamonadaceae	*Acidovorax*		9	0.003
Firmicutes	Clostridia	Halanaerobiales	Halanaerobiaceae	*Halanaerobium*		9	0.003
Actinobacteria	Actinobacteria	Actinomycetales	Microbacteriaceae	*Microbacterium*	*koreense*	9	0.003
Thermi	Deinococci	Thermales	Thermaceae	*Thermus*		9	0.003
Proteobacteria	Betaproteobacteria	Burkholderiales	Comamonadaceae	*Giesbergeria*		9	0.003
Proteobacteria	Deltaproteobacteria	Syntrophobacterales	Desulfobacteraceae			9	0.003
Proteobacteria	Epsilonproteobacteria	Campylobacterales	Helicobacteraceae	*Sulfurimonas*	*denitrificans*	9	0.003
Proteobacteria	Alphaproteobacteria	Rhodobacterales	Rhodobacteraceae	*Rubellimicrobium*	*roseum*	9	0.003
Proteobacteria	Alphaproteobacteria	Rhizobiales	Rhizobiaceae			9	0.003
Proteobacteria	Betaproteobacteria	Rhodocyclales	Rhodocyclaceae	*Azoarcus*		9	0.003
Firmicutes	Clostridia	Clostridiales	Peptococcaceae	*Desulfosporosinus*		9	0.003
Firmicutes	Bacilli	Bacillales	Bacillaceae	*Bacillus*	*arbutinivorans*	9	0.003
Proteobacteria	Alphaproteobacteria	Rhizobiales	Bartonellaceae	*Bartonella*	*rochalimae*	9	0.003
Firmicutes	Clostridia	Clostridiales	Peptococcaceae	*Pelotomaculum*	*isophthalicicum*	9	0.003
Bacteroidetes	Flavobacteriia	Flavobacteriales	Flavobacteriaceae	*Flavobacterium*	*fryxellicola*	9	0.003
Actinobacteria	Actinobacteria	Bifidobacteriales	Bifidobacteriaceae	*Bifidobacterium*	*indicum*	9	0.003
Firmicutes	Clostridia	Clostridiales	Clostridiaceae	*Peptoniphilus*	*gorbachii*	8	0.002
Proteobacteria	Alphaproteobacteria	Sphingomonadales	Sphingomonadaceae	*Sphingomonas*	*haloaromaticamans*	8	0.002
Tenericutes	Mollicutes					8	0.002
Actinobacteria	Actinobacteria	Actinomycetales	Pseudonocardiaceae	*Pseudonocardia*	*aurantiaca*	8	0.002
Cyanobacteria	Nostocophycideae	Nostocales	Nostocaceae	*Gloeotrichia*		8	0.002
Cyanobacteria	Nostocophycideae	Nostocales	Nostocaceae	*Nodularia*	*baltica*	8	0.002
Proteobacteria	Alphaproteobacteria	Sphingomonadales	Sphingomonadaceae	*Sphingobium*	*faniae*	8	0.002
Firmicutes	Bacilli	Lactobacillales	Streptococcaceae	*Streptococcus*	*tigurinus*	8	0.002
Firmicutes	Bacilli	Bacillales	Paenibacillaceae	*Brevibacillus*	*ginsengisoli*	8	0.002
Proteobacteria	Gammaproteobacteria	Pseudomonadales	Moraxellaceae	*Acinetobacter*	*johnsonii*	8	0.002

Firmicutes	Bacilli	Bacillales	Planococcaceae	*Sporosarcina*	*pasteurii*	8	0.002
Proteobacteria	Betaproteobacteria	Burkholderiales	Burkholderiaceae	*Candidatus Glomeribacter*		8	0.002
Actinobacteria	Actinobacteria	Actinomycetales	Geodermatophilaceae	*Geodermatophilus*		8	0.002
Proteobacteria	Alphaproteobacteria	Sphingomonadales	Sphingomonadaceae	*Novosphingobium*	*hassiacum*	8	0.002
Firmicutes	Clostridia	Clostridiales	Clostridiaceae	*Clostridium*	*bowmanii*	8	0.002
Firmicutes	Clostridia	Thermoanaerobacterales	Thermoanaerobacteraceae	*Caldanaerobacter*	*hydrothermalis*	8	0.002
Proteobacteria	Betaproteobacteria	Burkholderiales	Comamonadaceae	*Tepidimonas*		8	0.002
Proteobacteria	Gammaproteobacteria	Xanthomonadales	Xanthomonadaceae	*Stenotrophomonas*	*pavanii*	8	0.002
Proteobacteria	Gammaproteobacteria	Xanthomonadales	Xanthomonadaceae	*Stenotrophomonas*	*retroflexus*	7	0.002
Proteobacteria	Alphaproteobacteria	Rhizobiales	Xanthobacteraceae	*Ancylobacter*		7	0.002
Proteobacteria	Gammaproteobacteria	Chromatiales	Halothiobacillaceae	*Thiovirga*	*sulfuroxydans*	7	0.002
Firmicutes	Clostridia	Clostridiales	Veillonellaceae			7	0.002
Firmicutes	Clostridia	Clostridiales	Clostridiaceae	*Clostridium*	*cadaveris*	7	0.002
Actinobacteria	Actinobacteria	Actinomycetales	Kineosporiaceae			7	0.002
Proteobacteria	Alphaproteobacteria	Caulobacterales	Caulobacteraceae	*Mycoplana*		7	0.002
Proteobacteria	Betaproteobacteria	Burkholderiales	Comamonadaceae	*Thiomonas*		7	0.002
Actinobacteria	Actinobacteria	Actinomycetales	Streptosporangiaceae	*Nonomuraea*		7	0.002
Actinobacteria	Actinobacteria	Actinomycetales	Microbacteriaceae	*Microbacterium*	*xinjiangensis*	7	0.002
Proteobacteria	Betaproteobacteria	Burkholderiales	Burkholderiaceae	*Burkholderia*	*seminalis*	7	0.002
Proteobacteria	Gammaproteobacteria	Xanthomonadales	Xanthomonadaceae	*Lysobacter*	*deserti*	7	0.002
Proteobacteria	Alphaproteobacteria	Rhodospirillales	Acetobacteraceae	*Roseomonas*	*terpenica*	7	0.002
Actinobacteria	Actinobacteria	Actinomycetales	Microbacteriaceae	*Mycetocola*	*lacteus*	7	0.002
Firmicutes	Clostridia	Clostridiales	Clostridiaceae	*Clostridium*	*alkalicellulosi*	7	0.002
Actinobacteria	Actinobacteria	Actinomycetales	Micromonosporaceae	*Actinocatenispora*		7	0.002
Actinobacteria	Actinobacteria	Actinomycetales	Intrasporangiaceae	*Arsenicicoccus*	*bolidensis*	7	0.002
Proteobacteria	Alphaproteobacteria	Sphingomonadales	Sphingomonadaceae	*Sphingomonas*	*roseiflava*	7	0.002
Firmicutes	Bacilli	Lactobacillales	Streptococcaceae	*Streptococcus*		7	0.002
Bacteroidetes	Bacteroidia	Bacteroidales				7	0.002
Actinobacteria	Actinobacteria	Actinomycetales	Propionibacteriaceae	*Propionibacterium*		7	0.002
Proteobacteria	Alphaproteobacteria	Rhodobacterales	Rhodobacteraceae			7	0.002
Proteobacteria	Alphaproteobacteria	Caulobacterales	Caulobacteraceae	*Caulobacter*	*crescentus*	7	0.002
Actinobacteria	Actinobacteria	Actinomycetales	Actinopolysporaceae	*Actinopolyspora*	*salina*	7	0.002
Proteobacteria	Alphaproteobacteria	Rickettsiales	Anaplasmataceae	*Ehrlichia*	*ovina*	7	0.002
Actinobacteria	Actinobacteria	Actinomycetales	Cellulomonadaceae	*Demequina*	*globuliformis*	7	0.002
Firmicutes	Clostridia	Clostridiales	Ruminococcaceae	*Oscillospira*	*eae*	7	0.002
Actinobacteria	Actinobacteria	Actinomycetales	Streptosporangiaceae	*Streptosporangium*	*subroseum*	7	0.002
Actinobacteria	Actinobacteria	Actinomycetales	Microbacteriaceae	*Agrococcus*		6	0.002

Proteobacteria	Gammaproteobacteria	Alteromonadales	Chromatiaceae	*Alishewanella*		6	0.002
Actinobacteria	Actinobacteria	Actinomycetales	Dermabacteraceae	*Brachybacterium*	*squillarum*	6	0.002
Proteobacteria	Betaproteobacteria	Burkholderiales	Comamonadaceae	*Limnobacter*	*litoralis*	6	0.002
Actinobacteria	Actinobacteria	Actinomycetales	Cryptosporangiaceae	*Cryptosporangium*	*arvum*	6	0.002
Proteobacteria	Gammaproteobacteria	Xanthomonadales	Xanthomonadaceae	*Thermomonas*	*dokdonensis*	6	0.002
Firmicutes	Clostridia	Clostridiales	Symbiobacteriaceae	*Symbiobacterium*	*toebii*	6	0.002
Bacteroidetes	Flavobacteriia	Flavobacteriales	Flavobacteriaceae	*Gramella*		6	0.002
Proteobacteria	Gammaproteobacteria	Xanthomonadales	Xanthomonadaceae	*Lysobacter*	*daejeonensis*	6	0.002
Proteobacteria	Deltaproteobacteria	Myxococcales	Cystobacteraceae	*Cystobacter*		6	0.002
Firmicutes	Clostridia	Coriobacteriales	Coriobacteriaceae	*Atopobium*	*fossor*	6	0.002
Proteobacteria	Gammaproteobacteria	Xanthomonadales	Xanthomonadaceae	*Rhodanobacter*		6	0.002
Actinobacteria	Actinobacteria	Actinomycetales	Cellulomonadaceae	*Cellulomonas*	*chitinilytica*	6	0.002
Proteobacteria	Epsilonproteobacteria	Campylobacterales	Campylobacteraceae	*Campylobacter*		6	0.002
Proteobacteria	Betaproteobacteria	Burkholderiales	Comamonadaceae	*Acidovorax*	*valerianellae*	6	0.002
Actinobacteria	Actinobacteria	Actinomycetales	Streptomycetaceae	*Streptomyces*	*roseogilvus*	6	0.002
Proteobacteria	Alphaproteobacteria	Kiloniellales	Kiloniellaceae	*Thalassospira*		6	0.002
Proteobacteria	Betaproteobacteria	Hydrogenophilales	Hydrogenophilaceae	*Thiobacillus*		6	0.002
Firmicutes	Clostridia	Clostridiales	Veillonellaceae	*Phascolarctobacterium*	*succinatutens*	6	0.002
Firmicutes	Clostridia	Thermoanaerobacterales	Caldicellulosiruptoraceae	*Caldicellulosiruptor*		6	0.002
Proteobacteria	Alphaproteobacteria	Rhodospirillales	Acetobacteraceae	*Roseomonas*	*aquatica*	6	0.002
Proteobacteria	Betaproteobacteria	Burkholderiales	Comamonadaceae	*Azohydromonas*	*australica*	6	0.002
Proteobacteria	Betaproteobacteria	Burkholderiales	Burkholderiaceae			6	0.002
Actinobacteria	Actinobacteria	Actinomycetales	Gordoniaceae	*Gordonia*	*australis*	6	0.002
Actinobacteria	Actinobacteria	Actinomycetales	Nocardioidaceae			6	0.002
Actinobacteria	Actinobacteria	Actinomycetales	Actinosynnemataceae	*Actinokineospora*	*riparia*	6	0.002
Proteobacteria	Gammaproteobacteria	Oceanospirillales	Oceanospirillaceae	*Marinomonas*		5	0.001
Proteobacteria	Gammaproteobacteria	Chromatiales	Ectothiorhodospiraceae			5	0.001
Thermotogae	Thermotogae	Thermotogales	Thermotogaceae	*Petrotoga*		5	0.001
Proteobacteria	Betaproteobacteria	Hydrogenophilales	Hydrogenophilaceae	*Thiobacillus*	*thiophilus*	5	0.001
Bacteroidetes	Flavobacteriia	Flavobacteriales	Flavobacteriaceae	*Flavobacterium*	*weaverense*	5	0.001
Proteobacteria	Gammaproteobacteria	Pseudomonadales	Moraxellaceae	*Acinetobacter*	*indicus*	5	0.001
Actinobacteria	Actinobacteria	Actinomycetales	Actinomycetaceae			5	0.001
Proteobacteria	Betaproteobacteria	Burkholderiales	Burkholderiaceae	*Burkholderia*	*bryophila*	5	0.001
Proteobacteria	Gammaproteobacteria	Enterobacteriales	Enterobacteriaceae	*Serratia*	*entomophila*	5	0.001
Proteobacteria	Alphaproteobacteria	Rhizobiales	Xanthobacteraceae	*Xanthobacter*	*polyaromaticivorans*	5	0.001
Proteobacteria	Gammaproteobacteria	Pasteurellales	Pasteurellaceae			5	0.001
Firmicutes	Clostridia	Clostridiales	Clostridiaceae	*Clostridium*	*thermosuccinogenes*	5	0.001

Actinobacteria	Actinobacteria	Actinomycetales	Pseudonocardiaceae	*Saccharopolyspora*	*shandongensis*	5	0.001
Actinobacteria	Actinobacteria	Actinomycetales	Pseudonocardiaceae	*Pseudonocardia*	*khuvsgulensis*	5	0.001
Proteobacteria	Betaproteobacteria	Rhodocyclales	Rhodocyclaceae	*Hydrogenophilus*	*denitrificans*	5	0.001
Firmicutes	Bacilli	Bacillales	Planococcaceae	*Sporosarcina*	*luteola*	5	0.001
Firmicutes	Clostridia	Clostridiales	Ruminococcaceae	*Oscillospira*		5	0.001
Proteobacteria	Alphaproteobacteria	Rhizobiales	Methylobacteriaceae	*Methylobacterium*	*goesingense*	5	0.001
Bacteroidetes	Sphingobacteriia	Sphingobacteriales	Chitinophagaceae	*Niastella*		5	0.001
Proteobacteria	Gammaproteobacteria	Oceanospirillales	Halomonadaceae	*Kushneria*		5	0.001
Proteobacteria	Betaproteobacteria	Rhodocyclales	Rhodocyclaceae	*Uliginosibacterium*	*gangwonense*	5	0.001
Proteobacteria	Alphaproteobacteria	Rhizobiales	Phyllobacteriaceae	*Phyllobacterium*	*catacumbae*	5	0.001
Proteobacteria	Gammaproteobacteria	Oceanospirillales	Halomonadaceae			5	0.001
Proteobacteria	Alphaproteobacteria	Sphingomonadales	Sphingomonadaceae	*Novosphingobium*	*aromaticivorans*	5	0.001
Proteobacteria	Betaproteobacteria	Burkholderiales	Comamonadaceae	*Rubrivivax*	*benzoatilyticus*	5	0.001
Proteobacteria	Alphaproteobacteria	Sphingomonadales	Sphingomonadaceae	*Sphingomonas*	*hunanensis*	5	0.001
Proteobacteria	Gammaproteobacteria	Oceanospirillales	Litoricolaceae	*Litoricola*		5	0.001
Proteobacteria	Gammaproteobacteria	Xanthomonadales	Xanthomonadaceae	*Xanthomonas*		5	0.001
Actinobacteria	Actinobacteria	Actinomycetales	Glycomycetaceae	*Glycomyces*	*tenuis*	5	0.001
Proteobacteria	Betaproteobacteria	Methylophilales	Methylophilaceae			5	0.001
Actinobacteria	Actinobacteria	Actinomycetales	Micrococcaceae	*Rothia*		5	0.001
Actinobacteria	Actinobacteria	Actinomycetales	Actinosynnemataceae	*Actinoalloteichus*	*nanshanensis*	4	0.001
Proteobacteria	Epsilonproteobacteria	Campylobacterales	Campylobacteraceae			4	0.001
Proteobacteria	Betaproteobacteria	Burkholderiales	Alcaligenaceae	*Achromobacter*		4	0.001
Firmicutes	Clostridia	Clostridiales	Lachnospiraceae	*Lachnospira*	*pectinoschiza*	4	0.001
Proteobacteria	Alphaproteobacteria	Rhodobacterales	Rhodobacteraceae	*Anaerospora*	*hongkongensis*	4	0.001
Proteobacteria	Deltaproteobacteria	Desulfovibrionales	Desulfohalobiaceae	*Desulfonatronovibrio*		4	0.001
Bacteroidetes	Sphingobacteriia	Sphingobacteriales	Sphingobacteriaceae	*Sphingobacterium*	*siyangense*	4	0.001
Proteobacteria	Gammaproteobacteria	Pasteurellales	Pasteurellaceae	*Mannheimia*	*caviae*	4	0.001
Firmicutes	Clostridia	Clostridiales	Clostridiaceae	*Clostridium*	*tepidiprofundi*	4	0.001
Firmicutes	Clostridia	Natranaerobiales	Contubernalisaceae	*Candidatus Contubernalis*	*alkalaceticum*	4	0.001
Actinobacteria	Actinobacteria	Actinomycetales	Geodermatophilaceae	*Geodermatophilus*	*obscurus*	4	0.001
Proteobacteria	Alphaproteobacteria	Rhizobiales	Xanthobacteraceae	*Blastochloris*	*gulmargensis*	4	0.001
Proteobacteria	Alphaproteobacteria	Rhodospirillales	Acetobacteraceae	*Acidocella*	*aluminiidurans*	4	0.001
Bacteroidetes	Sphingobacteriia	Sphingobacteriales	Sphingobacteriaceae	*Pedobacter*	*panaciterrae*	4	0.001
Firmicutes	Clostridia	Clostridiales	Clostridiaceae	*Peptoniphilus*	*methioninivorax*	4	0.001
Proteobacteria	Gammaproteobacteria	Pseudomonadales	Moraxellaceae	*Acinetobacter*	*lwoffii*	4	0.001
Firmicutes	Bacilli	Lactobacillales	Leuconostocaceae	*Oenococcus*		4	0.001
Proteobacteria	Alphaproteobacteria	Rhodospirillales	Rhodospirillaceae	*Magnetospirillum*	*bellicus*	4	0.001

Proteobacteria	Alphaproteobacteria	Rhizobiales	Bradyrhizobiaceae	*Bradyrhizobium*	*pachyrhizi*	4	0.001
Proteobacteria	Alphaproteobacteria	Rhizobiales	Rhizobiaceae	*Agrobacterium*	*viscosum*	4	0.001
Actinobacteria	Actinobacteria	Actinomycetales	Mycobacteriaceae	*Mycobacterium*	*simulans*	4	0.001
Firmicutes	Bacilli	Bacillales	Staphylococcaceae	*Staphylococcus*	*equorum*	4	0.001
Proteobacteria	Alphaproteobacteria	Rhodobacterales	Rhodobacteraceae	*Rhodobacter*	*gluconicum*	4	0.001
Proteobacteria	Alphaproteobacteria	Rhodospirillales	Rhodospirillaceae	*Oleomonas*	*sagaranensis*	4	0.001
Proteobacteria	Deltaproteobacteria	Desulfovibrionales				4	0.001
Proteobacteria	Betaproteobacteria	Burkholderiales	Oxalobacteraceae	*Ralstonia*	*pickettii*	4	0.001
Actinobacteria	Actinobacteria	Actinomycetales	Dermabacteraceae	*Brachybacterium*		4	0.001
Proteobacteria	Gammaproteobacteria	Pseudomonadales	Pseudomonadaceae	*Pseudomonas*	*entomophila*	4	0.001
Firmicutes	Bacilli	Bacillales	Planococcaceae	*Lysinibacillus*	*xylanilyticus*	4	0.001
Proteobacteria	Betaproteobacteria	Neisseriales	Neisseriaceae	*Chromobacterium*	*haemolyticum*	4	0.001
Proteobacteria	Gammaproteobacteria	Xanthomonadales	Xanthomonadaceae	*Rhodanobacter*	*lindaniclasticus*	4	0.001
Actinobacteria	Actinobacteria	Actinomycetales	Micrococcaceae	*Zhihengliuella*	*salsuginis*	4	0.001
Firmicutes	Clostridia	Clostridiales	Clostridiaceae	*Thermoanaerobacterium*	*islandicum*	4	0.001
Actinobacteria	Actinobacteria	Actinomycetales	Nocardiaceae	*Nocardia*	*polyresistens*	4	0.001
Actinobacteria	Actinobacteria	Actinomycetales	Micromonosporaceae	*Actinocatenispora*	*thailandica*	4	0.001
Proteobacteria	Betaproteobacteria	Burkholderiales	Comamonadaceae	*Tepidimonas*	*ignava*	4	0.001
Bacteroidetes	Sphingobacteriia	Sphingobacteriales	Saprospiraceae	*Lewinella*	*marina*	4	0.001
Proteobacteria	Gammaproteobacteria	Thiotrichales	Thiotrichaceae	*Leucothrix*	*mucor*	4	0.001
Bacteroidetes	Flavobacteriia	Flavobacteriales	Flavobacteriaceae	*Flavobacterium*	*terrigena*	4	0.001
Proteobacteria	Alphaproteobacteria	Rhodobacterales	Rhodobacteraceae	*Rhodobacter*	*apigmentum*	4	0.001
Bacteroidetes	Flavobacteriia	Flavobacteriales	Flavobacteriaceae	*Flavobacterium*	*omnivorum*	4	0.001
Proteobacteria	Betaproteobacteria	Neisseriales	Neisseriaceae	*Chromobacterium*	*piscinae*	4	0.001
Proteobacteria	Gammaproteobacteria	Oceanospirillales	Oceanospirillaceae			4	0.001
Actinobacteria	Actinobacteria	Actinomycetales	Micrococcaceae	*Arthrobacter*	*soli*	4	0.001
Firmicutes	Bacilli	Lactobacillales	Carnobacteriaceae	*Carnobacterium*	*inhibens*	4	0.001
Proteobacteria	Alphaproteobacteria	Caulobacterales	Caulobacteraceae	*Brevundimonas*	*olei*	4	0.001
Proteobacteria	Alphaproteobacteria	Rhizobiales	Beijerinckiaceae	*Beijerinckia*	*derxii*	4	0.001
Actinobacteria	Actinobacteria	Actinomycetales	Actinosynnemataceae	*Actinoalloteichus*	*alkalophilus*	4	0.001
Proteobacteria	Alphaproteobacteria	Sphingomonadales	Sphingomonadaceae	*Novosphingobium*	*lentum*	4	0.001
Proteobacteria	Gammaproteobacteria	Thiotrichales	Piscirickettsiaceae	*Thiomicrospira*	*chilensis*	4	0.001
Proteobacteria	Deltaproteobacteria	Bdellovibrionales	Bdellovibrionaceae	*Bdellovibrio*		4	0.001
Actinobacteria	Actinobacteria	Actinomycetales	Nocardioidaceae	*Actinopolymorpha*	*rutila*	4	0.001
Actinobacteria	Actinobacteria	Actinomycetales	Pseudonocardiaceae	*Saccharopolyspora*	*flava*	4	0.001
Actinobacteria	Actinobacteria	Actinomycetales	Nocardioidaceae	*Friedmanniella*		4	0.001
Proteobacteria	Alphaproteobacteria	Sphingomonadales	Sphingomonadaceae	*Sphingopyxis*		3	0.001

Proteobacteria	Gammaproteobacteria	Xanthomonadales	Xanthomonadaceae	*Xanthomonas*	*oryzae*	3	0.001
Proteobacteria	Alphaproteobacteria	Rhizobiales	Methylocystaceae	*Pleomorphomonas*		3	0.001
Proteobacteria	Gammaproteobacteria	Chromatiales	Ectothiorhodospiraceae	*Halorhodospira*	*halochloris*	3	0.001
Proteobacteria	Gammaproteobacteria	Oceanospirillales	Halomonadaceae	*Halomonas*		3	0.001
Proteobacteria	Gammaproteobacteria	Chromatiales	Ectothiorhodospiraceae	*Thiorhodospira*		3	0.001
Acidobacteria	Acidobacteria	Acidobacteriales	Acidobacteriaceae	*Granulicella*	*tundricola*	3	0.001
Proteobacteria	Deltaproteobacteria	Syntrophobacterales	Syntrophobacteraceae			3	0.001
Actinobacteria	Actinobacteria	Actinomycetales	Pseudonocardiaceae	*Amycolatopsis*	*xylanica*	3	0.001
Firmicutes	Clostridia	Clostridiales	Clostridiaceae	*Peptoniphilus*	*coxii*	3	0.001
Bacteroidetes	Sphingobacteriia	Sphingobacteriales	Sphingobacteriaceae	*Sphingobacterium*	*bambusae*	3	0.001
Firmicutes	Bacilli	Bacillales	Bacillaceae	*Bacillus*	*kribbensis*	3	0.001
Proteobacteria	Betaproteobacteria	Neisseriales	Neisseriaceae	*Neisseria*	*mucosa*	3	0.001
Firmicutes	Clostridia	Clostridiales	Veillonellaceae	*Pectinatus*	*cerevisiiphilus*	3	0.001
Actinobacteria	Actinobacteria	Actinomycetales	Microbacteriaceae	*Microbacterium*	*profundi*	3	0.001
Proteobacteria	Alphaproteobacteria	Rhizobiales	Rhizobiaceae	*Candidatus Liberibacter*	*solanacearum*	3	0.001
Proteobacteria	Gammaproteobacteria	Oceanospirillales	Oceanospirillaceae	*Marinomonas*	*basaltis*	3	0.001
Firmicutes	Clostridia	Clostridiales	Veillonellaceae	*Propionispora*	*hippei*	3	0.001
Proteobacteria	Gammaproteobacteria	Oceanospirillales				3	0.001
Firmicutes	Clostridia	Thermoanaerobacterales	Thermoanaerobacteraceae	*Thermoanaerobacter*	*inferii*	3	0.001
Proteobacteria	Betaproteobacteria	Burkholderiales	Burkholderiaceae	*Burkholderia*	*lata*	3	0.001
Proteobacteria	Deltaproteobacteria	Syntrophobacterales	Desulfobacteraceae	*Desulfobacter*		3	0.001
Bacteroidetes	Flavobacteriia	Flavobacteriales	Flavobacteriaceae	*Tenacibaculum*		3	0.001
Proteobacteria	Epsilonproteobacteria	Campylobacterales				3	0.001
Actinobacteria	Actinobacteria	Actinomycetales	Corynebacteriaceae	*Corynebacterium*	*argentoratense*	3	0.001
Actinobacteria	Actinobacteria	Actinomycetales	Microbacteriaceae	*Agrococcus*	*citreus*	3	0.001
Proteobacteria	Gammaproteobacteria	Aeromonadales	Aeromonadaceae	*Tolumonas*	*auensis*	3	0.001
Proteobacteria	Deltaproteobacteria	Syntrophobacterales	Desulfobacteraceae	*Desulfofrigus*	*oceanense*	3	0.001
Proteobacteria	Gammaproteobacteria	Xanthomonadales	Sinobacteraceae	*Hydrocarboniphaga*	*daqingensis*	3	0.001
Actinobacteria	Actinobacteria	Actinomycetales	Propionibacteriaceae	*Propionibacterium*	*microaerophilum*	3	0.001
Actinobacteria	Actinobacteria	Actinomycetales	Micrococcaceae	*Arthrobacter*	*psychrochitiniphilus*	3	0.001
Bacteroidetes	Flavobacteriia	Flavobacteriales	Flavobacteriaceae	*Flavobacterium*	*algicola*	3	0.001
Actinobacteria	Actinobacteria	Actinomycetales	Pseudonocardiaceae	*Amycolatopsis*	*jejuensis*	3	0.001
Proteobacteria	Alphaproteobacteria	Rhizobiales	Phyllobacteriaceae	*Mesorhizobium*	*camelthorni*	3	0.001
Firmicutes	Bacilli	Lactobacillales	Aerococcaceae	*Alkalibacterium*	*subtropicum*	3	0.001
Actinobacteria	Actinobacteria	Actinomycetales	Micromonosporaceae	*Pilimelia*	*columellifera*	3	0.001
Actinobacteria	Actinobacteria	Actinomycetales	Intrasporangiaceae	*Knoellia*	*subterranea*	3	0.001
Actinobacteria	Actinobacteria	Actinomycetales	Actinosynnemataceae	*Saccharothrix*	*australiensis*	3	0.001

Proteobacteria	Gammaproteobacteria	Xanthomonadales	Xanthomonadaceae	*Lysobacter*	*niabensis*	3	0.001
Actinobacteria	Actinobacteria	Actinomycetales	Dietziaceae	*Dietzia*	*alimentaria*	3	0.001
Proteobacteria	Deltaproteobacteria	Syntrophobacterales				3	0.001
Cyanobacteria	Oscillatoriophycideae	Chroococcales	Phormidiaceae	*Trichodesmium*	*hildebrandtii*	3	0.001
Proteobacteria	Alphaproteobacteria	Rhodospirillales	Rhodospirillaceae	*Azospirillum*	*palatum*	3	0.001
Actinobacteria	Actinobacteria	Actinomycetales	Nocardiaceae	*Nocardia*	*roseoalba*	3	0.001
Actinobacteria	Actinobacteria	Actinomycetales	Nocardioidaceae	*Aeromicrobium*		3	0.001
Proteobacteria	Betaproteobacteria	Neisseriales	Neisseriaceae	*Deefgea*	*rivuli*	3	0.001
Proteobacteria	Gammaproteobacteria	Alteromonadales	Shewanellaceae	*Shewanella*	*profunda*	3	0.001
Proteobacteria	Betaproteobacteria	Burkholderiales	Burkholderiaceae	*Burkholderia*	*sartisoli*	3	0.001
Firmicutes	Clostridia	Clostridiales	Clostridiaceae	*Alkaliphilus*	*peptidifermentans*	3	0.001
Firmicutes	Clostridia	Clostridiales	Carboxydocellaceae	*Carboxydocella*	*ferrireducens*	3	0.001
Firmicutes	Clostridia	Clostridiales	Peptococcaceae	*Desulfurispora*	*thermophila*	3	0.001
Proteobacteria	Betaproteobacteria	Gallionellales	Gallionellaceae	*Gallionella*	*ferruginea*	3	0.001
Proteobacteria	Betaproteobacteria	Burkholderiales	Comamonadaceae	*Comamonas*	*kerstersii*	3	0.001
Acidobacteria	Acidobacteria	Acidobacteriales	Koribacteraceae	*Candidatus Koribacter*		3	0.001
Bacteroidetes	Sphingobacteriia	Sphingobacteriales	Saprospiraceae	*Lewinella*	*lutea*	3	0.001
Proteobacteria	Gammaproteobacteria	Pseudomonadales	Pseudomonadaceae	*Pseudomonas*	*azotoformans*	3	0.001
Proteobacteria	Gammaproteobacteria	Xanthomonadales	Xanthomonadaceae	*Luteimonas*		3	0.001
Firmicutes	Clostridia	Clostridiales	Peptococcaceae	*Desulfosporosinus*	*auripigmenti*	3	0.001
Proteobacteria	Betaproteobacteria	Burkholderiales	Oxalobacteraceae	*Ralstonia*		3	0.001
Actinobacteria	Actinobacteria	Actinomycetales	Corynebacteriaceae	*Corynebacterium*	*marinum*	3	0.001
Proteobacteria	Gammaproteobacteria	Enterobacteriales	Enterobacteriaceae	*Escherichia*	*albertii*	3	0.001
Proteobacteria	Alphaproteobacteria	Rhizobiales	Methylobacteriaceae	*Methylobacterium*	*longum*	3	0.001
Actinobacteria	Thermoleophilia	Solirubrobacterales	Solirubrobacteraceae	*Solirubrobacter*		3	0.001
Thermotogae	Thermotogae	Thermotogales	Thermotogaceae	*Fervidobacterium*		3	0.001
Bacteroidetes	Sphingobacteriia	Sphingobacteriales	Flexibacteraceae	*Spirosoma*	*linguale*	3	0.001
Proteobacteria	Gammaproteobacteria	Chromatiales	Halothiobacillaceae	*Thiovirga*		3	0.001
Proteobacteria	Gammaproteobacteria	Pseudomonadales	Moraxellaceae	*Acinetobacter*	*ursingii*	3	0.001
Firmicutes	Bacilli	Bacillales	Bacillaceae	*Bacillus*	*badius*	3	0.001
Proteobacteria	Epsilonproteobacteria	Campylobacterales	Helicobacteraceae	*Helicobacter*		3	0.001
Firmicutes	Clostridia	Clostridiales	Peptococcaceae	*Desulfosporosinus*	*hippei*	3	0.001
Proteobacteria	Gammaproteobacteria	Xanthomonadales	Xanthomonadaceae	*Stenotrophomonas*	*geniculata*	3	0.001
Firmicutes	Bacilli	Lactobacillales	Leuconostocaceae	*Fructobacillus*	*pseudoficulneus*	3	0.001
Firmicutes	Clostridia	Clostridiales	Clostridiaceae	*Peptoniphilus*		3	0.001
Bacteroidetes	Flavobacteriia	Flavobacteriales	Flavobacteriaceae	*Flavobacterium*	*antarcticum*	3	0.001
Actinobacteria	Actinobacteria	Actinomycetales	Streptosporangiaceae	*Sphaerisporangium*	*rubeum*	3	0.001

Actinobacteria	Actinobacteria	Actinomycetales	Yaniellaceae	*Yaniella*		3	0.001
Acidobacteria	Acidobacteria	Acidobacteriales	Acidobacteriaceae			3	0.001
Proteobacteria	Alphaproteobacteria	Caulobacterales	Caulobacteraceae	*Phenylobacterium*	*mobile*	3	0.001
Proteobacteria	Epsilonproteobacteria	Campylobacterales	Helicobacteraceae	*Sulfurimonas*	*paralvinellae*	3	0.001
Firmicutes	Clostridia	Clostridiales	Veillonellaceae	*Veillonella*		3	0.001
Proteobacteria	Betaproteobacteria	Burkholderiales	Comamonadaceae	*Curvibacter*		3	0.001
Proteobacteria	Alphaproteobacteria	Rhodobacterales	Rhodobacteraceae	*Rubellimicrobium*	*aerolatum*	3	0.001
Proteobacteria	Alphaproteobacteria	Rhodobacterales				3	0.001
Cyanobacteria	Oscillatoriophycideae	Chroococcales	Xenococcaceae	*Chroococcidiopsis*		3	0.001
Actinobacteria	Actinobacteria	Actinomycetales	Streptomycetaceae	*Streptomyces*	*yunnanensis*	2	0.001
Proteobacteria	Gammaproteobacteria	Chromatiales	Chromatiaceae	*Thiorhodococcus*	*pfennigii*	2	0.001
Proteobacteria	Gammaproteobacteria	Pseudomonadales	Pseudomonadaceae	*Pseudomonas*	*tremae*	2	0.001
Proteobacteria	Gammaproteobacteria	Chromatiales	Chromatiaceae	*Marichromatium*	*gracile*	2	0.001
Actinobacteria	Actinobacteria	Actinomycetales	Actinomycetaceae	*Actinomyces*	*lingnae*	2	0.001
Proteobacteria	Gammaproteobacteria	Oceanospirillales	Saccharospirillaceae	*Saccharospirillum*		2	0.001
Proteobacteria	Gammaproteobacteria	Thiotrichales	Piscirickettsiaceae	*Cycloclasticus*	*oligotrophus*	2	0.001
Proteobacteria	Alphaproteobacteria	Rhizobiales	Beijerinckiaceae	*Methylocella*		2	0.001
Proteobacteria	Gammaproteobacteria	Chromatiales	Halothiobacillaceae	*Thiofaba*		2	0.001
Bacteroidetes	Flavobacteriia	Flavobacteriales	Flavobacteriaceae	*Myroides*		2	0.001
Firmicutes	Clostridia	Clostridiales	Veillonellaceae	*Negativicoccus*	*succinicivorans*	2	0.001
Proteobacteria	Deltaproteobacteria	Desulfuromonadales	Desulfuromonadaceae	*Desulfuromonas*	*svalbardensis*	2	0.001
Proteobacteria	Gammaproteobacteria	Xanthomonadales	Xanthomonadaceae	*Luteibacter*	*yeojuensis*	2	0.001
Proteobacteria	Gammaproteobacteria	Alteromonadales	Pseudoalteromonadaceae	*Pseudoalteromonas*	*gracilis*	2	0.001
Firmicutes	Clostridia	Halanaerobiales	Halobacteroidaceae	*Halanaerobacter*	*chitinivorans*	2	0.001
Actinobacteria	Actinobacteria	Actinomycetales	Nocardiaceae	*Rhodococcus*	*rhodochrous*	2	0.001
Actinobacteria	Actinobacteria	Actinomycetales	Bogoriellaceae	*Georgenia*	*halophila*	2	0.001
Proteobacteria	Gammaproteobacteria	Chromatiales	Chromatiaceae	*Nitrosococcus*	*watsoni*	2	0.001
Thermotogae	Thermotogae	Thermotogales	Thermotogaceae	*Kosmotoga*	*arenicorallina*	2	0.001
Actinobacteria	Actinobacteria	Actinomycetales	Pseudonocardiaceae	*Saccharopolyspora*	*gloriosa*	2	0.001
Proteobacteria	Betaproteobacteria	Burkholderiales	Comamonadaceae	*Comamonas*	*odontotermitis*	2	0.001
Firmicutes	Bacilli	Bacillales	Bacillaceae	*Virgibacillus*	*byunsanensis*	2	0.001
Proteobacteria	Gammaproteobacteria	Enterobacteriales	Enterobacteriaceae	*Serratia*		2	0.001
Bacteroidetes	Sphingobacteriia	Sphingobacteriales	Chitinophagaceae	*Niabella*	*soli*	2	0.001
Proteobacteria	Gammaproteobacteria	Xanthomonadales	Xanthomonadaceae	*Luteimonas*	*mephitis*	2	0.001
Actinobacteria	Actinobacteria	Actinomycetales	Actinomycetaceae	*Arcanobacterium*	*bernardiae*	2	0.001
Firmicutes	Bacilli	Bacillales	Planococcaceae	*Lysinibacillus*	*parviboronicapiens*	2	0.001
Actinobacteria	Thermoleophilia	Solirubrobacterales				2	0.001

Thermotogae	Thermotogae	Thermotogales	Thermotogaceae	*Marinitoga*	*hydrogenitolerans*	2	0.001
Actinobacteria	Actinobacteria	Actinomycetales	Nocardiaceae	*Nocardia*	*pigrifrangens*	2	0.001
Proteobacteria	Gammaproteobacteria	Thiotrichales	Piscirickettsiaceae	*Thioalkalimicrobium*	*sibiricum*	2	0.001
Actinobacteria	Actinobacteria	Actinomycetales	Microbacteriaceae	*Agromyces*	*succinolyticus*	2	0.001
Proteobacteria	Betaproteobacteria	Neisseriales	Neisseriaceae	*Stenoxybacter*		2	0.001
Firmicutes	Bacilli	Bacillales	Staphylococcaceae	*Staphylococcus*	*haemolyticus*	2	0.001
Proteobacteria	Gammaproteobacteria	Vibrionales	Vibrionaceae	*Salinivibrio*	*budaii*	2	0.001
Actinobacteria	Actinobacteria	Actinomycetales	Microbacteriaceae	*Agromyces*	*fucosus*	2	0.001
Firmicutes	Clostridia	Clostridiales	Clostridiaceae	*Clostridium*	*caenicola*	2	0.001
Actinobacteria	Actinobacteria	Actinomycetales	Brevibacteriaceae	*Brevibacterium*	*album*	2	0.001
Actinobacteria	Actinobacteria	Actinomycetales	Dermacoccaceae	*Dermacoccus*	*abyssi*	2	0.001
Actinobacteria	Actinobacteria	Actinomycetales	Dermabacteraceae	*Brachybacterium*	*arcticum*	2	0.001
Firmicutes	Clostridia	Clostridiales	Sulfobacillaceae	*Sulfobacillus*	*thermotolerans*	2	0.001
Cyanobacteria	Nostocophycideae	Nostocales	Nostocaceae	*Anabaena*	*augstumalis*	2	0.001
Proteobacteria	Gammaproteobacteria	Pseudomonadales	Moraxellaceae			2	0.001
Proteobacteria	Alphaproteobacteria	Sphingomonadales	Sphingomonadaceae	*Sphingopyxis*	*granuli*	2	0.001
Bacteroidetes	Flavobacteriia	Flavobacteriales	Flavobacteriaceae	*Muricauda*	*lutimaris*	2	0.001
Actinobacteria	Actinobacteria	Actinomycetales	Pseudonocardiaceae	*Jiangella*		2	0.001
Proteobacteria	Epsilonproteobacteria	Campylobacterales	Campylobacteraceae	*Sulfurospirillum*	*arcachonense*	2	0.001
Bacteroidetes	Bacteroidia	Bacteroidales	Porphyromonadaceae	*Porphyromonas*	*canis*	2	0.001
Proteobacteria	Gammaproteobacteria	Oceanospirillales	Litoricolaceae	*Litoricola*	*lipolytica*	2	0.001
Actinobacteria	Actinobacteria	Actinomycetales	Micromonosporaceae	*Actinoplanes*		2	0.001
Bacteroidetes	Flavobacteriia	Flavobacteriales	Flavobacteriaceae	*Polaribacter*	*dokdonensis*	2	0.001
Firmicutes	Clostridia	Clostridiales	Lachnospiraceae	*Johnsonella*	*ignava*	2	0.001
Proteobacteria	Alphaproteobacteria	Rhizobiales	Beijerinckiaceae			2	0.001
Bacteroidetes	Sphingobacteriia	Sphingobacteriales	Sphingobacteriaceae	*Sphingobacterium*	*thalpophilum*	2	0.001
Actinobacteria	Actinobacteria	Actinomycetales	Nocardioidaceae	*Nocardioides*		2	0.001
Planctomycetes	Planctomycetia	Gemmatales				2	0.001
Proteobacteria	Gammaproteobacteria	Legionellales	Francisellaceae	*Francisella*	*hispaniensis*	2	0.001
Proteobacteria	Alphaproteobacteria	Rhodobacterales	Rhodobacteraceae	*Rubellimicrobium*		2	0.001
Proteobacteria	Alphaproteobacteria	Rhodospirillales	Rhodospirillaceae	*Rhodovibrio*	*sodomensis*	2	0.001
Actinobacteria	Actinobacteria	Actinomycetales	Micrococcaceae	*Kocuria*	*koreensis*	2	0.001
Actinobacteria	Actinobacteria	Bifidobacteriales	Bifidobacteriaceae	*Bifidobacterium*	*choerinum*	2	0.001
Firmicutes	Bacilli	Bacillales	Bacillaceae	*Bacillus*	*beringensis*	2	0.001
Proteobacteria	Deltaproteobacteria	Desulfuromonadales	Geobacteraceae	*Geobacter*	*pickeringii*	2	0.001
Actinobacteria	Actinobacteria	Actinomycetales	Corynebacteriaceae	*Corynebacterium*	*doosanense*	2	0.001
Actinobacteria	Actinobacteria	Actinomycetales	Corynebacteriaceae	*Corynebacterium*	*tuberculostearicum*	2	0.001

Proteobacteria	Alphaproteobacteria	Rhizobiales	Phyllobacteriaceae	*Pseudaminobacter*	*defluvii*	2	0.001
Proteobacteria	Gammaproteobacteria	Chromatiales	Ectothiorhodospiraceae	*Ectothiorhodospira*	*haloalkaliphila*	2	0.001
Actinobacteria	Actinobacteria	Actinomycetales	Nocardiaceae	*Nocardia*	*vermiculata*	2	0.001
Proteobacteria	Gammaproteobacteria	Xanthomonadales	Sinobacteraceae	*Steroidobacter*	*denitrificans*	2	0.001
Firmicutes	Clostridia	Clostridiales	Clostridiaceae	*Clostridium*	*histolyticum*	2	0.001
Proteobacteria	Betaproteobacteria	Rhodocyclales	Rhodocyclaceae	*Hydrogenophilus*	*hirschii*	2	0.001
Proteobacteria	Alphaproteobacteria	Rhizobiales	Hyphomicrobiaceae	*Rhodoplanes*		2	0.001
Proteobacteria	Gammaproteobacteria	Pseudomonadales	Pseudomonadaceae	*Pseudomonas*	*moraviensis*	2	0.001
Proteobacteria	Alphaproteobacteria	Rhodospirillales	Acetobacteraceae	*Gluconobacter*	*thailandicus*	2	0.001
Actinobacteria	Actinobacteria	Actinomycetales	Corynebacteriaceae	*Corynebacterium*	*acetoacidophilum*	2	0.001
Firmicutes	Bacilli	Bacillales	Bacillaceae	*Bacillus*	*methanolicus*	2	0.001
Firmicutes	Bacilli	Bacillales	Bacillaceae	*Anoxybacillus*		2	0.001
Bacteroidetes	Sphingobacteriia	Sphingobacteriales	Sphingobacteriaceae	*Sphingobacterium*		2	0.001
Proteobacteria	Alphaproteobacteria	Rhodospirillales	Rhodospirillaceae	*Skermanella*	*aerolata*	2	0.001
Firmicutes	Clostridia	Clostridiales	Heliobacteriaceae	*Heliorestis*	*baculata*	2	0.001
Proteobacteria	Betaproteobacteria	Burkholderiales	Comamonadaceae	*Tepidimonas*	*thermarum*	2	0.001
Actinobacteria	Actinobacteria	Bifidobacteriales	Bifidobacteriaceae	*Bifidobacterium*	*scardovii*	2	0.001
Proteobacteria	Gammaproteobacteria	Oceanospirillales	Halomonadaceae	*Halomonas*	*sediminis*	2	0.001
Actinobacteria	Actinobacteria	Actinomycetales	Micrococcaceae	*Kocuria*	*gwangalliensis*	2	0.001
Proteobacteria	Gammaproteobacteria	Pseudomonadales	Pseudomonadaceae	*Pseudomonas*	*chloritidismutans*	2	0.001
Actinobacteria	Actinobacteria	Actinomycetales	Gordoniaceae	*Gordonia*		2	0.001
Actinobacteria	Actinobacteria	Actinomycetales	Microbacteriaceae	*Microbacterium*	*ketosireducens*	2	0.001
Acidobacteria	Acidobacteria	Acidobacteriales	Acidobacteriaceae	*Granulicella*		2	0.001
Proteobacteria	Betaproteobacteria	Nitrosomonadales	Nitrosomonadaceae	*Nitrosovibrio*		2	0.001
Actinobacteria	Actinobacteria	Actinomycetales	Promicromonosporaceae	*Promicromonospora*		2	0.001
Actinobacteria	Actinobacteria	Actinomycetales	Streptosporangiaceae	*Streptosporangium*	*brasiliense*	2	0.001
Proteobacteria	Deltaproteobacteria	Desulfovibrionales	Desulfovibrionaceae	*Desulfovibrio*	*oryzae*	2	0.001
Proteobacteria	Deltaproteobacteria	Myxococcales	Polyangiaceae	*Chondromyces*		2	0.001
Proteobacteria	Gammaproteobacteria	Oceanospirillales	Halomonadaceae	*Halomonas*	*almeriensis*	2	0.001
Actinobacteria	Actinobacteria	Actinomycetales	Bogoriellaceae	*Georgenia*	*deserti*	2	0.001
Actinobacteria	Actinobacteria	Actinomycetales	Actinopolysporaceae	*Actinopolyspora*		2	0.001
Actinobacteria	Acidimicrobiia	Acidimicrobiales	Acidimicrobiaceae			2	0.001
Proteobacteria	Alphaproteobacteria	Rhizobiales	Bradyrhizobiaceae			2	0.001
Thermotogae	Thermotogae	Thermotogales	Thermotogaceae	*Fervidobacterium*	*pennivorans*	2	0.001
Proteobacteria	Betaproteobacteria	Burkholderiales	Comamonadaceae	*Variovorax*	*soli*	2	0.001
Firmicutes	Bacilli	Bacillales	Thermoactinomycetaceae	*Planifilum*	*fimeticola*	2	0.001
Actinobacteria	Actinobacteria	Actinomycetales	Glycomycetaceae	*Glycomyces*	*endophyticus*	2	0.001

Proteobacteria	Gammaproteobacteria	Oceanospirillales	Halomonadaceae	*Halomonas*	*fontilapidosi*	2	0.001
Bacteroidetes	Flavobacteriia	Flavobacteriales	Flavobacteriaceae	*Myroides*	*injenensis*	2	0.001
Proteobacteria	Alphaproteobacteria	Rhizobiales	Rhizobiaceae	*Agrobacterium*	*tumefaciens*	2	0.001
Firmicutes	Clostridia	Thermoanaerobacterales	Thermoanaerobacteraceae	*Moorella*	*glycerini*	2	0.001
Actinobacteria	Actinobacteria	Actinomycetales	Micrococcaceae	*Kocuria*	*assamensis*	2	0.001
Proteobacteria	Gammaproteobacteria	Xanthomonadales	Xanthomonadaceae	*Luteimonas*	*terricola*	2	0.001
Actinobacteria	Actinobacteria	Actinomycetales	Streptomycetaceae	*Streptomyces*	*olivogriseus*	2	0.001
Cyanobacteria	Synechococcophycideae	Synechococcales	Acaryochloridaceae	*Acaryochloris*		2	0.001
Firmicutes	Bacilli	Bacillales	Paenibacillaceae	*Ammoniphilus*	*oxalivorans*	2	0.001
Firmicutes	Bacilli	Lactobacillales	Lactobacillaceae			2	0.001
Proteobacteria	Deltaproteobacteria	Syntrophobacterales	Syntrophobacteraceae	*Desulfacinum*	*subterraneum*	2	0.001
Proteobacteria	Gammaproteobacteria	Pseudomonadales	Pseudomonadaceae	*Pseudomonas*	*teessidea*	2	0.001
Proteobacteria	Alphaproteobacteria	Rhizobiales	Aurantimonadaceae	*Aurantimonas*	*litoralis*	2	0.001
Proteobacteria	Deltaproteobacteria	Syntrophobacterales	Syntrophobacteraceae	*Syntrophobacter*	*wolinii*	2	0.001
Actinobacteria	Actinobacteria	Actinomycetales	Brevibacteriaceae	*Brevibacterium*	*antiquum*	2	0.001
Actinobacteria	Actinobacteria	Actinomycetales	Micrococcaceae	*Micrococcus*	*yunnanensis*	1	0.000
Proteobacteria	Alphaproteobacteria	Rhizobiales	Brucellaceae	*Ochrobactrum*		1	0.000
Proteobacteria	Betaproteobacteria	Rhodocyclales	Rhodocyclaceae	*Thauera*	*selenatis*	1	0.000
Bacteroidetes	Flavobacteriia	Flavobacteriales	Flavobacteriaceae	*Winogradskyella*		1	0.000
Actinobacteria	Actinobacteria	Actinomycetales	Actinomycetaceae	*Actinomyces*	*odontolyticus*	1	0.000
Thermi	Deinococci					1	0.000
Firmicutes	Clostridia	Coriobacteriales	Coriobacteriaceae	*Slackia*	*faecicanis*	1	0.000
Proteobacteria	Gammaproteobacteria	Alteromonadales	Alteromonadaceae	*Candidatus Endobugula*	*sertula*	1	0.000
Bacteroidetes	Flavobacteriia	Flavobacteriales	Flavobacteriaceae	*Flavobacterium*	*chungangense*	1	0.000
Proteobacteria	Alphaproteobacteria	Rhizobiales	Hyphomicrobiaceae	*Rhodoplanes*	*cryptolactis*	1	0.000
Proteobacteria	Betaproteobacteria	Burkholderiales	Comamonadaceae	*Rubrivivax*		1	0.000
Actinobacteria	Actinobacteria	Actinomycetales	Pseudonocardiaceae	*Pseudonocardia*	*zijingensis*	1	0.000
Actinobacteria	Actinobacteria	Actinomycetales	Micrococcaceae	*Microbispora*	*rosea*	1	0.000
Proteobacteria	Gammaproteobacteria	Enterobacteriales	Enterobacteriaceae	*Trabulsiella*	*odontotermitis*	1	0.000
Firmicutes	Bacilli	Lactobacillales	Aerococcaceae	*Aerococcus*	*viridans*	1	0.000
Proteobacteria	Gammaproteobacteria	Pseudomonadales	Moraxellaceae	*Acinetobacter*	*psychrotolerans*	1	0.000
Actinobacteria	Actinobacteria	Actinomycetales	Kineosporiaceae	*Kineosporia*	*rhizophila*	1	0.000
Actinobacteria	Actinobacteria	Actinomycetales	Actinomycetaceae	*Actinomyces*		1	0.000
Actinobacteria	Actinobacteria	Actinomycetales	Streptosporangiaceae	*Nonomuraea*	*asiatica*	1	0.000
Bacteroidetes	Flavobacteriia	Flavobacteriales	Flavobacteriaceae	*Bizionia*	*saleffrena*	1	0.000
Proteobacteria	Alphaproteobacteria	Caulobacterales	Caulobacteraceae	*Brevundimonas*	*bullata*	1	0.000
Actinobacteria	Actinobacteria	Actinomycetales	Intrasporangiaceae	*Phycicoccus*	*bigeumensis*	1	0.000

Proteobacteria	Alphaproteobacteria	Rhizobiales	Beijerinckiaceae	*Beijerinckia*	*mobilis*	1	0.000
Proteobacteria	Betaproteobacteria	Rhodocyclales	Rhodocyclaceae	*Propionivibrio*	*limicola*	1	0.000
Proteobacteria	Alphaproteobacteria	Rhizobiales	Methylobacteriaceae	*Methylobacterium*	*adhaesivum*	1	0.000
Firmicutes	Clostridia	Clostridiales	Clostridiaceae	*Sedimentibacter*	*hydroxybenzoicus*	1	0.000
Proteobacteria	Betaproteobacteria	Burkholderiales	Alcaligenaceae			1	0.000
Firmicutes	Clostridia	Clostridiales	Clostridiaceae	*Clostridium*	*taeniosporum*	1	0.000
Firmicutes	Bacilli	Lactobacillales	Streptococcaceae	*Streptococcus*	*pseudopneumoniae*	1	0.000
Proteobacteria	Betaproteobacteria	Burkholderiales	Comamonadaceae	*Limnohabitans*		1	0.000
Bacteroidetes	Sphingobacteriia	Sphingobacteriales	Flexibacteraceae	*Dyadobacter*	*hamtensis*	1	0.000
Proteobacteria	Alphaproteobacteria	Rhizobiales	Phyllobacteriaceae	*Aminobacter*	*aminovorans*	1	0.000
Firmicutes	Bacilli	Lactobacillales	Streptococcaceae	*Streptococcus*	*vestibularis*	1	0.000
Actinobacteria	Actinobacteria	Actinomycetales	Mycobacteriaceae	*Mycobacterium*	*senuense*	1	0.000
Actinobacteria	Actinobacteria	Actinomycetales	Bogoriellaceae	*Georgenia*	*ferrireducens*	1	0.000
Actinobacteria	Actinobacteria	Actinomycetales	Nocardiaceae	*Nocardia*	*alboflava*	1	0.000
Proteobacteria	Gammaproteobacteria	Alteromonadales	Shewanellaceae	*Shewanella*	*putrefaciens*	1	0.000
Actinobacteria	Actinobacteria	Actinomycetales	Microbacteriaceae	*Agromyces*	*subbeticus*	1	0.000
Proteobacteria	Alphaproteobacteria	Rhodobacterales	Rhodobacteraceae	*Paracoccus*	*sulfuroxidans*	1	0.000
Actinobacteria	Actinobacteria	Actinomycetales	Corynebacteriaceae	*Corynebacterium*	*hansenii*	1	0.000
Proteobacteria	Gammaproteobacteria	Alteromonadales	Alteromonadaceae	*Microbulbifer*	*epialgicus*	1	0.000
Actinobacteria	Actinobacteria	Actinomycetales	Actinomycetaceae	*Arcanobacterium*	*phocae*	1	0.000
Actinobacteria	Actinobacteria	Actinomycetales	Pseudonocardiaceae	*Pseudonocardia*	*benzenivorans*	1	0.000
Proteobacteria	Alphaproteobacteria	Sphingomonadales	Sphingomonadaceae	*Sphingopyxis*	*witflariensis*	1	0.000
Proteobacteria	Betaproteobacteria	Burkholderiales	Oxalobacteraceae	*Collimonas*	*pratensis*	1	0.000
Actinobacteria	Actinobacteria	Actinomycetales	Sporichthyaceae	*Sporichthya*	*brevicatena*	1	0.000
Actinobacteria	Actinobacteria	Actinomycetales	Corynebacteriaceae	*Corynebacterium*	*minutissimum*	1	0.000
Firmicutes	Clostridia	Clostridiales	Clostridiaceae	*Clostridium*	*thermobutyricum*	1	0.000
Proteobacteria	Gammaproteobacteria	Alteromonadales	Shewanellaceae	*Shewanella*	*pneumatophori*	1	0.000
Firmicutes	Clostridia	Clostridiales	Clostridiaceae	*Tindallia*	*magadiensis*	1	0.000
Firmicutes	Bacilli	Bacillales	Bacillaceae	*Bacillus*	*koreensis*	1	0.000
Fusobacteria	Fusobacteria	Fusobacteriales	Leptotrichiaceae	*Sebaldella*		1	0.000
Actinobacteria	Actinobacteria	Actinomycetales	Nocardiaceae	*Nocardia*	*gamkensis*	1	0.000
Proteobacteria	Alphaproteobacteria	Rhodobacterales	Rhodobacteraceae	*Paracoccus*	*kocurii*	1	0.000
Proteobacteria	Alphaproteobacteria	Rhizobiales	Methylobacteriaceae	*Methylobacterium*	*marchantiae*	1	0.000
Proteobacteria	Betaproteobacteria	Burkholderiales	Comamonadaceae	*Thiomonas*	*intermedia*	1	0.000
Actinobacteria	Actinobacteria	Actinomycetales	Propionibacteriaceae	*Propionibacterium*	*humerusii*	1	0.000
Bacteroidetes	Flavobacteriia	Flavobacteriales	Flavobacteriaceae	*Aquimarina*	*macrocephali*	1	0.000
Actinobacteria	Actinobacteria	Actinomycetales	Nocardiaceae	*Rhodococcus*	*yunnanensis*	1	0.000

Proteobacteria	Alphaproteobacteria	Sphingomonadales	Sphingomonadaceae	*Novosphingobium*	*capsulatum*	1	0.000
Proteobacteria	Gammaproteobacteria	Enterobacteriales	Enterobacteriaceae	*Candidatus Blochmannia*		1	0.000
Actinobacteria	Actinobacteria	Actinomycetales	Bogoriellaceae	*Georgenia*	*soli*	1	0.000
Actinobacteria	Actinobacteria	Actinomycetales	Microbacteriaceae	*Microbacterium*	*pygmaeum*	1	0.000
Proteobacteria	Gammaproteobacteria	Pseudomonadales	Pseudomonadaceae			1	0.000
Actinobacteria	Actinobacteria	Actinomycetales	Pseudonocardiaceae	*Amycolatopsis*	*methanolica*	1	0.000
Acidobacteria	Acidobacteria	Acidobacteriales	Acidobacteriaceae	*Acidobacterium*	*capsulatum*	1	0.000
Firmicutes	Clostridia	Clostridiales	Lachnospiraceae	*Catonella*	*morbi*	1	0.000
Proteobacteria	Alphaproteobacteria	Rhizobiales	Rhizobiaceae	*Rhizobium*	*alamii*	1	0.000
Bacteroidetes	Flavobacteriia	Flavobacteriales	Flavobacteriaceae	*Flavobacterium*	*croceum*	1	0.000
Proteobacteria	Gammaproteobacteria	Pseudomonadales	Moraxellaceae	*Enhydrobacter*	*aerosaccus*	1	0.000
Firmicutes	Bacilli	Bacillales	Paenibacillaceae			1	0.000
Actinobacteria	Actinobacteria	Actinomycetales	Micrococcaceae	*Arthrobacter*	*halodurans*	1	0.000
Proteobacteria	Alphaproteobacteria	Rhodospirillales	Acetobacteraceae	*Roseococcus*		1	0.000
Firmicutes	Clostridia	Clostridiales	Syntrophomonadaceae	*Syntrophomonas*		1	0.000
Firmicutes	Clostridia	Clostridiales	Syntrophomonadaceae	*Syntrophomonas*	*bryantii*	1	0.000
Proteobacteria	Gammaproteobacteria	Oceanospirillales	Litoricolaceae	*Litoricola*	*marina*	1	0.000
Firmicutes	Clostridia	Thermoanaerobacterales	Thermoanaerobacteraceae	*Thermoanaerobacter*	*sulfurigignens*	1	0.000
Firmicutes	Bacilli	Bacillales	Paenibacillaceae	*Cohnella*	*thermotolerans*	1	0.000
Proteobacteria	Gammaproteobacteria	Pseudomonadales	Pseudomonadaceae	*Pseudomonas*	*stutzeri*	1	0.000
Actinobacteria	Actinobacteria	Actinomycetales	Mycobacteriaceae	*Mycobacterium*	*novocastrense*	1	0.000
Acidobacteria	Acidobacteria	Acidobacteriales	Acidobacteriaceae	*Terriglobus*		1	0.000
Actinobacteria	Actinobacteria	Actinomycetales	Micrococcaceae	*Kocuria*		1	0.000
Proteobacteria	Alphaproteobacteria	Rhizobiales	Methylocystaceae	*Methylosinus*	*pucelana*	1	0.000
Proteobacteria	Gammaproteobacteria	Legionellales	Legionellaceae	*Legionella*	*shakespearei*	1	0.000
Proteobacteria	Alphaproteobacteria	Rhizobiales	Bradyrhizobiaceae	*Balneimonas*		1	0.000
Firmicutes	Bacilli	Bacillales	Paenibacillaceae	*Paenibacillus*	*darangshiensis*	1	0.000
Actinobacteria	Actinobacteria	Actinomycetales	Microbacteriaceae	*Frigoribacterium*		1	0.000
Proteobacteria	Gammaproteobacteria	Chromatiales	Chromatiaceae	*Allochromatium*	*palmeri*	1	0.000
Proteobacteria	Alphaproteobacteria	Rhizobiales	Bradyrhizobiaceae	*Nitrobacter*	*hamburgensis*	1	0.000
Bacteroidetes	Flavobacteriia	Flavobacteriales	Flavobacteriaceae	*Flavobacterium*	*kamogawaensis*	1	0.000
Firmicutes	Clostridia	Clostridiales	Sulfobacillaceae	*Sulfobacillus*		1	0.000
Firmicutes	Bacilli	Bacillales	Staphylococcaceae	*Macrococcus*	*bovicus*	1	0.000
Actinobacteria	Actinobacteria	Actinomycetales	Williamsiaceae	*Williamsia*	*marianensis*	1	0.000
Actinobacteria	Actinobacteria	Actinomycetales	Cellulomonadaceae	*Cellulomonas*	*oligotrophica*	1	0.000
Proteobacteria	Alphaproteobacteria	Rhodospirillales	Acetobacteraceae	*Acidisoma*		1	0.000
Actinobacteria	Actinobacteria	Actinomycetales	Intrasporangiaceae	*Phycicoccus*	*jejuensis*	1	0.000

Proteobacteria	Alphaproteobacteria	Sphingomonadales	Sphingomonadaceae	*Sphingomonas*	*yabuuchiae*	1	0.000
Proteobacteria	Deltaproteobacteria	Myxococcales				1	0.000
Actinobacteria	Actinobacteria	Actinomycetales	Brevibacteriaceae	*Brevibacterium*	*samyangense*	1	0.000
Proteobacteria	Gammaproteobacteria	Acidithiobacillales	Acidithiobacillaceae	*Acidithiobacillus*	*albertensis*	1	0.000
Firmicutes	Clostridia	Clostridiales	Eubacteriaceae	*Acetobacterium*	*tundrae*	1	0.000
Proteobacteria	Gammaproteobacteria	Alteromonadales	Alteromonadaceae	*Marinobacter*	*szutsaonensis*	1	0.000
Firmicutes	Clostridia	Halanaerobiales	Halanaerobiaceae	*Halanaerobium*	*fermentans*	1	0.000
Proteobacteria	Alphaproteobacteria	Rhodospirillales	Acetobacteraceae	*Roseococcus*	*suduntuyensis*	1	0.000
Actinobacteria	Actinobacteria	Actinomycetales	Corynebacteriaceae	*Corynebacterium*	*imitans*	1	0.000
Proteobacteria	Alphaproteobacteria	Rhizobiales	Phyllobacteriaceae	*Mesorhizobium*		1	0.000
Actinobacteria	Actinobacteria	Actinomycetales	Microbacteriaceae	*Curtobacterium*	*albidum*	1	0.000
Proteobacteria	Betaproteobacteria	Burkholderiales	Comamonadaceae	*Pelomonas*	*puraquae*	1	0.000
Proteobacteria	Alphaproteobacteria	Caulobacterales	Caulobacteraceae	*Asticcacaulis*		1	0.000
Actinobacteria	Actinobacteria	Actinomycetales	Micrococcaceae	*Micrococcus*		1	0.000
Proteobacteria	Gammaproteobacteria	Pseudomonadales	Pseudomonadaceae	*Pseudomonas*	*proteolytica*	1	0.000
Proteobacteria	Gammaproteobacteria	Oceanospirillales	Oceanospirillaceae	*Oceanospirillum*	*linum*	1	0.000
Firmicutes	Bacilli	Lactobacillales	Streptococcaceae	*Streptococcus*	*bovis*	1	0.000
Proteobacteria	Alphaproteobacteria	Caulobacterales	Caulobacteraceae	*Asticcacaulis*	*taihuensis*	1	0.000
Actinobacteria	Actinobacteria	Actinomycetales	Intrasporangiaceae	*Knoellia*		1	0.000
Firmicutes	Bacilli	Bacillales	Bacillaceae	*Bacillus*	*niacini*	1	0.000
Proteobacteria	Alphaproteobacteria	Rhizobiales	Methylobacteriaceae	*Methylobacterium*	*radiotolerans*	1	0.000
Proteobacteria	Deltaproteobacteria	Desulfuromonadales	Geobacteraceae	*Geobacter*	*toluenoxydans*	1	0.000
Firmicutes	Clostridia	Clostridiales	Clostridiaceae	*Clostridium*	*magnum*	1	0.000
Thermi	Deinococci	Deinococcales	Deinococcaceae	*Deinococcus*	*piscis*	1	0.000
Actinobacteria	Actinobacteria	Actinomycetales	Micromonosporaceae	*Actinoplanes*	*pallidoaurantiacus*	1	0.000
Firmicutes	Clostridia	Clostridiales	Eubacteriaceae	*Acetobacterium*	*malicum*	1	0.000
Actinobacteria	Actinobacteria	Actinomycetales	Streptomycetaceae	*Streptomyces*	*thermoluteus*	1	0.000
Proteobacteria	Gammaproteobacteria	Thiotrichales	Piscirickettsiaceae	*Thiomicrospira*		1	0.000
Proteobacteria	Gammaproteobacteria	Vibrionales	Vibrionaceae	*Vibrio*		1	0.000
Thermi	Deinococci	Thermales	Thermaceae	*Thermus*	*rehai*	1	0.000
Firmicutes	Clostridia	Clostridiales	Clostridiaceae	*Natronincola*		1	0.000
Firmicutes	Bacilli	Lactobacillales	Lactobacillaceae	*Pediococcus*		1	0.000
Proteobacteria	Deltaproteobacteria	Syntrophobacterales	Desulfobacteraceae	*Desulfosarcina*	*cetonica*	1	0.000
Actinobacteria	Actinobacteria	Actinomycetales	Nocardiopsaceae	*Nocardiopsis*	*alba*	1	0.000
Proteobacteria	Deltaproteobacteria	Desulfobacterales	Desulfobulbaceae	*Desulfocapsa*	*sulfexigens*	1	0.000
Firmicutes	Bacilli	Bacillales	Bacillaceae	*Bacillus*	*ginsenggisoli*	1	0.000
Firmicutes	Clostridia	Clostridiales	Syntrophomonadaceae	*Syntrophomonas*	*cellicola*	1	0.000

Actinobacteria	Actinobacteria	Actinomycetales	Actinomycetaceae	*Actinomyces*	*meyeri*	1	0.000
Proteobacteria	Alphaproteobacteria	Rhizobiales	Phyllobacteriaceae	*Phyllobacterium*	*bourgognense*	1	0.000
Proteobacteria	Deltaproteobacteria	Desulfovibrionales	Desulfovibrionaceae	*Desulfovibrio*	*aceae*	1	0.000
Proteobacteria	Gammaproteobacteria	Thiotrichales	Piscirickettsiaceae	*Thiomicrospira*	*thermophila*	1	0.000
Proteobacteria	Alphaproteobacteria	Caulobacterales	Caulobacteraceae	*Caulobacter*		1	0.000
Proteobacteria	Alphaproteobacteria	Caulobacterales	Caulobacteraceae	*Caulobacter*	*tundrae*	1	0.000
Proteobacteria	Alphaproteobacteria	Rhizobiales	Rhizobiaceae	*Agrobacterium*		1	0.000
Proteobacteria	Gammaproteobacteria	Pseudomonadales	Pseudomonadaceae	*Pseudomonas*	*lundensis*	1	0.000
Proteobacteria	Gammaproteobacteria	Alteromonadales	Ferrimonadaceae	*Ferrimonas*		1	0.000
Actinobacteria	Actinobacteria	Actinomycetales	Microbacteriaceae	*Agromyces*	*ramosus*	1	0.000
Proteobacteria	Gammaproteobacteria	Thiotrichales	Piscirickettsiaceae	*Thiomicrospira*	*frisia*	1	0.000
Actinobacteria	Actinobacteria	Actinomycetales	Micrococcaceae	*Rothia*	*dentocariosa*	1	0.000
Proteobacteria	Deltaproteobacteria	Desulfovibrionales	Desulfovibrionaceae	*Desulfovibrio*	*butyratiphilus*	1	0.000
Firmicutes	Bacilli	Lactobacillales	Streptococcaceae	*Streptococcus*	*lactarius*	1	0.000
Firmicutes	Clostridia	Clostridiales	Clostridiaceae	*Finegoldia*	*magna*	1	0.000
Proteobacteria	Gammaproteobacteria	Oceanospirillales	Halomonadaceae	*Halomonas*	*neptunia*	1	0.000
Firmicutes	Bacilli	Bacillales	Paenibacillaceae	*Ammoniphilus*		1	0.000
Proteobacteria	Alphaproteobacteria	Rhodobacterales	Rhodobacteraceae	*Amaricoccus*	*macauensis*	1	0.000
Actinobacteria	Actinobacteria	Actinomycetales	Pseudonocardiaceae	*Saccharopolyspora*	*halophila*	1	0.000
Actinobacteria	Actinobacteria	Actinomycetales	Micrococcaceae	*Arthrobacter*	*uratoxydans*	1	0.000
Cyanobacteria	Nostocophycideae	Nostocales	Nostocaceae	*Dolichospermum*	*affine*	1	0.000
Proteobacteria	Deltaproteobacteria	Desulfovibrionales	Desulfovibrionaceae	*Desulfovibrio*	*piger*	1	0.000
Bacteroidetes	Bacteroidia	Bacteroidales	Bacteroidaceae	*Bacteroides*	*denticanum*	1	0.000
Proteobacteria	Betaproteobacteria	Burkholderiales	Burkholderiaceae	*Burkholderia*	*fungorum*	1	0.000
Proteobacteria	Gammaproteobacteria	Pseudomonadales	Pseudomonadaceae	*Pseudomonas*	*mosselii*	1	0.000
Proteobacteria	Betaproteobacteria	Burkholderiales	Comamonadaceae	*Polaromonas*	*vacuolata*	1	0.000
Bacteroidetes	Flavobacteriia	Flavobacteriales	Flavobacteriaceae	*Polaribacter*		1	0.000
Proteobacteria	Alphaproteobacteria	Rhodobacterales	Rhodobacteraceae	*Marivita*		1	0.000
Cyanobacteria	Nostocophycideae	Nostocales	Nostocaceae	*Nostoc*	*ellipsosporum*	1	0.000
Proteobacteria	Alphaproteobacteria	Rhodobacterales	Rhodobacteraceae	*Amaricoccus*		1	0.000
Proteobacteria	Gammaproteobacteria	Alteromonadales	Idiomarinaceae	*Pseudidiomarina*		1	0.000
Proteobacteria	Epsilonproteobacteria	Campylobacterales	Campylobacteraceae	*Campylobacter*	*faecalis*	1	0.000
Actinobacteria	Actinobacteria	Actinomycetales	Pseudonocardiaceae	*Pseudonocardia*	*petroleophila*	1	0.000
Proteobacteria	Gammaproteobacteria	Thiotrichales	Piscirickettsiaceae			1	0.000
Firmicutes	Bacilli	Bacillales	Planococcaceae	*Planomicrobium*	*chinense*	1	0.000
Actinobacteria	Actinobacteria	Actinomycetales	Actinomycetaceae	*Arcanobacterium*		1	0.000
Bacteroidetes	Sphingobacteriia	Sphingobacteriales	Chitinophagaceae	*Chitinophaga*		1	0.000

Actinobacteria	Actinobacteria	Actinomycetales	Streptomycetaceae	*Streptomyces*	*goraiensis*	1	0.000
Firmicutes	Clostridia	Coriobacteriales	Coriobacteriaceae	*Eggerthella*	*sinensis*	1	0.000
Synergistetes	Synergistia	Synergistales	Dethiosulfovibrionaceae	*Dethiosulfovibrio*		1	0.000
Proteobacteria	Gammaproteobacteria	Enterobacteriales	Enterobacteriaceae	*Yersinia*	*massiliensis*	1	0.000
Proteobacteria	Alphaproteobacteria	Rhodobacterales	Rhodobacteraceae	*Amaricoccus*	*kaplicensis*	1	0.000
Firmicutes	Bacilli	Bacillales	Bacillaceae	*Bacillus*	*hackensackii*	1	0.000
Thermodesulfobacteria	Thermodesulfobacteria	Thermodesulfobacteriales	Thermodesulfobacteriaceae	*Thermodesulfatator*	*atlanticus*	1	0.000
Proteobacteria	Alphaproteobacteria	Rhizobiales	Bradyrhizobiaceae	*Bradyrhizobium*	*jicamae*	1	0.000
Actinobacteria	Actinobacteria	Actinomycetales	Dermabacteraceae	*Brachybacterium*	*alimentarium*	1	0.000
Proteobacteria	Gammaproteobacteria	Oceanospirillales	Halomonadaceae	*Kushneria*	*aurantia*	1	0.000
Actinobacteria	Actinobacteria	Actinomycetales	Streptosporangiaceae	*Streptosporangium*	*purpuratum*	1	0.000
Proteobacteria	Betaproteobacteria	Burkholderiales	Alcaligenaceae	*Pigmentiphaga*		1	0.000
Proteobacteria	Betaproteobacteria	Burkholderiales	Oxalobacteraceae	*Polynucleobacter*	*rarus*	1	0.000
Firmicutes	Bacilli	Bacillales	Staphylococcaceae			1	0.000
Actinobacteria	Acidimicrobiia	Acidimicrobiales				1	0.000
Proteobacteria	Betaproteobacteria	Rhodocyclales	Rhodocyclaceae	*Denitratisoma*		1	0.000
Firmicutes	Clostridia	Clostridiales	Clostridiaceae	*Anaerococcus*	*octavius*	1	0.000
Firmicutes	Bacilli	Lactobacillales	Streptococcaceae	*Streptococcus*	*parasanguinis*	1	0.000
Firmicutes	Bacilli	Bacillales	Planococcaceae	*Planococcus*	*maritimus*	1	0.000
Thermotogae	Thermotogae	Thermotogales	Thermotogaceae	*Thermosipho*	*ferriphilus*	1	0.000
Proteobacteria	Alphaproteobacteria	Rhodobacterales	Rhodobacteraceae	*Paracoccus*	*homiensis*	1	0.000
Proteobacteria	Alphaproteobacteria	Rhodobacterales	Hyphomonadaceae	*Hyphomonas*		1	0.000
Actinobacteria	Actinobacteria	Actinomycetales	Pseudonocardiaceae	*Saccharomonospora*		1	0.000
Bacteroidetes	Flavobacteriia	Flavobacteriales	Flavobacteriaceae	*Flavobacterium*	*succinicans*	1	0.000
Proteobacteria	Alphaproteobacteria	Sphingomonadales	Erythrobacteraceae	*Erythrobacter*	*aquimaris*	1	0.000
Bacteroidetes	Sphingobacteriia	Sphingobacteriales	Flexibacteraceae	*Emticicia*	*oligotrophica*	1	0.000
Bacteroidetes	Sphingobacteriia	Sphingobacteriales	Chitinophagaceae	*Chitinophaga*	*terrae*	1	0.000
Actinobacteria	Actinobacteria	Actinomycetales	Streptomycetaceae	*Streptomyces*	*coriofaciens*	1	0.000
Bacteroidetes	Sphingobacteriia	Sphingobacteriales	Sphingobacteriaceae	*Parapedobacter*	*koreensis*	1	0.000
Firmicutes	Clostridia	Clostridiales	Clostridiaceae	*Sarcina*	*maxima*	1	0.000
Proteobacteria	Betaproteobacteria	Neisseriales	Neisseriaceae	*Chromobacterium*	*subtsugae*	1	0.000
Actinobacteria	Actinobacteria	Actinomycetales	Thermomonosporaceae	*Actinocorallia*	*herbida*	1	0.000
Actinobacteria	Actinobacteria	Actinomycetales	Corynebacteriaceae	*Corynebacterium*	*pyruviciproducens*	1	0.000
Actinobacteria	Actinobacteria	Actinomycetales	Gordoniaceae	*Gordonia*	*rhizosphera*	1	0.000
Actinobacteria	Actinobacteria	Actinomycetales	Mycobacteriaceae	*Mycobacterium*	*buckleii*	1	0.000
Actinobacteria	Actinobacteria	Actinomycetales	Microbacteriaceae	*Cryobacterium*		1	0.000
Proteobacteria	Alphaproteobacteria	Kiloniellales	Kiloniellaceae	*Thalassospira*	*tepidiphila*	1	0.000

Actinobacteria	Actinobacteria	Actinomycetales	Pseudonocardiaceae	*Saccharomonospora*	*thermoviridis*	1	0.000
Bacteroidetes	Sphingobacteriia	Sphingobacteriales	Flexibacteraceae	*Hymenobacter*	*ocellatus*	1	0.000
Proteobacteria	Gammaproteobacteria	Pseudomonadales	Moraxellaceae	*Psychrobacter*		1	0.000
Firmicutes	Clostridia	Clostridiales	Clostridiaceae	*Clostridium*	*tagluense*	1	0.000
Firmicutes	Clostridia	Clostridiales	Ruminococcaceae	*Faecalibacterium*		1	0.000
Proteobacteria	Gammaproteobacteria	Pseudomonadales	Pseudomonadaceae	*Pseudomonas*	*alcaliphila*	1	0.000
Proteobacteria	Deltaproteobacteria	Desulfuromonadales	Geobacteraceae	*Geobacter*		1	0.000
Actinobacteria	Actinobacteria	Actinomycetales	Streptosporangiaceae	*Streptosporangium*	*yunnanense*	1	0.000
Actinobacteria	Actinobacteria	Actinomycetales	Streptomycetaceae	*Kitasatospora*	*melanogena*	1	0.000
Bacteroidetes	Bacteroidia	Bacteroidales	Prevotellaceae	*Prevotella*		1	0.000
Firmicutes	Bacilli	Exiguobacterales	Exiguobacteraceae	*Exiguobacterium*	*taiwanense*	1	0.000
Proteobacteria	Alphaproteobacteria	Rhodospirillales	Rhodospirillaceae	*Roseospira*	*thiosulfatophila*	1	0.000
Thermi	Deinococci	Deinococcales	Deinococcaceae	*Deinococcus*	*caeni*	1	0.000
Firmicutes	Clostridia	Clostridiales	Peptococcaceae	*Desulfitobacterium*	*chlororespirans*	1	0.000
Actinobacteria	Actinobacteria	Actinomycetales	Dermacoccaceae	*Dermacoccus*		1	0.000
Thermi	Deinococci	Deinococcales	Deinococcaceae	*Deinococcus*	*aeria*	1	0.000
Firmicutes	Bacilli	Bacillales	Planococcaceae	*Planococcus*	*columbae*	1	0.000
Proteobacteria	Betaproteobacteria	Burkholderiales	Comamonadaceae	*Variovorax*	*dokdonensis*	1	0.000
Firmicutes	Clostridia	Coriobacteriales	Coriobacteriaceae	*Slackia*	*piriformis*	1	0.000
Firmicutes	Clostridia	Clostridiales	Clostridiaceae	*Clostridium*	*akagii*	1	0.000
Proteobacteria	Betaproteobacteria	Burkholderiales	Comamonadaceae	*Ramlibacter*	*tataouinensis*	1	0.000
Proteobacteria	Gammaproteobacteria	Legionellales	Legionellaceae	*Legionella*	*cherrii*	1	0.000
Firmicutes	Bacilli	Bacillales	Paenibacillaceae	*Paenibacillus*	*pinihumi*	1	0.000
Bacteroidetes	Sphingobacteriia	Sphingobacteriales	Chitinophagaceae	*Niastella*	*populi*	1	0.000
Proteobacteria	Betaproteobacteria	Rhodocyclales	Rhodocyclaceae	*Sulfuritalea*		1	0.000
Firmicutes	Clostridia	Clostridiales	Clostridiaceae	*Peptoniphilus*	*ivorii*	1	0.000
Firmicutes	Bacilli	Bacillales	Bacillaceae	*Bacillus*	*pocheonensis*	1	0.000
Proteobacteria	Deltaproteobacteria	Desulfovibrionales	Desulfovibrionaceae	*Desulfovibrio*	*vietnamensis*	1	0.000
Actinobacteria	Actinobacteria	Actinomycetales	Nocardioidaceae	*Kribbella*		1	0.000
Firmicutes	Clostridia	Clostridiales	Clostridiaceae	*Peptoniphilus*	*tyrrelliae*	1	0.000
Proteobacteria	Alphaproteobacteria	Sphingomonadales	Sphingomonadaceae	*Novosphingobium*	*indicum*	1	0.000
Proteobacteria	Gammaproteobacteria	Enterobacteriales	Enterobacteriaceae	*Erwinia*	*billingiae*	1	0.000
Cyanobacteria	Oscillatoriophycideae	Chroococcales	Phormidiaceae	*Trichodesmium*	*havanum*	1	0.000
Actinobacteria	Actinobacteria	Actinomycetales	Streptomycetaceae	*Streptomyces*	*qinlingensis*	1	0.000
Actinobacteria	Actinobacteria	Actinomycetales	Propionibacteriaceae	*Luteococcus*	*peritonei*	1	0.000
Actinobacteria	Actinobacteria	Actinomycetales	Micromonosporaceae	*Actinoplanes*	*digitatis*	1	0.000
Proteobacteria	Alphaproteobacteria	Rhizobiales	Beijerinckiaceae	*Methylocella*	*silvestris*	1	0.000

Proteobacteria	Alphaproteobacteria	Rhizobiales	Hyphomicrobiaceae	*Devosia*	*limi*	1	0.000

Table S2. Presence of spots in gels from glacier samples and from cultures

Spot	Protein	Microorganism	NCBI Accession N°	Glacier samples	Samples from cultures							
				T1	T2	T3	T4	T5	T6	T7	T8	T9
1	Isocitrate dehydrogenase	*Pseudomonas*	WP_003219599									
2	Succinate-CoA ligase subunit beta	*Pseudomonas*	WP_003179235									
3	Isovaleryl-CoA dehydrogenase	*Pseudomonas*	WP_007904074									
4	Molecular chaperone GroEL	*Pseudomonas*	WP_007898438									
5	Oxidoreductase	*Klebsiella pneumoniae*	WP_004151005									
6	Molecular chaperone DnaK	*Pseudomonas mandelii*	WP_010463518									
7	Hypothetical protein	Multispecies	WP_004152656									
8	Glucosamine-fructose-6 P aminotransferase	*Salinispora arenicola*	WP_012184269									
9	Glycerol kinase	*Dickeya dadantii*	WP_013315890									
10	ATPase	*Pseudomonas* sp.	WP_004152656									
11	Hypothetical protein	*Methanobrevibacter smithii*	WP_019267207									
12	Recombinase	*Clostridium scindens*	WP_004608538									
13	DNA-binding response regulator	*Methylobacterium* sp. GXF4	WP_007559895									
14	Succinate-CoA ligase subunit alpha	*Pseudomonas*	WP_003210566									
15	Elongation factor Tu	*Pseudomonas*	WP_008145495									
16	Malonyl-CoA O-methyltransferase	*Geobacter uraniireducens* Rf4	WP_011940068									
17	MoxR family ATPase	*Bacillus cereus*	WP_000892980									
18	Amino acid ABC transporter substrate-binding protein	*Pseudomonas fluorescens*	WP_019691964									
19	ATP synthase subunit beta	*Pseudomonas* sp.	WP_007903035									
20	DUF262 domain-containing protein	*Pseudomonas aeruginosa*	WP_016253918									
21	O-succinylhomoserine sulfhydrylase	*Burkholderia*	WP_006404010									
22	Hypothetical protein NSED_02180	*Candidatus Nitrosopumilus* sp. AR2	WP_014964618									
23	Hypothetical protein	*Caldibacillus debilis*	WP_020154942									
24	Ketol-acid reductoisomerase	*Pseudomonas brassicacearum*	WP_003185459									
25	LLM class flavin-dependent oxidoreductase	*Nocardia speluncae*	WP_068047617									
26	6-phosphogluconate dehydrogenase (decarboxylating)	*Microbispora* sp. GKU 823	WP_079314207									
27	Arylsulfatase A family protein	*Pseudomonas* sp. GM33	ZP_10673425									
28	Ferritin	Geobacteraceae bacterium GWC2_55_20	OGU06320									

29	Acetyl-CoA acetyltransferase	*Pseudomonas*	WP_056742244									
30	30S ribosomal protein S1	*Pseudomonas*	WP_005786304									
31	ATPase	*Pseudomonas*	WP_007903032									
32	ATPase	*Pseudomonas fluorescens*	WP_042557759									
33	PhoH family protein	*Pseudomonas* sp. GM21	WP_007942274									
34	Succinyl-CoA synthetase subunit alpha	*Pseudomonas* sp. Ag1	WP_008148605									
35	Translation elongation factor TU	*Pseudomonas* sp. GM41	ZP_10666373									
36	Periplasmic component of amino acid ABC-type transporter/signal transduction system	*Pseudomonas* sp. GM18	ZP_10703116									
37	ATP synthase, F1 beta subunit	*Pseudomonas* sp. GM102	WP_007903035									
38	Glycine cleavage system T protein	*Pseudomonas* sp. GM41	WP_008146923									
39	Hypothetical protein	*Bryobacter aggregatus*	WP_080508095									
40	Polyketide synthase	*Bacillus subtilis*	WP_003245093									
41	Amino acid ABC transporter substrate-binding protein	*Halomonas* sp. BC04	EWG99667									
42	4-diphosphocytidyl-2-C-methyl-D-erythritol kinase	*Pelosinus fermentans* JBW45	AJQ25869									
43	Hypothetical protein HLUCCO06_06545	*Halomonas* sp. HL-93	KPQ26449									
44	Uvr ABC system protein C	*Stenotrophomonas maltophilia* (K279a)	WP_005408879									
45	Hypothetical protein	*Ruegeria lacuscaerulensis*	WP_005979176									
46	Hypothetical protein	*Corynebacterium falsenii*	WP_081737368									
47	Hypothetical protein	*Roseatales depolymerans*	WP_058934563									
48	Hypothetical protein	*Bacillus licheniformis* CG-B52	N399_09015									
49	50S ribosomal protein L17	*Gallibacterium* sp.	EQM28295									
50	Elongation factor P lysine(34) lysyltransferase	*Vibrio nigripulchritudo*	WP_065234045									
51	Hypothetical protein A2156_01595	Deltaproteobacteria bacterium RBG_16_48_10	WP_004399847									
52	Molybdopterin-guanine dinucleotide biosynthesis protein A	*Roseovarius mucosus* DSM 17069	OGP87982									
53	Phosphate ABC transporter ATP-binding protein	*Thalassospira mesophila*	WP_085579862									
54	Hypothetical protein A2664_03710	*Candidatus Taylorbacteria* bacterium	OHA17696									
55	tRNA-specific adenosine deaminase	*Actinomyces timonensis*	WP_082181133									
56	Branched-chain amino acid ABC transporter substrate-binding protein	*Pseudomonas protegens* Pf-5	WP_011058966									
57	MerR family transcriptional	*Streptomyces aureofaciens*	OEV38922									

58	Hypothetical protein SAMN02982985_04871	*Rugamonas rubra*	SFM66411								■	
59	Hypothetical protein	*Bacillus alkalitelluris*	WP_088074644			■						
60	Ribosome biogenesis GTPase Der	*Methylibium petroleiphilum*	WP_011829587			■						
61	Leucine ABC transporter subunit substrate-binding protein LivK	*Pseudomonas protegens* Pf-5	WP_011059642					■	■			
62	Class I SAM-dependent methyltransferase	*Mycobacterium* sp. E802	WP_067767194			■						
63	Hypothetical protein L917_14538	*Phytophthora parasitica*	ETL85988			■						
64	Collagenase	*Porphyromonas gingivalis*	P33437								■	
65	ABC transporter substrate-binding protein	*Acinetobacter* sp. CIP 64.2	WP_005243276			■						
66	Plasmid stabilization protein	*Pseudomonas* sp. A46	WP_088191231									
67	Phosphoenolpyruvate carboxykinase (ATP)	*Selenomonas* sp.	WP_009440364			■		■	■			
68	DNA-binding response regulator, partial	*Streptomyces hirsutus*	WP_055596475			■						
69	Conjugative transfer relaxase/helicase TraI	*Klebsiella oxytoca*	WP_064349674								■	
70	Mo cofactor biosysynthesis protein	*Streptomyces*	WP_030657664			■						
71	50S ribosomal protein L3	*Serratia*	WP_012147224								■	
72	Succinyl-CoA synthetase subunit beta	*Pseudomonas putida* KT2440	NP_746303					■	■			
73	Lysine-tRNA ligase	*Ruminococcus* sp. CAG:353	CDE80170								■	
74	Hypothetical protein UV73_C0010G0061	*Candidatus Gottesmanbacteria* bacterium GW2011_GWA2_43_14	KKS96476		■							
75	ABC transporter substrate-binding protein	*Pseudomonas putida* KT2440	NP_743302					■	■			
76	yaaT protein	*Dialister invisus* CAG:218	CCZ54943			■						
77	Hypothetical protein GL50803_115478	*Giardia lamblia* ATCC 50803	XP_001705438			■						
78	Sugar ABC transporter substrate-binding protein	*Pseudomonas extremaustralis*	WP_010567089					■	■			
79	Amino acid ABC transporter substrate-binding protein	*Pseudomonas* sp. 11/12A	WP_047538293					■	■			
80	Hypothetical protein A2Z93_11965	*Curvibacter* sp. GWA2_64_110	OGO97834					■	■			
81	Sensor histidine kinase	*Thermosulfurimonas dismutans*	WP_068671481							■		
82	Transcriptional regulator, IclR family	*Megasphaera paucivorans*	SDN17589					■			■	
83	Transcriptional regulator NrdR	*Zymomonas mobilis*	WP_012816913									
84	Uncharacterized protein	*Mycobacterium*	SHY74220			■						
85	Hypothetical protein	*Anaeromusa acidaminophila*	WP_018701773					■				
86	DUF3284 domain-containing protein	*Lactobacillus gasseri*	WP_049149326			■						
87	Hypothetical protein	*Chitinophaga niabensis*	WP_074239494		■							
88	Hypothetical protein	*Mycobacterium* sp. E802	WP_067770147			■				■		
89	Acetyl-CoA carboxylase carboxyl	unclassified Hyphomonadaceae	OUV66025					■	■			

	transferase subunit alpha											
90	Glutamate/aspartate ABC transporter periplasmic glutamate/aspartate-binding protein GltI	*Pseudomonas protegens* Pf-5	WP_011063125									
91	Elongation factor Tu	*Pseudomonas*	WP_046047317									
92	N-acetylmuramoyl-L-alanine amidase	*Bacillus cereus*	WP_080353948									
93	Hypothetical protein	*Acetobacter tropicalis*	WP_086640703									
94	B12-dependent methionine synthase	*Mycobacterium intracellulare*	WP_014380026									
95	Elongation factor Tu	*Actinobacillus pleuropneumoniae*	EFTU_ACTPJ									
96	Flagellar MS-ring protein	*Xanthomonas campestris*	KFA35468									
97	Hypothetical protein	*Streptomyces* sp. ERV7	WP_067156483									
98	Dihydrolipoyl dehydrogenase	*Pseudomonas fluorescens*	DLDH_PSEFL									
99	Penicillin-binding protein 2	*Clostridium botulinum*	WP_019278306									
100	Phosphohydrolase	*Deinococcus geothermalis*	WP_011529283									
101	Glutamate carboxypeptidase	*Variovorax* sp. EL159	SCX73833									
102	DNA mismatch repair protein	*Petrotoga mobilis*	WP_012208437									
103	Hypothetical protein A2Y25_00865	*Candidatus Melainabacteria* bacterium	OGI01296									
104	Hypothetical protein	*Bacteroides uniformis*	WP_057098237									
105	ATP synthase subunit beta	*Renibacterium salmoninarum*	WP_041684542									
106	Acetyl-CoA carboxylase carboxyl transferase subunit alpha	*Marinobacter hydrocarbonoclasticus*	WP_014421920									
107	Protein serine/threonine phosphatase	*Acetobacter* sp.	CCZ21917									
108	TadE family protein	*Rhodanobacter spathiphylli*	WP_007807391									
109	Hypothetical protein BN2364_4186	*Alcanivorax dieselolei*	CUR48627									
110	Aldehyde dehydrogenase family protein	*Staphylococcus aureus*	WP_001206106									
111	Penicillin-binding protein 2 2e-12	*Bacillus cereus*	WP_001249177									
112	Threonine ammonia-lyase	*Sediminibacterium* sp. C3	WP_026750379									
113	Helix-turn-helix domain-containing protein	*Pseudorhodobacter ferrugineus*	WP_022703777									
114	DNA-directed RNA polymerase subunit alpha	*Pseudomonas*	WP_003176403									
115	DNA ligase (NAD(+)) LigA	*Campylobacter concisus*	WP_087582529									
116	Tetratricopeptide repeat protein	*Thalassobaculum salexigens*	WP_028795026									
117	Hypothetical protein AKJ43_00885	candidate divison MSBL1 archaeon SCGC-AAA261D19	KXB02680									
118	Hypothetical protein WH7805_11873	*Synechococcus* sp. WH 7805	EAR19613									
119	Hypothetical protein OC00_16270	*Xanthomonas vasicola*	KGT82980									
120	Branched-chain alpha-keto acid	*Pseudomonas cissicola*	WP_078590197									

	dehydrogenase subunit E2											
121	Dodecin domain-containing protein	*Halomonas smyrnensis*	WP_026068441									
122	Nitrogen metabolism transcriptional regulator, NtrC, Fis family	*Paraburkholderia tuberum*	SDQ32891									
123	PadR family transcriptional regulator	*Paenibacillus* sp. IHBB 10380	WP_044875678									
124	50S ribosomal protein L23	*Lactobacillus plantarum*	KRL35237									
**TOTAL**				**30**	**8**	**28**	**3**	**37**	**31**	**16**	**14**	**0**

Table S3. Identification of total proteins isolated from Mount Pond Glacier

Spot Number	Protein	NCBI Accession N°	Th[a] Mw	Th pI	Score[b]	N	Microorganism	Taxon	Domain	Genus found by 16S rRNA sequencing	Phylum found by 16S rRNA sequencing	Genus found by proteomics
**Energy metabolism**												
1	Isocitrate dehydrogenase	WP_003219599	46	5.5	102	16/76	*Pseudomonas*	Gammaproteobacteria	Bacteria	+	+	-
2	Succinate-CoA ligase subunit beta	WP_003179235	42	5.7	115	18/88	*Pseudomonas*	Gammaproteobacteria	Bacteria	+	+	-
3	Isovaleryl-CoA dehydrogenase	WP_007904074	43	5.6	96	14/75	*Pseudomonas*	Gammaproteobacteria	Bacteria	+	+	-
8	Glucosamine-fructose-6-phosphate aminotransferase	WP_012184269	68	5.4	66	15/144	*Salinispora arenicola*	Actinobacteria	Bacteria	-	+	+
9	Glycerol kinase	WP_013315890	56	5.8	80	11/54	*Dickeya dadantii*	Gammaproteobacteria	Bacteria	-	+	+
10	ATPase	WP_008153474	52	5.4	98	12/42	*Pseudomonas* sp.	Gammaproteobacteria	Bacteria	+	+	-
14	Succinate-CoA ligase subunit alpha	WP_003210566	30	5.9	96	12/49	*Pseudomonas*	Gammaproteobacteria	Bacteria	+	+	-
16	Malonyl-CoA O-methyltransferase	WP_011940068	31	8.1	90	11/61	*Geobacter uraniireducens* Rf4	Deltaproteobacteria	Bacteria	+	+	-
19	ATP synthase subunit beta	WP_007903035	49	4.9	259	29/62	*Pseudomonas* sp.	Gammaproteobacteria	Bacteria	+	+	-
26	6-phosphogluconate dehydrogenase (decarboxylating)	WP_079314207	34	4.9	77	8/30	*Microbispora* sp. GKU 823	Actinobacteria	Bacteria	+	+	-
27	Arylsulfatase A family protein	ZP_10673425	61	5.6	217	13/70	*Pseudomonas* sp. GM33	Gammaproteobacteria	Bacteria	+	+	-
29	Acetyl-CoA acyltransferase	WP_056742244	41	5.8	220	12/91	*Pseudomonas*	Gammaproteobacteria	Bacteria	+	+	-
31	ATPase	WP_007903032	52	5.4	174	11/42	*Pseudomonas*	Gammaproteobacteria	Bacteria	+	+	-
32	ATPase	WP_042557759	52	5.4	172	11/54	*Pseudomonas fluorescens*	Gammaproteobacteria	Bacteria	+	+	-
33	PhoH family protein	WP_007942274	52	5.4	110	11/42	*Pseudomonas* sp. GM21	Gammaproteobacteria	Bacteria	+	+	-
34	Succinyl-CoA	WP_008148605	30	5.9	137	8/33	*Pseudomonas* sp.	Gammaproteobacteria	Bacteria	+	+	-

	synthetase subunit alpha						Ag1					
37	ATP synthase, F1 beta subunit	WP_007903035	50	4.9	165	7/61	*Pseudomonas* sp. GM102	Gammaproteobacteria	Bacteria	+	+	-
40	Polyketide synthase	WP_003245093	48	5.5	121	16/34	*Bacillus subtilis*	Firmicutes	Bacteria	+	+	-
64	Collagenase	P33437	38	8.3	142	8/33	*Porphyromonas gingivalis*	Bacteroidetes	Bacteria	+	+	-
67	Phosphoenolpyruvate carboxykinase (ATP)	WP_009440364	61	5.3	82	9/22	*Selenomonas* sp.	Firmicutes	Bacteria	+	+	-
70	Molybdenum cofactor biosysynthesis protein	WP_030657664	30	6.5	75	7/24	*Streptomyces*	Actinobacteria	Bacteria	+	+	-
72	Succinyl-CoA synthetase subunit beta	NP_746303	41	5.8			*Pseudomonas putida* KT2440	Gammaproteobacteria	Bacteria	+	+	-
89	Acetyl-CoA carboxylase carboxyl transferase subunit alpha	OUV66025	36	5.6	81	7/15	unclassified Hyphomonadaceae	Alphaproteobacteria	Bacteria	+	+	-
92	N-acetylmuramoyl-L-alanine amidase	WP_080353948	39	7.8	98	11/37	*Bacillus cereus*	Firmicutes	Bacteria	+	+	-
94	B12-dependent methionine synthase	WP_014380026	37	4.1			*Mycobacterium intracellulare*	Actinobacteria	Bacteria	+	+	-
98	Dihydrolipoyl dehydrogenase	DLDH_PSEFL	50	6.5			*Pseudomonas fluorescens*	Gammaproteobacteria	Bacteria	+	+	-
100	Phosphohydrolase	WP_011529283	22	6.1	81	6/16	*Deinococcus geothermalis*	Deinococci	Bacteria	+	+	-
101	Glutamate carboxypeptidase	SCX73833	44	7	137	8/8	*Variovorax* sp. EL159	Betaproteobacteria	Bacteria	+	+	-
105	ATP synthase subunit beta	WP_041684542	52	4.8	133	9/9	*Renibacterium salmoninarum*	Actinobacteria	Bacteria	-	-	+
106	Acetyl-CoA carboxylase carboxyl transferase subunit alpha	WP_014421920	36	5.4	146	9/9	*Marinobacter hydrocarbonoclasticus*	Gammaproteobacteria	Bacteria	+	+	-
110	Aldehyde dehydrogenase family protein	WP_001206106	52	5.0	166	11/11	*Staphylococcus aureus*	Firmicutes	Bacteria	+	+	-
**DNA replication, restriction, binding and recombination**												

12	Recombinase	WP_004608538	42	9.2	81	11/53	*Clostridium scindens*	Firmicutes	Bacteria	+	+	-
13	DNA-binding response regulator	WP_007559895	26	8.0	83	8/34	*Methylobacterium* sp. GXF4	Alphaproteobacteria	Bacteria	+	+	-
20	DUF262 domain-containing protein	WP_016253918	45	6.1	76	10/54	*Pseudomonas aeruginosa*	Gammaproteobacteria	Bacteria	+	+	-
22	Hypothetical protein NSED_02180	WP_014964618	27	7.1	118	6/81	Candidatus *Nitrosopumilus* sp. AR2	Thaumarchaeota	Archaea	-	-	+
52	Molybdopterin-guanine dinucleotide biosynthesis protein A	KGM88408	29	8.2	70	5/14	*Roseovarius mucosus* DSM 17069	Alphaproteobacteria	Bacteria	-	-	+
55	tRNA-specific adenosine deaminase	WP_082181133	20	6.3	71	5/12	*Actinomyces timonensis*	Actinobacteria	Bacteria	+	+	-
62	Class I SAM-dependent methyltransferase	WP_067767194	30	6.1	82	8/33	*Mycobacterium* sp. E802	Actinobacteria	Bacteria	+	+	-
66	Plasmid stabilization protein	WP_088191231	9	8.2	86	7/30	*Pseudomonas* sp. A46	Gammaproteobacteria	Bacteria	+	+	-
68	DNA-binding response regulator, partial	WP_055596475	23	4.8	78	6/17	*Streptomyces hirsutus*	Actinobacteria	Bacteria	+	+	-
69	Conjugative transfer relaxase/helicase TraI, partial	WP_064349674	15	5.7	79	11/20	*Klebsiella oxytoca*	Gammaproteobacteria	Bacteria	-	-	+
102	DNA mismatch repair protein	WP_012208437	71	5.4	142	10/10	*Petrotoga mobilis*	Thermotogae	Bacteria	+	+	-
113	Helix-turn-helix domain-containing protein	WP_022703777	44	9.1	85	10/37	*Pseudorhodobacter ferrugineus*	Alphaproteobacteria	Bacteria	-	+	+
114	DNA-directed RNA polymerase subunit alpha	WP_003176403	37	4.9	142	8/8	*Pseudomonas*	Gammaproteobacteria	Bacteria	+	+	-
115	DNA ligase (NAD(+)) LigA	WP_087582529	72	5.3	85	11/36	*Campylobacter concisus*	Epsilonproteobacteria	Bacteria	+	+	-
**Ribosomal-related proteins**												

30	30S ribosomal protein S1	WP_005786304	62	4.9	181	12/67	*Pseudomonas*	Gammaproteobacteria	Bacteria	+	+	-
49	50S ribosomal protein L17	WP_065234045	15	11.0	89	7/20	*Gallibacterium sp.*	Gammaproteobacteria	Bacteria	-	+	+
60	Ribosome biogenesis GTPase Der	WP_011829587	49	8.9	108	7/19	*Methylibium petroleiphilum*	Betaproteobacteria	Bacteria	+	+	-
71	50S ribosomal protein L3	WP_012147224	22	9.9	117	6/6	*Serratia*	Gammaproteobacteria	Bacteria	+	+	-
124	50S ribosomal protein L23	KRL35237	10	9.6	75	4/4	*Lactobacillus plantarum*	Firmicutes	Bacteria	+	+	-
**Transcription**												
57	MerR family transcriptional	OEV38922	33	5.8	84	7/18	*Streptomyces aureofaciens*	Actinobacteria	Bacteria	+	+	-
82	Transcriptional regulator, IclR family	SDN17589	25	5.8	81	8/31	*Megasphaera paucivorans*	Firmicutes	Bacteria	+	+	-
122	Nitrogen metabolism transcriptional regulator, NtrC, Fis family	SDQ32891	55	5.8	74	6/13	*Paraburkholderia tuberum*	Betaproteobacteria	Bacteria	-	+	+
123	PadR family transcriptional regulator	WP_044875678	21	5.6	80	6/16	*Paenibacillus* sp. IHBB 10380	Firmicutes	Bacteria	+	+	-
83	Transcriptional regulator NrdR	WP_012816913	18	8.2	137	7/7	*Zymomonas mobilis*	Alphaproteobacteria	Bacteria	+	+	-
**Amino acid biosynthesis**												
21	O-succinylhomoserine sulfhydrylase	WP_006404010	42	5.8	81	10/47	*Burkholderia*	Betaproteobacteria	Bacteria	+	+	-
38	Glycine cleavage system T protein	WP_008146923	40	5.6	198	13/83	*Pseudomonas* sp. GM41	Gammaproteobacteria	Bacteria	+	+	-
24	Ketol-acid reductoisomerase	WP_003185459	36	5.5	83	6/13	*Pseudomonas brassicacearum*	Gammaproteobacteria	Bacteria	+	+	-
42	4-diphosphocytidyl-2-C-methyl-D-erythritol kinase	AJQ25869	32	8.8	88	8/29	*Pelosinus fermentans* JBW45	Firmicutes	Bacteria	-	+	+
112	Threonine ammonia-lyase	WP_026750379	46	5.7	104	13/46	*Sediminibacterium* sp. C3	Bacteroidetes	Bacteria	-	+	+

**Protein synthesis**												
15	Elongation factor Tu	WP_008145495	44	5.2	168	24/84	*Pseudomonas*	Gammaproteobacteria	Bacteria	+	+	-
35	Translation elongation factor TU	ZP_10666373	44	5.3	168	24/84	*Pseudomonas* sp. GM41	Gammaproteobacteria	Bacteria	+	+	-
50	Elongation factor P lysine (34) lysyltransferase	WP_004399847	37	5.0	68	6/18	*Vibrio nigripulchritudo*	Gammaproteobacteria	Bacteria	+	+	-
73	Lysine-tRNA ligase	CDE80170	61	5.1	73	9/23	*Ruminococcus* sp. CAG:353	Firmicutes	Bacteria	-	-	+
91	Elongation factor Tu	WP_046047317	44	5.3	105	11/32	*Pseudomonas*	Gammaproteobacteria	Bacteria	+	+	-
95	Elongation factor Tu	EFTU_ACTPJ	43	5.3			*Actinobacillus pleuropneumoniae*	Gammaproteobacteria	Bacteria	+	+	-
107	Protein serine/threonine phosphatase	CCZ21917	44	5.9	178	11/11	*Acetobacter* sp.	Alphaproteobacteria	Bacteria	+	+	-
**Transport**												
18	Amino acid ABC transporter substrate-binding protein	WP_019691964	36	6.4	106	12/46	*Pseudomonas fluorescens*	Gammaproteobacteria	Bacteria	+	+	-
36	Periplasmic component of amino acid ABC-type transporter/signal transduction system	ZP_10703116	37	6.5	106	12/46	*Pseudomonas* sp. GM18	Gammaproteobacteria	Bacteria	+	+	-
41	Amino acid ABC transporter substrate-binding protein	EWG99667	37	4.1	94	12/46	*Halomonas* sp. BC04	Gammaproteobacteria	Bacteria	+	+	-
44	Uvr ABC system protein C	WP_005408879	68	8.5	95	6/29	*Stenotrophomonas maltophilia* (strain K279a)	Gammaproteobacteria	Bacteria	+	+	-
53	Phosphate ABC transporter ATP-binding protein	WP_085579862	29	5.9	74	6/18	*Thalassospira mesophila*	Alphaproteobacteria	Bacteria	+	+	-
56	Branched-chain amino acid ABC transporter substrate-binding protein	WP_011058966	40	6.2			*Pseudomonas protegens* Pf-5	Gammaproteobacteria	Bacteria	+	+	-

61	Leucine ABC transporter subunit substrate-binding protein LivK	WP_011059642	40	6.1			*Pseudomonas protegens* Pf-5	Gammaproteobacteria	Bacteria	+	+	-
65	ABC transporter substrate-binding protein	WP_005243276	71	9.6	154	11/23	*Acinetobacter* sp. CIP 64.2	Gammaproteobacteria	Bacteria	+	+	-
75	ABC transporter substrate-binding protein	NP_743302	41	5.8			*Pseudomonas putida* KT2440	Gammaproteobacteria	Bacteria	+	+	-
78	Sugar ABC transporter substrate-binding protein	WP_010567089	40	6.2			*Pseudomonas extremaustralis*	Gammaproteobacteria	Bacteria	+	+	-
79	Amino acid ABC transporter substrate-binding protein	WP_047538293	33	6.9	131	13/28	*Pseudomonas* sp. 11/12A	Gammaproteobacteria	Bacteria	+	+	-
90	Glutamate/aspartate ABC transporter periplasmic glutamate/aspartate-binding protein GltI	WP_011063125	40	6.2			*Pseudomonas protegens* Pf-5	Gammaproteobacteria	Bacteria	+	+	-
**Cell envelope**												
81	Sensor histidine kinase	WP_068671481	31	6.6	84	9/38	*Thermosulfurimonas dismutans*	Thermodesulfobacteria	Bacteria	-	-	+
99	Penicillin-binding protein 2	WP_019278306	65	8.8	82	11/37	*Clostridium botulinum*	Firmicutes	Bacteria	+	+	-
108	TadE family protein	WP_007807391	24	9.9	90	11/51	*Rhodanobacter spathiphylli*	Gammaproteobacteria	Bacteria	+	+	-
111	Penicillin-binding protein 2_2e-12	WP_001249177	81	8.7	198	16/16	*Bacillus cereus*	Firmicutes	Bacteria	+	+	-
**Cell motility**												
96	Flagellar MS-ring protein	KFA35468	35	5.5	76	6/13	*Xanthomonas campestris*	Gammaproteobacteria	Bacteria	+	+	-
120	Branched-chain alpha-keto acid dehydrogenase subunit E2	WP_078590197	52	5.5	77	6/10	*Pseudomonas cissicola*	Gammaproteobacteria	Bacteria	+	+	-
**Chaperone and stress protein**												

4	Molecular chaperone GroEL	WP_007898438	57	5.0	89	14/38	*Pseudomonas*	Gammaproteobacteria	Bacteria	+	+	-
6	Molecular chaperone DnaK	WP_010463518	68	4.8	145	35/74	*Pseudomonas mandelii*	Gammaproteobacteria	Bacteria	+	+	-
17	MoxR family ATPase	WP_000892980	33	4.9	89	10/43	*Bacillus cereus*	Firmicutes	Bacteria	+	+	-
116	Tetratricopeptide repeat protein	WP_028795026	57	8.8	88	12/52	*Thalassobaculum salexigens*	Alphaproteobacteria	Bacteria	-	+	+
**Redox homeostasis**												
5	Oxidoreductase	WP_004151005	67	6.9	67	13/74	*Klebsiella pneumoniae*	Gammaproteobacteria	Bacteria	-	-	+
25	LLM class flavin-dependent oxidoreductase	WP_068047617	39	5.4	72	6/14	*Nocardia speluncae*	Actinobacteria	Bacteria	+	+	-
28	Ferritin	OGU06320	18	5.1	67	6/35	Geobacteraceae bacterium GWC2_55_20	Deltaproteobacteria bacteria	Bacteria	+	+	-
121	Dodecin domain-containing protein	WP_026068441	8	6.0	72	5/32	*Halomonas smyrnensis*	Gammaproteobacteria	Bacteria	+	+	-
**Hypothetical and unknown protein**												
7	Hypothetical protein	WP_004152656	74	5.0	66	11/67	Multispecies	Enterobacteriaceae	Bacteria	-	+	-
11	Hypothetical protein	WP_019267207	39	9.2	66	10/70	*Methanobrevibacter smithii*	Euryarchaeota	Archaea	-	+	+
23	Hypothetical protein	WP_020154942	36	6.2	75	8/31	*Caldibacillus debilis*	Firmicutes	Bacteria	-	+	+
39	Hypothetical protein	WP_080508095	51	9.5	84	10/34	*Bryobacter aggregatus*	Acidobacteria	Bacteria	-	-	+
43	Hypothetical protein HLUCCO06_06545	KPQ26449	21	10.2	73	5/12	*Halomonas* sp. HL-93	Gammaproteobacteria	Bacteria	+	+	-
45	Hypothetical protein	WP_005979176	45	4.9	150	9/29	*Ruegeria lacuscaerulensis*	Alphaproteobacteria	Bacteria	+	+	-
46	Hypothetical protein	WP_081737368	71	6.6	88	6/18	*Corynebacterium falsenii*	Actinobacteria	Bacteria	+	+	-
47	Hypothetical protein	WP_058934563	74	7.7	85	7/10	*Roseatales depolymerans*	Betaproteobacteria	Bacteria	-	+	+
48	Hypothetical protein N399_09015	EQM28295	22	5.4	75	5/11	*Bacillus licheniformis* CG-B52	Firmicutes	Bacteria	+	+	-
51	Hypothetical protein	OGP87982	22	10.0	75	7/22	Deltaproteobacteria	Deltaproteobacteria	Bacteria	-	-	+

	A2156_01595						bacterium RBG_16_48_10	bacteria				
54	Hypothetical protein A2664_03710	OHA17696	57	7.1	82	10/25	Candidatus Taylorbacteria bacterium	unclassified bacteria	Bacteria	-	-	+
58	Hypothetical protein SAMN02982985_04871	SFM66411	8	9.9	72	4/19	*Rugamonas rubra*	Gammaproteobacteria	Bacteria	-	+	+
59	Hypothetical protein	WP_088074644	8	5.7	71	4/12	*Bacillus alkalitelluris*	Firmicutes	Bacteria	+	+	-
63	Hypothetical protein L917_14538	ETL85988	85	6.2	93	10/16	*Phytophthora parasitica*	Stramenopiles	Eukarya	-	-	+
74	Hypothetical protein UV73_C0010G0061	KKS96476	21	8.6	82	6/13	Candidatus Gottesmanbacteria bacterium GW2011_GWA2_43_14	unclassified Bacteria	Bacteria	-	-	+
76	yaaT protein	CCZ54943	32	8.2	67	6/13	*Dialister invisus* CAG:218	Firmicutes	Bacteria	-	+	+
77	Hypothetical protein GL50803_115478	XP_001705438	80	4.9	75	8/15	*Giardia lamblia* ATCC 50803	Diplomonadida	Eukarya	-	-	+
80	Hypothetical protein A2Z93_11965	OGO97834	95	5.6	70	8/17	*Curvibacter* sp. GWA2_64_110	Betaproteobacteria	Bacteria	+	+	-
84	Uncharacterized protein	SHY74220	34	4.8	72	6/16	*Mycobacterium*	Actinobacteria	Bacteria	+	+	-
85	Hypothetical protein	WP_018701773					*Anaeromusa acidaminophila*	Firmicutes	Bacteria	-	-	+
86	DUF3284 domain-containing protein	WP_049149326	16	9.4	77	6/20	*Lactobacillus gasseri*	Firmicutes	Bacteria	+	+	-
87	Hypothetical protein	WP_074239494	34	8.6	87		*Chitinophaga niabensis*	Bacteroidetes	Bacteria	+	+	-
88	Hypothetical protein	WP_067770147	30	4.3	69	5/14	*Mycobacterium* sp. E802	Actinobacteria	Bacteria	+	+	-
93	Hypothetical protein	WP_086640703	17	9.0	85	7/32	*Acetobacter tropicalis*	Alphaproteobacteria	Bacteria	+	+	-
97	Hypothetical protein	WP_067156483	34	7.9	80	7/19	*Streptomyces* sp. ERV7	Actinobacteria	Bacteria	+	+	-
103	Hypothetical protein	OGI01296	117	9.3	91	16/42	Candidatus	unclassified bacteria	Bacteria	-	-	+

	A2Y25_00865						Melainabacteria bacterium					
104	Hypothetical protein	WP_057098237	76	6.1	84	9/18	*Bacteroides uniformis*	Bacteroidetes	Bacteria	+	+	-
109	Hypothetical protein BN2364_4186	CUR48627	4	7.9	99	6/23	*Alcanivorax dieselolei*	Gammaproteobacteria	Bacteria	-	+	+
117	Hypothetical protein AKJ43_00885	KXB02680	23	5.9	80	9/40	candidate divison MSBL1 archaeon SCGC-AAA261D19	unclassified Euryarchaeota	Archaea	-	+	-
118	Hypothetical protein WH7805_11873	EAR19613	46	9.5	204	12/12	*Synechococcus* sp. WH 7805	Cyanobacteria	Bacteria	-	+	+
119	Hypothetical protein OC00_16270, partial	KGT82980	25	8.6	82		*Xanthomonas vasicola*	Gammaproteobacteria	Bacteria	-	+	+
**TOTAL**										91	109	31

[a] Abbreviations used: Theoretical molecular weight (ThMw), theoretical isoelectric point (Th pI), number of mass values searched/number of mass values matched (N), % sequence coverage (C).

[b] Protein scores higher than 66 were significant ($p<0.05$) in the Mascot database search algorithm (Matrix science, UK).

Table S4. Culture conditions. Culture conditions in red color are those analyzed by proteomics.

MP1																																									
medium	T1	T2					T3					T4					T5					T6					T7					T8					T9				
T (ºC)	X	−4	0	4	20	28	−4	0	4	20	28	−4	0	4	20	28	−4	0	4	20	28	−4	0	4	20	28	−4	0	4	20	28	−4	0	4	20	28	−4	0	4	20	28

MP2																																									
medium	T1	T2					T3					T4					T5					T6					T7					T8					T9				
T (ºC)	X	−4	0	4	20	28	−4	0	4	20	28	−4	0	4	20	28	−4	0	4	20	28	−4	0	4	20	28	−4	0	4	20	28	−4	0	4	20	28	−4	0	4	20	28

MP3																																									
medium	T1	T2					T3					T4					T5					T6					T7					T8					T9				
T (ºC)	X	−4	0	4	20	28	−4	0	4	20	28	−4	0	4	20	28	−4	0	4	20	28	−4	0	4	20	28	−4	0	4	20	28	−4	0	4	20	28	−4	0	4	20	28

Samples: MP1, PM2, MP3; T1: direct sample; Culture media: T2, T3, T4, T5, T6, T7, T8, T9; Temperatures: -4, 0, 4, 20, 28ºC;

Table S5. Oligonucleotide sequences used in this study.

Primers	Sequence (5’ to 3’)*	Product length (bp)
(V3-V4) 341F	**ACACTGACGACATGGTTCTACA**CCTACGGGNGGCWGCAG	100
(V3-V4) 805R	**TACGGTAGCAGAGACTTGGTCT**GACTACHVGGGTATCTAATCC	100

*Herlemann DP, Labrenz M, Jurgens K, Bertilsson S, Waniek JJ, Andersson AF. (2011). Transitions in bacterial communities along the 2000 km salinity gradient of the Baltic Sea. *ISME J.* 5, 1571-1579.

II. Supplementary Figures

Figure S1. - Soluble proteins from glacier samples and from cultures resolved by 2-DE. Numbered spots marked with circles corresponded to proteins identified by MALDI-TOF-TOF and described in Tables S6 and S7. Gels A to I correspond to glacier samples (T1) and culture conditions (T2 to T9) respectively. The figure is representative of four 2-DE experiments.

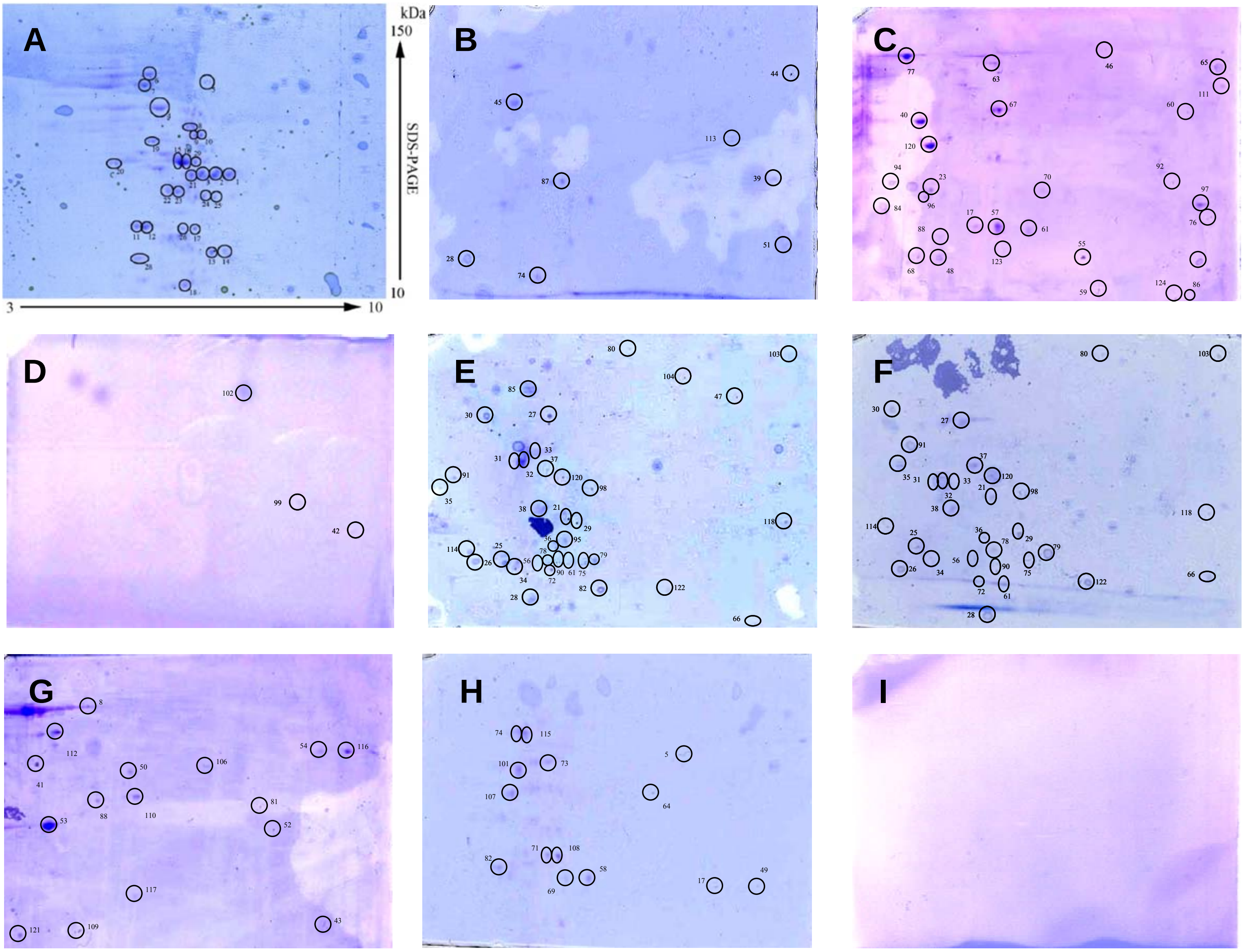

Figure S2. - Growth rates monitored by optical density at 600 nm. The data shown are the mean ±SEM of 3 cultures for each culture condition.

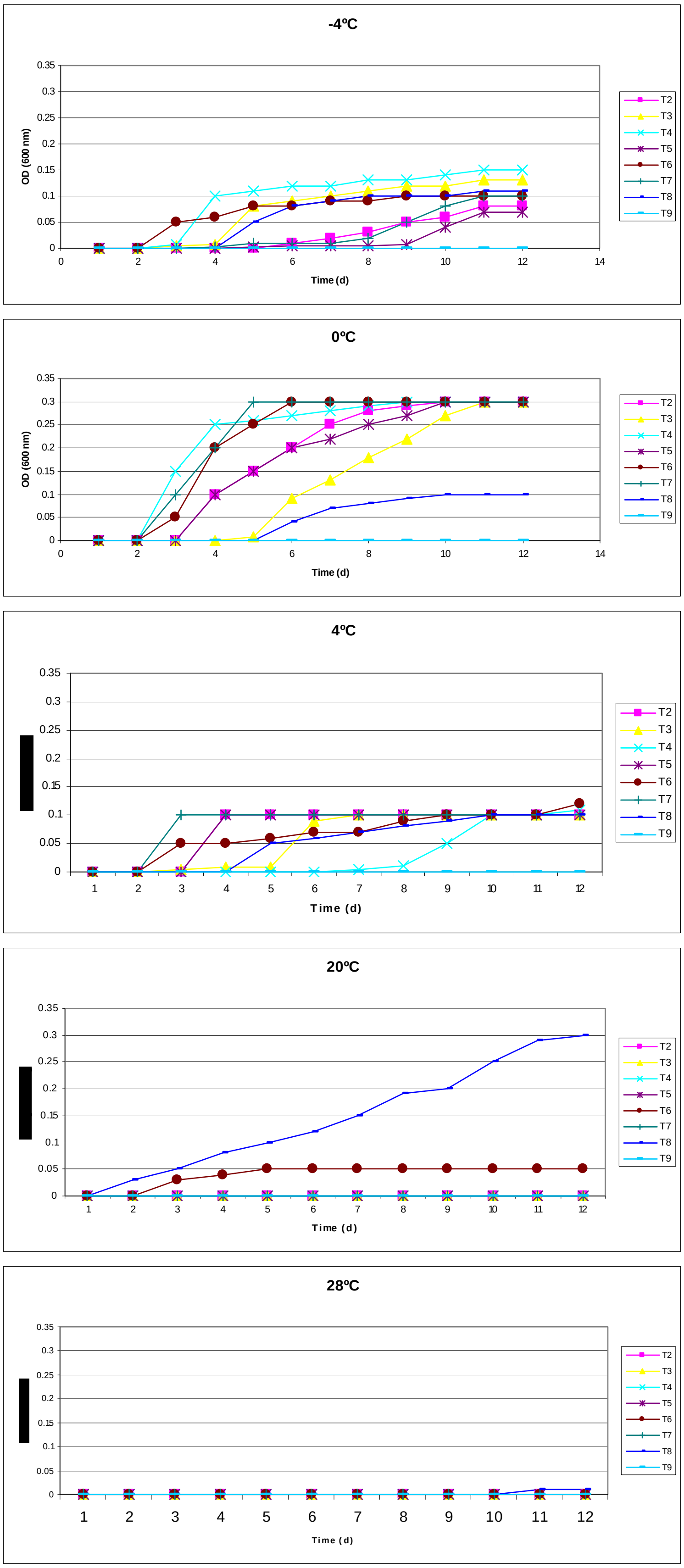

Figure S3. - Proteome-inferred microbial activity. Main pathways found by metaproteomics in key functional members of the englacial microbial community. (A) *Pseudomonas*, (B) *Halomonas*, (C) *Geobacter*, (D) *Bacillus*, (E) *Mycobacterium*, (F) *Streptomyces*.

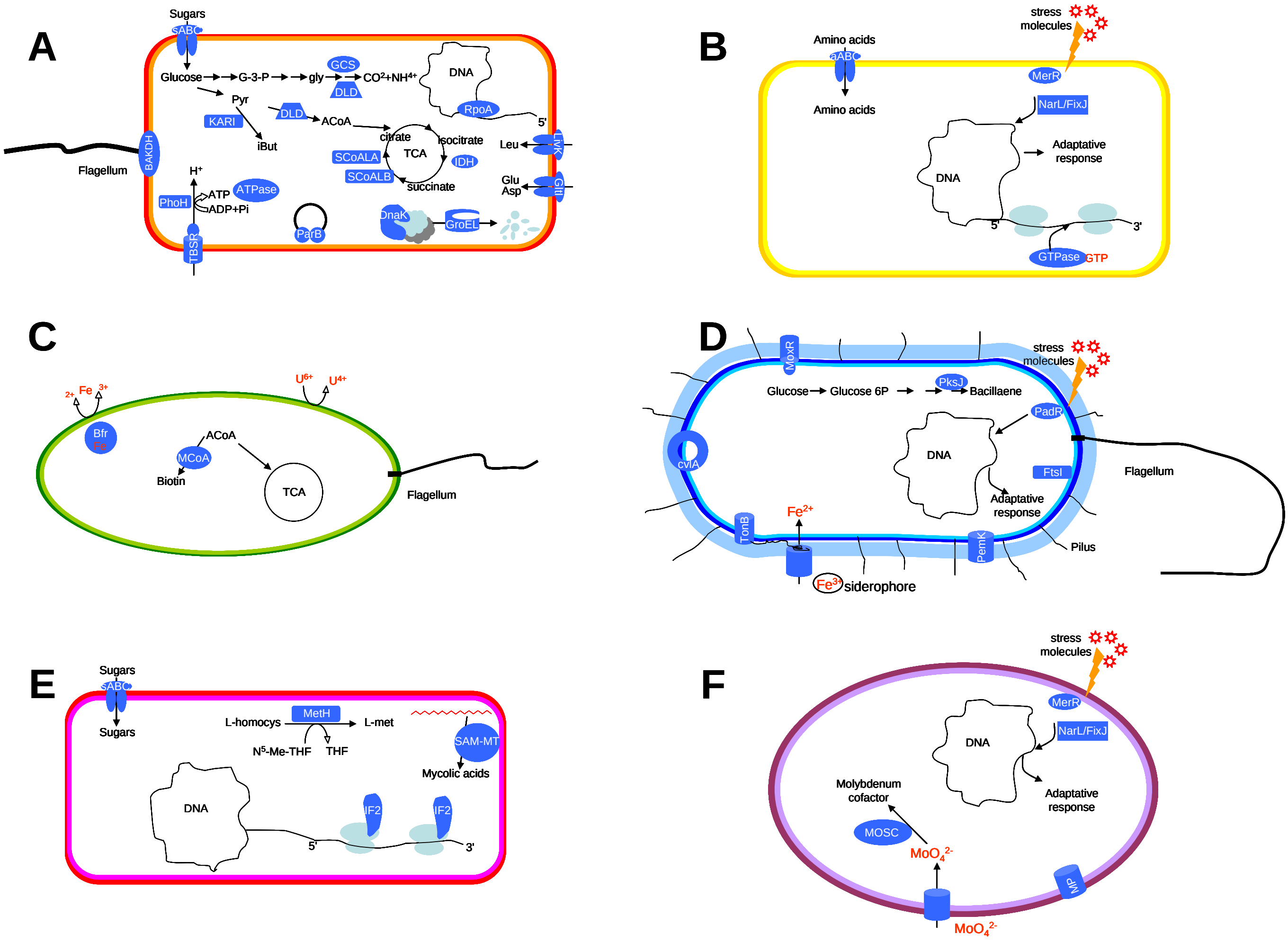

Figure S4. - Rarefaction curves determined for 16S rRNA gene clones. Rarefaction curves indicating the observed OTUs at a genetic distance of 3%.

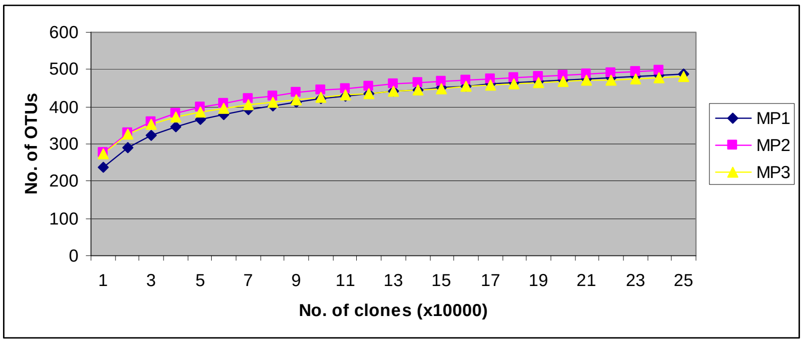

III. Mascot search results

Search Parameters
Mascot: http://www.matrixscience.com/
Enzyme: Trypsin
Fixed modifications: Carbamidomethyl (C)
Variable modifications: Oxidation (M)
Mass values: MONOISOTOPIC
Protein Mass: Unrestricted
Peptide Mass Tolerance: ± 80 ppm
Fragment Mass Tolerance: ± 0.3 Da
Max Missed Cleavages: 1
Instrument type: MALDI-TOF-TOF
Database:

Spot number: 1
Mass: 45533 Score: 102 Queries matched: 16
Isocitrate dehydrogenase [Pseudomonas sp. Ag1]

Start – End	Observed	Mr(expt)	Mr(calc)	ppm	M	Peptide
64 – 75	1440.6800	1439.6727	1439.7119	-27.2	1	R.KISWMEVYAGEK.A
64 – 75	1456.6930	1455.6857	1455.7068	-14.5	1	R.KISWMEVYAGEK.A + Oxidation (M)
76 – 95	2321.1290	2320.1217	2320.1223	-0.25	0	K.ATQVYDQDTWLPQETLDAVK.D
103 – 114	1124.6290	1123.6217	1123.6350	-11.8	0	K.GPLTTPVGGGIR.S
122 – 134	1659.8910	1658.8837	1658.8926	-5.37	0	R.QQLDLYVCLRPVR.W
135 – 144	1145.5900	1144.5827	1144.5917	-7.85	0	R.WFEGVPSPVK.K
145 – 168	2813.3500	2812.3427	2812.3378	1.76	1	K.KPGDVDMTIFRENSEDIYAGIEWK.A
190 – 209	2232.1790	2231.1717	2231.1732	-0.68	1	K.IRFDQNCGIGIKPVSLEGTK.R
192 – 209	1962.9860	1961.9787	1961.9881	-4.76	0	R.FDQNCGIGIKPVSLEGTK.R
192 – 210	2119.0810	2118.0737	2118.0892	-7.29	1	R.FDQNCGIGIKPVSLEGTKR.L
215 – 232	2086.0900	2085.0827	2085.0855	-1.31	1	K.ALQYVVDNDRDSLTIVHK.G
245 – 269	2845.3420	2844.3347	2844.3105	8.52	0	K.EWAYEVAAEEFGATLLDGGPWMQFK.N
347 – 367	2309.1550	2308.1477	2308.1555	-3.37	1	K.YAGKDQVNPGSLILSAEMMLR.H + Oxidation (M)
347 – 367	2325.1560	2324.1487	2324.1504	-0.73	1	K.YAGKDQVNPGSLILSAEMMLR.H + 2 Oxidation (M)

Start – End	Observed	Mr(expt)	Mr(calc)	ppm	M	Peptide
390 – 397	1038.4970	1037.4897	1037.4931	-3.21	0	K.TVTYDFHR.L
398 – 418	2164.1430	2163.1357	2163.0704	30.2	1	R.LMDGAKLLSSSAFGDALISHM.-

Spot number: 2
Mass: 41544 Score: 115 Queries matched: 18
Succinate-CoA ligase subunit beta [Pseudomonas]

Start – End	Observed	Mr(expt)	Mr(calc)	ppm	M	Peptide
38 – 54	1750.9440	1749.9367	1749.9275	5.29	1	K.IGGSEWVVKAQVHAGGR.G
71 – 80	1204.6660	1203.6587	1203.6513	6.20	1	K.AFAQQWLGKR.L
80 – 95	1790.9340	1789.9267	1789.9323	-3.10	1	K.RLVTYQTDANGQPVTK.I
81 – 95	1634.8010	1633.7937	1633.8312	-22.9	0	R.LVTYQTDANGQPVTK.I
96 – 116	2364.2650	2363.2577	2363.2406	7.23	1	K.ILVESCTDIAKELYLGAVVDR.S
107 – 116	1134.6210	1133.6137	1133.6081	4.99	0	K.ELYLGAVVDR.S
121 – 143	2503.2530	2502.2457	2502.2312	5.80	1	R.IVFMASTEGGVDIEKIAHDTPEK.I + Oxidation (M)
136 – 146	1264.6740	1263.6667	1263.7187	-41.1	1	K.IAHDTPEKILK.A
147 – 161	1569.8400	1568.8327	1568.8311	1.04	0	K.ATIDPLVGAQPFQGR.E
205 – 215	1214.5450	1213.5377	1213.5397	-1.66	0	K.ADGDLHCLDAK.I
216 – 225	1180.5940	1179.5867	1179.5706	13.7	0	K.INIDANAMYR.Q
216 – 225	1196.5750	1195.5677	1195.5655	1.84	0	K.INIDANAMYR.Q + Oxidation (M)
216 – 228	1533.7920	1532.7847	1532.7769	5.10	1	K.INIDANAMYRQPK.L
216 – 228	1549.7690	1548.7617	1548.7718	-6.52	1	K.INIDANAMYRQPK.L + Oxidation (M)
229 – 241	1555.7680	1554.7607	1554.7427	11.6	1	K.LKTFHDPSQDDPR.E
231 – 241	1314.5710	1313.5637	1313.5637	0.049	0	K.TFHDPSQDDPR.E
338 – 348	1194.7580	1193.7507	1193.7496	0.94	1	K.EVGVKIPVVVR.L
343 – 359	1779.0180	1778.0107	1778.0050	3.22	1	K.IPVVVRLEGNNAELGAK.V

Spot number: 3
Mass: 42542 Score: 96 Queries matched: 14
Isovaleryl-CoA dehydrogenase [Pseudomonas sp. GM79]

Start – End	Observed	Mr(expt)	Mr(calc)	ppm	M	Peptide

Start – End	Observed	Mr(expt)	Mr(calc)	ppm	M	Peptide
28 – 48	2424.2520	2423.2447	2423.2597	-6.18	1	K.EIAPRAAQIDIDNLFPADLWR.K
33 – 48	1857.9140	1856.9067	1856.9421	-19.0	0	R.AAQIDIDNLFPADLWR.K
177 – 193	1882.9970	1881.9897	1881.9949	-2.73	1	K.TDLEKGPHGITAFIVER.D
182 – 193	1296.6900	1295.6827	1295.6986	-12.3	0	K.GPHGITAFIVER.D
239 – 248	1182.5670	1181.5597	1181.5750	-13.0	0	K.VLMSGLDYER.V
290 – 301	1368.6440	1367.6367	1367.6503	-9.95	0	K.VADMYTQLNASR.A
302 – 313	1414.6690	1413.6617	1413.6711	-6.60	0	R.AYLYAVAQACER.G
302 – 318	1928.9210	1927.9137	1927.9210	-3.78	1	R.AYLYAVAQACERGETAR.K
319 – 334	1724.8440	1723.8367	1723.8563	-11.4	1	R.KDAAGVILYSAECATR.M
320 – 334	1596.7570	1595.7497	1595.7613	-7.28	0	K.DAAGVILYSAECATR.M
335 – 356	2320.1790	2319.1717	2319.1681	1.55	0	R.MALDAIQILGGNGYINEFPAGR.L
360 – 374	1622.8050	1621.7977	1621.8311	-20.6	1	R.DAKLYEIGAGTSEIR.R
363 – 375	1464.7540	1463.7467	1463.7732	-18.1	1	K.LYEIGAGTSEIRR.M
381 – 387	908.4360	907.4287	907.4399	-12.3	0	R.ELFNETR.-

Spot number: 4
Mass: 57168 Score: 89 Queries matched: 14
Molecular chaperone GroEL [Pseudomonas sp. GM102]

Start – End	Observed	Mr(expt)	Mr(calc)	ppm	M	Peptide
106 – 118	1373.6980	1372.6907	1372.6955	-3.48	1	K.AVAAGMNPMDLKR.G
106 – 118	1389.6790	1388.6717	1388.6904	-13.5	1	K.AVAAGMNPMDLKR.G + Oxidation (M)
169 – 197	3107.5470	3106.5397	3106.5129	8.65	1	K.VGKEGVITVEEGSGLENELSVVEGMQFDR.G
172 – 197	2823.3550	2822.3477	2822.3280	6.98	0	K.EGVITVEEGSGLENELSVVEGMQFDR.G
172 – 197	2839.3550	2838.3477	2838.3229	8.73	0	K.EGVITVEEGSGLENELSVVEGMQFDR.G + Oxidation (M)
328 – 345	1858.9180	1857.9107	1857.9068	2.10	0	K.ENTTVIDGAGVEADIQAR.V
351 – 364	1641.7420	1640.7347	1640.7278	4.22	1	R.QQVADTSSDYDREK.L
394 – 404	1238.6480	1237.6407	1237.6527	-9.68	1	K.ARVEDALHATR.A
396 – 404	1011.5240	1010.5167	1010.5145	2.20	0	R.VEDALHATR.A
405 – 421	1579.8870	1578.8797	1578.8730	4.28	0	R.AAVEEGVVPGGGVALVR.A

Start – End	Observed	Mr(expt)	Mr(calc)	ppm	M	Peptide
422 – 444	2510.3390	2509.3317	2509.3136	7.22	1	R.ALQAISELKGDNDDQNVGIQLLR.R
445 – 452	911.5430	910.5357	910.5348	0.99	1	R.RAVEAPLR.Q
471 – 498	2922.3180	2921.3107	2921.2848	8.89	0	K.QGSGNFGYNAATGEYGDMIEMGILDPAK.V + Oxidation (M)
471 – 498	2938.3200	2937.3127	2937.2797	11.3	0	K.QGSGNFGYNAATGEYGDMIEMGILDPAK.V + 2 Oxidation (M)

Spot number: 5
Mass: 66799 Score: 67 Queries matched: 13
Oxidoreductase [Klebsiella pneumoniae]

Start – End	Observed	Mr(expt)	Mr(calc)	ppm	M	Peptide
25 – 32	906.3750	905.3677	905.4528	-93.9	0	R.IAEMLGEK.A + Oxidation (M)
62 – 68	955.4120	954.4047	954.4667	-64.9	0	R.YCQLIMK.E
62 – 68	971.4010	970.3937	970.4616	-69.9	0	R.YCQLIMK.E + Oxidation (M)
62 – 71	1356.5790	1355.5717	1355.6577	-63.4	1	R.YCQLIMKEQK.E + Oxidation (M)
117 – 127	1302.5990	1301.5917	1301.6979	-81.6	1	R.KPPEKNLFSDK.Q
162 – 173	1432.6090	1431.6017	1431.7259	-86.7	1	K.SRQIGATYYFAR.E
164 – 184	2328.0530	2327.0457	2327.2274	-78.0	1	R.QIGATYYFAREALIDALVTGR.N
305 – 326	2342.9980	2341.9907	2342.1689	-76.1	0	R.VDIDLTHSALAAGLLCADGQFR.Q
327 – 336	1129.5240	1128.5167	1128.6139	-86.1	0	R.QIVTVEDAVR.G
441 – 449	1248.5160	1247.5087	1247.5618	-42.6	1	R.HQWRGMDFR.A + Oxidation (M)
491 – 499	1109.4760	1108.4687	1108.5553	-78.1	0	R.EFVYNPNVK.N
500 – 513	1612.8030	1611.7957	1611.9096	-70.7	1	K.NALVLKAYDIISHR.R
515 – 532	2038.0050	2036.9977	2036.9626	17.3	0	R.IEFDAGHTDIAQSFMAIR.R + Oxidation (M)

Spot number: 6
Mass: 68322 Score: 145 Queries matched: 35
Molecular chaperone DnaK [Pseudomonas mandelii]

Start – End	Observed	Mr(expt)	Mr(calc)	ppm	M	Peptide
35 – 55	2148.1000	2147.0927	2147.1110	-8.51	0	R.TTPSIIAYANDGEILVGQSAK.R
35 – 55	2148.1000	2147.0927	2147.1110	-8.51	0	R.TTPSIIAYANDGEILVGQSAK.R
35 – 56	2304.2270	2303.2197	2303.2121	3.31	1	R.TTPSIIAYANDGEILVGQSAKR.Q

Start – End	Observed	Mr(expt)	Mr(calc)	ppm	M	Peptide
35 – 56	2304.2270	2303.2197	2303.2121	3.31	1	R.TTPSIIAYANDGEILVGQSAKR.Q
57 – 71	1711.9280	1710.9207	1710.9165	2.45	1	R.QAVTNPHNTLYAVKR.L
57 – 71	1711.9280	1710.9207	1710.9165	2.45	1	R.QAVTNPHNTLYAVKR.L
125 – 151	3013.5160	3012.5087	3012.4716	12.3	1	K.KTAEDYLGEPVTEAVITVPAYFNDSQR.Q
125 – 151	3013.5160	3012.5087	3012.4716	12.3	1	K.KTAEDYLGEPVTEAVITVPAYFNDSQR.Q
126 – 151	2885.4260	2884.4187	2884.3767	14.6	0	K.TAEDYLGEPVTEAVITVPAYFNDSQR.Q
126 – 151	2885.4260	2884.4187	2884.3767	14.6	0	K.TAEDYLGEPVTEAVITVPAYFNDSQR.Q
236 – 246	1382.7430	1381.7357	1381.7493	-9.82	1	R.LIDYLVDEFKK.E
236 – 246	1382.7430	1381.7357	1381.7493	-9.82	1	R.LIDYLVDEFKK.E
303 – 315	1483.8510	1482.8437	1482.8406	2.13	1	R.AKLEALVEDLVQR.T
303 – 315	1483.8510	1482.8437	1482.8406	2.13	1	R.AKLEALVEDLVQR.T
352 – 359	940.4400	939.4327	939.5066	-78.6	0	K.LVTEFFGK.E
423 – 445	2472.2270	2471.2197	2471.2041	6.32	0	K.SQVFSTADDNQGAVTIHVLQGER.K
423 – 445	2472.2270	2471.2197	2471.2041	6.32	0	K.SQVFSTADDNQGAVTIHVLQGER.K
423 – 446	2600.3220	2599.3147	2599.2991	6.03	1	K.SQVFSTADDNQGAVTIHVLQGERK.Q
423 – 446	2600.3220	2599.3147	2599.2991	6.03	1	K.SQVFSTADDNQGAVTIHVLQGERK.Q
453 – 467	1610.8960	1609.8887	1609.8828	3.70	1	K.SLGKFDLAEIPPAPR.G
453 – 467	1610.8960	1609.8887	1609.8828	3.70	1	K.SLGKFDLAEIPPAPR.G
457 – 467	1225.6480	1224.6407	1224.6503	-7.78	0	K.FDLAEIPPAPR.G
457 – 467	1225.6480	1224.6407	1224.6503	-7.78	0	K.FDLAEIPPAPR.G
468 – 491	2536.3430	2535.3357	2535.3333	0.95	1	R.GVPQIEVTFDIDANGILHVGAKDK.A
468 – 491	2536.3430	2535.3357	2535.3333	0.95	1	R.GVPQIEVTFDIDANGILHVGAKDK.A
503 – 517	1704.8300	1703.8227	1703.8148	4.65	0	K.ANSGLSEEEIQQMIR.D
503 – 517	1704.8300	1703.8227	1703.8148	4.65	0	K.ANSGLSEEEIQQMIR.D
503 – 517	1720.8070	1719.7997	1719.8097	-5.81	0	K.ANSGLSEEEIQQMIR.D + Oxidation (M)
503 – 517	1720.8070	1719.7997	1719.8097	-5.81	0	K.ANSGLSEEEIQQMIR.D + Oxidation (M)
529 – 547	2027.0810	2026.0737	2026.0344	19.4	1	K.FEALAAARNQGDALVHSTR.K
537 – 547	1197.5940	1196.5867	1196.5898	-2.57	0	R.NQGDALVHSTR.K

Start – End	Observed	Mr(expt)	Mr(calc)	ppm	M	Peptide
537 – 547	1197.5940	1196.5867	1196.5898	-2.57	0	R.NQGDALVHSTR.K
537 – 548	1325.6570	1324.6497	1324.6848	-26.4	1	R.NQGDALVHSTRK.M
537 – 548	1325.6570	1324.6497	1324.6848	-26.4	1	R.NQGDALVHSTRK.M
549 – 562	1479.7890	1478.7817	1478.6923	60.5	1	K.MVADAGDKVTAEEK.T + Oxidation (M)

Spot number: 7

Mass: 74281 Score: 66 Queries matched: 11

Hypothetical protein KPN_pKPN4p07111 [Klebsiella pneumoniae subsp. pneumoniae MGH 78578]

Start – End	Observed	Mr(expt)	Mr(calc)	ppm	M	Peptide
18 – 28	1111.5720	1110.5647	1110.6397	-67.5	0	K.NVKPAEAAALK.E
96 – 117	2441.2030	2440.1957	2440.4053	-85.9	1	R.LAALQLLLTENRIDAGYQVIVK.R
119 – 135	1876.8940	1875.8867	1875.8996	-6.88	0	R.VSDELAVVASMVENNQR.V + Oxidation (M)
136 – 148	1426.6390	1425.6317	1425.7187	-61.0	0	R.VAMHPAEQIAGFR.K
232 – 244	1481.7660	1480.7587	1480.7886	-20.1	0	R.VITETEISVTHPR.F
379 – 400	2455.2090	2454.2017	2454.2294	-11.3	1	K.ASCGVVACLHNGELCILRGIQK.K
404 – 417	1375.7740	1374.7667	1374.6375	94.0	0	K.TPGEADAATQATSR.D
426 – 440	1580.8150	1579.8077	1579.8821	-47.1	1	K.EPDAAEGISLPLLKK.M
597 – 619	2396.1780	2395.1707	2395.2958	-52.2	1	K.NQIVEALQEAGLDSVAVEAAKLK.K
621 – 629	915.4350	914.4277	914.4821	-59.5	0	K.GDAAILAER.S
648 – 669	2242.0650	2241.0577	2240.8975	71.5	1	K.KSGSTTTPDTDADNEDNTACAA.-

Spot number: 8

Mass: 68976 Score: 66 Queries matched: 15

Glucosamine-fructose-6-phosphate aminotransferase [Salinispora arenicola CNS-205]

Start – End	Observed	Mr(expt)	Mr(calc)	ppm	M	Peptide
1 – 23	2489.3520	2488.3447	2488.3043	16.3	1	-.MCGIVGYAGERPALGIVLDGLRR.L + Oxidation (M)
57 – 87	3109.2910	3108.2837	3108.4895	-66.2	1	K.VLSERSAQDPEACGASPIGIGDGTTGIGHTR.W
109 – 124	1807.9050	1806.8977	1807.0468	-82.5	1	R.IAVIHNGIIENFAKLR.A
156 – 171	1577.8810	1576.8737	1576.7780	60.7	0	R.AAGHPDGPQLLSAGMR.V
156 – 176	2236.0880	2235.0807	2235.1001	-8.67	1	R.AAGHPDGPQLLSAGMRVVCQR.L + Oxidation (M)

Start – End	Observed	Mr(expt)	Mr(calc)	ppm	M	Peptide
208 – 226	2050.9030	2049.8957	2049.9756	-39.0	0	R.GAGENYLASDVTAFIEHTR.D
296 – 308	1507.7130	1506.7057	1506.7236	-11.8	0	R.LTESGEIMLDEVR.L + Oxidation (M)
296 – 315	2318.9690	2317.9617	2318.1424	-77.9	1	R.LTESGEIMLDEVRLSDQDLR.D
428 – 447	2286.1230	2285.1157	2285.2718	-68.3	0	K.AFLTQLVACYLIGLHLAQVR.G
451 – 470	2223.0540	2222.0467	2222.1630	-52.3	1	K.FADEVAAVVNQLHQMPGKLR.E
498 – 512	1594.8010	1593.7937	1593.9242	-81.9	1	R.HVGYPVALEGALKLK.E
513 – 528	1736.9470	1735.9397	1735.8239	66.7	0	K.ELAYMHAEGFAAGELK.H
552 – 566	1740.9570	1739.9497	1739.8988	29.2	1	R.GMLHDKVVSNIQEVR.A + Oxidation (M)
558 – 566	1043.5250	1042.5177	1042.5771	-57.0	0	K.VVSNIQEVR.A
586 – 595	1230.6830	1229.6757	1229.6557	16.3	0	R.FADHLIYVPR.T

Spot number: **9**
Mass: 56213 Score: 80 Queries matched: 11
Glycerol kinase [Dickeya dadantii 3937]

Start – End	Observed	Mr(expt)	Mr(calc)	ppm	M	Peptide
44 – 68	2771.2240	2770.2167	2770.3272	-39.9	0	K.AGWVEHDPMEIWASQSSTLVEALAK.A + Oxidation (M)
69 – 85	1687.8100	1686.8027	1686.8537	-30.2	0	K.AGISSDEVAGIGITNQR.E
159 – 174	1947.9070	1946.8997	1947.0618	-83.2	1	R.RGELLFGTIDTWLIWK.M
160 – 179	2365.2450	2364.2377	2364.2300	3.26	1	R.GELLFGTIDTWLIWKMTQGR.V
175 – 190	1835.8450	1834.8377	1834.8744	-20.0	1	K.MTQGRVHVTDYTNASR.T
199 – 213	1915.9120	1914.9047	1914.9509	-24.1	1	K.LDWDERMLEVLDIPR.A + Oxidation (M)
286 – 297	1295.6390	1294.6317	1294.6816	-38.5	0	R.HGLLTTIACGPR.G
298 – 323	2835.2910	2834.2837	2834.4603	-62.3	1	R.GEVNYALEGAVFIGGASIQWLRDELK.L
324 – 339	1787.8170	1786.8097	1786.8811	-40.0	1	K.LIGDAMDSEYFATKVK.D
392 – 409	2017.9670	2016.9597	2016.9898	-14.9	1	R.DVLDAMQADADTRLQSLR.V
497 – 503	915.4300	914.4227	914.3406	89.8	0	R.AWEEHDE.-

Spot number: **10**
Mass: 51953 Score: 98 Queries matched: 12
ATPase [Pseudomonas sp. GM41(2012)]

Start – End	Observed	Mr(expt)	Mr(calc)	ppm	M	Peptide
53 – 63	1210.5190	1209.5117	1209.5309	-15.9	0	K.SGHHSVAAECR.Q
68 – 89	2365.2340	2364.2267	2364.2536	-11.4	1	R.LIDKTLGDASPEDVELGVPIQR.G
72 – 89	1895.9350	1894.9277	1894.9636	-18.9	0	K.TLGDASPEDVELGVPIQR.G
106 – 131	2988.6330	2987.6257	2987.6192	2.19	0	R.AEPNLILPEHLNDNIIINQLIDLHAR.E
189 – 198	1197.6400	1196.6327	1196.5647	56.9	1	R.NVDTRQDHGR.T
287 – 303	1816.0000	1814.9927	1815.0288	-19.9	1	K.TILALAAAIEQTMVSKR.Y
312 – 330	2017.9640	2016.9567	2017.0004	-21.7	0	R.SVQGLDQEIGFLPGTEAEK.M
377 – 397	2530.2360	2529.2287	2529.2356	-2.70	0	R.SFQQSLILIDECQNLTPHQMK.T
377 – 397	2546.2220	2545.2147	2545.2305	-6.19	0	R.SFQQSLILIDECQNLTPHQMK.T + Oxidation (M)
409 – 436	3042.5620	3041.5547	3041.5379	5.52	0	K.VVCLGNLAQIDTPYLSATSSGLTYLTER.F
437 – 453	1925.0070	1923.9997	1924.0319	-16.7	1	R.FKDFPNGVHITLQGVPR.S
454 – 464	1232.5810	1231.5737	1231.6084	-28.2	0	R.SILAEYAESHL.-

Spot number: **11**
Mass: 39064 Score: 66 Queries matched: 11
Hypothetical protein [Methanobrevibacter smithii]

Start – End	Observed	Mr(expt)	Mr(calc)	ppm	M	Peptide
1 – 12	1357.7110	1356.7037	1356.6595	32.6	1	-.MSGKIIYDSSEK.I
79 – 92	1765.7310	1764.7237	1764.8464	-69.5	1	K.YENEMNRDLINNIK.Y
104 – 112	993.4640	992.4567	992.5443	-88.3	0	K.YGGFPILAR.K
118 – 129	1434.7570	1433.7497	1433.7548	-3.53	1	R.RLSTNIVDMIEK.V + Oxidation (M)
130 – 139	1089.5630	1088.5557	1088.5900	-31.5	0	K.VVNTDMVAIK.N
190 – 204	1653.7860	1652.7787	1652.8410	-37.7	0	K.THLIFSIEAYGTSSK.H
212 – 220	1107.5350	1106.5277	1106.5396	-10.8	0	K.YYFATSSIR.N
280 – 291	1370.7340	1369.7267	1369.7354	-6.33	1	R.DFKNPVPIEVGR.G
283 – 293	1193.6160	1192.6087	1192.6676	-49.4	1	K.NPVPIEVGRGR.K
324 – 333	1194.6190	1193.6117	1193.6557	-36.8	0	K.HNNIIYVPPK.T

Spot number: **12**
Mass: 42035 Score: 81 Queries matched: 11

Recombinase [Clostridium scindens]

Start – End	Observed	Mr(expt)	Mr(calc)	ppm	M	Peptide
11 – 26	1659.8560	1658.8487	1658.9468	-59.1	1	K.IFDLAVLGRGAASITR.I
75 – 84	1155.5080	1154.5007	1154.5833	-71.5	0	K.TYIGHSVHNK.Q
96 – 105	1390.7370	1389.7297	1389.7153	10.4	1	K.VRKPQEEWYR.V
106 – 129	2769.3320	2768.3247	2768.4344	-39.6	1	R.VENTHEAIISEEVFQKVQELIASR.R
122 – 129	915.4520	914.4447	914.5185	-80.7	0	K.VQELIASR.R
135 – 146	1292.5990	1291.5917	1291.6958	-80.6	0	R.NGMTQIFAGLIK.C
199 – 208	1267.6910	1266.6837	1266.6431	32.1	0	R.LQYWSMLAQK.D
253 – 261	1130.5660	1129.5587	1129.4498	96.4	0	K.MYEDWSAGR.I + Oxidation (M)
253 – 273	2584.2650	2583.2577	2583.1410	45.2	1	K.MYEDWSAGRITEYNFNMLSEK.Y
287 – 310	2717.1090	2716.1017	2716.3200	-80.4	1	R.QLHETMEAAVQTAADAEKWIALMK.Q + 2 Oxidation (M)
311 – 329	2187.1330	2186.1257	2186.1834	-26.4	0	K.QYVNPVELTAELLNTLIEK.I

Spot number: **13**
Mass: 26801 Score: 83 Queries matched: 8
Fis family transcriptional regulator [Methylobacterium sp. GXF4]

Start – End	Observed	Mr(expt)	Mr(calc)	ppm	M	Peptide
47 – 60	1568.8260	1567.8187	1567.8644	-29.1	0	R.VDLVILDLGLPDMR.G
61 – 69	1050.5540	1049.5467	1049.6094	-59.7	1	R.GHDLLRAIR.T
90 – 113	2610.2890	2609.2817	2609.3047	-8.80	0	K.VEALDLGADDYVTKPFGMAELLAR.L + Oxidation (M)
120 – 132	1550.7620	1549.7547	1549.8477	-60.0	0	R.HQLAVQGERPIFR.V
136 – 143	971.5730	970.5657	970.5923	-27.4	1	R.LSIDLVRR.I
144 – 153	1131.5580	1130.5507	1130.6183	-59.8	1	R.IVKVDDSEVK.L
172 – 192	2342.9980	2341.9907	2342.2053	-91.6	0	K.VLTHAQLMQAVSVSSDPQYLR.V
222 – 240	1988.0820	1987.0747	1987.0599	7.46	1	R.LRAPDDEPTLRPPGVAGAR.-

Spot number: **14**
Mass: 30313 Score: 96 Queries matched: 12
Succinyl-CoA synthetase subunit alpha [Pseudomonas sp. Ag1]

Start – End	Observed	Mr(expt)	Mr(calc)	ppm	M	Peptide

Start – End	Observed	Mr(expt)	Mr(calc)	ppm	M	Peptide
2 – 10	1017.5700	1016.5627	1016.5866	-23.5	1	M.SVLINKDTK.V
11 – 34	2610.2780	2609.2707	2609.2544	6.25	0	K.VICQGITGSQGSFHTQQAIEYGTK.M
44 – 58	1568.8210	1567.8137	1567.8359	-14.1	0	K.GGTEHLGLPVFNTVK.D
80 – 91	1220.6330	1219.6257	1219.6448	-15.7	0	K.DSILEAAFGGIK.L
110 – 134	2711.3670	2710.3597	2710.3493	3.86	1	K.VKCDELGVVLIGPNCPGVITPGECK.I
135 – 147	1369.7440	1368.7367	1368.7700	-24.3	0	K.IGIMPGHIHLPGK.V
135 – 147	1385.7470	1384.7397	1384.7649	-18.2	0	K.IGIMPGHIHLPGK.V + Oxidation (M)
135 – 153	1981.1360	1980.1287	1980.1455	-8.48	1	K.IGIMPGHIHLPGKVGIVSR.S
135 – 153	1997.1300	1996.1227	1996.1404	-8.87	1	K.IGIMPGHIHLPGKVGIVSR.S + Oxidation (M)
224 – 243	1966.1020	1965.0947	1965.1048	-5.10	0	K.AHVTKPVVSYIAGVTAPAGK.R
224 – 244	2122.2090	2121.2017	2121.2059	-1.95	1	K.AHVTKPVVSYIAGVTAPAGKR.M
257 – 272	1621.7930	1620.7857	1620.7995	-8.50	1	K.GTADEKFAALEDAGVK.T

Spot number: **15**
Mass: 43677 Score: 168 Queries matched: 24
Elongation factor Tu [Pseudomonas]

Start	–	End	Observed	Mr(expt)	Mr(calc)	ppm	M	Peptide
9	–	25	1780.9290	1779.9217	1779.9380	-9.17	0	R.TLPHVNVGTIGHVDHGK.T
9	–	34	2709.4830	2708.4757	2708.4722	1.29	1	R.TLPHVNVGTIGHVDHGKTTLTAALTR.V
26	–	34	947.5500	946.5427	946.5447	-2.13	0	K.TTLTAALTR.V
60	–	75	1800.9710	1799.9637	1799.9530	5.97	0	R.GITINTAHVEYNSLIR.H
76	–	90	1768.7810	1767.7737	1767.7787	-2.84	0	R.HYAHVDCPGHADYVK.N
91	–	117	2776.2910	2775.2837	2775.2812	0.90	0	K.NMITGAAQMDGAILVCSAADGPMPQTR.E
138	–	155	2076.9740	2075.9667	2075.9755	-4.22	0	K.ADMVDDAELLELVEMEVR.D
138	–	155	2092.9720	2091.9647	2091.9704	-2.71	0	K.ADMVDDAELLELVEMEVR.D + Oxidation (M)
138	–	155	2108.9660	2107.9587	2107.9653	-3.13	0	K.ADMVDDAELLELVEMEVR.D + 2 Oxidation (M)
195	–	208	1616.8470	1615.8397	1615.8457	-3.72	0	K.LVETLDSYIPDPVR.V
209	–	227	2163.1530	2162.1457	2162.1446	0.54	0	R.VIDKPFLMPIEDVFSISGR.G
209	–	227	2179.1420	2178.1347	2178.1395	-2.18	0	R.VIDKPFLMPIEDVFSISGR.G + Oxidation (M)

Start	–	End	Observed	Mr(expt)	Mr(calc)	ppm	M	Peptide
242	–	252	1238.7210	1237.7137	1237.7030	8.64	0	K.VQDPLEIVGLR.D
253	–	267	1745.8250	1744.8177	1744.8124	3.03	1	R.DTTVTTCTGVEMFRK.L
268	–	283	1771.9230	1770.9157	1770.9046	6.25	1	K.LLDEGRAGENCGVLLR.G
274	–	283	1088.5420	1087.5347	1087.5444	-8.91	0	R.AGENCGVLLR.G
293	–	322	3241.7350	3240.7277	3240.7143	4.15	1	R.GQVLVKPGSVKPHTTFEAEVYVLSKEEGGR.H
318	–	328	1304.6360	1303.6287	1303.6309	-1.70	1	K.EEGGRHTPFFK.G
323	–	337	1991.0150	1990.0077	1990.0002	3.77	1	R.HTPFFKGYRPQFYFR.T
329	–	337	1233.6450	1232.6377	1232.6091	23.2	0	K.GYRPQFYFR.T
338	–	361	2606.1960	2605.1887	2605.1710	6.80	0	R.TTDVTGNCELPEGVEMVMPGDNIK.M
369	–	377	1005.4960	1004.4887	1004.4961	-7.31	0	K.TIAMEDGLR.F
369	–	377	1021.4750	1020.4677	1020.4910	-22.8	0	K.TIAMEDGLR.F + Oxidation (M)
369	–	381	1508.7670	1507.7597	1507.7817	-14.6	1	K.TIAMEDGLRFAIR.E + Oxidation (M)

Spot number: **16**

Mass: 31062 Score: 90 Queries matched: 11

Type 11 methyltransferase [Geobacter uraniireducens Rf4]

Start	–	End	Observed	Mr(expt)	Mr(calc)	ppm	M	Peptide
12	–	29	2086.0930	2085.0857	2085.0028	39.8	1	K.AAFHRQAEDYDSHALVQK.R
17	–	30	1659.8850	1658.8777	1658.8012	46.1	1	R.QAEDYDSHALVQKR.V
72	–	86	1552.8090	1551.8017	1551.7538	30.9	0	R.AVGADLAFGMCLTAR.A
153	–	162	1271.5680	1270.5607	1270.6557	-74.8	1	R.TLFELKDSYR.L
199	–	219	2342.9990	2341.9917	2342.2117	-93.9	1	R.ADAELELEYHADVSALLRSLK.R
220	–	232	1249.6180	1248.6107	1248.7051	-75.6	1	K.RIGAGNAAPVAPR.G
221	–	237	1547.7920	1546.7847	1546.8692	-54.6	1	R.IGAGNAAPVAPRGLAGR.R
238	–	248	1442.6320	1441.6247	1441.6880	-43.9	1	R.RVMLEMMDVYR.S
239	–	248	1334.6790	1333.6717	1333.5716	75.1	0	R.VMLEMMDVYR.S + 3 Oxidation (M)
251	–	270	2115.1430	2114.1357	2114.0432	43.7	1	K.YANADGIPATYEVVYGAGRK.Q
271	–	280	1184.7250	1183.7177	1183.6019	97.8	1	K.QHKEVSALIC.-

Spot number: **17**

Mass: 32991 Score: 89 Queries matched: 10
MoxR family ATPase [Bacillus cereus]

Start – End	Observed	Mr(expt)	Mr(calc)	ppm	M	Peptide
1 – 21	2562.2190	2561.2117	2561.3060	-36.8	1	-.MLEQFHIHADLKQQLSTIHEK.N + Oxidation (M)
40 – 62	2425.1520	2424.1447	2424.3839	-98.6	1	K.ASDNSIIEDAITALLLGKNILLK.G
108 – 125	1894.9460	1893.9387	1893.9328	3.10	1	K.DGASEVTFVNGPLMKAMK.N
155 – 172	2192.1490	2191.1417	2190.9892	69.6	0	K.MITNPFTQEVVYGDNEYR.V + Oxidation (M)
173 – 192	2090.9680	2089.9607	2090.0895	-61.6	0	R.VIAAINEGYVGTSELNEALK.N
210 – 226	1901.9420	1900.9347	1901.0357	-53.1	1	K.ELLLSESKLNDVATIEK.F
218 – 239	2421.1490	2420.1417	2420.2773	-56.0	1	K.LNDVATIEKFVAFASDLMPLAR.D
218 – 239	2437.1350	2436.1277	2436.2723	-59.3	1	K.LNDVATIEKFVAFASDLMPLAR.D + Oxidation (M)
227 – 242	1765.7500	1764.7427	1764.8981	-88.0	1	K.FVAFASDLMPLARDGR.V
252 – 267	1791.7460	1790.7387	1790.9059	-93.4	0	R.GIIDACDLGVYIPVMR.A

Spot number: **18**
Mass: 36847 Score: 106 Queries matched: 12
Amino acid ABC transporter substrate-binding protein [Pseudomonas fluorescens]

Start – End	Observed	Mr(expt)	Mr(calc)	ppm	M	Peptide
89 – 104	1868.9070	1867.8997	1867.9098	-5.39	1	K.ERFTALQSGEIDMLSR.N + Oxidation (M)
91 – 104	1567.7800	1566.7727	1566.7712	0.99	0	R.FTALQSGEIDMLSR.N
91 – 104	1583.7630	1582.7557	1582.7661	-6.55	0	R.FTALQSGEIDMLSR.N + Oxidation (M)
113 – 137	2684.3340	2683.3267	2683.2992	10.2	1	R.DAGMGLKFPGFITYYDGVGFLANSK.L + Oxidation (M)
120 – 137	1995.9940	1994.9867	1994.9778	4.46	0	K.FPGFITYYDGVGFLANSK.L
145 – 168	2674.2920	2673.2847	2673.2592	9.54	0	K.ELDGATICIQAGTTTELNVSDYFR.A
174 – 187	1574.7160	1573.7087	1573.7148	-3.85	0	K.YTPITFDTSDESAK.S
194 – 201	937.4230	936.4157	936.4223	-6.98	0	R.CDVLTSDK.S
194 – 208	1783.8760	1782.8687	1782.8570	6.55	1	R.CDVLTSDKSQLYAQR.S
216 – 234	2111.1920	2110.1847	2110.1674	8.21	1	K.DYVVLPETISKEPLGPVVR.N
235 – 245	1287.6310	1286.6237	1286.6255	-1.38	0	R.NGDDEWLAIVR.W
318 – 343	2838.4820	2837.4747	2837.4824	-2.71	1	K.STPLEIDRGLNALWTNGGIQYAPPVR.-

Spot number: **19**
Mass: 49604 Score: 359 Queries matched: 29
ATP synthase subunit beta [Pseudomonas]

Start – End		Observed	Mr(expt)	Mr(calc)	ppm	M	Peptide
6 –	20	1668.9760	1667.9687	1667.9610	4.60	0	R.IVQIIGAVIDVEFPR.D
53 –	64	1263.6840	1262.6767	1262.6653	9.08	1	R.TIAMGSTEGLKR.G
53 –	64	1279.6600	1278.6527	1278.6602	-5.82	1	R.TIAMGSTEGLKR.G + Oxidation (M)
87 –	106	2184.0730	2183.0657	2183.0416	11.1	0	R.IMDVLGNPIDEAGPIDTEER.W
87 –	106	2200.0700	2199.0627	2199.0365	11.9	0	R.IMDVLGNPIDEAGPIDTEER.W + Oxidation (M)
107 –	131	2678.3990	2677.3917	2677.3612	11.4	0	R.WGIHRPAPTFAEQAGGNDLLETGIK.V
132 –	141	1161.6220	1160.6147	1160.6264	-10.0	0	K.VIDLVCPFAK.G
142 –	155	1203.6720	1202.6647	1202.6772	-10.4	1	K.GGKVGLFGGAGVGK.T
156 –	164	1106.5760	1105.5687	1105.5624	5.75	0	K.TVNMMELIR.N
156 –	164	1122.5570	1121.5497	1121.5573	-6.74	0	K.TVNMMELIR.N + Oxidation (M)
156 –	164	1138.5450	1137.5377	1137.5522	-12.7	0	K.TVNMMELIR.N + 2 Oxidation (M)
165 –	182	1905.9710	1904.9637	1904.9381	13.5	0	R.NIAIEHSGYSVFAGVGER.T
183 –	194	1526.6680	1525.6607	1525.6620	-0.81	1	R.TREGNDFYHEMK.D
202 –	216	1644.8290	1643.8217	1643.8090	7.76	0	K.VALVYGQMNEPPGNR.L
202 –	216	1660.8050	1659.7977	1659.8039	-3.71	0	K.VALVYGQMNEPPGNR.L + Oxidation (M)
217 –	229	1418.7920	1417.7847	1417.7963	-8.14	1	R.LRVALTGLTMAEK.F + Oxidation (M)
219 –	229	1133.5990	1132.5917	1132.6162	-21.6	0	R.VALTGLTMAEK.F
219 –	229	1149.5960	1148.5887	1148.6111	-19.5	0	R.VALTGLTMAEK.F + Oxidation (M)
230 –	246	2085.0560	2084.0487	2084.0327	7.69	1	K.FRDEGNDVLLFVDNIYR.Y
247 –	260	1450.8010	1449.7937	1449.7827	7.58	0	R.YTLAGTEVSALLGR.M
261 –	281	2308.1260	2307.1187	2307.0875	13.5	0	R.MPSAVGYQPTLAEEMGTLQER.I
261 –	281	2324.1220	2323.1147	2323.0824	13.9	0	R.MPSAVGYQPTLAEEMGTLQER.I + Oxidation (M)
261 –	281	2340.1080	2339.1007	2339.0773	10.0	0	R.MPSAVGYQPTLAEEMGTLQER.I + 2 Oxidation (M)
287 –	323	3842.9650	3841.9577	3841.9374	5.28	0	K.NGSITSIQAVYVPADDLTDPSPATTFAHLDATVVLSR.D
324 –	342	1990.0310	1989.0237	1989.0055	9.17	0	R.DIASLGIYPAVDPLDSTSR.Q
343 –	358	1841.8910	1840.8837	1840.8704	7.24	0	R.QLDPNVIGQDHYDTAR.G

Start – End	Observed	Mr(expt)	Mr(calc)	ppm	M	Peptide
359 – 366	962.5520	961.5447	961.5345	10.6	0	R.GVQYVLQR.Y
399 – 416	1931.9920	1930.9847	1930.9829	0.93	0	R.FLSQPFFVAEVFTGASGK.Y
417 – 428	1341.7280	1340.7207	1340.7340	-9.90	1	K.YVSLKDTIAGFK.G

Spot number: **20**
Mass: 44468 Score: 76 Queries matched: 10
Hypothetical protein [Pseudomonas aeruginosa]

Start – End	Observed	Mr(expt)	Mr(calc)	ppm	M	Peptide
26 – 49	2741.3800	2740.3727	2740.4072	-12.6	1	K.SSISDAVLFNTDWTVETIFRQIAK.G
62 – 68	919.4060	918.3987	918.3831	17.0	0	R.EAWNDER.K
132 – 153	2514.2500	2513.2427	2513.1922	20.1	1	K.KFSDLESNGTDDISTLENYPIR.T
159 – 172	1865.9380	1864.9307	1864.8420	47.6	0	R.NWPNEDYLYSVFYR.L
173 – 187	1638.8630	1637.8557	1637.9100	-33.2	1	R.LNSGSLPLSPQELRK.A
210 – 219	1087.4900	1086.4827	1086.5822	-91.5	0	K.SIFGAKPDPR.M
229 – 244	1985.0940	1984.0867	1983.9254	81.3	0	R.YVAFDLFYEQYDGNLK.R
272 – 289	2033.9980	2032.9907	2033.0582	-33.2	1	K.LDNALTLTHKVFGENSFK.K
332 – 341	1326.6130	1325.6057	1325.5782	20.7	1	K.TTCNQDERFR.N
368 – 379	1430.6590	1429.6517	1429.7347	-58.0	1	K.ILGKNLDQENMR.L

Spot number: **21**
Mass: 42957 Score: 81 Queries matched: 10
O-succinylhomoserine sulfhydrylase [Burkholderia multivorans ATCC 17616]

Start – End	Observed	Mr(expt)	Mr(calc)	ppm	M	Peptide
1 – 13	1512.8190	1511.8117	1511.6926	78.8	0	-.MDDSLNFDTLAVR.A + Oxidation (M)
1 – 18	1994.8550	1993.8477	1993.9891	-70.9	1	-.MDDSLNFDTLAVRAGTLR.T
196 – 207	1244.6300	1243.6227	1243.6231	-0.28	0	K.LGADVVMHSATK.F + Oxidation (M)
196 – 214	2018.0040	2016.9967	2017.0051	-4.17	1	K.LGADVVMHSATKFLDGQGR.V + Oxidation (M)
208 – 224	1673.7850	1672.7777	1672.9261	-88.7	1	K.FLDGQGRVLGGALVGSK.A
215 – 230	1563.9350	1562.9277	1562.8854	27.1	1	R.VLGGALVGSKAFIMGK.V + Oxidation (M)
225 – 236	1427.7860	1426.7787	1426.7795	-0.56	1	K.AFIMGKVFPFVR.S + Oxidation (M)

Start – End	Observed	Mr(expt)	Mr(calc)	ppm	M	Peptide
284 – 299	1851.8090	1850.8017	1850.9315	-70.1	0	R.VFYPGLESHPQHELAK.R
346 – 358	1407.6440	1406.6367	1406.7266	-63.9	0	R.TTITHPATTTHAR.I
376 – 392	1796.8020	1795.7947	1795.9540	-88.7	1	R.LAVGLENAGDLRNDLAR.G

Spot number: **22**
Mass: 26982 Score: 118 Queries matched: 6
Hypothetical protein NSED_02180 [Candidatus Nitrosopumilus sp. AR2]

Start – End	Observed	Mr(expt)	Mr(calc)	ppm	M	Peptide
2 – 17	1867.8800	1866.8727	1866.9615	-47.5	1	M.EGEFDVFEALLKTLEK.N
34 – 47	1621.7780	1620.7707	1620.8471	-47.1	1	K.YISNSQGRIVDLEK.I
110 – 125	1894.9180	1893.9107	1893.9472	-19.3	0	R.AILYPTNPYQTSEEIR.R
145 – 169	2598.1390	2597.1317	2597.3492	-83.7	1	R.LMPARIGTSGVAIACAGIEPVLDMR.A
222 – 238	1951.8360	1950.8287	1950.9179	-45.7	0	K.ISPSEMAISPDQCVYVR.G
222 – 246	2717.0670	2716.0597	2716.3200	-95.8	1	K.ISPSEMAISPDQCVYVRGLSNPPSI.-

Spot number: **23**
Mass: 36465 Score: 75 Queries matched: 8
Hypothetical protein [Caldibacillus debilis]

Start – End	Observed	Mr(expt)	Mr(calc)	ppm	M	Peptide
1 – 21	2342.9470	2341.9397	2342.1187	-76.4	0	-.MNIGVISFAHMHAYSYASALK.K + 2 Oxidation (M)
41 – 52	1329.5550	1328.5477	1328.6183	-53.1	1	K.KAAEMFGAAYDR.D
103 – 110	965.5170	964.5097	964.4358	76.7	0	R.AMIDVCEK.N
133 – 146	1728.8070	1727.7997	1727.9141	-66.2	1	K.MIQEHRFGEIIAIR.T + Oxidation (M)
163 – 181	2073.9070	2072.8997	2072.9772	-37.4	0	R.YSGGGAVIDHTVHMVDIMR.W + Oxidation (M)
163 – 186	2705.1010	2704.0937	2704.3254	-85.7	1	R.YSGGGAVIDHTVHMVDIMRWYLGK.E
229 – 248	2233.0430	2232.0357	2232.1175	-36.6	1	R.NKNFPTWGDVTIEVLGTEGR.A
284 – 290	923.4090	922.4017	922.4331	-34.0	0	R.DFVQCVR.E

Spot number: **24**
Mass: 36414 Score: 83 Queries matched: 6
Ketol-acid reductoisomerase [Pseudomonas brassicacearum subsp. brassicacearum NFM421]

Start – End	Observed	Mr(expt)	Mr(calc)	ppm	M	Peptide
19 – 47	2985.4250	2984.4177	2984.5138	-32.2	1	K.VAIIGYGSQGHAQACNLKDSGVDVTVGLR.K
99 – 119	2313.1440	2312.1367	2312.2178	-35.1	1	K.KGATLAFSHGFAIHYNQVVPR.A
100 – 119	2185.0550	2184.0477	2184.1229	-34.4	0	K.GATLAFSHGFAIHYNQVVPR.A
162 – 175	1291.6250	1290.6177	1290.6680	-39.0	0	K.NVALSYAAGVGGGR.T
208 – 232	2891.2290	2890.2217	2890.3193	-33.8	0	K.AGFETLVEAGYAPEMAYFECLHELK.L + Oxidation (M)
307 – 321	1634.7910	1633.7837	1633.8536	-42.8	0	R.NNAAHGIEIIGEQLR.S

Spot number: **25**
Mass: 38874 Score: 72 Queries matched: 6
LLM class flavin-dependent oxidoreductase [Nocardia speluncae]

Start	–	End	Observed	Mr(expt)	Mr(calc)	ppm	M	Peptide
141	–	146	800.4000	799.3927	799.4341	-51.7	0	K.IDLFHR.L
147	–	164	2028.9450	2027.9377	2028.0640	-62.3	1	R.LLDETPVTWEGTTRAALR.D
165	–	171	807.4170	806.4097	806.3810	35.6	0	R.DADVYPK.T
177	–	195	2012.9480	2011.9407	2012.0803	-69.4	1	R.LNTWVGVGGTPESVLRTAR.Y
193	–	199	870.5350	869.5277	869.4508	88.5	1	R.TARYGFR.L
328	–	339	1304.7120	1303.7047	1303.7136	-6.80	0	R.SVELYGTAVIPR.V

Spot number: **26**
Mass: 33620 Score: 77 Queries matched: 8
6-phosphogluconate dehydrogenase (decarboxylating) [Microbispora sp. GKU 823]

Start	–	End	Observed	Mr(expt)	Mr(calc)	ppm	M	Peptide
1	–	10	1079.5610	1078.5537	1078.5515	2.08	0	-.MQIGMIGLGK.M + 2 Oxidation (M)
2	–	18	1794.8030	1793.7957	1793.8586	-35.1	1	M.QIGMIGLGKMGGNMAER.L + 2 Oxidation (M)
11	–	18	881.2770	880.2697	880.3531	-94.7	0	K.MGGNMAER.L + Oxidation (M)
11	–	20	1134.6230	1133.6157	1133.5433	63.8	1	K.MGGNMAERLR.R
43	–	52	1196.5630	1195.5557	1195.6673	-93.3	1	K.DLVERLQAPR.A
66	–	72	832.4650	831.4577	831.4450	15.3	0	R.STVEQLR.D
267	–	274	879.3730	878.3657	878.3804	-16.7	0	R.QADSPSMK.M + Oxidation (M)
267	–	280	1536.7890	1535.7817	1535.7436	24.8	1	R.QADSPSMKMVAALR.N + 2 Oxidation (M)

Spot number: **27**
Mass: 59528 Score: 217 Queries matched: 13
Arylsulfatase [Pseudomonas sp. GM33]

Start	–	End	Observed	Mr(expt)	Mr(calc)	ppm	M	Peptide
170	–	177	1010.5030	1009.4957	1009.5266	-30.6	1	R.EMFQKVTK.G
247	–	260	1644.8220	1643.8147	1643.8242	-5.78	0	K.VHQPNMPAPEFIHK.S
265	–	280	1803.9300	1802.9227	1802.9163	3.59	1	K.SKYADSIVELDTHIGR.I
267	–	280	1588.8020	1587.7947	1587.7893	3.43	0	K.YADSIVELDTHIGR.I
287	–	318	3560.7360	3559.7287	3559.7260	0.77	1	K.ALGLDKNTLVVYTTDNGAWQDVYPDAGYTPFR.G
293	–	318	2963.4070	2962.3997	2962.3774	7.54	0	K.NTLVVYTTDNGAWQDVYPDAGYTPFR.G
326	–	340	1608.8450	1607.8377	1607.8420	-2.65	1	R.EGGNRVPAIAVWPDK.I
347	–	371	2594.2720	2593.2647	2593.3938	-49.8	1	K.NHEILGGLDLMATFAAVAGVKLPEK.D
374	–	397	2578.2730	2577.2657	2577.2421	9.16	0	R.EGQPIIFDSYDMTPVLTGSAPSPR.K
398	–	414	2044.9900	2043.9827	2043.9690	6.71	1	R.KEWFYFTENELSPGAAR.V
399	–	414	1916.8820	1915.8747	1915.8741	0.35	0	K.EWFYFTENELSPGAAR.V
449	–	466	2191.0890	2190.0817	2190.0746	3.26	0	K.YVATVPQVFDLWQDPQER.Y
467	–	477	1449.6480	1448.6407	1448.6394	0.89	0	R.YDLFMNNFTER.T

Spot number: **28**
Mass: 18018 Score: 67 Queries matched: 6
Ferritin [Geobacteraceae bacterium GWC2_55_20]

Start	–	End	Observed	Mr(expt)	Mr(calc)	ppm	M	Peptide
20	–	26	913.3230	912.3157	912.3647	-53.7	0	K.MEEEGFR.T + Oxidation (M)
20	–	33	1717.7570	1716.7497	1716.8141	-37.5	1	K.MEEEGFRTYLSAIR.M + Oxidation (M)
27	–	38	1410.6820	1409.6747	1409.7700	-67.6	1	R.TYLSAIRMLTNK.G
99	–	117	2157.0760	2156.0687	2156.0612	3.49	1	R.EAMVYAIHLEKGAVDFYGK.V + Oxidation (M)
137	–	157	2635.3720	2634.3647	2634.1656	75.6	1	K.EESRHLQALEDLYEQHFMTEN.- + Oxidation (M)
141	–	157	2117.9430	2116.9357	2116.9524	-7.87	0	R.HLQALEDLYEQHFMTEN.-

Spot number: **29**
Mass: 40611 Score: 220 Queries matched: 12

Acetyl-CoA C-acyltransferase [Pseudomonas sp.]

Start – End	Observed	Mr(expt)	Mr(calc)	ppm	M	Peptide
12 – 35	2314.2830	2313.2757	2313.2692	2.81	0	R.TAIGSFQGALSAIPATELGAAVIR.R
37 – 67	3180.6640	3179.6567	3179.6310	8.09	0	R.LLEETGLDGSQIDEVILGQVLTAGSGQNPAR.Q
94 – 103	1068.6520	1067.6447	1067.6451	-0.36	0	K.AVQLAVQAIR.C
168 – 185	2056.9730	2055.9657	2055.9497	7.78	1	K.YELSREDQDAFAAASQQK.A
173 – 196	2501.2890	2500.2817	2500.1870	37.9	1	R.EDQDAFAAASQQKAAAAIEAGYFK.R
186 – 197	1267.6920	1266.6847	1266.6720	10.0	1	K.AAAAIEAGYFKR.E
198 – 207	1179.6690	1178.6617	1178.7023	-34.4	0	R.EITPILIPQR.K
337 – 356	1830.9920	1829.9847	1829.9860	-0.71	0	K.VNVNGGAIALGHPIGASGAR.I
357 – 367	1305.8140	1304.8067	1304.8180	-8.62	0	R.ILVSLLHELIR.R
357 – 368	1461.9300	1460.9227	1460.9191	2.49	1	R.ILVSLLHELIRR.D
372 – 391	2028.1110	2027.1037	2027.1198	-7.91	1	K.KGLATLCIGGGQGVSLVIER.-
373 – 391	1900.0400	1899.0327	1899.0248	4.17	0	K.GLATLCIGGGQGVSLVIER.-

Spot number: **30**

Mass: 62200 Score: 181 Queries matched: 12

30S ribosomal protein S1 [Pseudomonas]

Start – End	Observed	Mr(expt)	Mr(calc)	ppm	M	Peptide
131 – 144	1525.8770	1524.8697	1524.8777	-5.20	0	R.AFLPGSLVDVRPVR.D
145 – 157	1546.7580	1545.7507	1545.7675	-10.8	1	R.DTTHLEGKELEFK.V
174 – 198	2757.4200	2756.4127	2756.3828	10.9	1	R.SVLEAENSAEREALLESLQEGQQVK.G
230 – 246	1892.0010	1890.9937	1891.0051	-6.01	1	R.IKHPSEIVNVGDEIDVK.V
274 – 281	979.4710	978.4637	978.4883	-25.1	1	K.ARYPESTR.V
323 – 341	2173.0770	2172.0697	2172.0620	3.54	0	K.VVQVGDEVEVMVLDIDEER.R
353 – 368	1855.8170	1854.8097	1854.8173	-4.08	1	K.SNPWEDFSGQFNKGDK.I
437 – 457	2396.1780	2395.1707	2395.1543	6.85	1	K.QLESDPFSEYVQENDKGAIVK.G
467 – 481	1543.8330	1542.8257	1542.8505	-16.0	0	K.GAIITLADDIEATLK.A
467 – 487	2187.1760	2186.1687	2186.1794	-4.88	1	K.GAIITLADDIEATLKASEISR.D
531 – 554	2471.2050	2470.1977	2470.2737	-30.7	1	K.EAIQSLKEAAPEAAADTTMAALLR.Q

Start	–	End	Observed	Mr(expt)	Mr(calc)	ppm	M	Peptide
531	–	554	2487.2020	2486.1947	2486.2686	-29.7	1	K.EAIQSLKEAAPEAAADTTMAALLR.Q + Oxidation (M)

Spot number: **31**
Mass: 55473 Score: 196 Queries matched: 14
F0F1 ATP synthase subunit alpha [Pseudomonas]

Start	–	End	Observed	Mr(expt)	Mr(calc)	ppm	M	Peptide
17	–	27	1245.6770	1244.6697	1244.6725	-2.19	1	R.IDKLDVTSQAR.N
28	–	41	1431.7390	1430.7317	1430.7365	-3.37	0	R.NEGTVVSVSDGIVR.I
153	–	162	1028.5520	1027.5447	1027.5484	-3.62	0	K.AVDAMIPVGR.G
203	–	211	987.5530	986.5457	986.5509	-5.23	0	K.QSTIANVVR.K
252	–	266	1708.8250	1707.8177	1707.8315	-8.08	1	R.DRGEDALIVYDDLSK.Q
285	–	297	1553.7350	1552.7277	1552.7310	-2.13	0	R.EAYPGDVFYLHSR.L
403	–	419	1854.8860	1853.8787	1853.8795	-0.44	0	R.ELAAFAQFASDLDEATR.K
403	–	420	1982.9740	1981.9667	1981.9745	-3.92	1	R.ELAAFAQFASDLDEATRK.Q
420	–	427	995.5260	994.5187	994.5308	-12.1	1	R.KQLEHGQR.V
436	–	454	2100.9920	2099.9847	2100.0019	-8.20	0	K.QYAPMSIADMALSLYAAER.G
436	–	454	2116.9880	2115.9807	2115.9969	-7.62	0	K.QYAPMSIADMALSLYAAER.G + Oxidation (M)
436	–	454	2132.9790	2131.9717	2131.9918	-9.41	0	K.QYAPMSIADMALSLYAAER.G + 2 Oxidation (M)
465	–	478	1612.8310	1611.8237	1611.8409	-10.7	0	K.IGSFEQALIAFFNR.D
487	–	502	1765.7310	1764.7237	1764.8352	-63.2	1	K.INVKGDFNDEIDAGMK.A

Spot number: **32**
Mass: 51803 Score: 172 Queries matched: 11
ATPase [Pseudomonas fluorescens]

Start	–	End	Observed	Mr(expt)	Mr(calc)	ppm	M	Peptide
52	–	62	1210.5460	1209.5387	1209.5309	6.47	0	K.SGHHSVAAECR.Q
67	–	88	2365.2450	2364.2377	2364.2536	-6.73	1	R.LIDKTLGDASPEDVELGVPIQR.G
71	–	88	1895.9400	1894.9327	1894.9636	-16.3	0	K.TLGDASPEDVELGVPIQR.G
95	–	103	977.5260	976.5187	976.5627	-45.0	0	K.GLLSILMSK.Q + Oxidation (M)
286	–	302	1816.0010	1814.9937	1815.0288	-19.3	1	K.TILALAAAIEQTMVSKR.Y

Start	–	End	Observed	Mr(expt)	Mr(calc)	ppm	M	Peptide
311	–	329	2017.9670	2016.9597	2017.0004	-20.2	0	R.SVQGLDQEIGFLPGTEAEK.M
376	–	396	2530.2260	2529.2187	2529.2356	-6.66	0	R.SFQQSLILIDECQNLTPHQMK.T
376	–	396	2546.2260	2545.2187	2545.2305	-4.62	0	R.SFQQSLILIDECQNLTPHQMK.T + Oxidation (M)
408	–	435	3042.5590	3041.5517	3041.5379	4.53	0	K.VVCLGNLAQIDTPYLSATSSGLTYLTER.F
436	–	452	1925.0070	1923.9997	1924.0319	-16.7	1	R.FKDFPNGVHITLQGVPR.S
453	–	463	1232.5900	1231.5827	1231.6084	-20.9	0	R.SILAEYAESHL.-

Spot number: **33**
Mass: 51883 Score: 178 Queries matched: 11
PhoH family protein [Pseudomonas sp. GM21]

Start	–	End	Observed	Mr(expt)	Mr(calc)	ppm	M	Peptide
53	–	63	1210.5190	1209.5117	1209.5309	-15.9	0	K.SGHHSVAAECR.Q
68	–	89	2365.2340	2364.2267	2364.2536	-11.4	1	R.LIDKTLGDASPEDVELGVPIQR.G
72	–	89	1895.9350	1894.9277	1894.9636	-18.9	0	K.TLGDASPEDVELGVPIQR.G
106	–	131	2988.6330	2987.6257	2987.6192	2.19	0	R.AEPNIILPEHLNDNIIINQLIDLHAR.E
287	–	303	1816.0000	1814.9927	1815.0288	-19.9	1	K.TILALAAAIEQTMVSKR.Y
312	–	330	2017.9640	2016.9567	2017.0004	-21.7	0	R.SVQGLDQEIGFLPGTEAEK.M
377	–	397	2530.2360	2529.2287	2529.2356	-2.70	0	R.SFQQSLILIDECQNLTPHQMK.T
377	–	397	2546.2220	2545.2147	2545.2305	-6.19	0	R.SFQQSLILIDECQNLTPHQMK.T + Oxidation (M)
409	–	436	3042.5620	3041.5547	3041.5379	5.52	0	K.VVCLGNLAQIDTPYLSATSSGLTYLTER.F
437	–	453	1925.0070	1923.9997	1924.0319	-16.7	1	R.FKDFPNGVHITLQGVPR.S
454	–	464	1232.5810	1231.5737	1231.6084	-28.2	0	R.SILAEYAESHL.-

Spot number: **34**
Mass: 30327 Score: 137 Queries matched: 8
Succinate-CoA ligase subunit alpha [Pseudomonas]

Start	–	End	Observed	Mr(expt)	Mr(calc)	ppm	M	Peptide
11	–	34	2610.2890	2609.2817	2609.2544	10.5	0	K.VICQGITGSQGSFHTQQAIEYGTK.M
44	–	58	1568.8260	1567.8187	1567.8359	-10.9	0	K.GGTEHLGLPVFNTVK.D
80	–	91	1220.6350	1219.6277	1219.6448	-14.0	0	K.DSILEAAFGGIK.L

Start – End	Observed	Mr(expt)	Mr(calc)	ppm	M	Peptide
110 – 134	2711.3850	2710.3777	2710.3493	10.5	1	K.VKCDELGVVLIGPNCPGVITPGECK.I
135 – 147	1369.7750	1368.7677	1368.7700	-1.67	0	K.IGIMPGHIHLPGK.V
135 – 147	1385.7540	1384.7467	1384.7649	-13.1	0	K.IGIMPGHIHLPGK.V + Oxidation (M)
224 – 243	1966.1090	1965.1017	1965.1048	-1.54	0	K.AHVTKPVVSYIAGVTAPAGK.R
257 – 272	1621.7920	1620.7847	1620.7995	-9.12	1	K.GTADEKFAALEDAGVK.T

Spot number: **35**
Mass: 43677 Score: 168 Queries matched: 24
Elongation factor Tu [Pseudomonas sp. GM41 (2012)]

Start – End	Observed	Mr(expt)	Mr(calc)	ppm	M	Peptide
9 – 25	1780.9290	1779.9217	1779.9380	-9.17	0	R.TLPHVNVGTIGHVDHGK.T
9 – 34	2709.4830	2708.4757	2708.4722	1.29	1	R.TLPHVNVGTIGHVDHGKTTLTAALTR.V
26 – 34	947.5500	946.5427	946.5447	-2.13	0	K.TTLTAALTR.V
60 – 75	1800.9710	1799.9637	1799.9530	5.97	0	R.GITINTAHVEYNSLIR.H
76 – 90	1768.7810	1767.7737	1767.7787	-2.84	0	R.HYAHVDCPGHADYVK.N
91 – 117	2776.2910	2775.2837	2775.2812	0.90	0	K.NMITGAAQMDGAILVCSAADGPMPQTR.E
138 – 155	2076.9740	2075.9667	2075.9755	-4.22	0	K.ADMVDDAELLELVEMEVR.D
138 – 155	2092.9720	2091.9647	2091.9704	-2.71	0	K.ADMVDDAELLELVEMEVR.D + Oxidation (M)
138 – 155	2108.9660	2107.9587	2107.9653	-3.13	0	K.ADMVDDAELLELVEMEVR.D + 2 Oxidation (M)
195 – 208	1616.8470	1615.8397	1615.8457	-3.72	0	K.LVETLDSYIPDPVR.V
209 – 227	2163.1530	2162.1457	2162.1446	0.54	0	R.VIDKPFLMPIEDVFSISGR.G
209 – 227	2179.1420	2178.1347	2178.1395	-2.18	0	R.VIDKPFLMPIEDVFSISGR.G + Oxidation (M)
242 – 252	1238.7210	1237.7137	1237.7030	8.64	0	K.VQDPLEIVGLR.D
253 – 267	1745.8250	1744.8177	1744.8124	3.03	1	R.DTTVTTCTGVEMFRK.L
268 – 283	1771.9230	1770.9157	1770.9046	6.25	1	K.LLDEGRAGENCGVLLR.G
274 – 283	1088.5420	1087.5347	1087.5444	-8.91	0	R.AGENCGVLLR.G
293 – 322	3241.7350	3240.7277	3240.7143	4.15	1	R.GQVLVKPGSVKPHTTFEAEVYVLSKEEGGR.H
318 – 328	1304.6360	1303.6287	1303.6309	-1.70	1	K.EEGGRHTPFFK.G
323 – 337	1991.0150	1990.0077	1990.0002	3.77	1	R.HTPFFKGYRPQFYFR.T

Start – End	Observed	Mr(expt)	Mr(calc)	ppm	M	Peptide
329 – 337	1233.6450	1232.6377	1232.6091	23.2	0	K.GYRPQFYFR.T
338 – 361	2606.1960	2605.1887	2605.1710	6.80	0	R.TTDVTGNCELPEGVEMVMPGDNIK.M
369 – 377	1005.4960	1004.4887	1004.4961	-7.31	0	K.TIAMEDGLR.F
369 – 377	1021.4750	1020.4677	1020.4910	-22.8	0	K.TIAMEDGLR.F + Oxidation (M)
369 – 381	1508.7670	1507.7597	1507.7817	-14.6	1	K.TIAMEDGLRFAIR.E + Oxidation (M)

Spot number: **36**

Mass: 36847 Score: 106 Queries matched: 12

Amino acid ABC transporter substrate-binding protein [Pseudomonas fluorescens]

Start – End	Observed	Mr(expt)	Mr(calc)	ppm	M	Peptide
89 – 104	1868.9070	1867.8997	1867.9098	-5.39	1	K.ERFTALQSGEIDMLSR.N + Oxidation (M)
91 – 104	1567.7800	1566.7727	1566.7712	0.99	0	R.FTALQSGEIDMLSR.N
91 – 104	1583.7630	1582.7557	1582.7661	-6.55	0	R.FTALQSGEIDMLSR.N + Oxidation (M)
113 – 137	2684.3340	2683.3267	2683.2992	10.2	1	R.DAGMGLKFPGFITYYDGVGFLANSK.L + Oxidation (M)
120 – 137	1995.9940	1994.9867	1994.9778	4.46	0	K.FPGFITYYDGVGFLANSK.L
145 – 168	2674.2920	2673.2847	2673.2592	9.54	0	K.ELDGATICIQAGTTTELNVSDYFR.A
174 – 187	1574.7160	1573.7087	1573.7148	-3.85	0	K.YTPITFDTSDESAK.S
194 – 201	937.4230	936.4157	936.4223	-6.98	0	R.CDVLTSDK.S
194 – 208	1783.8760	1782.8687	1782.8570	6.55	1	R.CDVLTSDKSQLYAQR.S
216 – 234	2111.1920	2110.1847	2110.1674	8.21	1	K.DYVVLPETISKEPLGPVVR.N
235 – 245	1287.6310	1286.6237	1286.6255	-1.38	0	R.NGDDEWLAIVR.W
318 – 343	2838.4820	2837.4747	2837.4824	-2.71	1	K.STPLEIDRGLNALWTNGGIQYAPPVR.-

Spot number: **37**

Mass: 49604 Score: 165 Queries matched: 7

F0F1 ATP synthase subunit beta [Pseudomonas]

Start – End	Observed	Mr(expt)	M	Peptide
21 – 31	1206.6374	1206.6364	0	DSVPSIYNALK + Oxidation (M)
65 – 81	1629.8690	1629.8693	0	GLEVTDSGTAISVPVGK
87 – 106	2184.0480	2184.0488	0	IMDVLGNPIDEAGPIDTEER + Oxidation (M)

Start	–	End	Observed	Mr(expt)	M	Peptide
247	–	260	1450.7900	1450.7900	0	YTLAGTEVSALLGR
324	–	342	1990.0122	1990.0127	0	DIASLGIYPAVDPLDSTSR
372	–	387	1732.8670	1732.8673	0	DIIAILGMDELSEADK + Oxidation (M)

Spot number: **38**

Mass: 40381 Score: 198 Queries matched: 13

Glycine cleavage system aminomethyltransferase T [Pseudomonas sp. GM41(2012)]

Start	–	End	Observed	Mr(expt)	Mr(calc)	ppm	M	Peptide
2	–	21	2201.1690	2200.1617	2200.1964	-15.8	1	M.STEQLSKTPLHALHIELGAR.M
9	–	21	1427.7860	1426.7787	1426.8045	-18.0	0	K.TPLHALHIELGAR.M
47	–	61	1673.7850	1672.7777	1672.7991	-12.8	0	R.DQAGLFDVSHMGQIR.L
47	–	61	1689.7680	1688.7607	1688.7941	-19.7	0	R.DQAGLFDVSHMGQIR.L + Oxidation (M)
147	–	162	1563.9350	1562.9277	1562.9508	-14.8	0	R.ALLALQGPAAVTVLAR.L
170	–	177	1031.4690	1030.4617	1030.4728	-10.8	0	K.MTFMQFAR.V
170	–	177	1047.4530	1046.4457	1046.4678	-21.1	0	K.MTFMQFAR.V + Oxidation (M)
178	–	189	1392.7450	1391.7377	1391.7595	-15.7	1	R.VKLLGVDCFVSR.S
180	–	189	1165.5920	1164.5847	1164.5961	-9.81	0	K.LLGVDCFVSR.S
190	–	212	2341.0700	2340.0627	2340.0869	-10.4	0	R.SGYTGEDGYEISVPAANAEALAR.A
213	–	228	1550.8690	1549.8617	1549.8828	-13.6	0	R.ALLAEPEVAAIGLGAR.D
270	–	289	1963.9420	1962.9347	1962.9548	-10.2	0	R.AGGFPGAETVFGQQQAGVSR.K
292	–	299	911.5280	910.5207	910.5236	-3.16	0	R.VGLLPQER.T

Spot number: **39**

Mass: 50881 Score: 84 Queries matched: 10

Hypothetical protein [Bryobacter aggregatus]

Start	–	End	Observed	Mr(expt)	Mr(calc)	ppm	M	Peptide
42	–	60	2111.1770	2110.1697	2110.0365	63.2	1	K.LTQGITTEIMGDQADSKFR.G
96	–	115	1995.9800	1994.9727	1994.9190	26.9	0	K.GFAMGESGPAELASMQGALR.E + Oxidation (M)
148	–	161	1578.8830	1577.8757	1577.8024	46.5	0	K.TMAPFGGLYISHIR.S + Oxidation (M)
200	–	212	1549.8220	1548.8147	1548.7606	34.9	0	K.LMDPAIEFLNQSR.A + Oxidation (M)

Start	–	End	Observed	Mr(expt)	Mr(calc)	ppm	M	Peptide
246	–	256	1288.6220	1287.6147	1287.6207	-4.67	0	K.LWANLDNADTR.K
258	–	268	1287.6290	1286.6217	1286.6765	-42.5	1	K.RIAAEMLQNNK.G
259	–	268	1131.5770	1130.5697	1130.5753	-4.97	0	R.IAAEMLQNNK.G
317	–	333	1943.0050	1941.9977	1942.0047	-3.60	0	R.QTISTIYYTINEANIAK.Q
379	–	396	1928.0080	1927.0007	1926.9721	14.9	1	K.TIGLEDAVFKMSSSVATR.L + Oxidation (M)
389	–	396	854.3740	853.3667	853.3964	-34.7	0	K.MSSSVATR.L + Oxidation (M)

Spot number: **40**
Mass: 478457 Score: 121 Queries matched: 16
Polyketide synthase [Bacillus]

Start	–	End	Observed	Mr(expt)	Mr(calc)	ppm	M	Peptide
264	–	275	1350.6450	1349.6377	1349.6510	-9.84	1	K.SVRNMSAFHSAK.E + Oxidation (M)
930	–	949	2111.1770	2110.1697	2110.0766	44.1	0	K.ENQEAAGLASVNALNNNLIR.D
1338	–	1349	1288.6220	1287.6147	1287.6353	-16.0	1	K.RAVLCGAAEADR.T
1354	–	1376	2650.2290	2649.2217	2649.3187	-36.6	0	K.HHYPQISFVDIRPADDIEAIADK.L
1547	–	1556	1233.6050	1232.5977	1232.6666	-55.9	0	R.YQAQIVWIGR.S
1829	–	1835	900.4820	899.4747	899.4501	27.4	1	K.TREFYGK.W
1839	–	1851	1549.8220	1548.8147	1548.7936	13.6	0	R.QSSEFLLQHGYLK.K
2274	–	2296	2550.4170	2549.4097	2549.2582	59.4	1	R.QVQQHTPAESRTQSSQKPDQAAK.R
2786	–	2800	1783.8620	1782.8547	1782.8570	-1.28	1	R.IAYTLQTGREAMEER.L + Oxidation (M)
2815	–	2830	1926.0290	1925.0217	1924.9530	35.7	1	K.LNDFIENKADSLYLDR.I
2870	–	2886	1928.0080	1927.0007	1926.9298	36.8	0	K.GLSFDWGMLYGTQTPVR.I
2934	–	2943	1131.5770	1130.5697	1130.5931	-20.7	1	K.EEPATLTSKR.I
3341	–	3354	1578.8830	1577.8757	1577.7798	60.8	1	K.SSGQRFLEAEEGIR.M
3732	–	3742	1250.6710	1249.6637	1249.5431	96.5	0	R.AEMLSPNGQCK.V + Oxidation (M)
4079	–	4094	1912.9850	1911.9777	1911.9115	34.6	0	K.GYQIPWELLHDGDDVR.M
4108	–	4121	1583.7730	1582.7657	1582.7627	1.89	0	R.YWISSGTQQSEAVK.Q

Spot number: **41**
Mass: 36847 Score: 106 Queries matched: 12

Amino acid ABC transporter substrate-binding protein [Pseudomonas fluorescens]

Start – End		Observed	Mr(expt)	Mr(calc)	ppm	M	Peptide
89	– 104	1868.9070	1867.8997	1867.9098	-5.39	1	K.ERFTALQSGEIDMLSR.N + Oxidation (M)
91	– 104	1567.7800	1566.7727	1566.7712	0.99	0	R.FTALQSGEIDMLSR.N
91	– 104	1583.7630	1582.7557	1582.7661	-6.55	0	R.FTALQSGEIDMLSR.N + Oxidation (M)
113	– 137	2684.3340	2683.3267	2683.2992	10.2	1	R.DAGMGLKFPGFITYYDGVGFLANSK.L + Oxidation (M)
120	– 137	1995.9940	1994.9867	1994.9778	4.46	0	K.FPGFITYYDGVGFLANSK.L
145	– 168	2674.2920	2673.2847	2673.2592	9.54	0	K.ELDGATICIQAGTTTELNVSDYFR.A
174	– 187	1574.7160	1573.7087	1573.7148	-3.85	0	K.YTPITFDTSDESAK.S
194	– 201	937.4230	936.4157	936.4223	-6.98	0	R.CDVLTSDK.S
194	– 208	1783.8760	1782.8687	1782.8570	6.55	1	R.CDVLTSDKSQLYAQR.S
216	– 234	2111.1920	2110.1847	2110.1674	8.21	1	K.DYVVLPETISKEPLGPVVR.N
235	– 245	1287.6310	1286.6237	1286.6255	-1.38	0	R.NGDDEWLAIVR.W
318	– 343	2838.4820	2837.4747	2837.4824	-2.71	1	K.STPLEIDRGLNALWTNGGIQYAPPVR.-

Spot number: **42**
Mass: 31580 Score: 88 Queries matched: 8
4-diphosphocytidyl-2-C-methyl-D-erythritol kinase [Pelosinus fermentans JBW45]

Start	–	End	Observed	Mr(expt)	Mr(calc)	ppm	M	Peptide
1	–	11	1179.5730	1178.5657	1178.6151	-41.9	1	-.MSMLTVKGNAK.I
22	–	43	2586.2560	2585.2487	2585.2730	-9.39	1	K.REDGFHQVEMIMQAIDLADVLR.L
93	–	116	2251.1050	2250.0977	2250.2696	-76.4	1	K.KIPVAAGLAGGSTDAASVLLGLNR.L
94	–	119	2550.4000	2549.3927	2549.4329	-15.8	1	K.IPVAAGLAGGSTDAASVLLGLNRLWK.L
134	–	152	1995.9480	1994.9407	1994.9918	-25.6	1	K.LGSDVPFCIKGGTMLATGR.G + Oxidation (M)
144	–	152	879.3730	878.3657	878.4280	-70.9	0	K.GGTMLATGR.G + Oxidation (M)
201	–	213	1288.6050	1287.5977	1287.6671	-53.8	0	K.SSLDQGDLVGVAK.G
284	–	291	804.3680	803.3607	803.4137	-66.0	1	K.TVGRSEGV.-

Spot number: **43**
Mass: 21494 Score: 73 Queries matched: 5
Hypothetical protein HLUCCO06_06545 [Halomonas sp. HL-93]

Start	–	End	Observed	Mr(expt)	Mr(calc)	ppm	M	Peptide
2	–	12	1288.6050	1287.5977	1287.6670	-53.8	1	M.IESREDLDAIK.Q
6	–	12	803.3410	802.3337	802.4072	-91.6	0	R.EDLDAIK.Q
111	–	133	2251.1050	2250.0977	2250.2624	-73.2	1	K.GVTKFVPLIGSAVAGSISFTATK.V
115	–	133	1865.9140	1864.9067	1865.0299	-66.0	0	K.FVPLIGSAVAGSISFTATK.V
115	–	139	2586.2560	2585.2487	2585.3676	-46.0	1	K.FVPLIGSAVAGSISFTATKVMGNYR.R

Spot number: **44**
Mass: 67910 Score: 95 Queries matched: 6
Excinuclease ABC subunit UvrC [Stenotrophomonas maltophilia]

Start	–	End	Observed	Mr(expt)	Mr(calc)	ppm	M	Peptide
2	–	12	1117.5470	1116.5397	1116.5452	-4.86	0	M.TDVPAPAFDGK.A
2	–	26	2550.4000	2549.3927	2549.2914	39.7	1	M.TDVPAPAFDGKAFAAQLSTAPGVYR.M
60	–	76	1995.9480	1994.9407	1995.0315	-45.5	1	R.IMSMISQIVRMDVTVTR.S + Oxidation (M)
77	–	95	2111.1540	2110.1467	2110.1633	-7.86	1	R.SEAEALLLENQLIKSLSPR.Y
198	–	211	1549.7970	1548.7897	1548.8147	-16.2	1	R.AALFLEGKSDELTR.E
427	–	443	1927.9850	1926.9777	1926.8894	45.8	1	R.RFNISGIEPGDDYAAMR.Q + Oxidation (M)

Spot number: **45**
Mass: 45005 Score: 150 Queries matched: 9
Hypothetical protein [Ruegeria lacuscaerulensis]

Start	–	End	Observed	Mr(expt)	Mr(calc)	ppm	M	Peptide
45	–	51	845.3330	844.3257	844.3828	-67.5	0	R.FAEEHGR.V
52	–	59	879.3730	878.3657	878.4359	-79.8	0	R.VPQHGEGR.D
138	–	145	832.4780	831.4707	831.4086	74.7	0	K.AAEEVASR.N
214	–	229	1927.9850	1926.9777	1926.9292	25.2	1	R.LRVIFDNGTESNMLMR.S + 2 Oxidation (M)
216	–	233	2111.1540	2110.1467	2110.0299	55.4	1	R.VIFDNGTESNMLMRSLQR.A
284	–	298	1683.8300	1682.8227	1682.9025	-47.4	1	R.ELVHKIGVTNMSVEK.R
289	–	298	1077.5620	1076.5547	1076.5536	1.05	0	K.IGVTNMSVEK.R
289	–	299	1233.5940	1232.5867	1232.6547	-55.1	1	K.IGVTNMSVEKR.I
343	–	358	1925.9970	1924.9897	1925.0669	-40.1	1	R.LQIEIMDRFGRPVVPR.E

Spot number: **46**

Mass: 71242 Score: 88 Queries matched: 6

Start	–	End	Observed	Mr(expt)	M	Peptide
42	–	50	943.4850	943.4955	0	HVLGTSSSR
54	–	69	1636.7590	1636.8540	0	DALETTFLAGATGLTR
297	–	303	803.3580	803.4257	0	ASDLIER
304	–	315	1320.6520	1318.7113	1	GATEYRNVALPK
562	–	576	1679.7460	1679.8282	1	ADDMVIRGGENIHPR + Oxidation (M)
569	–	576	879.3590	879.4431	0	GGENIHPR
630	–	644	1701.7480	1702.9486	1	FSALDSIIRVSELPR

Spot number: **47**

Mass: 74458 Score: 85 Queries matched: 7

Hypothetical protein [Roseateles depolymerans]

Start	–	End	Observed	Mr(expt)	Mr(calc)	ppm	M	Peptide
6	–	25	2171.1150	2170.1077	2170.1899	-37.9	0	K.STPPHRPPPPPPVLTPQDIK.D
234	–	249	1701.7470	1700.7397	1700.8039	-37.7	0	R.HAMSTLEAELQDAAAK.G + Oxidation (M)
287	–	301	1733.7190	1732.7117	1732.8468	-77.9	1	R.TNAAAKTCAHTVWFR.F
345	–	354	1166.5620	1165.5547	1165.5952	-34.7	1	K.RHPDTSANIR.S
515	–	528	1679.7610	1678.7537	1678.7984	-26.6	0	R.DYDAAMRPLLDDIR.T + Oxidation (M)
554	–	568	1653.7440	1652.7367	1652.8747	-83.5	1	K.GRALFIHDAVNGDLR.S
638	–	648	1127.6020	1126.5947	1126.6346	-35.4	0	R.ETLGPAALSLR.R

Spot number: **48**

Mass: 21772 Score: 75 Queries matched: 5

Hypothetical protein N399_09015 [Bacillus licheniformis CG-B52]

Start	–	End	Observed	Mr(expt)	Mr(calc)	ppm	M	Peptide
8	–	30	2635.3410	2634.3337	2634.1696	62.3	1	K.ESWGNIERGTEFEAAFMFPSDSK.R
16	–	30	1679.7470	1678.7397	1678.7185	12.6	0	R.GTEFEAAFMFPSDSK.R + Oxidation (M)
31	–	40	1233.5830	1232.5757	1232.6917	-94.1	0	K.RPLSFFIPEK.S
111	–	119	1166.5510	1165.5437	1165.6131	-59.5	1	R.NKDWYSILK.D

Start	–	End	Observed	Mr(expt)	Mr(calc)	ppm	M	Peptide
120	–	133	1733.7160	1732.7087	1732.7944	-49.4	0	K.DDEHPIYTYIPNEK.F

Spot number: **49**
Mass: 14552 Score: 89 Queries matched: 7
50S ribosomal protein L17 [Gallibacterium genomosp. 3]

Start	–	End	Observed	Mr(expt)	Mr(calc)	ppm	M	Peptide
13	–	22	1233.6040	1232.5967	1232.5833	10.9	1	R.NSSHRQAMFR.N
65	–	71	832.4840	831.4767	831.5079	-37.5	1	R.LAFARVR.D
79	–	90	1448.7360	1447.7287	1447.7572	-19.6	1	K.LFNELGPRFAER.A
87	–	96	1127.6190	1126.6117	1126.5519	53.1	1	R.FAERAGGYTR.I
100	–	118	2171.1020	2170.0947	2169.9935	46.6	1	K.CGFRAGDNAPMAYIELVDR.Q + Oxidation (M)
104	–	118	1634.8460	1633.8387	1633.7770	37.8	0	R.AGDNAPMAYIELVDR.Q
119	–	129	1117.5720	1116.5647	1116.5299	31.2	0	R.QVTEEAAVAAE.-

Spot number: **50**
Mass: 36515 Score: 68 Queries matched: 6
Elongation factor P lysine (34) lysyltransferase [Vibrio nigripulchritudo]

Start	–	End	Observed	Mr(expt)	Mr(calc)	ppm	M	Peptide
25	–	32	1117.5560	1116.5487	1116.5716	-20.5	1	R.IRSFFYER.G
33	–	56	2635.3340	2634.3267	2634.3112	5.89	0	R.GVLEVDTPAMSHATVTDIHLHTFK.T + Oxidation (M)
82	–	96	1636.7510	1635.7437	1635.8766	-81.2	1	K.RLLAAGSGSIYQICK.S
235	–	240	832.4530	831.4457	831.4167	34.9	0	R.FEVYFK.G
241	–	255	1653.7410	1652.7337	1652.8158	-49.7	0	K.GIELANGFHELDNPK.E
269	–	287	2171.1080	2170.1007	2170.1092	-3.91	0	R.ETMGLEPQPIDHHLIEALK.S

Spot number: **51**
Mass: 21933 Score: 75 Queries matched: 7
Hypothetical protein A2156_01595 [Deltaproteobacteria bacterium RBG_16_48_10]

Start	–	End	Observed	Mr(expt)	Mr(calc)	ppm	M	Peptide
18	–	27	1166.5550	1165.5477	1165.5768	-24.9	0	K.LEDGGPIFYR.Q
28	–	33	803.3400	802.3327	802.4086	-94.5	1	R.QERWGK.G

Start	–	End	Observed	Mr(expt)	Mr(calc)	ppm	M	Peptide
43	–	52	1233.5850	1232.5777	1232.5972	-15.8	1	K.FRTMVPHSDK.V + Oxidation (M)
53	–	65	1448.7330	1447.7257	1447.7419	-11.2	1	K.VFGIKQATENDAR.I
99	–	113	1653.7490	1652.7417	1652.7940	-31.7	0	R.ALAVGEILHDENGCR.V
126	–	140	1636.7550	1635.7477	1635.9018	-94.2	0	R.LSVRPGLTGMTTIYK.A
152	–	159	1117.5550	1116.5477	1116.5968	-43.9	1	R.YDLFYIRK.Q

Spot number: **52**
Mass: 29031 Score: 60 Queries matched: 5
Molybdopterin-guanine dinucleotide biosynthesis protein A [Roseovarius mucosus DSM 17069]

Start	–	End	Observed	Mr(expt)	Mr(calc)	ppm	M	Peptide
2	–	16	1636.7760	1635.7687	1635.8216	-32.3	1	M.TDFASVILAGERDSR.D
14	–	20	832.4120	831.4047	831.4199	-18.2	1	R.DSRDALR.D
21	–	28	879.3170	878.3097	878.3804	-80.4	0	R.DETGVACK.A
41	–	47	842.4410	841.4337	841.5134	-94.6	1	R.RVIDALR.S
200	–	210	1320.6600	1319.6527	1319.7601	-81.4	0	R.LLGLWSLLSYR.M

Spot number: **53**
Mass: 28926 Score: 74 Queries matched: 6
Phosphate ABC transporter ATP-binding protein [Thalassospira mesophila]

Start	–	End	Observed	Mr(expt)	Mr(calc)	ppm	M	Peptide
1	–	8	943.4610	942.4537	942.4341	20.8	0	-.MTNAPHTR.G + Oxidation (M)
2	–	10	981.4490	980.4417	980.5152	-74.9	1	M.TNAPHTRGK.S
52	–	60	1166.5430	1165.5357	1165.6026	-57.4	1	K.STFLRCLNR.M
119	–	126	895.5420	894.5347	894.5035	34.9	0	R.IHGLVNSR.D
233	–	250	2117.8990	2116.8917	2117.0389	-69.5	1	K.LVEVGETDQIFTNPRDER.T
253	–	260	870.5160	869.5087	869.4396	79.6	1	K.GYITGRFG.-

Spot number: **54**
Mass: 56956 Score: 82 Queries matched: 10
Hypothetical protein A2664_03710 [Candidatus Taylorbacteria bacterium RIFCSPHIGHO2_01_FULL_46_22b]

Start	–	End	Observed	Mr(expt)	Mr(calc)	ppm	M	Peptide

Start	–	End	Observed	Mr(expt)	Mr(calc)	ppm	M	Peptide
1	–	6	808.3700	807.3627	807.3949	-39.8	1	-.MESWKK.K
159	–	164	803.3610	802.3537	802.4225	-85.7	0	R.DILEWK.K
186	–	193	832.5030	831.4957	831.4702	30.7	0	K.ILSSIGDK.K
231	–	248	2234.1360	2233.1287	2233.0395	40.0	1	K.DIMERMEHLFDIGEIPEK.I + 2 Oxidation (M)
251	–	268	2157.0980	2156.0907	2156.1187	-13.0	1	K.MLEHKPAEVEKLAEEFIK.K + Oxidation (M)
279	–	284	800.3820	799.3747	799.3972	-28.1	0	K.MIYMVK.E + Oxidation (M)
343	–	348	801.3910	800.3837	800.4504	-83.3	1	K.IRIDER.K
385	–	398	1448.7670	1447.7597	1447.7671	-5.09	1	K.DIKEISGQVGFAGK.V
418	–	436	2117.9570	2116.9497	2117.0425	-43.8	0	R.GDILVTVMTFPSYIAAMEK.A + 2 Oxidation (M)
457	–	467	1320.6730	1319.6657	1319.6941	-21.5	1	R.EMKKPCVISTK.I

Spot number: **55**
Mass: 20180 Score: 69 Queries matched: 5
tRNA-specific adenosine deaminase [Actinomyces timonensis]

Start	–	End	Observed	Mr(expt)	Mr(calc)	ppm	M	Peptide
1	–	20	2096.0820	2095.0747	2094.8888	88.8	0	-.MGDCGGGAAGLWEAWGVTER.S + Oxidation (M)
151	–	162	1304.7190	1303.7117	1303.6667	34.6	1	R.TGACGSIRDVLR.D
159	–	165	870.4950	869.4877	869.4719	18.2	1	R.DVLRDPR.A
163	–	169	865.3610	864.3537	864.4314	-89.9	1	R.DPRANHR.V
170	–	177	842.4480	841.4407	841.5022	-73.0	0	R.VEVVAGLR.A

Spot number: **56**
Branched-chain amino acid ABC transporter substrate-binding protein [Pseudomonas protegens Pf-5]

Calc. MH+	Meas. Mr	Calc. Mr	Dev.(Da)	Score	Mascot Score	Sequence
1304.721	1303.735	1303.714	0.021	18	51	GVTPVLYEGLTR
2096.092	2095.094	2095.085	0.009	101	94	AAGADVVYFGGLHPEAGPLVR

Spot number: **57**
Mass: 32940 Score: 84 Queries matched: 7
MerR family transcriptional regulator [Streptomyces viridifaciens]

Start	–	End	Observed	Mr(expt)	Mr(calc)	ppm	M	Peptide
1	–	7	832.5090	831.5017	831.4385	76.1	1	-.MGELARR.T
30	–	36	807.3910	806.3837	806.4259	-52.4	1	R.NPAGHRR.Y
68	–	87	2096.1070	2095.0997	2095.1286	-13.8	1	R.EHGLPEVAAAHAAALDVRIR.V
98	–	114	1794.8080	1793.8007	1793.9748	-97.0	1	R.TVAARGATAEELTLVHR.L
103	–	114	1296.6990	1295.6917	1295.6834	6.46	0	R.GATAEELTLVHR.L
235	–	245	1304.7320	1303.7247	1303.7248	-0.064	1	R.VGDLLLERYAR.L
263	–	268	801.3780	800.3707	800.4504	-99.6	1	R.ERLVER.L

Spot number: **58**
Mass: 8060
Score: 72 Queries matched: 7
Hypothetical protein SAMN02982985_04871 [Rugamonas rubra]

Start	–	End	Observed	Mr(expt)	Mr(calc)	ppm	M	Peptide
1	–	9	1079.5510	1078.5437	1078.5746	-28.6	1	-.MGAAFWIKR.Y
10	–	28	2140.1070	2139.0997	2139.2384	-64.8	0	R.YLLAAVPLFAILALVDYFK.G
10	–	31	2424.2260	2423.2187	2423.3980	-74.0	1	R.YLLAAVPLFAILALVDYFKGAR.T
60	–	68	870.5330	869.5257	869.4970	33.0	1	R.KEGPAALGK.Q

Spot number: **59**
Mass: 8804 Score: 71 Queries matched: 4
Hypothetical protein [Bacillus alkalitelluris]

Start	–	End	Observed	Mr(expt)	Mr(calc)	ppm	M	Peptide
37	–	43	806.4110	805.4037	805.3970	8.33	0	K.DGSITWK.S
37	–	50	1536.7770	1535.7697	1535.7468	15.0	1	K.DGSITWKSAITDDK.E
51	–	71	2665.4020	2664.3947	2664.1988	73.5	1	K.EKSFLESCVHDLMLYHVYEEH.-
53	–	71	2424.2260	2423.2187	2423.0562	67.1	0	K.SFLESCVHDLMLYHVYEEH.- + Oxidation (M)

Spot number: **60**
Mass: 49092 Score: 108Queries matched: 7
Ribosome biogenesis GTPase Der [Methylibium petroleiphilum]

Start	–	End	Observed	Mr(expt)	Mr(calc)	ppm	M	Peptide

Start	–	End	Observed	Mr(expt)	Mr(calc)	ppm	M	Peptide
16	–	24	1079.5510	1078.5437	1078.6135	-64.7	1	K.STLFNRLTK.S
71	–	77	879.3910	878.3837	878.4280	-50.4	1	K.EMAKQTR.Q + Oxidation (M)
106	–	112	808.4260	807.4187	807.4603	-51.4	1	R.YLRTAGK.K
113	–	120	856.5150	855.5077	855.5542	-54.3	1	K.KVLLAANK.A
229	–	238	1134.6060	1133.5987	1133.6081	-8.24	0	K.FELIDTAGLR.R
356	–	363	870.5330	869.5257	869.5334	-8.88	0	K.LATPVLTR.V
376	–	383	902.4960	901.4887	901.5246	-39.8	1	K.RAGAFRPK.L

Spot number: **61**
Leucine ABC transporter subunit substrate-binding protein LivK [Pseudomonas protegens Pf-5]

Calc. MH+	Meas. Mr	Calc. Mr	Dev.(Da)	Score	Mascot Score	Sequence
2424.235	2423.219	2423.227	-0.009	24	74	QANVDFVYYGGYHPELGLILR
2666.334	2666.302	2666.339	0.001	11	60	EDPSGPFVFPSYSAVEVIAG AITNAK

Spot number: **62**
Mass: 30078 Score: 82 Queries matched: 8
Class I SAM-dependent methyltransferase [Mycobacterium sp. E802]

Start	–	End	Observed	Mr(expt)	Mr(calc)	ppm	M	Peptide
1	–	7	803.3880	802.3807	802.4007	-24.9	0	-.MPVVDAR.H + Oxidation (M)
24	–	43	2140.1380	2139.1307	2139.0994	14.7	0	R.ATEAARPDGIIEDPMAITLR.E
115	–	125	1297.7690	1296.7617	1296.7514	7.99	1	R.LRLQLLPSSDR.L
175	–	195	2500.2520	2499.2447	2499.2699	-10.1	1	R.FPGGQLIFDSVPRFLSWYSQR.R
188	–	196	1242.6780	1241.6707	1241.6305	32.4	1	R.FLSWYSQRR.G
223	–	239	1794.8290	1793.8217	1793.9822	-89.4	1	R.AIPGVLAVRELEMPAGR.G + Oxidation (M)
240	–	252	1304.7420	1303.7347	1303.7612	-20.3	1	R.GKILSGAAGLVYR.S
255	–	260	807.4250	806.4177	806.4399	-27.5	1	R.SLERFR.A

Spot number: **63**
Mass: 84867 Score: 83 Queries matched: 10
Hypothetical protein L917_14538 [Phytophthora parasitica]

Start	–	End	Observed	Mr(expt)	Mr(calc)	ppm	M	Peptide
1	–	9	807.4250	806.4177	806.3956	27.4	0	-.MGGAVSSAK.Y
10	–	23	1569.8530	1568.8457	1568.7868	37.5	1	K.YGSDNTKMVVNTLK.K
94	–	104	1242.6780	1241.6707	1241.6404	24.4	1	R.KTGDYVSFVAR.D
105	–	121	2012.9670	2011.9597	2011.7921	83.3	0	R.DQLVAQMMCDDSEDAPR.T + 2 Oxidation (M)
164	–	172	993.5420	992.5347	992.4419	93.5	0	R.MTVHSAGMK.N + 2 Oxidation (M)
356	–	369	1536.8100	1535.8027	1535.8130	-6.68	0	R.VAPDAAVVLLEHMR.L + Oxidation (M)
520	–	533	1794.8290	1793.8217	1793.8631	-23.1	1	K.TRLEHIMQHYEGHK.L + Oxidation (M)
543	–	558	2096.1130	2095.1057	2095.0163	42.7	1	R.FRHHEVSLFEYIEDFK.W
737	–	745	832.5240	831.5167	831.4562	72.7	1	K.TKAAAGASR.K
747	–	753	800.4280	799.4207	799.4116	11.4	0	K.LSTSFVF.-

Spot number: **64**
Mass: 37895 Score: 142 Queries matched: 8
Collagenase [Porphyromonas]

Start	–	End	Observed	Mr(expt)	Mr(calc)	ppm	M	Peptide
1	–	10	1134.6300	1133.6227	1133.5863	32.2	1	-.MRSVIDAAQK.A + Oxidation (M)
3	–	10	831.3760	830.3687	830.4498	-97.6	0	R.SVIDAAQK.A
73	–	83	1304.7420	1303.7347	1303.6204	87.7	1	R.DHRQGHICGPK.G
105	–	118	1701.8260	1700.8187	1700.7689	29.3	0	K.CYLSLHEHNTSANR.G
105	–	126	2618.3570	2617.3497	2617.1696	68.8	1	K.CYLSLHEHNTSANRGACAQICR.R
128	–	149	2500.2520	2499.2447	2499.2203	9.78	1	R.GYTVKDSGLELDIENQYIMSPK.D
245	–	252	865.4660	864.4587	864.5069	-55.7	1	K.TYVGKGIK.Y
250	–	256	870.5690	869.5617	869.4759	98.7	1	K.GIKYFSR.L

Spot number: **65**
Mass: 71395 Score: 154 Queries matched: 11
ABC transporter substrate-binding protein [Acinetobacter sp. CIP 64.2]

Start	–	End	Observed	Mr(expt)	Mr(calc)	ppm	M	Peptide
57	–	77	2140.1380	2139.1307	2139.0266	48.7	0	K.GGILSQSSLGTFDNLNSMNGK.G
101	–	109	1079.5790	1078.5717	1078.5732	-1.41	0	R.VMYPLLAEK.V + Oxidation (M)

Start	–	End	Observed	Mr(expt)	Mr(calc)	ppm	M	Peptide
143	–	151	1180.6080	1179.6007	1179.5270	62.5	0	K.FTFEMYQSK.A
236	–	242	865.4660	864.4587	864.4130	52.9	0	R.SPNYWAK.D
273	–	282	1297.7690	1296.7617	1296.6462	89.1	1	K.ARQFNLYEEK.N
286	–	297	1460.8410	1459.8337	1459.7361	66.9	0	R.NWVTAYHFPAVR.S
298	–	305	909.6030	908.5957	908.5331	68.9	1	R.SGLIKTYK.A
323	–	332	1204.6570	1203.6497	1203.6836	-28.2	1	R.RAPLNDIHLR.Q
422	–	431	1134.6300	1133.6227	1133.5941	25.2	1	K.DAGYVIRQGR.L
432	–	438	879.4010	878.3937	878.4610	-76.6	1	R.LYDRQGK.A
606	–	611	803.3880	802.3807	802.4119	-38.9	0	K.LDRPMR.K + Oxidation (M)

Spot number: **66**
Mass: 8842 Score: 86 Queries matched: 7
Plasmid stabilization protein [Pseudomonas sp. A46]

Start	–	End	Observed	Mr(expt)	Mr(calc)	ppm	M	Peptide
1	–	7	807.3830	806.3757	806.4320	-69.8	0	-.MASITIR.N + Oxidation (M)
8	–	14	832.4840	831.4767	831.3974	95.4	0	R.NLDDDLK.A
17	–	26	1079.5660	1078.5587	1078.6359	-71.6	1	R.LRVLAASHGR.S
19	–	26	810.3820	809.3747	809.4508	-94.0	0	R.VLAASHGR.S
27	–	33	879.3770	878.3697	878.3804	-12.1	0	R.SMEEEVR.V
34	–	52	2028.9780	2027.9707	2028.0786	-53.2	1	R.VILAEALSRQDKPSAMGSR.I
43	–	56	1569.8400	1568.8327	1568.7841	31.0	1	R.QDKPSAMGSRIHSR.F

Spot number: **67**
Mass: 60842Score: 82 Queries matched: 9
Phosphoenolpyruvate carboxykinase (ATP) [Selenomonas sp. oral taxon 138]

Start	–	End	Observed	Mr(expt)	Mr(calc)	ppm	M	Peptide
112	–	130	2140.1310	2139.1237	2139.0718	24.3	1	R.LFVVDAFCGANVNTRMAVR.F
234	–	249	1656.7770	1655.7697	1655.8155	-27.7	0	K.EGQNTTLFFGLSGTGK.T
284	–	301	2112.0980	2111.0907	2111.1011	-4.90	1	K.VINLDPEAEPDIYQAIRR.N
420	–	426	865.4290	864.4217	864.4341	-14.3	0	K.YADELVR.K

Start –	End	Observed	Mr(expt)	Mr(calc)	ppm	M	Peptide
428 –	434	845.3520	844.3447	844.3497	-5.93	0	K.MEEHGSR.A
435 –	448	1536.8040	1535.7967	1535.7845	7.98	1	R.AYLVNTGWNGTGKR.I
449 –	455	832.4840	831.4767	831.4450	38.1	0	R.ISIQDTR.G
503 –	508	807.3830	806.3757	806.3446	38.6	0	R.TEWDEK.A
531 –	538	800.3740	799.3667	799.4440	-96.6	0	R.ELVSAGPK.L

Spot number: **68**
Mass: 22847 Score: 78 Queries matched: 6
DNA-binding response regulator, partial [Streptomyces hirsutus]

Start –	End	Observed	Mr(expt)	Mr(calc)	ppm	M	Peptide
5 –	19	1687.9950	1686.9877	1686.9417	27.3	1	R.VLLADDQALLRSAFR.V
46 –	57	1460.8320	1459.8247	1459.7453	54.4	1	R.EQRADVVLMDIR.M + Oxidation (M)
81 –	98	2096.0970	2095.0897	2095.1201	-14.5	0	R.VVILTTFEVDDYVVQSLR.A
99 –	107	807.3810	806.3737	806.4286	-68.1	0	R.AGASGFLGK.G
140 –	152	1304.7310	1303.7237	1303.6157	82.9	0	R.FLAQAGPSDGADR.D
209 –	215	800.4040	799.3967	799.4552	-73.1	1	R.DRAQLVV.-

Spot number: **69**
Mass: 147809 Score: 79 Queries matched: 11
Conjugative transfer relaxase/helicase TraI, partial [Klebsiella oxytoca]

Start –	End	Observed	Mr(expt)	Mr(calc)	ppm	M	Peptide
69 –	75	807.3730	806.3657	806.3593	8.02	0	R.MQDGVNK.H + Oxidation (M)
125 –	143	2012.9420	2011.9347	2012.1266	-95.4	1	R.VQKDGVSETVLTGNLIIAR.F
324 –	331	832.4970	831.4897	831.4450	53.8	0	K.TIAGLSDR.K
448 –	458	1304.7340	1303.7267	1303.6918	26.8	1	R.ERVAELTLMAR.E + Oxidation (M)
688 –	695	993.5300	992.5227	992.4597	63.5	1	K.EGERLDMK.V + Oxidation (M)
711 –	718	870.5480	869.5407	869.4607	92.1	0	K.LPVAEGER.L
725 –	731	856.5230	855.5157	855.5178	-2.40	1	K.IPETRLK.G
843 –	858	1687.9880	1686.9807	1686.8536	75.3	1	K.ARSGETELDAAIAQQK.A
845 –	858	1460.8200	1459.8127	1459.7154	66.7	0	R.SGETELDAAIAQQK.A

Start	–	End	Observed	Mr(expt)	Mr(calc)	ppm	M	Peptide
1031	–	1037	806.3680	805.3607	805.3752	-18.0	0	R.AVGEMQR.A + Oxidation (M)
1121	–	1129	921.3840	920.3767	920.4637	-94.5	0	R.SAADVAIMK.E + Oxidation (M)

Spot number: **70**
Mass: 29595 Score: 75 Queries matched: 7
Molybdenum cofactor biosysynthesis protein [Streptomyces]

Start	–	End	Observed	Mr(expt)	Mr(calc)	ppm	M	Peptide
4	–	15	1377.7290	1376.7217	1376.8041	-59.8	0	K.LHVQALHVHPVK.S
38	–	52	1740.9040	1739.8967	1739.9319	-20.2	1	R.WALIDPEGTVVTQRR.H
122	–	131	1134.6230	1133.6157	1133.6193	-3.16	0	R.LVHLDDPAVR.R
213	–	219	805.3400	804.3327	804.3661	-41.4	1	R.GARECGR.C
220	–	228	1079.5610	1078.5537	1078.5077	42.6	0	R.CVVTTTDQR.T
220	–	232	1536.7890	1535.7817	1535.7362	29.6	1	R.CVVTTTDQRTAER.G
235	–	242	927.5130	926.5057	926.5549	-53.1	1	R.EPLKTLAR.H

Spot number: **71**
Mass: 22222 Score: 117 Queries matched: 6
50S ribosomal protein L3 [Serratia]

Start	–	End	Observed	Mr(expt)	Mr(calc)	ppm	M	Peptide
1	–	8	861.5030	860.4957	860.5154	-22.8	1	-.MIGLVGKK.L + Oxidation (M)
39	–	55	1794.8030	1793.7957	1793.8908	-53.0	1	K.DLANDGYRAVQVTTGSK.K
71	–	83	1447.7280	1446.7207	1446.7368	-11.1	1	K.AGVEAGRGLWEFR.T
78	–	83	807.3540	806.3467	806.4075	-75.4	0	R.GLWEFR.T
84	–	106	2497.1090	2496.1017	2496.2384	-54.7	1	R.TAEGEEFTAGQNISVEIFAEVKK.V
142	–	157	1656.8890	1655.8817	1655.8995	-10.7	1	R.VPGSIGQNQTPGKVFK.G

Spot number: **72**
Succinyl-CoA synthetase subunit beta [Pseudomonas putida KT2440]

Calc. MH+	Meas. Mr	Calc. Mr	Dev.(Da)	Score	Mascot Score	Sequence
1569.838	1568.829	1568.831	-0.002	210	83	ATIDPLVGAQPFQGR

Calc. MH+	Meas. Mr	Calc. Mr	Dev.(Da)	Score	Mascot Score	Sequence
1734.865	1734.860	1734.858	0.002	120	15	NLVTYQTDANGQPVSK

Spot number: **73**
Mass: 60913 Score: 73 Queries matched: 9
Lysine-tRNA ligase [Ruminococcus sp. CAG: 353]

Start	–	End	Observed	Mr(expt)	Mr(calc)	ppm	M	Peptide
82	–	92	1242.6720	1241.6647	1241.6728	-6.47	1	K.AALDENRINVK.I
89	–	96	870.5250	869.5177	869.5447	-31.0	1	R.INVKIAGR.I
118	–	123	811.3390	810.3317	810.4058	-91.4	0	R.MQVYVR.M + Oxidation (M)
124	–	136	1630.8560	1629.8487	1629.7345	70.1	1	R.MNDIGEEQFKEFK.T + Oxidation (M)
260	–	268	1134.6080	1133.6007	1133.6808	-70.6	1	R.ISLELYLKR.L
333	–	356	2665.4010	2664.3937	2664.4197	-9.73	1	K.ITYGGVEIDLGKPFERLTMVEAVK.K
470	–	484	1722.7960	1721.7887	1721.7744	8.32	0	R.LGDEEANSIDEDFLR.A
515	–	523	1079.5410	1078.5337	1078.5733	-36.7	0	R.DVLLFPTMK.T + Oxidation (M)
515	–	527	1536.7720	1535.7647	1535.8269	-40.5	1	R.DVLLFPTMKTLDK.- + Oxidation (M)

Spot number: **74**
Mass: 20163 Score: 82 Queries matched: 6
Hypothetical protein UV73_C0010G0061 [Candidatus Gottesmanbacteria bacterium GW2011_GWA2_43_14]

Start	–	End	Observed	Mr(expt)	Mr(calc)	ppm	M	Peptide
50	–	61	1409.7020	1408.6947	1408.7602	-46.5	0	K.WLPIEPVALDEK.C
50	–	64	1794.7910	1793.7837	1793.9386	-86.3	1	K.WLPIEPVALDEKCPK.C
65	–	78	1568.7820	1567.7747	1567.7673	4.71	1	K.CGSPVVMMVTRFGK.K
94	–	114	2424.2210	2423.2137	2423.0951	49.0	0	K.QATGCDFVEWINGTTEPLEEK.C
115	–	121	879.3580	878.3507	878.3262	27.9	0	K.CPECGEK.L
122	–	131	1079.5470	1078.5397	1078.6023	-58.0	0	K.LVLFTTNSGK.R

Spot number: **75**
ABC transporter substrate-binding protein [Pseudomonas putida KT2440]

Calc. MH+	Meas. Mr	Calc. Mr	Dev.(Da)	Score	Mascot Score	Sequence
1536.800	1535.777	1535.793	-0.016	610	84	FVVYEWHFGKPK

Calc. MH+	Meas. Mr	Calc. Mr	Dev.(Da)	Score	Mascot Score	Sequence
1264.653	1264.633	1264.801	0.002	254	78	QQYGEGIATAVK

Spot number: **76**
Mass: 32268 Score: 67 Queries matched: 6
yaaT protein [Dialister invisus CAG:218]

Start	–	End	Observed	Mr(expt)	Mr(calc)	ppm	M	Peptide
38	–	52	1671.8110	1670.8037	1670.7974	3.79	0	R.GMEYGYIVTIPAGER.V + Oxidation (M)
98	–	104	856.5010	855.4937	855.5364	-49.9	1	K.VPMKLLR.T
114	–	127	1703.7850	1702.7777	1702.8831	-61.9	1	K.IVFFFTAEGRVDFR.E
124	–	131	1005.5060	1004.4987	1004.5655	-66.4	1	R.VDFRELVK.E
201	–	206	852.4310	851.4237	851.3816	49.5	0	R.LMCCLR.Y
266	–	280	1794.7740	1793.7667	1793.8142	-26.5	1	R.TKTMQWDVVESVENE.-

Spot number: **77**
Mass: 80512 Score: 75 Queries matched: 8
Hypothetical protein GL50803_115478 [Giardia lamblia ATCC 50803]

Start	–	End	Observed	Mr(expt)	Mr(calc)	ppm	M	Peptide
2	–	8	852.4340	851.4267	851.3661	71.2	0	M.PDDSQYK.R
10	–	24	1706.8190	1705.8117	1705.8423	-18.0	1	R.NLDAWFEAARSGEIK.F
404	–	411	856.5010	855.4937	855.5178	-28.1	0	R.LADLLGVR.D
515	–	529	1753.8770	1752.8697	1752.8828	-7.48	1	R.GEVELMNAHVRELEK.H
562	–	577	1630.8660	1629.8587	1629.7706	54.1	1	R.AEAAGGNAARELDETR.R
593	–	602	1339.6940	1338.6867	1338.5795	80.1	0	K.LLEEMEEMQR.N + 2 Oxidation (M)
665	–	671	832.4270	831.4197	831.4814	-74.2	1	K.RLSDTLK.K
728	–	735	808.3570	807.3497	807.3987	-60.7	0	R.EAHAGPAR.F

Spot number: **78**
Sugar ABC transporter substrate-binding protein [Pseudomonas extremaustralis]

Calc. MH+	Meas. Mr	Calc. Mr	Dev.(Da)	Score	Mascot Score	Sequence
1630.87	1629.859	1629.863	-0.004	1137	111	YKGDYVAVPVNIHR

Calc. MH+	Meas. Mr	Calc. Mr	Dev.(Da)	Score	Mascot Score	Sequence
1671.812	1671.812	1671.812	0.003	147	58	GTVEVVHWWTSGGEK

Spot number: **79**
Mass: 33123 Score: 131 Queries matched: 13
Amino acid ABC transporter substrate-binding protein [Pseudomonas sp. 11/12A]

Start	–	End	Observed	Mr(expt)	Mr(calc)	ppm	M	Peptide
33	–	44	1309.7720	1308.7647	1308.7514	10.2	1	K.IKESGVITLGHR.D
35	–	44	1068.5880	1067.5807	1067.5724	7.84	0	K.ESGVITLGHR.D
78	–	87	1154.6220	1153.6147	1153.6343	-16.9	0	K.DLDLPNLQVK.Y
88	–	96	1081.5630	1080.5557	1080.5564	-0.59	0	K.YNLVTSQTR.I
119	–	134	1823.9310	1822.9237	1822.9214	1.28	0	R.QQQVDFSVGIFEIGTR.L
153	–	164	1235.6290	1234.6217	1234.6154	5.16	0	K.NVVTTAGTTSER.I
168	–	183	1756.8170	1755.8097	1755.7953	8.19	1	K.SMNADKQMGMNVISAK.D + 2 Oxidation (M)
184	–	196	1492.6500	1491.6427	1491.6412	1.00	0	K.DHGESFQMLESGR.A
217	–	237	2419.0830	2418.0757	2418.0620	5.66	0	K.ADDWAVTGTPQSYEIYGCMVR.K
217	–	237	2435.0690	2434.0617	2434.0570	1.96	0	K.ADDWAVTGTPQSYEIYGCMVR.K + Oxidation (M)
217	–	238	2547.1730	2546.1657	2546.1570	3.43	1	K.ADDWAVTGTPQSYEIYGCMVRK.G
217	–	238	2563.1730	2562.1657	2562.1519	5.39	1	K.ADDWAVTGTPQSYEIYGCMVRK.G + Oxidation (M)
267	–	275	1143.5820	1142.5747	1142.5947	-17.5	0	K.WFMQPIPPK.G

Spot number: **80**
Mass: 94537 Score: 70 Queries matched: 8
Hypothetical protein A2Z93_11965 [Curvibacter sp. GWA2_64_110]

Start	–	End	Observed	Mr(expt)	Mr(calc)	ppm	M	Peptide
9	–	17	1143.5820	1142.5747	1142.6196	-39.3	1	R.AQQVWRDIK.W
219	–	242	2510.2680	2509.2607	2509.4070	-58.3	1	R.NALVTGHGLIWGIGLFGLFVGGRR.Q
328	–	334	800.3620	799.3547	799.3937	-48.7	1	R.DGKSTHR.D
384	–	404	2403.1690	2402.1617	2402.3256	-68.2	1	K.HSQALLNSIVEHIPAMVFLKR.A
458	–	467	1154.6220	1153.6147	1153.6594	-38.7	0	R.LLEIAEEPIK.A
506	–	511	807.3430	806.3357	806.3592	-29.2	0	R.MQDELR.D + Oxidation (M)

Start	–	End	Observed	Mr(expt)	Mr(calc)	ppm	M	Peptide
594	–	600	832.4760	831.4687	831.4636	6.12	1	R.LLMKDGR.I
684	–	691	919.3880	918.3807	918.4229	-45.9	0	R.AMGLNEER.I

Spot number: **81**
Mass: 30682 Score: 84 Queries matched: 9
Sensor histidine kinase [Thermosulfurimonas dismutans]

Start	–	End	Observed	Mr(expt)	Mr(calc)	ppm	M	Peptide
39	–	52	1701.7730	1700.7657	1700.9032	-80.8	1	R.GIYHDLRNCLTVLK.G
46	–	61	1757.8810	1756.8737	1757.0121	-78.7	1	R.NCLTVLKGGLEILSLK.C
90	–	102	1337.7250	1336.7177	1336.7099	5.86	0	R.LSALQANGLEGHK.E
120	–	132	1407.7040	1406.6967	1406.8358	-98.8	1	R.SIQTGLPPGIRLR.F
133	–	143	1320.6660	1319.6587	1319.6584	0.24	0	R.FEPSFCPLPVK.M
178	–	185	1036.5240	1035.5167	1035.4291	84.6	0	R.QVSEEECR.L
215	–	229	1679.7810	1678.7737	1678.8348	-36.4	1	K.IFEPMVTTKGENASR.N
255	–	269	1740.8520	1739.8447	1739.9246	-45.9	1	K.LGEGTRFEIYFPLAK.-
261	–	269	1127.6140	1126.6067	1126.6063	0.41	0	R.FEIYFPLAK.-

Spot number: **82**
Mass: 28490 Score: 81 Queries matched: 8
Transcriptional regulator, IclR family [Megasphaera paucivorans]

Start	–	End	Observed	Mr(expt)	Mr(calc)	ppm	M	Peptide
1	–	7	807.3820	806.3747	806.3956	-25.9	0	-.MIGSIDR.A + Oxidation (M)
30	–	35	832.4640	831.4567	831.4095	56.8	1	K.KMCIHK.S + Oxidation (M)
41	–	52	1407.7040	1406.6967	1406.7518	-39.1	1	R.TLETLESRGFVR.Q
49	–	59	1263.6840	1262.6767	1262.6367	31.7	1	R.GFVRQNQETGK.Y
75	–	81	870.5160	869.5087	869.4607	55.3	0	R.QQEPLQK.L
145	–	165	2635.3870	2634.3797	2634.2029	67.1	1	K.CLMAYADDAFLRQYENCILPR.F + Oxidation (M)
231	–	240	1082.6010	1081.5937	1081.6383	-41.2	0	R.ELPEIIAAVK.E
241	–	250	1127.6140	1126.6067	1126.5506	49.8	0	K.ETAEEISHIV.-

Spot number: **83**
Mass: 18355 Score: 137 Queries matched: 7
Transcriptional regulator NrdR [Zymomonas mobilis]

Start – End	Observed	Mr(expt)	Mr(calc)	ppm	M	Peptide
30 – 43	1790.8860	1789.8787	1789.7512	71.2	1	R.QCEECGARFTTFER.I
38 – 43	800.3760	799.3687	799.3865	-22.2	0	R.FTTFER.I
38 – 47	1337.7250	1336.7177	1336.6710	34.9	1	R.FTTFERIHMR.D
77 – 94	2064.1370	2063.1297	2063.1375	-3.76	0	K.RPVSPDQIELLVNSIQQK.I
108 – 126	2171.1490	2170.1417	2170.0762	30.2	1	R.LGEMTMEGLRTLDSVAYIR.F + Oxidation (M)
127 – 138	1433.7290	1432.7217	1432.6987	16.1	1	R.FASVYKDFTEAR.D

Spot number: **84**
Mass: 33827 Score: 72 Queries matched: 6
Uncharacterised protein [Mycobacterium]

Start – End	Observed	Mr(expt)	Mr(calc)	ppm	M	Peptide
1 – 7	832.4710	831.4637	831.4636	0.10	1	-.MGLLRDK.I
6 – 18	1549.7990	1548.7917	1548.7784	8.62	1	R.DKILETVDNPSYR.G
19 – 42	2518.1090	2517.1017	2517.2639	-64.4	1	R.GVYAFADDGGVTLTPEYAKTLTTK.D
185 – 192	879.3620	878.3547	878.3916	-42.0	0	K.QDSTAMAR.T
193 – 204	1350.6320	1349.6247	1349.6067	13.3	0	R.TNLDAAMQAMPR.G + 2 Oxidation (M)
205 – 223	1959.9620	1958.9547	1958.8759	40.2	0	R.GYEGPVAFQSGYGSVDGNR.A

Spot number: **85**
Hypothetical protein [Anaeromusa acidaminophila]

Calc. MH+	Meas. Mr	Calc. Mr	Dev.(Da)	Score	Mascot Score	Sequence
1350.709	1349.606	1349.701	-0.096	162	68	ILAIVDAYDAMR
1477.800	1477.790	1477.810	0.006	140	66	GVSLSVSIGFALDGR

Spot number: **86**
Mass: 15830 Score: 77 Queries matched: 6
DUF3284 domain-containing protein [Lactobacillus gasseri]

Start – End	Observed	Mr(expt)	Mr(calc)	ppm	M	Peptide

Start –	End	Observed	Mr(expt)	Mr(calc)	ppm	M	Peptide
1 –	11	1311.7390	1310.7317	1310.6904	31.5	1	-.MITITDQAKFK.A + Oxidation (M)
32 –	38	800.3730	799.3657	799.4076	-52.4	0	R.DNDLPVK.I
39 –	51	1350.6380	1349.6307	1349.6939	-46.8	0	K.IAAGTTYQQGNVK.A
39 –	61	2518.1230	2517.1157	2517.2387	-48.9	1	K.IAAGTTYQQGNVKAEITEYEFGK.K
86 –	101	1927.9830	1926.9757	1926.9397	18.7	1	R.GCQIIFKEDVLSFDEK.K
93 –	101	1081.5620	1080.5547	1080.4975	52.9	0	K.EDVLSFDEK.K

Spot number: **87**
Mass: 34115 Score: 87 Queries matched: 8
Hypothetical protein [Chitinophaga niabensis]

Start –	End	Observed	Mr(expt)	Mr(calc)	ppm	M	Peptide
35 –	41	806.3760	805.3687	805.4181	-61.4	0	K.QTSELTK.R
43 –	49	803.3750	802.3677	802.4185	-63.2	0	R.ILEESGR.F
70 –	89	2388.1120	2387.1047	2387.2862	-76.0	1	K.YAVVIQNTNNVFNLALRWPR.N
124 –	130	832.5190	831.5117	831.4425	83.2	0	K.MIGIGWR.S
133 –	144	1311.7210	1310.7137	1310.6354	59.8	0	R.TTGYAIEIDSNK.H
149 –	162	1350.6180	1349.6107	1349.6324	-16.1	0	R.IPPGEGEGTGHGSR.F
216 –	224	1187.5890	1186.5817	1186.6209	-33.0	0	K.MWPVEWIVK.Y
228 –	249	2571.1330	2570.1257	2570.2012	-29.4	1	K.GHVYSSSMGHLWKGETYPLSYR.C + Oxidation (M)

Spot number: **88**
Mass: 27575 Score: 69 Queries matched: 5
Hypothetical protein [Mycobacterium sp. E802]

Start –	End	Observed	Mr(expt)	Mr(calc)	ppm	M	Peptide
6 –	12	800.3830	799.3757	799.4552	-99.4	0	R.VNVAELR.A
95 –	101	809.3470	808.3397	808.3967	-70.4	0	R.LAEFDSK.F
200 –	205	805.2970	804.2897	804.3362	-57.8	1	R.DDRDER.E
203 –	208	819.3140	818.3067	818.3518	-55.1	1	R.DEREDR.D
206 –	212	919.3810	918.3737	918.4043	-33.3	1	R.EDRDEQK.D

Spot number: **89**

Mass: 35949 Score: 81 Queries matched: 7

Acetyl-CoA carboxylase carboxyl transferase subunit alpha [Hyphomonadaceae bacterium TMED125]

Start –	End	Observed	Mr(expt)	Mr(calc)	ppm	M	Peptide
2 –	11	1309.7200	1308.7127	1308.6350	59.4	0	M.NYLEFEEPIR.Q
15 –	21	845.3670	844.3597	844.4403	-95.4	0	R.EQLTQAR.D
44 –	63	2418.9960	2417.9887	2418.2292	-99.4	1	R.EVTEQVYSNLTPWQRVQVSR.H
142 –	148	832.5010	831.4937	831.4888	5.96	1	R.LMKLAEK.F
172 –	179	801.3500	800.3427	800.4140	-89.1	0	R.GQGEAIAR.N
180 –	188	1176.5790	1175.5717	1175.5791	-6.25	0	R.NLIEMCQLR.V
302 –	307	800.4060	799.3987	799.4664	-84.7	1	R.IDQRIR.K

Spot number: **90**

Glutamate/aspartate ABC transporter periplasmic glutamate/aspartate-binding protein GltI [Pseudomonas protegens Pf-5]

Calc. MH+	Meas. Mr	Calc. Mr	Dev.(Da)	Score	Mascot Score	Sequence
1783.842	1783.899	1783.840	0.005	875	98	AVAFMMDDALLAGEMAK
1823.929	1822.993	1822.921	0.071	2229	129	QQQVDFSVGIFEIGTR

Spot number: **91**

Mass: 43691 Score: 105 Queries matched: 11

Elongation factor Tu [Pseudomonas]

Start –	End	Observed	Mr(expt)	Mr(calc)	ppm	M	Peptide
26 –	34	947.5560	946.5487	946.5447	4.21	0	K.TTLTAALTR.V
60 –	75	1800.9240	1799.9167	1799.9530	-20.1	0	R.GITINTAHVEYNSLIR.H
76 –	90	1768.7250	1767.7177	1767.7787	-34.5	0	R.HYAHVDCPGHADYVK.N
118 –	124	867.5210	866.5137	866.4974	18.9	0	R.EHILLSR.Q
195 –	208	1616.8060	1615.7987	1615.8457	-29.1	0	R.LVETLDSYIPDPVR.V
242 –	252	1238.6720	1237.6647	1237.7030	-31.0	0	K.VQDPLEIVGLR.D
267 –	273	830.4950	829.4877	829.4657	26.5	1	R.KLLDEGR.A
274 –	283	1088.5420	1087.5347	1087.5444	-8.91	0	R.AGENCGVLLR.G
318 –	328	1304.6450	1303.6377	1303.6309	5.21	1	K.EEGGRHTPFFK.G
329 –	337	1233.5890	1232.5817	1232.6091	-22.2	0	K.GYRPQFYFR.T

Start	–	End	Observed	Mr(expt)	Mr(calc)	ppm	M	Peptide
369	–	377	1005.5000	1004.4927	1004.4961	-3.32	0	K.TIAMEDGLR.F

Spot number: 92
Mass: 39197 Score: 98 Queries matched: 11
N-acetylmuramoyl-L-alanine amidase [Bacillus cereus]

Start	–	End	Observed	Mr(expt)	Mr(calc)	ppm	M	Peptide
1	–	19	2159.9930	2158.9857	2159.0867	-46.8	1	-.MIVPKGNENIRPGYAMEPK.Y + Oxidation (M)
41	–	47	879.4380	878.4307	878.4246	6.96	0	K.YLDNQAR.G
162	–	174	1490.7830	1489.7757	1489.7235	35.1	1	R.KDVMPPPEAPHEK.D + Oxidation (M)
231	–	247	1897.9530	1896.9457	1896.9330	6.72	0	K.FTDLNEAHPSLVDGINR.A
262	–	269	892.4500	891.4427	891.4702	-30.8	0	K.FDPTATIK.R
270	–	279	1233.5760	1232.5687	1232.6183	-40.2	1	K.RDEAVIMIDR.A + Oxidation (M)
271	–	288	2269.9800	2268.9727	2269.1201	-64.9	1	R.DEAVIMIDRALEYNWIYR.K
306	–	321	1794.7800	1793.7727	1793.9424	-94.6	1	K.ALQNVYAYGIVKGNER.N
318	–	327	1189.5820	1188.5747	1188.5887	-11.8	1	K.GNERNEFVPK.G
322	–	332	1219.5630	1218.5557	1218.6357	-65.6	1	R.NEFVPKGTATR.A
342	–	348	803.4720	802.4647	802.4622	3.10	1	R.MLKVIEA.-

Spot number: 93
Mass: 16866 Score: 85 Queries matched: 7
Hypothetical protein [Acetobacter tropicalis]

Start	–	End	Observed	Mr(expt)	Mr(calc)	ppm	M	Peptide
1	–	15	1867.8470	1866.8397	1866.8393	0.24	1	-.MWPETARDCTAWSLK.T + Oxidation (M)
25	–	41	1856.8810	1855.8737	1855.9714	-52.6	0	R.LTGIVTPMVLDDPINSR.S + Oxidation (M)
87	–	99	1558.7080	1557.7007	1557.7463	-29.3	0	R.YLLPYSPDFNSSR.R
100	–	112	1524.8300	1523.8227	1523.8936	-46.5	1	R.RPSANLPPIYLRK.V
117	–	126	1189.5850	1188.5777	1188.5670	9.05	1	R.TTRCPVGQDR.H
127	–	133	870.5330	869.5257	869.4468	90.8	1	R.HTDRSVR.A
137	–	147	1536.7620	1535.7547	1535.7451	6.27	1	R.MLYKLLHCCWI.-

Spot number: **94**

B12-dependent methionine synthase [Mycobacterium intracellulare ATCC 13950]

Calc. MH+	Meas. Mr	Calc. Mr	Dev.(Da)	Score	Mascot Score	Sequence
1283.568	1282.529	1282.561	-0.032	99	69	MAQDQVADYAR 1: Oxidation (M)
1399.681	1399.677	1399.689	0.008	80	66	TASISPDVNDPGAR

Spot number: **95**

Elongation factor Tu [Actinobacillus pleuropneumoniae serotype 3 (strain JL03)]

Calc. MH+	Meas. Mr	Calc. Mr	Dev.(Da)	Score	Mascot Score	Sequence
1233.616	1232.582	1232.609	-0.027	561	60	GYRPQFYFR
1376.632	1376.632	1376.632	0.023	147	61	AFDQIDNAPEEK

Spot number: **96**

Mass: 34772 Score: 76 Queries matched: 6

Flagellar MS-ring protein, partial [Xanthomonas campestris pv. musacearum NCPPB 4384]

Start	–	End	Observed	Mr(expt)	Mr(calc)	ppm	M	Peptide
1	–	16	1807.8950	1806.8877	1806.8604	15.1	1	-.MALAISKENMNQNAEK.A + Oxidation (M)
17	–	23	879.4370	878.4297	878.4035	29.9	0	K.AGQWFDR.V
26	–	32	845.4500	844.4427	844.5130	-83.3	1	R.SLQITRK.L
107	–	115	803.4750	802.4677	802.4549	16.0	0	K.LAGSGLTGK.E
228	–	250	2571.2590	2570.2517	2570.2296	8.63	1	R.MLSIADPNSDAAQHAAQFEQVRR.Q + Oxidation (M)
299	–	318	2056.0600	2055.0527	2054.9869	32.0	1	K.LRSEQVNDTSTTATGPQGPP.-

Spot number: **97**

Mass: 34626 Score: 80 Queries matched: 7

Hypothetical protein [Streptomyces sp. ERV7]

Start	– End	Observed	Mr(expt)	Mr(calc)	ppm	M	Peptide
38	– 44	800.4480	799.4407	799.4915	-63.6	1	R.AKELIAR.E
118	– 125	879.3930	878.3857	878.4167	-35.3	0	R.MSVAAEQK.A + Oxidation (M)
227	– 238	1525.8650	1524.8577	1524.8048	34.7	1	K.WLEEIQPRLDAR.T
398	– 404	819.3820	818.3747	818.4286	-65.9	0	R.AWQSLSK.L
482	– 491	1219.6000	1218.5927	1218.5889	3.12	1	K.MSRGMSLFFK.L + Oxidation (M)
521	– 531	1233.6180	1232.6107	1232.6084	1.87	1	R.AWGTMDAGLRR.T

Start – End	Observed	Mr(expt)	Mr(calc)	ppm	M	Peptide
599 – 607	1093.6040	1092.5967	1092.5676	26.7	1	R.SLRAYVNDR.V

Spot number: **98**

Dihydrolipoyl dehydrogenase [Pseudomonas fluorescens]

Calc. MH+	Meas. Mr	Calc. Mr	Dev.(Da)	Score	Mascot Score	Sequence
1807.897	1806.878	1806.89	-0.012	68	72	AEGVEVNVGTFPFAASGR
1867.079	1867.085	1867.071	0.005	67	70	LGVIGAGVIGLELGSVWAR

Spot number: **99**

Mass: 64753 Score: 82 Queries matched: 11

Penicillin-binding protein 2 [Clostridium botulinum]

Start	–	End	Observed	Mr(expt)	Mr(calc)	ppm	M	Peptide
2	–	8	832.4910	831.4837	831.4086	90.3	0	M.INTENNK.R
105	–	120	1966.9260	1965.9187	1965.8850	17.1	1	K.NCNKNYDIDNEVNINK.G
124	–	138	1850.8910	1849.8837	1849.9574	-39.8	1	K.IIWSIDENTYNNLKK.I
249	–	263	1668.7370	1667.7297	1667.8076	-46.7	0	K.YDQIGVILMEADTGK.I + Oxidation (M)
304	–	312	1053.5530	1052.5457	1052.5363	8.98	0	K.NNNISLNHK.Y
304	–	314	1344.7260	1343.7187	1343.6945	18.0	1	K.NNNISLNHKYK.H
437	–	448	1536.7890	1535.7817	1535.6820	64.9	1	K.NNNIIEKCDTCR.K
455	–	469	1700.8970	1699.8897	1699.9654	-44.5	1	K.INSNILKSQMLNVVK.E
462	–	469	918.4990	917.4917	917.5004	-9.49	0	K.SQMLNVVK.E
504	–	518	1882.8380	1881.8307	1881.8396	-4.72	1	K.DLKEYCDGWFAGFFK.L
519	–	535	2033.8410	2032.8337	2033.0292	-96.1	1	K.LNDKYYSMVVFVQNIGK.D + Oxidation (M)

Spot number: **100**

Mass: 22345 Score: 81 Queries matched: 6

Phosphohydrolase [Deinococcus geothermalis]

Start	–	End	Observed	Mr(expt)	Mr(calc)	ppm	M	Peptide
1	–	9	1056.6200	1055.6127	1055.5433	65.7	0	-.MIAEPPNLR.H + Oxidation (M)
10	–	21	1407.6780	1406.6707	1406.6942	-16.7	0	R.HPLAELAGWEER.V
22	–	29	1026.6080	1025.6007	1025.6280	-26.6	1	R.VRLMVRPR.R

Start	–	End	Observed	Mr(expt)	Mr(calc)	ppm	M	Peptide
31	–	36	800.3840	799.3767	799.4341	-71.7	0	R.FEHVLR.V
47	–	54	845.3660	844.3587	844.4039	-53.5	0	R.ANGLDEAR.A
76	–	97	2307.0650	2306.0577	2306.2066	-64.5	1	R.LAPPECAIDAAHPLALHGRAAR.T

Spot number: **101**
Mass: 44132 Score: 137 Queries matched: 8
Glutamate carboxypeptidase [Variovorax sp. EL159]

Start	–	End	Observed	Mr(expt)	Mr(calc)	ppm	M	Peptide
30	–	46	1858.8660	1857.8587	1857.9472	-47.6	0	R.DNVLFQAATDEQPAVIK.T
87	–	101	1469.8430	1468.8357	1468.8613	-17.4	1	K.SAGIVVGDNIVGKIK.G
121	–	129	972.5200	971.5127	971.5916	-81.2	1	K.GILAKAPFR.V
179	–	192	1536.7890	1535.7817	1535.7580	15.5	1	K.GSFGSRDLIQEEAK.L
271	–	289	1882.8380	1881.8307	1881.9908	-85.1	0	K.AGNVSNIIPASATLNADVR.Y
293	–	306	1668.7370	1667.7297	1667.7461	-9.80	1	R.NEDFDAAMKTLEER.A
324	–	335	1160.5190	1159.5117	1159.5734	-53.2	0	R.GRPAFNAGEGGK.K
390	–	400	1233.5880	1232.5807	1232.6401	-48.2	0	K.AEYVDISAIPR.R

Spot number: **102**
Mass: 71334 Score: 142 Queries matched: 10
DNA mismatch repair protein MutL [Petrotoga mobilis]

Start	–	End	Observed	Mr(expt)	Mr(calc)	ppm	M	Peptide
210	–	216	918.4990	917.4917	917.5334	-45.4	1	K.IYPELKR.D
217	–	230	1700.8970	1699.8897	1699.7723	69.1	0	R.DDLIEIEHNDSLCK.I
246	–	255	1233.5880	1232.5807	1232.6051	-19.7	0	R.TAQHFFVNNR.Y
302	–	317	1850.8910	1849.8837	1849.9859	-55.2	1	K.LEVKFTDEQMVASLLK.K
306	–	318	1525.8530	1524.8457	1524.7858	39.3	1	K.FTDEQMVASLLKK.V + Oxidation (M)
431	–	438	972.5200	971.5127	971.5763	-65.5	1	K.INSLEKLR.I
439	–	446	870.5250	869.5177	869.5334	-18.1	0	R.ILGIVAER.Y
518	–	528	1344.7260	1343.7187	1343.7184	0.26	1	K.LEEDEKEIIVK.G
542	–	551	1204.6110	1203.6037	1203.6499	-38.4	0	R.LIFEIADDLR.I

Start	–	End	Observed	Mr(expt)	Mr(calc)	ppm	M	Peptide
579	–	595	1966.9260	1965.9187	1965.9717	-27.0	1	R.DNPTGMETLLNTIFEKK.L + Oxidation (M)

Spot number: **103**
Mass: 117424 Score: 91 Queries matched: 16
Hypothetical protein A2Y25_00865 [Candidatus Melainabacteria bacterium GWF2_37_15]

Start	–	End	Observed	Mr(expt)	Mr(calc)	ppm	M	Peptide
250	–	259	1203.6500	1202.6427	1202.6441	-1.15	1	K.AQLQAEMLKR.F + Oxidation (M)
265	–	280	1832.8930	1831.8857	1831.9931	-58.6	1	K.NIIKYDPTTDIIADLK.L
321	–	327	803.3520	802.3447	802.4185	-91.9	0	K.NIDINSK.L
348	–	355	977.4890	976.4817	976.4978	-16.4	0	K.QDFNLNVK.T
417	–	423	800.3840	799.3767	799.4440	-84.1	0	K.DLSQPLK.E
424	–	435	1456.7770	1455.7697	1455.6994	48.3	1	K.EFNSTLNFNKDK.V
460	–	474	1700.9010	1699.8937	1699.9872	-55.0	1	K.LFSDIKISIPNVNLK.T
485	–	494	1172.5870	1171.5797	1171.6965	-99.6	0	K.LFADLKPQLK.D
703	–	719	1850.9040	1849.8967	1849.9091	-6.71	0	K.SDAILLTTGVLNMEDSR.I + Oxidation (M)
703	–	721	2092.0030	2090.9957	2091.0882	-44.2	1	K.SDAILLTTGVLNMEDSRIK.L + Oxidation (M)
730	–	737	870.5510	869.5437	869.5375	7.19	0	K.LAPPFVVK.A
738	–	753	1867.8460	1866.8387	1866.9145	-40.6	1	K.ALEINSDFMNVDRLSK.A + Oxidation (M)
754	–	767	1525.8490	1524.8417	1524.8008	26.8	0	K.ALEQKPVNSAGQQR.G
868	–	885	1945.9330	1944.9257	1945.0091	-42.9	1	R.DQIFGGMKGHIQLSTIGK.T + Oxidation (M)
959	–	970	1375.7280	1374.7207	1374.6636	41.6	1	R.LSSFMSGTVRMK.D + 2 Oxidation (M)
971	–	985	1593.8710	1592.8637	1592.8410	14.3	1	K.DNYADLTVLGKLSGK.I

Spot number: **104**
Mass: 76384 Score: 84 Queries matched: 9
Hypothetical protein [Bacteroides uniformis]

Start	–	End	Observed	Mr(expt)	Mr(calc)	ppm	M	Peptide
58	–	73	1807.8630	1806.8557	1806.8193	20.1	0	K.DLCEDIIEDIGSVSDK.Y
58	–	77	2319.0600	2318.0527	2318.0624	-4.17	1	K.DLCEDIIEDIGSVSDKYGYK.E
175	–	193	2056.0310	2055.0237	2055.1398	-56.5	1	K.IITIQGLSGTGKTELLMHK.L + Oxidation (M)

Start	–	End	Observed	Mr(expt)	Mr(calc)	ppm	M	Peptide
223	–	231	1172.5870	1171.5797	1171.5736	5.23	0	R.IPEFFNFMK.V
362	–	378	1945.9330	1944.9257	1944.9689	-22.2	1	K.TLMFAQALGMGLFEKEK.L + 2 Oxidation (M)
411	–	422	1456.7770	1455.7697	1455.6769	63.7	0	R.FEDVDLDFESLK.I
524	–	535	1536.8060	1535.7987	1535.7224	49.7	1	K.SYRNTMYTMLTR.S
540	–	554	1682.8120	1681.8047	1681.8675	-37.3	1	R.SYLVLPKSNDNGFTK.E
578	–	587	1243.6820	1242.6747	1242.6972	-18.1	1	K.EQIDIKAWIK.D

Spot number: **105**
Mass: 52475 Score: 133 Queries matched: 9
ATP synthase subunit beta [Renibacterium salmoninarum]

Start	–	End	Observed	Mr(expt)	Mr(calc)	ppm	M	Peptide
2	–	11	1005.5790	1004.5717	1004.4887	82.7	0	M.TATATEQGAR.T
175	–	184	1203.6500	1202.6427	1202.6693	-22.1	0	K.TVLIQEMITR.V
175	–	184	1219.5930	1218.5857	1218.6642	-64.4	0	K.TVLIQEMITR.V + Oxidation (M)
188	–	201	1397.6590	1396.6517	1396.6736	-15.6	0	R.NFGGTSVFAGVGER.T
202	–	219	2092.0030	2090.9957	2090.9942	0.71	1	R.TREGNDLWVEMEESGVLK.D
204	–	219	1850.9040	1849.8967	1849.8404	30.5	0	R.EGNDLWVEMEESGVLK.D + Oxidation (M)
267	–	280	1465.7370	1464.7297	1464.7573	-18.8	0	R.FTQAGSEVSTLLGR.M
281	–	301	2319.0600	2318.0527	2318.1035	-21.9	0	R.MPSAVGYQPNLADEMGLLQER.I
349	–	362	1490.7430	1489.7357	1489.7776	-28.1	0	R.GLYPAIDPLTSTSR.I

Spot number: **106**
Mass: 35443 Score: 146 Queries matched: 9
Acetyl-CoA carboxylase carboxyl transferase subunit alpha [Marinobacter hydrocarbonoclasticus]

Start	–	End	Observed	Mr(expt)	Mr(calc)	ppm	M	Peptide
24	–	41	2033.8460	2032.8387	2033.0099	-84.2	1	R.MVGNDTDINITDEISRLK.K
69	–	90	2757.3410	2756.3337	2756.2718	22.5	0	R.RPYTQDYIDLIFEDFDELHGDR.R
105	–	117	1465.7370	1464.7297	1464.7759	-31.5	0	R.LDNTPVMIIGHQK.G
105	–	117	1481.7350	1480.7277	1480.7708	-29.1	0	R.LDNTPVMIIGHQK.G + Oxidation (M)
140	–	149	1219.5930	1218.5857	1218.6212	-29.1	1	K.ALRLMEMAER.F

Start	–	End	Observed	Mr(expt)	Mr(calc)	ppm	M	Peptide
143	–	149	879.3810	878.3737	878.3990	-28.7	0	R.LMEMAER.F
173	–	187	1593.8710	1592.8637	1592.7981	41.2	0	R.GQSEAIAFNLAVMSR.L
173	–	189	1850.9040	1849.8967	1849.9720	-40.7	1	R.GQSEAIAFNLAVMSRLK.T + Oxidation (M)
275	–	284	1160.5230	1159.5157	1159.5907	-64.6	1	R.NPEKMAETLK.T

Spot number: **107**

Mass: 44178 Score: 179 Queries matched: 11

Protein serine/threonine phosphatase [Acetobacter sp. CAG: 977]

Start	–	End	Observed	Mr(expt)	Mr(calc)	ppm	M	Peptide
4	–	16	1525.8490	1524.8417	1524.7130	84.4	0	R.SETLFIDVDCAQK.C
33	–	38	803.3520	802.3447	802.3821	-46.5	0	R.IEEQER.L
67	–	76	1203.6500	1202.6427	1202.5720	58.8	0	K.FVSADFNFTR.A
77	–	91	1700.9010	1699.8937	1699.8749	11.1	0	R.AAEVIMNSLPICQVR.K
139	–	144	807.4000	806.3927	806.4035	-13.3	1	K.NFENRK.M
158	–	176	2092.0030	2090.9957	2091.0208	-12.0	0	R.LIFYSDGVTQSGMGNPLHR.F
236	–	247	1375.7280	1374.7207	1374.7442	-17.1	1	R.RLMVLSGPPYSR.D
237	–	247	1219.5930	1218.5857	1218.6431	-47.1	0	R.LMVLSGPPYSR.D
256	–	261	800.3840	799.3767	799.4116	-43.6	0	R.TFIEYK.G
264	–	277	1465.7370	1464.7297	1464.7507	-14.3	1	K.KAICGGTTANFVAR.E
387	–	393	809.3780	808.3707	808.4331	-77.1	0	K.NVTVTYI.-

Spot number: **108**

Mass: 23728 Score: 90 Queries matched: 11

TadE family protein [Rhodanobacter spathiphylli]

Start	–	End	Observed	Mr(expt)	Mr(calc)	ppm	M	Peptide
1	–	8	947.5490	946.5417	946.4767	68.7	1	-.MNLGGRQR.G + Oxidation (M)
2	–	8	800.3880	799.3807	799.4413	-75.7	1	M.NLGGRQR.G
70	–	78	1029.6040	1028.5967	1028.5477	47.7	0	R.GLMPLYAHK.V
79	–	86	803.3680	802.3607	802.3933	-40.6	0	K.VGNGEATR.A
105	–	117	1433.7840	1432.7767	1432.7714	3.69	1	R.VISPTKAAFADWK.E
163	–	170	914.5020	913.4947	913.5307	-39.3	0	K.MIVPVIDK.L

Start	–	End	Observed	Mr(expt)	Mr(calc)	ppm	M	Peptide
163	–	170	930.4680	929.4607	929.5256	-69.8	0	K.MIVPVIDK.L + Oxidation (M)
178	–	196	2160.0170	2159.0097	2159.0317	-10.2	1	R.ESFYQGLSAREIAMLESGR.L + Oxidation (M)
188	–	196	1005.5890	1004.5817	1004.4960	85.3	0	R.EIAMLESGR.L
188	–	206	2132.0050	2130.9977	2131.1129	-54.1	1	R.EIAMLESGRLPIVSQAMVR.M + 2 Oxidation (M)
197	–	212	1855.9280	1854.9207	1855.0172	-52.0	1	R.LPIVSQAMVRMQTPIR.D + Oxidation (M)

Spot number: **109**
Mass: 4385 Score: 99 Queries matched: 6
Hypothetical protein BN2364_4186 [Alcanivorax dieselolei]

Start	–	End	Observed	Mr(expt)	Mr(calc)	ppm	M	Peptide
1	–	7	803.3680	802.3607	802.4007	-49.8	0	-.MSPNNIK.K
1	–	8	947.5490	946.5417	946.4906	54.0	1	-.MSPNNIKK.E + Oxidation (M)
2	–	8	800.3880	799.3807	799.4552	-93.1	1	M.SPNNIKK.E
8	–	21	1361.7280	1360.7207	1360.7562	-26.1	1	K.KESGLATVATGSIK.T
9	–	21	1233.6250	1232.6177	1232.6612	-35.3	0	K.ESGLATVATGSIK.T
9	–	23	1490.8300	1489.8227	1489.8100	8.53	1	K.ESGLATVATGSIKTR.I

Spot number: **110**
Mass: 52051 Score: 116 Queries matched: 11
Aldehyde dehydrogenase family protein [Staphylococcus aureus]

Start	–	End	Observed	Mr(expt)	Mr(calc)	ppm	M	Peptide
42	–	59	2025.0020	2023.9947	2023.9851	4.77	1	K.ADVDKAVEAADNVYLEFR.H
65	–	72	972.5510	971.5437	971.5400	3.87	1	K.ERQALLDK.I
81	–	101	2270.0070	2268.9997	2269.1801	-79.5	1	R.KDDIVQAITDELGAPLSLSER.V
216	–	230	1558.7560	1557.7487	1557.7280	13.3	1	K.VRMMSFTGSGPTGSK.I + Oxidation (M)
216	–	230	1574.7470	1573.7397	1573.7229	10.7	1	K.VRMMSFTGSGPTGSK.I + 2 Oxidation (M)
231	–	237	806.4760	805.4687	805.4367	39.7	1	K.IMEKAAK.D + Oxidation (M)
242	–	261	2160.0170	2159.0097	2159.1725	-75.4	1	K.VSLELGGKSPYIVLDDVDIK.E
308	–	324	1766.8560	1765.8487	1765.9323	-47.3	1	R.VGNPREDGTQVGPIISK.K
325	–	336	1524.8860	1523.8787	1523.7732	69.2	1	K.KQFDQVQNYINK.G

Start	–	End	Observed	Mr(expt)	Mr(calc)	ppm	M	Peptide
405	–	416	1283.5830	1282.5757	1282.6921	-90.7	1	K.YGLAGYVIGKDK.E
415	–	421	870.5510	869.5437	869.4607	95.5	1	K.DKETLHK.V

Spot number: **111**
Mass: 80990 Score: 198 Queries matched: 19
Penicillin-binding protein 2 [Bacillus cereus]

Start	–	End	Observed	Mr(expt)	Mr(calc)	ppm	M	Peptide
185	–	193	1029.6040	1028.5967	1028.5753	20.8	0	K.EDIEVLAIK.S
194	–	201	914.5020	913.4947	913.4327	67.9	1	K.SKMNAGYK.M + Oxidation (M)
196	–	208	1480.7470	1479.7397	1479.7578	-12.2	1	K.MNAGYKMTPQIVK.K
202	–	208	832.5020	831.4947	831.4524	50.9	0	K.MTPQIVK.K + Oxidation (M)
223	–	237	1700.9430	1699.9357	1699.8781	33.9	0	R.LSILPGVDTTVDWER.E
281	–	295	1855.9280	1854.9207	1854.8822	20.8	1	K.SYIEKQYEDVLHGMK.E + Oxidation (M)
286	–	295	1219.6130	1218.6057	1218.5703	29.1	0	K.QYEDVLHGMK.E
300	–	310	1242.7050	1241.6977	1241.6840	11.0	1	K.NITDRAGNIIR.T
319	–	333	1694.8260	1693.8187	1693.8015	10.2	0	K.SGNNLMLTVDMDLQK.K + Oxidation (M)
342	–	355	1722.7580	1721.7507	1721.7614	-6.17	1	R.NLRAFHSSEPMMDR.A + 2 Oxidation (M)
356	–	374	2033.9100	2032.9027	2033.0802	-87.3	1	R.AFVVMMNPKNGQILSLAGK.K + Oxidation (M)
467	–	481	1675.8160	1674.8087	1674.8287	-11.9	0	K.MAGIDYVPNNPLDIK.Q + Oxidation (M)
553	–	573	2521.2490	2520.2417	2520.3118	-27.8	1	R.MKPQIVQEIREQPNRPEEVGK.V + Oxidation (M)
586	–	597	1433.7840	1432.7767	1432.7020	52.1	0	R.VDMDLSYINHVK.E
586	–	597	1449.7020	1448.6947	1448.6970	-1.54	0	R.VDMDLSYINHVK.E + Oxidation (M)
617	–	625	930.4680	929.4607	929.5334	-78.2	0	K.GLPYKPAGK.T

Spot number: **112**
Mass: 46455 Score: 104 Queries matched: 13
Threonine ammonia-lyase [Sediminibacterium sp. C3]

Start	–	End	Observed	Mr(expt)	Mr(calc)	ppm	M	Peptide
49	–	58	1250.6470	1249.6397	1249.6666	-21.5	1	R.EDLQIVRSYK.L
59	–	74	1849.8240	1848.8167	1848.9152	-53.3	1	K.LRGAYNMISSLPEDQR.A
96	–	113	2024.9620	2023.9547	2024.1492	-96.1	0	K.VLNIQGIVFMPIITPNQK.V

Start	–	End	Observed	Mr(expt)	Mr(calc)	ppm	M	Peptide
143	–	160	2035.9380	2034.9307	2034.9510	-9.94	0	K.AFTESNGMTFIPPFDHPK.V
208	–	230	2307.9920	2306.9847	2307.2144	-99.5	1	K.IIGVEPEGAPSMSEALKQGAPVK.L
256	–	268	1474.6640	1473.6567	1473.7385	-55.5	0	K.EVIDEMVLVNEGK.V
256	–	268	1490.7310	1489.7237	1489.7334	-6.49	0	K.EVIDEMVLVNEGK.V + Oxidation (M)
306	–	320	1675.7750	1674.7677	1674.8359	-40.7	1	K.KVVCIVSGSNNDIDR.M
328	–	341	1734.8130	1733.8057	1733.9617	-90.0	1	R.SLQFEGLKHFFLIR.F
336	–	350	1800.9380	1799.9307	1800.0311	-55.8	1	K.HFFLIRFAQRPGALK.E
366	–	371	845.4470	844.4397	844.3789	72.0	0	R.FEYMQK.H
372	–	388	1882.8640	1881.8567	1881.9254	-36.5	1	K.HNKETGPALVGIEMQDK.A + Oxidation (M)
394	–	400	919.4250	918.4177	918.4593	-45.2	0	R.LLENMQR.Y + Oxidation (M)

Spot number: **113**
Mass: 40437 Score: 85 Queries matched: 10
Helix-turn-helix domain-containing protein [Pseudorhodobacter ferrugineus]

Start	–	End	Observed	Mr(expt)	Mr(calc)	ppm	M	Peptide
3	–	20	1974.9510	1973.9437	1973.9847	-20.7	0	R.SNLSSDFSLPAWLPDAVR.L
72	–	82	1334.6810	1333.6737	1333.5755	73.7	0	R.VQETMPCNPSR.K + Oxidation (M)
83	–	93	1233.5720	1232.5647	1232.6183	-43.5	1	R.KDAQTMTAPVR.S + Oxidation (M)
115	–	135	2213.1480	2212.1407	2212.1773	-16.5	1	R.LAEADTVLAIAPDMEKAAVLR.S + Oxidation (M)
170	–	184	1506.7260	1505.7187	1505.8566	-91.5	1	R.VSTYVITGAGRAALK.R
185	–	206	2131.9550	2130.9477	2131.0116	-30.0	1	K.RMGGDEVAGLAEAPAGFAGAER.A
256	–	273	2224.0490	2223.0417	2223.0201	9.73	1	R.MDFELAQMGPRVTQNWER.F + Oxidation (M)
289	–	297	930.4800	929.4727	929.4679	5.21	1	K.GGALDARDR.V
304	–	315	1242.6600	1241.6527	1241.6438	7.18	0	R.DLGPGLGDMVLR.C
329	–	335	879.4330	878.4257	878.4181	8.69	1	K.RMGWSAR.S + Oxidation (M)

Spot number: **114**
Mass: 36737 Score: 142 Queries matched: 8
DNA-directed RNA polymerase subunit alpha [Pseudomonas]

Start	–	End	Observed	Mr(expt)	Mr(calc)	ppm	M	Peptide

Start	–	End	Observed	Mr(expt)	Mr(calc)	ppm	M	Peptide
13	–	23	1250.6470	1249.6397	1249.6779	-30.6	0	R.HIDVQVVSPTR.A
26	–	33	970.5890	969.5817	969.5495	33.3	0	K.ITLEPLER.G
34	–	44	1142.5810	1141.5737	1141.5992	-22.4	0	R.GFGHTLGNALR.R
92	–	104	1468.7720	1467.7647	1467.8045	-27.1	1	K.LHGRDEVTLTLSK.K
182	–	190	1034.5510	1033.5437	1033.5556	-11.5	0	R.IAYVVENAR.V
271	–	283	1567.7760	1566.7687	1566.8042	-22.6	0	K.AENIYYIGDLIQR.T
298	–	309	1331.7170	1330.7097	1330.7456	-27.0	1	K.SLTEIKDVLASR.G
310	–	326	1854.8540	1853.8467	1853.9822	-73.1	1	R.GLSLGMRLDNWPPASLK.K

Spot number: **115**
Mass: 71757 Score: 85 Queries matched: 11
DNA ligase (NAD(+)) LigA [Campylobacter concisus]

Start	–	End	Observed	Mr(expt)	Mr(calc)	ppm	M	Peptide
77	–	94	2171.1460	2170.1387	2170.0115	58.6	0	R.MWSMEDIFSLGELDAWLK.R
77	–	95	2342.9800	2341.9727	2342.1075	-57.5	1	R.MWSMEDIFSLGELDAWLKR.G + Oxidation (M)
199	–	207	932.4740	931.4667	931.4975	-33.0	0	R.QLDSAVTAK.R
226	–	232	845.3510	844.3437	844.3749	-36.9	0	K.DHSEVMK.F
251	–	264	1636.7840	1635.7767	1635.7740	1.68	0	K.DELESAYNELLANR.D
278	–	292	1765.7380	1764.7307	1764.8829	-86.2	1	R.VNDLARCEQLGYTVK.F
489	–	510	2315.1660	2314.1587	2314.1739	-6.58	1	K.GAELSRFITGLGCEHIGEVAAK.K
495	–	510	1701.7760	1700.7687	1700.8556	-51.1	0	R.FITGLGCEHIGEVAAK.K
611	–	623	1323.6340	1322.6267	1322.6354	-6.56	0	K.TDFVLAGEEAGSK.L
611	–	626	1679.7850	1678.7777	1678.8414	-37.9	1	K.TDFVLAGEEAGSKLDK.A
627	–	642	1835.9660	1834.9587	1834.8948	34.8	0	K.ANELGVLVIDESEYER.L

Spot number: **116**
Mass: 57098 Score: 88 Queries matched: 12
Tetratricopeptide repeat protein [Thalassobaculum salexigens]

Start	–	End	Observed	Mr(expt)	Mr(calc)	ppm	M	Peptide
12	–	21	1219.5960	1218.5887	1218.6258	-30.4	1	R.QPPPRWADPR.N

Start	–	End	Observed	Mr(expt)	Mr(calc)	ppm	M	Peptide
51	–	73	2586.2750	2585.2677	2585.2730	-2.05	1	R.FRAVLDQDIEHPDALMGMGIVAR.A + 2 Oxidation (M)
53	–	73	2251.1250	2250.1177	2250.1137	1.80	0	R.AVLDQDIEHPDALMGMGIVAR.A
74	–	86	1540.7320	1539.7247	1539.7940	-45.0	1	R.AMRQHQTAAELLR.R + Oxidation (M)
134	–	154	2425.1270	2424.1197	2424.2662	-60.4	0	R.QNLGSVLNSIDRPYQALPHFR.E
160	–	176	1732.7830	1731.7757	1731.9155	-80.7	0	R.SGGPIDAVINYATVLSR.V
277	–	294	1994.9690	1993.9617	1993.9965	-17.4	1	R.GGDITLECSAKLMPLFAR.S + Oxidation (M)
295	–	305	1189.6060	1188.5987	1188.6503	-43.4	0	R.SFPGLTLVEAR.H
366	–	375	1130.5790	1129.5717	1129.6356	-56.6	1	K.VGLSWRSGLR.G
380	–	396	1968.9740	1967.9667	1968.0026	-18.2	1	R.SDMYAAIEDLAPLFRLK.D + Oxidation (M)
397	–	409	1567.7760	1566.7687	1566.7678	0.58	0	K.DIVFVNLQYDDAR.E
410	–	416	816.4600	815.4527	815.4389	17.0	0	R.EELAQVK.D

Spot number: **117**
Mass: 23307Score: 80 Queries matched: 9
Hypothetical protein AKJ43_00885 [candidate divison MSBL1 archaeon SCGC-AAA261D19]

Start	–	End	Observed	Mr(expt)	Mr(calc)	ppm	M	Peptide
61	–	68	1018.4630	1017.4557	1017.4516	4.08	0	K.EQVGEDWR.K
81	–	89	1130.5790	1129.5717	1129.6091	-33.1	1	K.ESELREIVR.L
86	–	99	1549.8130	1548.8057	1548.8624	-36.6	1	R.EIVRLVGPDALPDR.D
112	–	131	2425.1270	2424.1197	2424.0362	34.4	0	R.EDFLQQNAMHDVDTYCPVDK.Q
132	–	137	810.3990	809.3917	809.4469	-68.2	1	K.QLKMFK.T + Oxidation (M)
135	–	141	866.5240	865.5167	865.5095	8.30	1	K.MFKTVLK.L
142	–	151	1139.5320	1138.5247	1138.6346	-96.5	1	K.LHDKSLEAVK.K
166	–	172	816.4600	815.4527	815.4865	-41.4	1	K.SEIARLK.I
173	–	181	1117.5630	1116.5557	1116.5815	-23.1	0	K.ILPQEEFNK.K

Spot number: **118**
Mass: 45804 Score: 204 Queries matched: 12
Hypothetical protein WH7805_11873 [Synechococcus sp. WH 7805]

Start	–	End	Observed	Mr(expt)	Mr(calc)	ppm	M	Peptide

Start	–	End	Observed	Mr(expt)	Mr(calc)	ppm	M	Peptide
4	–	15	1320.6550	1319.6477	1319.6259	16.6	0	K.TTAGAPWEQGFR.A
16	–	33	1865.9520	1864.9447	1864.9656	-11.2	1	R.AAVRANAVGWTVSQHNGK.M
20	–	35	1755.8460	1754.8387	1754.8635	-14.1	1	R.ANAVGWTVSQHNGKMR.L
36	–	46	1219.5960	1218.5887	1218.6833	-77.6	1	R.LEHRPPGTGKK.Q
111	–	117	900.4890	899.4817	899.4283	59.4	0	R.DVLMNHR.N + Oxidation (M)
139	–	158	1994.9690	1993.9617	1994.0796	-59.1	1	R.LIDAGQASDGHSLLKGTLAK.W
167	–	183	1926.0200	1925.0127	1924.9723	21.0	1	R.AACCIALRNLTDHAIAR.F
297	–	303	930.4340	929.4267	929.4970	-75.6	0	R.WNLIEQK.H
304	–	319	1732.7830	1731.7757	1731.8329	-33.0	0	K.HAGVLEWPVGNDGEPR.K
304	–	320	1860.9050	1859.8977	1859.9278	-16.2	1	K.HAGVLEWPVGNDGEPRK.L
321	–	332	1449.6900	1448.6827	1448.7888	-73.2	1	K.LDGHHVEVFLKR.Q
368	–	386	2035.9480	2034.9407	2034.9727	-15.7	0	K.IELGAICAAMGHNIEAHGR.A + Oxidation (M)

Spot number: **119**
Mass: 24846 Score: 82 Queries matched: 9
Hypothetical protein OC00_16270, partial [Xanthomonas vasicola]

Start	–	End	Observed	Mr(expt)	Mr(calc)	ppm	M	Peptide
1	–	15	1701.7840	1700.7767	1700.8702	-54.9	1	-.CLPLLMLADARASDR.A
1	–	15	1717.7490	1716.7417	1716.8651	-71.9	1	-.CLPLLMLADARASDR.A + Oxidation (M)
30	–	44	1774.7740	1773.7667	1773.8138	-26.6	0	R.VHCEMDTSQGVQLVR.Q + Oxidation (M)
116	–	121	808.3950	807.3877	807.3408	58.2	0	R.SMWPCK.E
116	–	124	1166.5760	1165.5687	1165.5008	58.2	1	R.SMWPCKEGR.T + Oxidation (M)
125	–	130	806.3660	805.3587	805.4017	-53.4	1	R.TWGMRR.N
138	–	158	2171.1420	2170.1347	2169.9274	95.6	0	R.GCDGEFEVGAEDGSGFVSVPR.M
171	–	181	1233.6070	1232.5997	1232.6296	-24.2	1	R.TCGVSVERAVR.L
202	–	217	1851.9190	1850.9117	1850.8258	46.4	1	R.DGVWVNDGCRAEFIVD.-

Spot number: **120**
Mass: 51734 Score: 77 Queries matched: 6
Branched-chain alpha-keto acid dehydrogenase subunit E2 [[Pseudomonas] cissicola]

Start	–	End	Observed	Mr(expt)	Mr(calc)	ppm	M	Peptide
143	–	170	2717.0740	2716.0667	2716.3086	-89.1	0	R.DDAGTVVGAMQSSNAVQSEQAIAVGGVR.A
239	–	247	801.3690	800.3617	800.4140	-65.4	0	R.AAGIAADGR.D
248	–	264	1636.7780	1635.7707	1635.8329	-38.0	0	R.DRPATAAPLPSAGEGAR.R
265	–	274	943.4870	942.4797	942.4995	-21.0	1	R.RADVGGAAAR.S
457	–	472	1774.7740	1773.7667	1773.8832	-65.7	1	R.ARYQLTPVMGGAETHK.V + Oxidation (M)
484	–	495	1219.5880	1218.5807	1218.6469	-54.3	1	R.AATGGEAARFLR.A

Spot number: **121**
Mass: 7556 Score: 72 Queries matched: 5
Dodecin domain-containing protein [Halomonas smyrnensis]

Start	–	End	Observed	Mr(expt)	Mr(calc)	ppm	M	Peptide
1	–	6	809.2980	808.2907	808.3538	-78.0	0	-.MNHTYK.H + Oxidation (M)
7	–	28	2286.0910	2285.0837	2285.1387	-24.0	0	K.HIELTGSSETSIEDAVQGALTK.A
38	–	52	1851.8920	1850.8847	1850.9176	-17.8	1	R.WFEVIDTRGHIEHGR.V
46	–	52	805.3320	804.3247	804.3991	-92.4	0	R.GHIEHGR.V
46	–	61	1867.8800	1866.8727	1866.9965	-66.3	1	R.GHIEHGRVAHWQVTIK.V

Spot number: **122**
Mass: 55472 Score: 74 Queries matched: 6
Nitrogen metabolism transcriptional regulator, NtrC, Fis family [Paraburkholderia tuberum]

Start	–	End	Observed	Mr(expt)	Mr(calc)	ppm	M	Peptide
109	–	115	856.5100	855.5027	855.5290	-30.7	1	K.AVELIRR.A
116	–	122	807.3680	806.3607	806.3592	1.84	0	R.AVDESMR.G
116	–	130	1794.7800	1793.7727	1793.7639	4.94	1	R.AVDESMRGEQTWDDR.V
292	–	297	832.4620	831.4547	831.3875	80.9	0	R.EDLYHR.L
455	–	469	1765.7120	1764.7047	1764.8134	-61.6	1	R.MLRENAADVMDELAR.R + 2 Oxidation (M)
478	–	486	1064.5840	1063.5767	1063.5410	33.5	1	R.EALDFTRGR.K

Spot number: **123**
Mass: 21161 Score: 80 Queries matched: 6

PadR family transcriptional regulator [Paenibacillus sp. IHBB 10380]

Start	–	End	Observed	Mr(expt)	Mr(calc)	ppm	M	Peptide
1	–	14	1638.8310	1637.8237	1637.8772	-32.6	0	-.MLEYIILGLLMEGK.M + Oxidation (M)
2	–	21	2286.0820	2285.0747	2285.2051	-57.0	1	M.LEYIILGLLMEGKMSGYDLK.K
15	–	21	829.3030	828.2957	828.3687	-88.1	0	K.MSGYDLK.K + Oxidation (M)
22	–	33	1391.7660	1390.7587	1390.7133	32.7	1	K.KTIDSSVGFFYK.A
23	–	33	1263.6780	1262.6707	1262.6183	41.5	0	K.TIDSSVGFFYK.A
172	–	181	1193.5920	1192.5847	1192.5836	0.91	1	R.KELDHVNPNQ.-

Spot number: **124**
Mass: 9362 Score: 75 Queries matched: 4
50S ribosomal protein L23 [Lactobacillus plantarum subsp. plantarum]

Start	–	End	Observed	Mr(expt)	Mr(calc)	ppm	M	Peptide
1	–	7	807.4010	806.3937	806.3480	56.7	0	-.MADLDDK.R
2	–	8	832.4930	831.4857	831.4086	92.7	1	M.ADLDDKR.Y
37	–	43	803.3800	802.3727	802.4371	-80.2	0	K.VNVMNVK.G
75	–	81	879.3660	878.3587	878.3770	-20.8	0	K.LFNDDQQ.-
